# Synthesis of Functionalized Cyclopropanes from Carboxylic Acids by a Radical Addition–Polar Cyclization Cascade

**DOI:** 10.1002/anie.201808598

**Published:** 2018-10-25

**Authors:** Chao Shu, Riccardo S. Mega, Björn J. Andreassen, Adam Noble, Varinder K. Aggarwal

**Affiliations:** ^1^ School of Chemistry University of Bristol, Cantock's Close Bristol BS8 1TS UK

**Keywords:** carboxylic acids, cyclopropanes, decarboxylation, photoredox catalysis, radical–polar crossover

## Abstract

Herein, we describe the development of a photoredox‐catalyzed decarboxylative radical addition–polar cyclization cascade approach to functionalized cyclopropanes. Reductive termination of radical–polar crossover reactions between aliphatic carboxylic acids and electron‐deficient alkenes yielded carbanion intermediates that were intercepted in intramolecular alkylations with alkyl chlorides appended to the alkene substrate. The mild conditions, which make use of a readily available organic photocatalyst and visible light, were demonstrated to be amenable to a broad range of structurally complex carboxylic acids and a wide variety of chloroalkyl alkenes, demonstrating exquisite functional group tolerance.

Over the last decade, photoredox catalysis has been extensively explored.^1^ The mild conditions, high functional group tolerance, and diversity of compatible carbon‐centered radical precursors, has resulted in numerous synthetically valuable methodologies, particularly in the area of alkene functionalization.^2^ The mechanistic pathways of such reactions are dependent on the fate of the open‐shell intermediate generated upon addition of a radical to the alkene substrate, which can undergo a radical‐mediated bond‐forming process (Scheme 1 A, path 1)^3^ or a radical–polar crossover (Scheme 1 A, path 2).^4^, ^5^ In the latter case, a single‐electron transfer (SET) event leads to a carbocation (oxidative termination) or a carbanion (reductive termination), which can then react with a nucleophile or electrophile, respectively. Whilst both processes have been extensively reported, in the case of reductive termination the anion generated has invariably been protonated (Scheme 1 A, path 2, E=H).^5^ We were interested in exploring whether the anion generated could be trapped by a carbon‐based electrophile as this would be much more synthetically useful but has rarely been reported.^6^

**Scheme 1:**
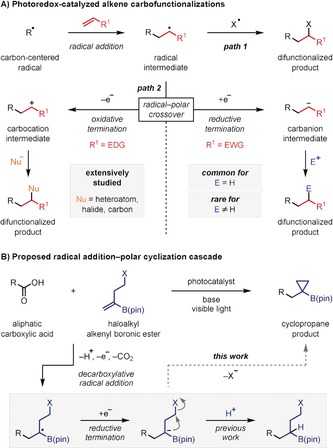
Photoredox‐catalyzed alkene difunctionalizations.

We recently reported a photoredox‐catalyzed decarboxylative radical addition of carboxylic acids to vinyl boronic esters, proceeding through a radical–polar crossover with reductive termination to give an α‐boryl anion.^7^ We queried whether the catalytically‐generated carbanions could undergo intramolecular alkylations with alkyl halides, as this would provide a decarboxylative radical addition–polar cyclization cascade for the synthesis of cyclic boronic esters (Scheme 1 B).^8^ We were particularly interested in targeting cyclopropanes, as these strained carbocycles are highly valuable motifs in drug development and are common components of bioactive natural products.^9^

Herein, we report the successful development of a photoredox‐catalyzed radical–polar crossover cyclopropanation, in which a broad range of readily available carboxylic acids are directly converted to structurally diverse cyclopropanes through decarboxylative reactions with chloroalkyl alkenes. During the preparation of this manuscript, Molander and co‐workers reported a cyclopropanation of styrene derivatives using iodomethyl silicates that proceeds through a radical‐polar crossover with intramolecular alkylation of an intermediate β‐iodo anion.^10^ Our process represents a fragment coupling approach, which is distinct from Molander's, and other, photoredox‐catalyzed cyclopropanations that use carbenoid‐like radicals to introduce a one‐carbon unit in a formal [2+1] cycloaddition.^10^, ^11^

We began our investigation by studying the reaction of (4‐chlorobut‐1‐en‐2‐yl)boronic acid pinacol ester **1 a** (Scheme 2). We were delighted to discover that irradiation of a mixture of **1 a**, Boc‐Pro‐OH (**2**), and cesium carbonate in the presence of the iridium photocatalyst Ir(ppy)_2_(dtbbpy)PF_6_ (**3**) led to the formation of cyclopropyl boronic ester **4 a** in 90 % yield. Analysis of the crude reaction mixture confirmed that no hydrofunctionalization product **5** was formed, which highlights the high rate of intramolecular alkylation of the carbanion intermediate. Remarkably, it was found that 1 mol % of the more economical cyanobenzene‐based organic photocatalyst 4CzIPN (**6**) could promote the reaction with even higher efficiency than **3**, providing cyclopropane **4 a** in quantitative yield.^12^, ^13^

**Scheme 2:**
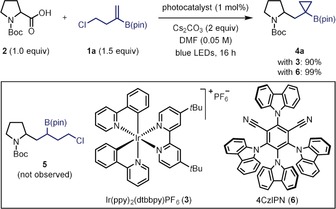
Optimized conditions.

Having identified optimal conditions, we began to investigate the scope of the transformation with respect to other homoallyl chlorides **1** (Table 1). The reaction was found to be amenable to a diverse range of electron‐withdrawing groups, including carboxylate esters (**4 b**), nitriles (**4 c**), primary amides (**4 d**), sulfones (**4 e**), and phosphonate esters (**4 f**), providing the cyclopropane products in excellent yields. The methodology could also be applied to the preparation of vicinally disubstituted cyclopropanes **7** by using allyl chlorides **8**. Boronic ester **7 a** was formed in moderate yield, which was attributed to the formation of allyl ester side‐products by S_N_2 displacement of the chloride of **8 a** by the carboxylate of **2**. For allyl chloride substrates **8**, the enhanced reactivity of the alkyl chloride towards *O*‐alkylation resulted in ester formation becoming competitive with SET and decarboxylation. Switching solvents from DMF to CH_2_Cl_2_ greatly reduced the amount of *O*‐alkylation in the case of carboxylate ester and thioester substrates **8 b** and **8 c**, enabling products **7 b** and **7 c** to be generated in high yields. Cyclopropanes **7 a**–**c** were formed in high diastereoselectivity with respect to the vicinal cyclopropyl substituents but with low stereocontrol at the pyrrolidine stereocenter, which is in keeping with a radical–polar crossover process. The reaction was not limited to the use of homoallyl chlorides bearing electron‐withdrawing groups, as styrene derivatives **1 g**–**j** were also capable substrates, with the stabilized benzylic radical intermediate undergoing efficient single‐electron reduction prior to cyclization. For example, cyclopropanes containing phenyl (**4 g**) and naphthyl (**4 h**) groups were accessed in high yields. Furthermore, the reaction proved to be relatively insensitive to the electronics of the aromatic ring, with homoallyl chlorides functionalized with electron‐deficient pyridines or electron‐rich benzofurans yielding the corresponding heteroaromatic‐substituted cyclopropanes **4 i** and **4 j** in 70 % and 68 % yield, respectively.

**Table 1:** Scope of the chloroalkyl alkenes.^[a]^

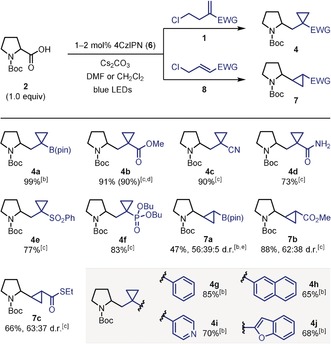

[a] Reactions were performed using 0.3 mmol of **2** and 1.5–2.0 equiv of **1** or **8**. See Supporting Information for exact experimental procedures. Yields are of isolated products. Diastereomeric ratios were determined by ^1^H NMR analysis of the purified products. [b] Solvent=DMF, temperature ca. 50 °C. [c] Solvent=CH_2_Cl_2_, temperature ca. 30 °C (with fan cooling). [d] Number in parenthesis is the yield of isolated product on a 2.0 mmol scale. [e] Using 5 mol % **6** and K_2_CO_3_ as the base.

Next, we turned our attention to determining the generality of the reaction with respect to the carboxylic acid substrate (Table 2). Initially, we focussed on the reaction of α‐amino acids with **1 a** because of the potential for rapid access to cyclopropanated γ‐amino boronic acid derivatives. Further to boronic acid derivatives being highly valuable synthetic handles in organic synthesis,^14^ they are also finding increased application as novel drug candidates.^15^ We also applied the reactions to **1 b** to access cyclopropanated analogues of γ‐amino butyric acid (GABA), the main inhibitory neurotransmitter in the mammalian nervous system.^16^ Structurally diverse α‐amino acids reacted efficiently with **1 a** to yield the corresponding cyclopropyl boronic esters, including those possessing various carbamoyl protecting groups (**9 a**), as well as cyclic (**10 a**) and acyclic (**11 a**) amino acids. By using an increased catalyst loading of 2 mol %, α‐amino acids with a carbamate N−H group also reacted efficiently (**12**–**23**). Increasing the size of the α‐substituent from methyl to *iso*‐propyl to *tert*‐butyl resulted in a gradual decrease in yield (products **12 a**, **13 a** and **14 a**); however, *N*‐Boc‐protected *tert*‐leucine still reacted to give **14 a** in synthetically useful yield. Primary and tertiary α‐amino acids, Boc‐Gly‐OH and Boc‐Aib‐OH, also gave the desired products in reasonable yields (**15 a** and **16 a**). Reaction of these lower yielding substrates with **1 b** led to substantially improved yields of methyl ester cyclopropanes **15 b** and **16 b**. The higher yields obtained with carboxylate ester **1 b** compared to boronic ester **1 a** are in keeping with Carboni's observations that acrylates react more rapidly with alkyl radicals than vinyl boronic esters.^17^ Diversely functionalized amino acid substrates were tolerated, including those with aromatic and heteroaromatic rings, sulfides, esters, and primary amides (products **17 a**–**21 a**, **18 b** and **19 b**). Dipeptides Z‐Gly‐Phe‐OH and Z‐Phe‐Leu‐OH were also viable substrates (products **22 a**, **22 b** and **23 a**), demonstrating the success of this methodology in peptide modification.

**Table 2:** Scope of the carboxylic acids.^[a]^

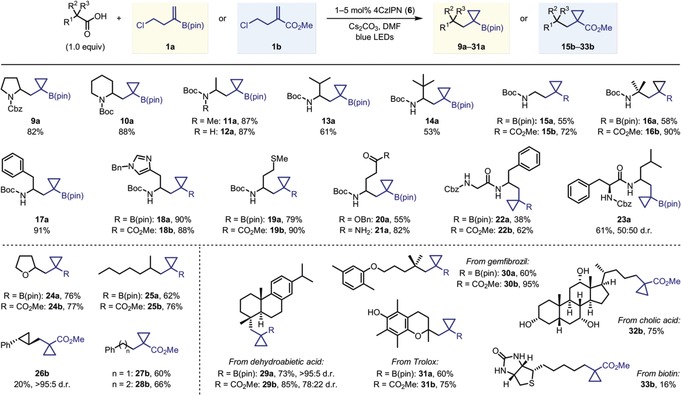

[a] Reactions were performed using 0.3 mmol of the carboxylic acid and 1.2–2.0 equiv of **1 a** or **1 b**. The heat generated by the LEDs resulted in reaction temperatures of ca. 50 °C. See Supporting Information for exact experimental procedures. Yields are of isolated products. Diastereomeric ratios were determined by ^1^H NMR analysis of the purified products.

We then proceeded to investigate a wider range of carboxylic acids. Tetrahydrofuran‐2‐carboxylic acid reacted efficiently with both homoallyl chlorides **1 a** and **1 b**, demonstrating that α‐oxy acids also function well under the optimized conditions (products **24 a** and **24 b**). This was also the case for simple secondary alkyl carboxylic acids, with 2‐methylheptanoic acid yielding cyclopropanes **25 a** and **25 b** in good yields. Bis‐cyclopropane product **26 b** could also be prepared upon reaction of a cyclopropyl carboxylic acid with **1 b**. The poor yield of product **26 b** reflects the challenges associated with the generation and subsequent addition reactions of cyclopropyl radicals.^18^ Indeed, attempted reaction of the same cyclopropyl carboxylic acid with the less reactive alkenyl boronic ester **1 a** 17 only resulted in recovery of unreacted starting materials. Similarly, primary alkyl carboxylic acids, including benzylic and non‐benzylic, were found to be unreactive with **1 a** but reacted efficiently with **1 b** to give good yields of cyclopropanes **27 b** and **28 b**.

We then targeted more complex carboxylic acids as substrates for our reaction, with the aim of demonstrating its potential as a valuable method for late‐stage diversification of bioactive natural products and drug molecules. To this end, dehydroabietic acid underwent efficient reaction with both **1 a** and **1 b** to give the **29 a** and **29 b** in high yields and good levels of diastereocontrol. Similarly, other tertiary alkyl carboxylic acids, including the phenol‐containing vitamin E analogue Trolox and the fibrate drug gemfibrozil, provided the cyclopropane products **30 a**, **30 b**, **31 a** and **31 b** in good to excellent yields. Furthermore, the densely functionalized naturally‐occurring primary alkyl carboxylic acids cholic acid and biotin reacted with **1 b** to give the structurally complex cyclopropane products **32 b** and **33 b**, respectively.

To probe the generality of this cyclization reaction, we also explored the formation of larger rings using haloalkyl alkenes **34**, **35**, and **36** (Table 3). Of the substrates explored, only cyclopentane formation was successful and optimum results were obtained using iodide **35 c** (entry 6). Reactions with haloalkyl alkenes **34** and **36** gave the protonated Giese addition products **40** and **42** instead of the 4‐ and 6‐membered rings. The failure to generate cyclobutane **37** and cyclohexane **39** reflects the significantly slower rates of 4‐ and 6‐*exo*‐*tet* cyclization compared to 3‐ and 5‐*exo*‐*tet*, which results in protonation to form **40** or **42** becoming the dominant pathway.^19^

**Table 3:** Formation of larger rings.^[a]^ 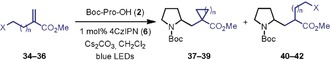

Entry	Substrate	*n*	X	Cyclized product, yield	Protonated product, yield
1	**34 a**	2	Cl	**37**, 0 %	**40 a**, 65 %
2	**34 b**	2	Br	**37**, 0 %	**40 b**, 62 %
3	**34 c**	2	I	**37**, trace^[b]^	**40 c**, 36 %
4	**35 a**	3	Cl	**38**, 10 %	**41 a**, 76 %
5	**35 b**	3	Br	**38**, 41 %	**41 b**, 25 %
6	**35 c**	3	I	**38**, 86 %	**41 c**, trace^[b]^
7	**36 b**	4	Br	**39**, trace^[b]^	**42 b**, 91 %
8	**36 c**	4	I	**39**, trace^[b]^	**42 c**, 84 %

[a] Reactions were performed using 0.2 mmol of **2** and 2.0 equiv of **34**–**36**. Yields are of isolated products. [b] A trace amount was observed in the crude product mixture.

To gain insight into the mechanism of this radical cascade process, we conducted several experiments to elucidate the presence or absence of radical and carbanion intermediates (Scheme 3). Reaction of *cis*‐pinonic acid, a cyclobutane‐containing primary alkyl carboxylic acid, generated the ring‐opened product **43**, confirming the initial formation of a primary carbon‐centered radical, which undergoes ring‐opening to generate the more stable tertiary radical prior to reaction with **1 b** (Scheme 3 A). The formation of carbanion intermediates was confirmed by submitting allyl acetate **44** to the standard reaction conditions, during which elimination of the acetate group occurred to give alkene **45** in excellent yield (Scheme 3 B). Elimination of the acetate group will only take place after reduction of the intermediate α‐carbonyl radical to the carbanion (enolate).^20^ Although this result supports a radical–polar crossover mechanism, a possible alternative pathway during the cyclopropanation step is a homolytic substitution reaction (S_H_2) between the intermediate α‐carbonyl radical and the alkyl halide, a process that has previously been reported for radical cyclopropanations with alkyl iodides.11b,11c While this alternative mechanism is unlikely given that chlorine is a much poorer radical leaving group than iodine,^21^ we gained conclusive evidence for a polar (S_N_2) cyclopropanation by carrying out the reaction of **2** with homoallyl tosylates **46 a** and **46 b**, which gave cyclopropanes **4 a** and **4 b** in comparable yields to those obtained with the homoallyl chlorides (Scheme 3 C). In a related study forming 3‐membered rings, Molander and co‐workers compared the activation barriers of radical and anionic cyclizations with different leaving groups and also concluded that the cyclization occurred through an anionic pathway, thereby supporting our observations.^10^

**Scheme 3:**
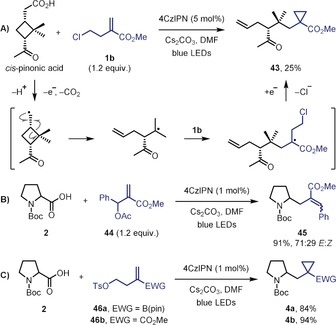
Mechanistic studies.

Based on these observations, we propose the mechanism outlined in Scheme 4. Initially, visible‐light irradiation of the photocatalyst (**PC**) leads to generation of the excited state catalyst (PC*). SET with the carboxylate generated from in situ deprotonation of carboxylic acid **2** results in reduction of the photocatalyst, to radical anion **PC^.−^**, and oxidation of the carboxylate to a carboxyl radical. Extrusion of CO_2_ provides carbon‐centered radical **47** that undergoes addition to homoallyl chloride **1** to form the stabilized alkyl radical **48**. A second SET between radical **48** and the **PC^.−^** state of the photocatalyst completes the photoredox catalytic cycle and results in reductive termination of the radical process. Finally, the resulting stabilized carbanion **49** undergoes a polar 3‐*exo*‐*tet* cyclization to give the cyclopropane product **4**. Measurement of a quantum yield (*Φ*) of 0.65 for the reaction of Boc‐Pro‐OH (**2**) with alkenyl boronic ester **1 a** suggests that alternative radical chain mechanisms are not operative, thus providing further support for the closed photoredox catalytic cycle shown in Scheme 4. The remarkable breadth of homoallyl chloride substituents that could be employed is especially noteworthy, ranging from strong electron‐withdrawing groups (such as carboxylate esters and nitriles) all the way to electron‐rich aromatics (for example benzofuran). This highlights the broad synthetic utility of 4CzIPN, as the reduced photocatalyst **PC^.−^** (*E*_1/2_ [4CzIPN/4CzIPN^.−^]=−1.21 V vs. SCE in MeCN)12b was able to undergo SET with carbon‐centered radicals of vastly different reduction potentials, including α‐carboxylate ester radicals (*E*_1/2_ [R^.^/R^−^]≈−0.6 V vs. SCE in MeCN)^22^ and electron‐rich benzylic radicals (*E*_1/2_ [R^.^/R^−^] < −1.4 V vs. SCE in MeCN).^23^

**Scheme 4:**
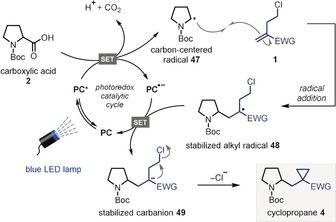
Proposed mechanism.

In conclusion, we have developed a photoredox‐catalyzed cyclopropane synthesis proceeding through a decarboxylative radical addition–polar cyclization cascade. The mild conditions, catalyzed by an organic photocatalyst, were found to tolerate a broad range of functional groups on both the carboxylic acid and the chloroalkyl alkenes substrates. Mechanistic studies confirmed that the reaction proceeds by a radical–polar crossover mechanism involving reductive termination and subsequent alkylation of a carbanion intermediate. Given the abundance of carboxylic acids in sustainable chemical feedstocks, we believe that this new fragment coupling‐based cyclopropanation reaction provides a valuable, atom‐economical methodology for the expedient preparation of structurally diverse cyclopropanes.

## Conflict of interest

The authors declare no conflict of interest.

## Supporting information

As a service to our authors and readers, this journal provides supporting information supplied by the authors. Such materials are peer reviewed and may be re‐organized for online delivery, but are not copy‐edited or typeset. Technical support issues arising from supporting information (other than missing files) should be addressed to the authors.

Supplementary
