# Supplementary material for: Synthesis of Functionalized Cyclopropanes from Carboxylic Acids by a Radical Addition–Polar Cyclization Cascade
Source: Angew Chem Int Ed Engl. 2018 Oct 25;57(47):15430–4. doi: 10.1002/anie.201808598 (PMC6282618; doi:10.1002/anie.201808598)

Angewandte Chemie
Eine Zeitschrift der Gesellschaft Deutscher Chemiker

Supporting Information

Synthesis of Functionalized Cyclopropanes from Carboxylic Acids by a Radical Addition–Polar Cyclization Cascade

Chao Shu[+], Riccardo S. Mega[+], Björn J. Andreassen, Adam Noble, and Varinder K. Aggarwal**

anie_201808598_sm_miscellaneous_information.pdf

Contents

1. General Information ... 2

1.1. Solvents, Reagents and Starting Materials ... 2

1.2. Chromatography and Instrumentation ... 2

1.3. Photochemical Equipment and Setup ... 3

2. Optimisation Studies and Control Reactions ... 4

3. General Procedures ... 5

4. Synthesis of Starting Materials ... 7

4.1. Synthesis of Homoallyl Chlorides ... 7

4.2. Synthesis of Allyl Chlorides ... 14

4.3. Synthesis of Styrene Derivatives ... 15

5. Product Characterisation ... 20

6. Scale-up Reaction ... 54

7. Studies into the Formation of Larger Rings ... 55

7.1. Substrate Synthesis ... 55

7.2. Attempted Cyclobutane Synthesis ... 61

7.3. Cyclopentane Synthesis ... 64

7.4. Attempted Cyclohexane Synthesis ... 66

8. Mechanistic Studies ... 68

8.1. Ring Opening of Pinonic Acid ... 68

8.2. Reaction of Allyl Acetate Substrate ... 69

8.3. Synthesis and Reactions of Homoallyl Tosylates ... 71

8.4. Quantum Yield Measurement ... 74

9. References ... 77

10. NMR Spectra ... 78

1. General Information

1.1. Solvents, Reagents and Starting Materials

All reagents were used as rece ived unless otherwise stated. Water is de-ionised and brine refers to a saturated aqueous solution of NaCl. DMF was anhydrous, purchased from Sigma-Aldrich and used as received. Dichloromethane (CH_2Cl_2) was anhydrous (purification using a column composed of activated alumina). 4CzIPN (**6**) was prepared following the method of Zhang and co-workers.[1]

1.2. Chromatography and Instrumentation

Flash column chromatography was carried out using silica gel (Aldrich, silica gel 60, 40-63 μm). Analytical thin-layer chromatography (TLC) was performed using aluminium-backed silica plates (0.25 mm, Merck, silica gel 60 F254). Compounds were visualised under UV light or by staining with aqueous basic potassium permanganate, an ethanolic solution of phosphomolybdic acid (PMA), or an ethanolic solution of ninhydrin.

^{1}H, ^{13}C and ^{11}B NMR spectra were acquired at various field strengths, as indicated, using Bruker 400 MHz, Varian VNMR 400 MHz, Varian VNMR 500 MHz, and Bruker Cryo 500 MHz spectrometers. All NMR spectra were recorder at 25 °C unless otherwise stated. Chemical shifts (δ) are given in parts per million (ppm) and referenced to $CDCl_3$ (^{1}H: 7.26 ppm) or DMSO-*d*$_6$ (^{1}H: 2.50 ppm). Coupling constants (*J*) are given in Hertz (Hz) and refer to apparent multiplicities (s = singlet, br. s = broad singlet, d = doublet, t = triplet, q = quartet, quin = quintet, sex = sextet, h = heptet, m = multiplet, dd = doublet of doublets, etc.). The ^{1}H NMR spectra are reported as follows: chemical shift (multiplicity, coupling constants, number of protons).

Gas chromatography (GC) was performed on an Agilent Technologies 6890N Network GC System using an Agilent HP-5 column (15 m × 0.25 mm × 0.25 μm).

High-resolution mass spectra (HRMS) were recorded on a Bruker micrOTOF instrument using electrospray ionisation (ESI). Low-resolution mass spectra (LRMS) were recorded on an Agilent 7820A GC-MS equipped with a HP-5MS UI column (30 m x 0.25 mm x 0.25 μm) using electron ionisation (EI).

Infra-red (IR) spectra were recorded on a Perkin Elmer Spectrum One FT-IR spectrometer as a thin film. Selected absorption maxima (ν_{max}) are reported in wavenumbers (cm^{-1}).

Melting points were recorded in degrees Celsius (°C), using a Kofler hot-stage microscope apparatus and are reported uncorrected.

Optical rotation ($[\alpha]_D^T$) was measured on a Bellingham and Stanley Ltd. ADP220 polarimeter and is quoted in (° mL)(g dm)$^{-1}$.

For quantum yield experiments, commercially available potassium ferrioxalate trihydrate (Alfa Aesar) was used for actinometry, and all the absorption spectra were measured using a Perkin Elmer Lambda 25 UV/Vis Spectrophotometer.

1.3. Photochemical Equipment and Setup

The blue LED lamps were either 40 W Kessil A160WE Tuna Blue LED Aquarium Lights (used with the colour dial turned fully anticlockwise and the intensity dial turned fully clockwise) or 40 W Kessil PR160-427 nm LED Photoredox Lights (used with the intensity dial set to 100).

During the course of the photoredox reactions, heat generated from the LED lamps resulted in warming of the reaction mixtures to approximately 50 ºC. For reactions performed in dichloromethane (General Procedure B), fan cooling was used to maintain a temperature of 25–30 °C.

Reaction set-up:

The reaction vials were positioned 5 cm from a single 40 W Kessil LED lamp (Figure S1).

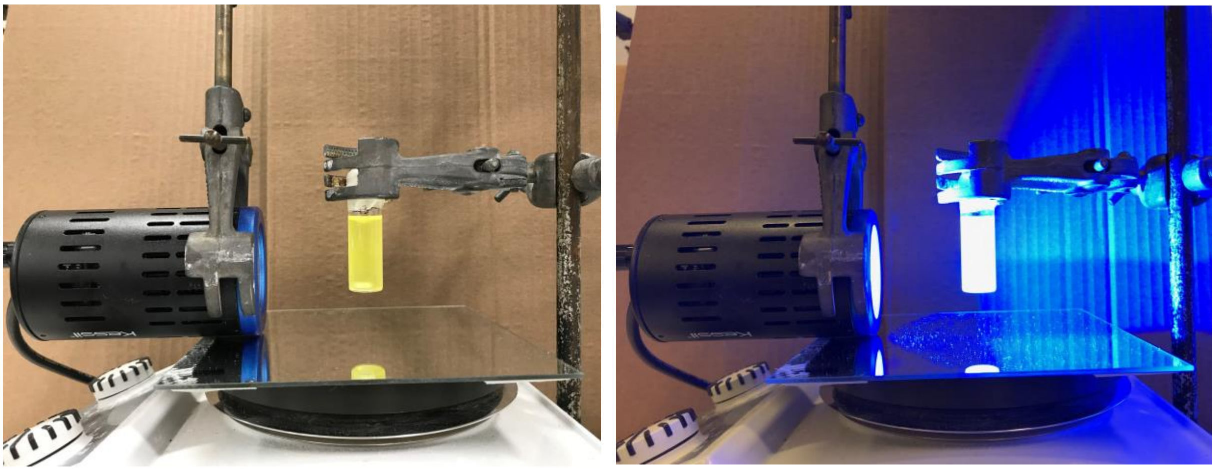

Figure S1. Photoredox reaction setup.

2. Optimisation Studies and Control Reactions

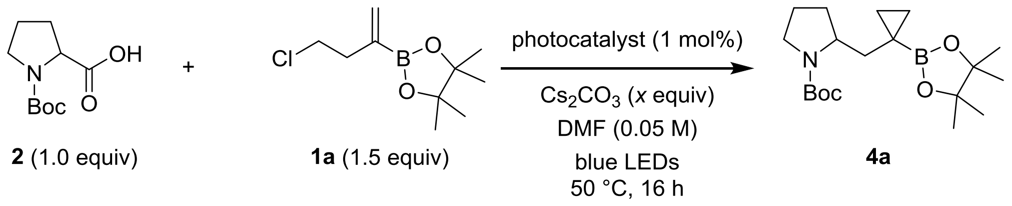

2 (1.0 equiv) **1a** (1.5 equiv) **4a**

entry	photocatalyst	Cs_2CO_3 equiv	**4a** (%)
1	$Ir(ppy)_2(dtbbpy)PF_6$ (**3**)	1.0	76
2	$Ir(ppy)_2(dtbbpy)PF_6$ (**3**)	2.0	90
3	4CzIPN (**6**)	2.0	99
4	4CzIPN (**6**)	-	2
5	none	2.0	3
6[a]	4CzIPN (**6**)	2.0	0

Table S1. Optimisation Studies and Control Reactions.

All reactions were performed on a 0.1 mmol scale. Yields were determined by GC analysis using an internal standard. [a]Reaction performed in the dark.

3. General Procedures

General Procedure A (for reactions of carboxylic acids with homoallyl chlorides **1**):

To a 7 mL vial equipped with a magnetic stir bar was added the carboxylic acid (1.0 equiv.), 4CzIPN (1.0–2.0 mol%) and Cs_2CO_3 (2.0 equiv.). Anhydrous DMF (0.05 M) was then added followed by the homoallyl chloride (1.5–2.0 equiv.). The vial was sealed with a septum and the reaction mixture degassed by sparging with nitrogen for 10 min. The nitrogen inlet was removed, and the vial further sealed with parafilm. The reaction mixture was stirred at 800 rpm and irradiated with a 40 W Kessil LED lamp for 15–62 h. The reaction mixture was diluted with EtOAc (20 mL), washed with H_2O (3 × 20 mL), brine (20 mL), dried (Na_2SO_4), filtered, and concentrated *in vacuo*. The crude product was then purified by flash column chromatography.

General Procedure B (for reactions of carboxylic acids with allyl chlorides **8b** and **8c**, and homoallyl chlorides **1b**–**1f**):

To a 7 mL vial equipped with a magnetic stir bar was added the carboxylic acid (1.0 equiv.), 4CzIPN (1.0 mol%) and Cs_2CO_3 (2.0 equiv.). Anhydrous CH_2Cl_2 (0.05 M) was then added followed by the alkenyl chloride (2.0 equiv.). The vial was sealed with a septum and the reaction mixture degassed by sparging with nitrogen for 10 min. The nitrogen inlet was removed, and the vial further sealed with parafilm. The reaction mixture was stirred at 800 rpm and irradiated with a 40 W Kessil LED lamp with fan cooling for 15 h. The organic phase was washed with H_2O (20 mL), brine (20 mL), dried ($MgSO_4$), filtered, and concentrated *in vacuo*. The crude product was then purified by flash column chromatography.

General Procedure C (for reactions of primary and secondary alkyl carboxylic acids with homoallyl chloride **1b**):

To a 7 mL vial equipped with a magnetic stir bar was added the carboxylic acid (1.0 equiv.), 4CzIPN (5.0 mol%) and Cs_2CO_3 (2.0 equiv.). Anhydrous DMF (0.05 M) was then added followed by the homoallyl chloride (1.2 equiv.). The vial was sealed with a septum and the reaction mixture degassed by sparging with nitrogen for 10 min. The nitrogen inlet was removed, and the vial further sealed with parafilm. The reaction mixture was stirred at 800 rpm and irradiated with a 40 W Kessil LED lamp for 20 h. The reaction mixture was diluted with H_2O (20 mL) and extracted into EtOAc (3 × 20 ml). The combined organic phases were washed with H_2O (20 mL), brine (20 mL), dried (Na_2SO_4), filtered, and concentrated *in vacuo*. The crude product was then purified by flash column chromatography.

Note on base equivalents:

During our investigations into the scope of the reaction, we found that the use of 2.0 equiv. of Cs_2CO_3 was not always necessary to obtain the desired products in high yield, with 1.0 equiv. giving similar yields. However, using the increased amount of base did prove beneficial with some substrates, therefore, 2.0 equiv. of Cs_2CO_3 was used as standard.

4. Synthesis of Starting Materials

4.1. Synthesis of Homoallyl Chlorides

2-(4-Chlorobut-1-en-2-yl)-4,4,5,5-tetramethyl-1,3,2-dioxaborolane (**1a**)

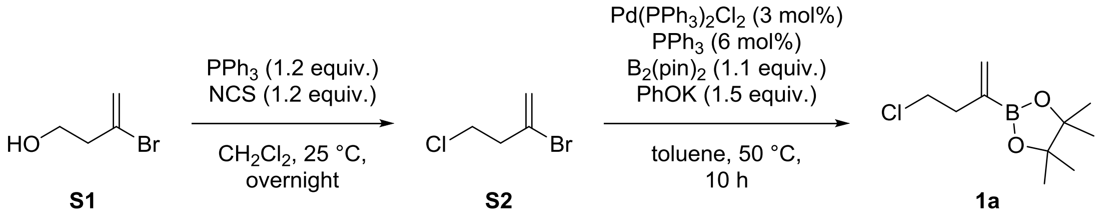

S1 **S2** **1a** 74%

Alkenyl bromide **S2** was prepared from commercially available alcohol **S1** using a literature procedure.[2]

Alkenyl boronic ester **1a** was prepared following a modified literature procedure:[3] $PdCl_2(PPh_3)_2$ (126 mg, 0.180 mmol, 3.00 mol%), Ph_3P (96 mg, 0.36 mmol, 6.0 mol%), bis(pinacolato)diboron (1.67 g, 6.60 mmol, 1.10 equiv.), and PhOK (fine powder, 1.19 g, 9.00 mmol, 1.50 equiv.) were added to a flask equipped with a magnetic stir bar, a septum inlet, and a condenser. The flask was flushed with nitrogen and then charged with toluene (36 mL) and **S2** (1.02 g, 6.00 mmol, 1.00 equiv.). The mixture was then stirred at 50 °C for 10 h. The reaction mixture was treated with H_2O (20 mL) at r.t., extracted into Et_2O (3 × 20 mL), washed with brine (20 mL), dried ($MgSO_4$), filtered, and concentrated *in vacuo*. The residue was purified by flash column chromatography (5% Et_2O/pentane) to give **1a** (955 mg, 4.41 mmol, 74%) as a colourless liquid.

TLC: R_f = 0.45 (5% Et_2O/pentane, $KMnO_4$ stain).

^{1}H NMR (400 MHz, $CDCl_3$): δ_H 5.91 (d, *J* = 3.2 Hz, 1H), 5.72 (d, *J* = 3.1 Hz, 1H), 3.62 (t, *J* = 7.3 Hz, 2H), 2.61 (t, *J* = 7.3 Hz, 1H), 1.26 (s, 12H) ppm.

^{13}C NMR (101 MHZ, $CDCl_3$): δ_C 132.6, 83.8, 44.3, 38.9, 24.9 ppm. The carbon directly attached to boron was not detected due to the boron quadrupole.

^{11}B NMR (128 MHz, $CDCl_3$): δ_B 30.1 (br. s, 1B) ppm.

IR (film) ν_{max}: 2920, 2851, 1739, 1464, 1372, 1314, 1243, 1145, 1022 cm^{-1}.

HRMS (ESI^+) calcd. for $C_{10}H_{18}BClNaO_2$ $[M+Na]^+$ 239.0982, found 239.0987.

An alternative route to **1a** was also developed starting from but-3-yn-1-ol (**S3**):

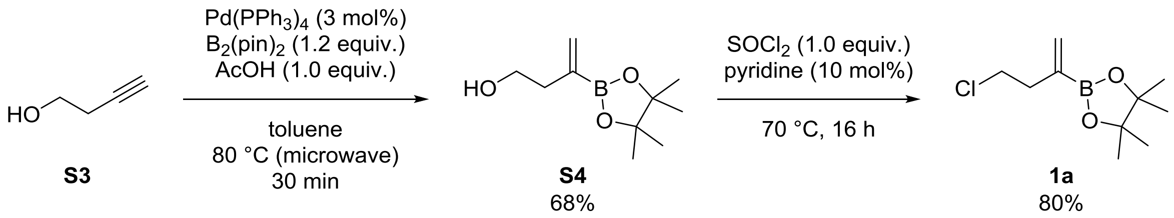

A microwave vial containing a magnetic stir bar was charged with $Pd(PPh_3)_4$ (0.32 g, 0.28 mmol, 3.0 mol%) and bis(pinacolato)diboron (2.81 g, 11.1 mmol, 1.20 equiv.). The vial was evacuated and back-filled with nitrogen three times. Toluene (18.5 mL) was added, followed by but-3-yn-1-ol (**S3**) (758 mg, 9.23 mmol, 1.00 equiv.) and acetic acid (571 mg, 9.23 mmol, 1.00 equiv.). The reaction mixture was prestirred for 5 min before heating to 80 °C for 30 min in the microwave. After allowing to cool to r.t., the mixture was concentrated *in vacuo* and the residue purified by flash column chromatography (20% EtOAc/hexane) to give the 3-(4,4,5,5-tetramethyl-1,3,2-dioxaborolan-2-yl)but-3-en-1-ol (**S4**) (1.25 g, 6.28 mmol, 68%) as a colourless oil.

To an oven-dried flask was added 3-(4,4,5,5-tetramethyl-1,3,2-dioxaborolan-2-yl)but-3-en-1-ol (**S4**) (1.03 mL, 5.05 mmol, 1.00 equiv.) and pyridine (40 μL, 0.50 mmol, 0.10 equiv.) and the mixture was cooled to 0 °C. Thionyl chloride (0.370 mL, 5.05 mmol, 1.00 equiv.) was added dropwise before the reaction mixture was heated to 70 °C for 16 h. After cooling to r.t., the reaction mixture was concentrated in vacuo and purified by flash column chromatography (2% EtOAc/pentane) to give **1a** (877 mg, 4.05 mmol, 80%) as a pale yellow liquid.

Methyl 4-chloro-2-methylenebutanoate (1b)

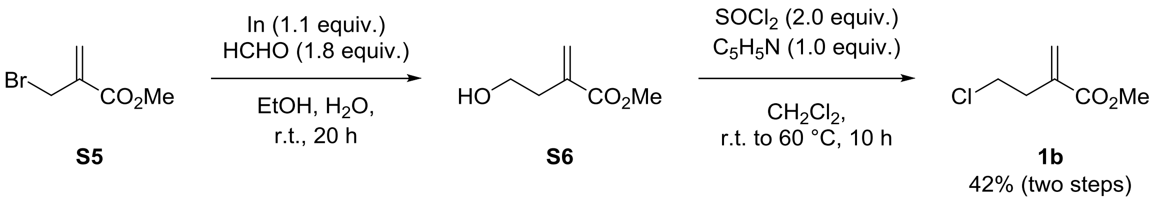

Methyl 4-chloro-2-methylenebutanoate (**1b**) was prepared following a modified literature procedure:[4] To a flask equipped with a magnetic stir bar was added methyl 2-(bromomethyl) acrylate (**S5**) (1.0 g, 5.6 mmol, 1.00 equiv). H_2O (10 mL) and EtOH (10 mL) were then added with vigorous stirring, followed by formaldehyde (0.300 g, 10.1 mmol, 1.80 equiv.) and indium powder (0.71 g, 6.2 mmol, 1.1 equiv.). The reaction mixture was stirred vigorously for 20 h, then partitioned between CH_2Cl_2 (50 mL) and H_2O (50 mL). The phases were separated and the aqueous phase was extracted into CH_2Cl_2 (3 × 25 mL). The combined organic phases were washed with brine (50 mL), dried ($MgSO_4$), filtered, and

concentrated *in vacuo*. The residue was purified by flash column chromatography (20% EtOAc/hexane) to give methyl 4-hydroxy-2-methylenebutanoate (**S6**) (510 mg, 3.92 mmol, 70%) as a pale yellow oil.

To a stirred solution of methyl 4-hydroxy-2-methylenebutanoate (**S6**) (510 mg, 3.92 mmol, 1.00 equiv.) in CH_2Cl_2 (20 mL) under N_2 at 0 °C was added dry pyridine (0.4 mL) followed by dropwise addition of $SOCl_2$ (0.75 mL, 10 mmol, 2.6 equiv.). The resulting solution was heated to reflux for 10 h, after which the reaction mixture was cooled to r.t. before diluting with Et_2O (30 mL) and slowly adding H_2O (20 mL). The phases were separated, and the aqueous phase was extracted into Et_2O (3 × 30 mL). The organic extracts were combined, washed with H_2O (30 mL) and brine (30 mL), then dried ($MgSO_4$), filtered, and concentrated *in vacuo*. The residue was purified by flash column chromatography (10% Et_2O/pentane) to give **1b** (348 mg, 2.35 mmol, 60%) as a colourless oil.

TLC: R_f = 0.50 (10% Et_2O/pentane, $KMnO_4$ stain).

^{1}H NMR (400 MHz, $CDCl_3$): δ_H 6.29 (d, *J* = 1.2 Hz, 1H), 5.70 (d, *J* = 1.1 Hz, 1H), 3.76 (s, 3H), 3.66 (t, *J* = 6.8 Hz, 2H), 2.79 – 2.73 (m, 2H) ppm.

^{13}C NMR (101 MHz, $CDCl_3$): δ_C 166.8, 136.2, 128.2, 52.0, 42.8, 35.4 ppm.

IR (film) ν_{max}: 3000, 2954, 1715, 1632, 1438, 1320, 1290, 1170, 1196, 1126 cm^{-1}.

HRMS (ESI$^+$) calcd. for $C_6H_9ClNaO_2$ $[M+Na]^+$ 171.0183, found 171.0180.

4-Chloro-2-methylenebutanenitrile (1c)

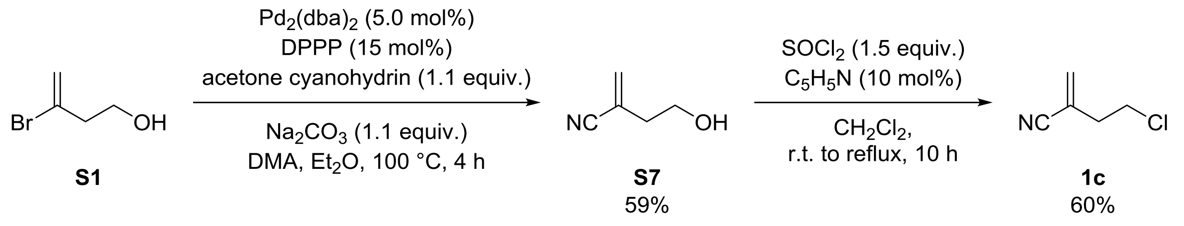

4-Hydroxy-2-methylenebutanenitrile (**S7**) was prepared following a modified literature procedure:[5] A solution of 3-bromobut-3-en-1-ol (**S1**) (166 mg, 1.10 mmol, 1.00 equiv.), $Pd_2(dba)_3$ (50 mg, 0.054 mmol, 5.0 mol%), and 1,3-bis(diphenylphosphino)propane (68 mg, 0.17 mmol, 15 mol%) in Et_2O (2 mL) was stirred at room temperature for 15 min. Sodium carbonate (128 mg, 1.20 mmol, 1.10 equiv.) and DMA (1.0 mL) were then added and the mixture was heated to 100 °C. Acetone cyanohydrin (1.1 mL, 0.60 mmol, 1.1 equiv.) was then added in one portion and the mixture stirred at 100 °C for 4 h. After cooling to r.t., H_2O (15 mL) was added the mixture extracted into CH_2Cl_2 (3 × 20 mL). The combined organic layers were dried ($MgSO_4$), filtered, and concentrated *in vacuo*. The residue was purified by flash

column chromatography (30% EtOAc/hexane) to give the 4-hydroxy-2-methylenebutanenitrile (**S7**) (63 mg, 0.65 mmol, 59%) as a yellow oil.

To a stirred solution of 4-hydroxy-2-methylenebutanenitrile (**S7**) (260 mg, 2.68 mmol, 1.00 equiv.) in CH_2Cl_2 (20 mL) under N_2 at 0 °C was added dry pyridine (21 mg, 0.27 mmol, 10 mol%) followed by dropwise addition of $SOCl_2$ (488 mg, 4.10 mmol, 1.53 equiv.). The resulting solution was heated to reflux for 6 h, after which the reaction mixture was cooled to 0 °C before diluting with Et_2O (30 mL) and slowly adding H_2O (20 mL). The phases were separated, and the aqueous phase was extracted into Et_2O (3 × 30 mL). The organic extracts were combined, washed with H_2O (20 mL) and brine (20 mL), then dried ($MgSO_4$), filtered, and concentrated *in vacuo*. The residue was purified by flash column chromatography (50% Et_2O/pentane) to give desired product **1c** (187 mg, 1.62 mmol, 60%) as a colourless oil.

TLC: R_f = 0.25 (10% Et_2O/pentane, $KMnO_4$ stain).

^{1}H NMR (400 MHz, $CDCl_3$): δ_H 5.99 (t, *J* = 0.8 Hz, 1H), 5.85 (t, *J* = 1.4 Hz, 1H), 3.67 (t, *J* = 6.5 Hz, 2H), 2.68 (tdd, *J* = 6.5, 1.4, 0.8 Hz, 2H) ppm.

^{13}C NMR (101 MHz, $CDCl_3$): δ_C 133.3, 119.0, 117.6, 40.9, 37.2 ppm.

IR (film) ν_{max}: 2969, 2874, 1734, 1625, 1431, 1373, 1243, 1045, 944, 910 cm^{-1}.

LRMS (EI^+): m/z 115 (M^+ [C_5H_6ClN], 9), 85 (100), 71 (41).

4-Chloro-2-methylenebutanamide (1d)

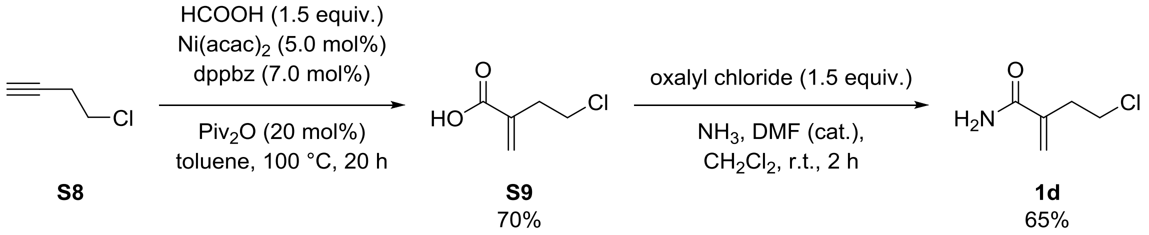

4-Chloro-2-methylenebutanoic acid (**S9**) was prepared following a modified literature procedure:[6] A Schlenk tube containing a magnetic stir bar was charged with $Ni(acac)_2$ (64 mg, 0.25 mmol, 5.0 mol%) and 1,2-bis(diphenylphosphino)benzene (156 mg, 0.350 mmol, 7.00 mol%). The tube was evacuated and back-filled with N_2 three times. Toluene (15 mL) was added, followed by 4-chlorobut-1-yne (**S8**) (443 mg, 5.00 mmol, 1.00 equiv.), formic acid (345 mg, 7.50 mmol, 1.50 equiv.) and pivalic anhydride (187 mg, 1.00 mmol, 20.0 mol%). The reaction mixture was heated to 100 °C for 24 h before cooling to r.t. and concentrating *in vacuo*. The residue was purified by flash column chromatography (50%

EtOAc/hexane) to give the 4-chloro-2-methylenebutanoic acid (**S9**) (471 mg, 3.50 mmol, 70%) as a colourless oil.

4-Chloro-2-methylenebutanamide (**1d**) was prepared following a modified literature procedure:[7] Oxalyl chloride (673 mg, 5.30 mmol, 1.51 equiv.) was added to a solution of 4-chloro-2-methylenebutanoic acid (**S9**) (471 mg, 3.50 mmol, 1.00 equiv.) in dichloromethane (30 mL) at r.t. To the solution was added a few drops of DMF. The reaction was continued for 1.5 h before cooling to 0 °C and adding 25-30% wt. ammonium hydroxide (10 mL). The organic solvent was removed *in vacuo*, and the resulting residue was purified by flash column chromatography (50% EtOAc/hexane) to give 4-chloro-2-methylenebutanamide (**1d**) (240 mg, 2.28 mmol, 65%) as a colourless oil.

TLC: R_f = 0.20 (50% EtOAc/hexane, $KMnO_4$ stain).

^{1}H NMR (400 MHz, $CDCl_3$): δ_H 5.81 (br. s, 2H), 5.75 (s, 1H), 5.53 (s, 1H), 3.68 (t, J = 6.6 Hz, 2H), 2.78 (t, J = 6.6 Hz, 2H) ppm.

^{13}C NMR (101 MHz, $CDCl_3$): δ_C 169.8, 140.6, 121.4, 42.9, 35.6 ppm.

IR (film) ν_{max}: 3368, 3181, 2972, 2913, 1659, 1599, 1435, 1406, 1302, 1107 cm^{-1}.

HRMS (ESI$^+$) calcd. for $C_5H_8ClNNaO$ $[M+Na]^+$ 156.0178, found 156.0186.

((4-Chlorobut-1-en-2-yl)sulfonyl)benzene (**1e**)

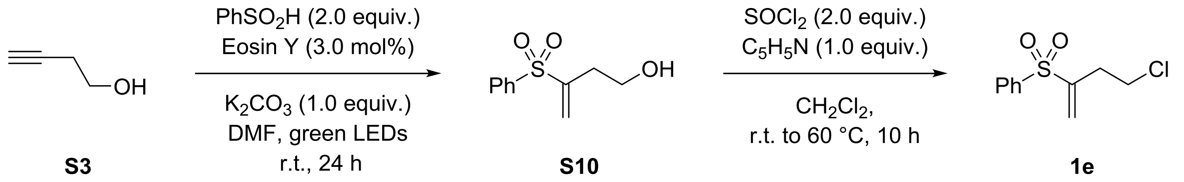

S3 **S10** **1e**
34% (2 steps)

3-(Phenylsulfonyl)but-3-en-1-ol (**S10**) was prepared following a modified literature procedure:[8] A mixture of but-3-yn-1-ol (**S3**) (105 mg, 1.50 mmol, 1.00 equiv.), benzenesulphinic acid (426 mg, 3.00 mmol, 2.00 equiv.), K_2CO_3 (487 mg, 1.50 mmol, 1.00 equiv.), eosin Y (29 mg, 0.045 mmol, 3.0 mol%) in degassed dry DMF (15 mL) was stirred under a nitrogen atmosphere and irradiated with green LEDs for 24 h. H_2O (50 mL) was added and the mixture was extracted into EtOAc (3 × 20 mL). The combined organic phases were dried ($MgSO_4$), filtered, and concentrated *in vacuo*. The residue was purified by flash column chromatography (40% EtOAc/hexane) to afford sulfone **S10** (153 mg, 0.720 mmol, 48%) as a yellow oil.

Homoallyl chloride **1e** was then prepared following the same chlorination procedure as described in the synthesis of **1b**, with alcohol **S10** (140 mg, 0.660 mmol, 1.00 equiv.), pyridine (52 mg, 0.66 mmol, 1.0

equiv.), and thionyl chloride (157 mg, 1.32 mmol, 2.00 equiv.). The crude product was purified by flash column chromatography (20% EtOAc/hexane) to afford **1e** (108 mg, 0.469 mmol, 71%) as a yellow oil.

TLC: R_f = 0.25 (20% EtOAc/pentane, $KMnO_4$ stain).

^{1}H NMR (400 MHz, $CDCl_3$): δ_H 7.89 – 7.83 (m, 2H), 7.67 – 7.60 (m, 1H), 7.58 – 7.52 (m, 2H), 6.46 (d, *J* = 0.8 Hz, 1H), 5.90 (dt, *J* = 1.2, 1.0 Hz, 1H), 3.60 (t, *J* = 6.9 Hz, 2H), 2.68 (td, *J* = 6.8, 1.3 Hz, 2H) ppm.

^{13}C NMR (101 MHz, $CDCl_3$): δ_C 146.4, 138.3, 133.7, 129.3, 128.1, 126.3, 41.5, 32.8 ppm.

IR (film) ν_{max}: 3064, 2968, 1633, 1584, 1478, 1446, 1303, 1144, 1080, 956 cm^{-1}.

HRMS (ESI$^+$) calcd. for $C_{10}H_{11}ClNaO_2S$ [M+Na]$^+$ 253.0060, found 253.0060.

Dibutyl (4-chlorobut-1-en-2-yl)phosphonate (**1f**)

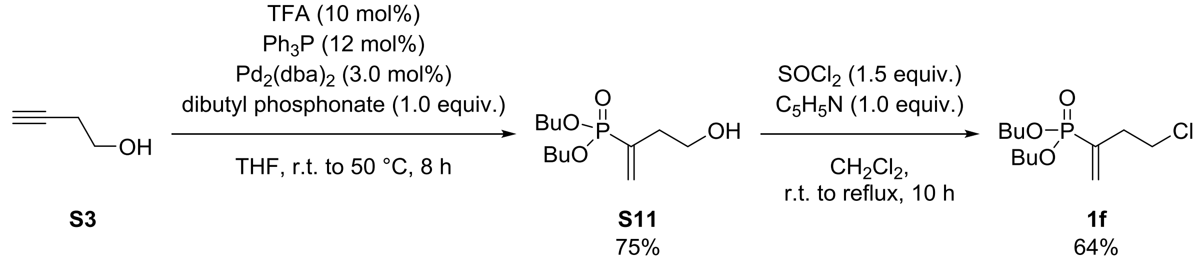

Dibutyl (4-hydroxybut-1-en-2-yl)phosphonate (**S11**) was prepared following a modified literature procedure:[9] Under argon, $Pd_2(dba)_3$ (137 mg, 0.150 mmol, 3.00 mol%) and Ph_3P (157 mg, 0.600 mmol, 12.0 mol%) were placed in a septum-sealed tube equipped with a magnetic stir bar. THF (2.5 mL) was added and the mixture was stirred for 3 min before the dibutyl phosphonate (971 mg, 5.00 mmol, 1.00 equiv.) and alkyne **S3** (0.35 g, 5.0 mmol, 1.0 equiv.) were added. TFA (57 mg, 0.50 mmol, 10 mol%) was added and the tube was capped with a PTFE-sealed screw cap. The mixture was stirred at 50 °C for 8 h, then H_2O (20 mL) was added carefully and the mixture was extracted into EtOAc (3 × 20 mL). The combined organic phases were dried ($MgSO_4$), filtered, and concentrated *in vacuo*. The residue was purified by flash column chromatography (80% EtOAc/hexane) to afford dibutyl (4-hydroxybut-1-en-2-yl)phosphonate (**S11**) (990 mg, 3.75 mmol, 75%) as a yellow oil.

Homoallyl chloride **1f** was then prepared following the same chlorination procedure as described in the synthesis of **1b**, with alcohol **S11** (990 mg, 3.75 mmol, 1.00 equiv.), pyridine (297 mg, 3.75 mmol, 1.00 equiv.), and thionyl chloride (670 mg, 5.63 mmol, 1.50 equiv.). The crude product was purified by flash column chromatography (40% EtOAc/hexane) to afford **1f** (679 mg, 2.40 mmol, 64%) as a yellow oil.

TLC: R_f = 0.50 (50% EtOAc/hexane, $KMnO_4$ stain).

^{1}H NMR (400 MHz, $CDCl_3$): δ_H 6.13 (d, *J* = 22.3 Hz, 1H), 5.86 (dt, *J* = 47.4, 1.3 Hz, 1H), 4.09 – 3.92 (m, 4H), 3.67 (t, *J* = 7.0 Hz, 2H), 2.69 (dt, *J* = 14.7, 7.0, 2H), 1.70 – 1.58 (m, 4H), 1.46 – 1.31 (m, 4H), 0.92 (t, *J* = 7.3 Hz, 6H) ppm.

^{13}C NMR (101 MHz, $CDCl_3$): δ_C 135.2 (d, *J* = 175.3 Hz), 132.2 (d, *J* = 9.0 Hz), 65.7 (d, *J* = 6.1 Hz), 42.3 (d, *J* = 4.6 Hz), 35.9 (d, *J* = 11.3 Hz), 32.4 (d, *J* = 6.5 Hz), 18.7, 13.6 ppm.

IR (film) ν_{max}: 2959, 2935, 2874, 1463, 1382, 1252, 1229, 1063, 1020, 975, 902 cm^{-1}.

HRMS (ESI^+) calcd. for $C_{12}H_{24}ClNaO_3P$ $[M+Na]^+$ 305.1044, found 305.1059.

4.2. Synthesis of Allyl Chlorides

Methyl (*E*)-4-chlorobut-2-enoate (8b)

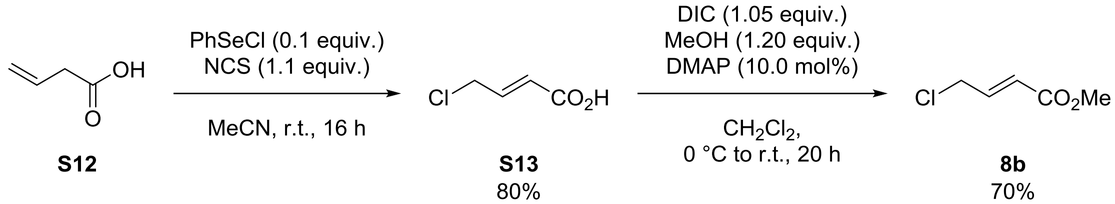

S12 **S13** 80% **8b** 70%

Allyl chloride **8b** was synthesised in two steps from but-3-enoic acid (**S12**) following a literature procedure.[10] All recorded spectroscopic data matched those previously reported in the literature.

^{1}H NMR (400 MHz, $CDCl_3$): δ_H 6.97 (dt, *J* = 15.4, 6.1 Hz, 1H), 6.10 (dt, *J* = 15.4, 1.6 Hz, 1H), 4.15 (dd, *J* = 6.1, 1.6 Hz, 2H), 3.75 (s, 3H) ppm.

^{13}C NMR (101 MHz, $CDCl_3$): δ_C 166.0, 141.9, 123.6, 51.8, 42.4 ppm.

***S*-Ethyl (*E*)-4-chlorobut-2-enethioate (8c)**

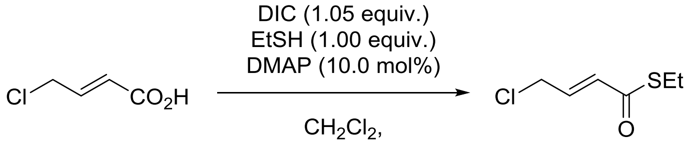

S13 **8c**

Thioester **8c** was prepared from carboxylic acid **S13** following a literature procedure.[10] All recorded spectroscopic data matched those previously reported in the literature.

^{1}H NMR (400 MHz, $CDCl_3$): δ_H 6.88 (dt, *J* = 15.2, 6.0 Hz, 1H), 6.35 (dt, *J* = 15.2, 1.5 Hz, 1H), 4.16 (dd, *J* = 6.0, 1.5 Hz, 2H), 2.97 (q, *J* = 7.4 Hz, 2H), 1.28 (t, *J* = 7.4 Hz, 3H) ppm.

^{13}C NMR (101 MHz, $CDCl_3$): δ_C 189.4, 137.2, 130.5, 42.5, 23.4, 14.6 ppm.

4.3. Synthesis of Styrene Derivatives

(4-Chlorobut-1-en-2-yl)benzene (1g)

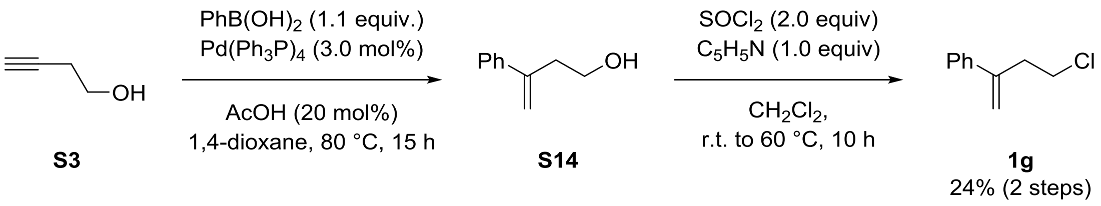

3-Phenylbut-3-en-1-ol (**S14**) was prepared following a modified literature procedure:[11] Under an argon atmosphere but-3-yn-1-ol (**S3**) (0.70 g, 10 mmol, 1.0 equiv.), $Pd(PPh_3)_4$ (347 mg, 0.300 mmol, 3.00 mol%) and phenylboronic acid (1.34 g, 11.0 mmol, 1.10 equiv.) were placed in a thick-walled glass vessel. Dry 1,4-dioxane (20 mL) and AcOH (120 mg, 2.00 mmol, 20.0 mol%) were added and the solution was stirred at r.t. for 15 min, then at 80 °C for 24 h. The reaction was cooled to r.t. and the 1,4-dioxane was removed *in vacuo*. The resulting crude product was purified by flash column chromatography (20% EtOAc/hexane) to afford 3-phenylbut-3-en-1-ol (**S14**) (1.03 g, 7.00 mmol, 70%) as a yellow oil.

Homoallyl chloride **1g** was then prepared following the same chlorination procedure as described in the synthesis of **1b**, with alcohol **S14** (1.03 g, 7.00 mmol, 1.00 equiv.), pyridine (0.55 g, 7.0 mmol, 1.0 equiv.), and thionyl chloride (1.66 g, 14.0 mmol, 2.00 equiv.). The crude product was purified by flash column chromatography (5% Et_2O/pentane) to afford **1g** (396 mg, 2.38 mmol, 34%) as a pale yellow oil.

TLC: R_f = 0.85 (pentane, $KMnO_4$ stain).

^{1}H NMR (400 MHz, $CDCl_3$): δ_H 7.42 – 7.25 (m, 5H), 5.40 (s, 1H), 5.18 (s, 1H), 3.59 (t, J = 7.3 Hz, 2H), 3.00 – 2.96 (m, 2H) ppm.

^{13}C NMR (101 MHz, $CDCl_3$): δ_C 144.6, 139.9, 128.5, 127.8, 126.1, 114.9, 42.8, 38.7 ppm.

IR (film) ν_{max}: 3026, 3087, 2960, 1628, 1574, 1494, 1443, 1242, 1028, 966, 901 cm^{-1}.

LRMS (EI^+): m/z 166 (M^+ [$C_{10}H_{11}Cl$], 12), 105 (100), 91 (24), 77 (52).

2-(4-Chlorobut-1-en-2-yl)naphthalene (1h)

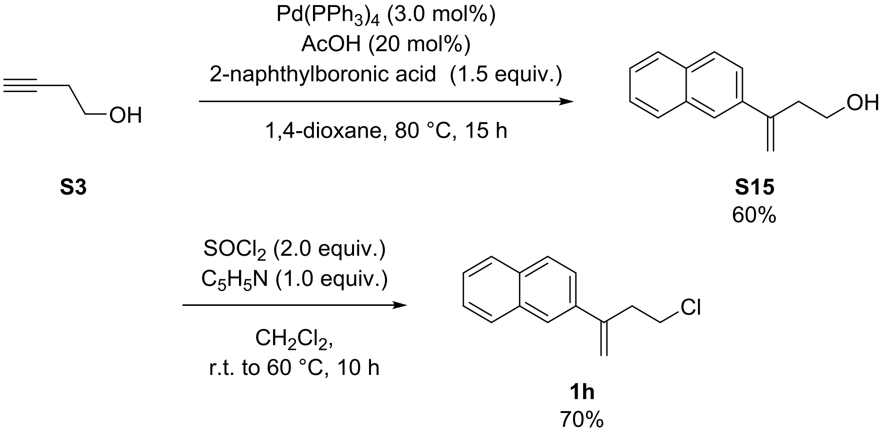

3-(Naphthalen-2-yl)but-3-en-1-ol (**S15**) was prepared following a literature procedure.[12]

Homoallyl chloride **1h** was then prepared following the same chlorination procedure as described in the synthesis of **1b**, with alcohol **S15** (0.99 g, 5.0 mmol, 1.0 equiv.), pyridine (0.40 g, 5.0 mmol, 1.0 equiv.), and thionyl chloride (1.19 g, 10.0 mmol, 2.00 equiv.). The crude product was purified by flash column chromatography (5% Et_2O/pentane) to afford **1h** (0.76 g, 3.5 mmol, 70%) as a pale yellow oil.

TLC: R_f = 0.60 (5% EtOAc/hexane, $KMnO_4$ stain).

^{1}H NMR (400 MHz, $CDCl_3$): δ_H 7.87 – 7.81 (m, 4H), 7.57 (dd, *J* = 8.6, 1.8 Hz, 1H), 7.55 – 7.43 (m, 2H), 5.56 (d, *J* = 1.0 Hz, 1H), 5.29 (td, *J* = 1.2, 1.0 Hz, 1H), 3.65 (t, *J* = 7.3 Hz, 2H), 3.11 (td, *J* = 7.4, 1.2 Hz, 2H) ppm.

^{13}C NMR (101 MHz, $CDCl_3$): δ_C 144.4, 137.1, 133.3, 132.9, 128.1, 127.5, 126.3, 126.0, 124.8, 124.4, 115.48, 115.46, 42.9, 38.7 ppm.

IR (film) ν_{max}: 3056, 2958, 2875, 1653, 1624, 1573, 1504, 1439, 1367, 1271, 1041, 892, 858 cm^{-1}.

LRMS (EI^+): m/z 216 (M^+ [$C_{14}H_{13}Cl$], 47), 165 (65), 127 (47), 71 (35).

4-(4-Chlorobut-1-en-2-yl)pyridine (**1i**)

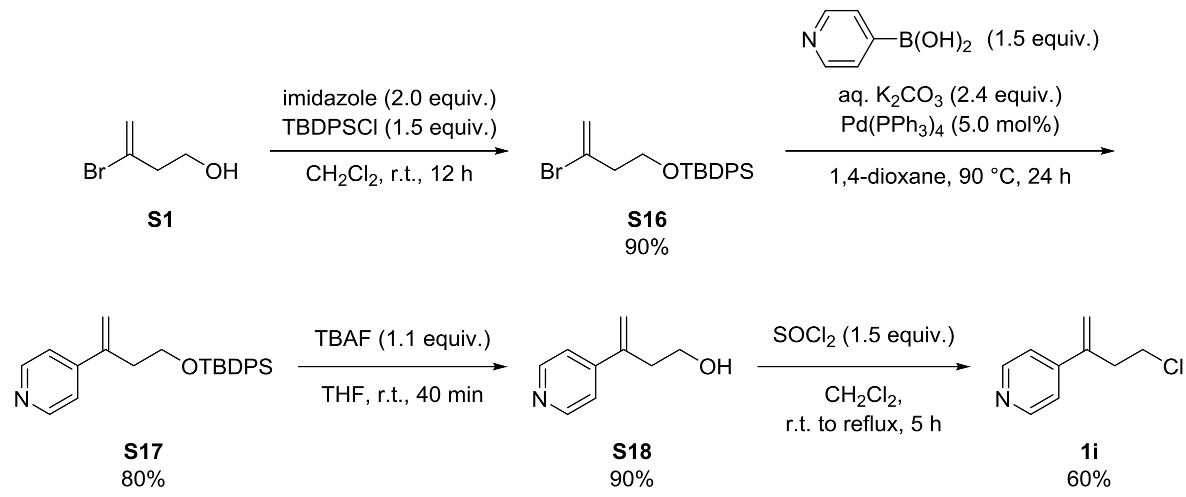

To a stirred solution of 3-bromobut-3-en-1-ol (**S1**) (1.51 g, 10.0 mmol, 1.00 equiv.) in CH_2Cl_2 (60 mL) under N_2 at 0 °C was added imidazole (1.4 g, 20 mmol, 2.0 equiv.) and TBDPSCl (4.1 g, 15 mmol, 1.5 equiv.) and the resulting solution was warmed to r.t. with stirring. After 10 h, the reaction mixture was diluted with Et_2O (30 mL) and the reaction was quenched by addition of H_2O (30 mL). The phases were separated and the aqueous phase was extracted into Et_2O (3 × 50 mL). The organic extracts were combined, washed with H_2O (30 mL) and brine (30 mL), then dried ($MgSO_4$), filtered, and concentrated *in vacuo*. The residue was purified by flash column chromatography (10% EtOAc/hexane) to give **S16** (3.50 g, 9.00 mmol, 90%) as a colourless oil.

4-(4-((*tert*-Butyldiphenylsilyl)oxy)but-1-en-2-yl)pyridine (**S17**) was prepared following a modified literature procedure:[13] An oven-dried Schlenk flask was charged with $Pd(PPh_3)_4$ (116 mg, 0.100 mmol, 5.00 mol%), ((3-bromobut-3-en-1-yl)oxy)(*tert*-butyl)diphenylsilane (**S16**) (1.75 g, 5.00 mmol, 1.00 equiv.), 3 M aqueous K_2CO_3 solution (4.0 mL, 12 mmol, 2.4 equiv.), pyridin-4-ylboronic acid (0.92 g, 7.5 mmol, 1.5 equiv.) and 1,4-dioxane (15 mL). The flask was evacuated and back-filled with argon three times before stirring at 90 °C for 24 h. The reaction was quenched with H_2O (10 mL) and extracted into EtOAc (3 × 20 mL). The organic phases were dried ($MgSO_4$), filtered, and concentrated *in vacuo*. Purification by flash column chromatography (50% EtOAc/hexane) afforded **S17** (1.55 g, 4.00 mmol, 80%) as a colourless oil.

To a solution of 4-(4-((*tert*-butyldiphenylsilyl)oxy)but-1-en-2-yl)pyridine (**S17**) (1.55 g, 4.00 mmol, 1.00 equiv.) in THF (20 mL) was added TBAF (1.0 M solution in THF, 4.4 mL, 4.4 mmol, 1.1 equiv.). After stirring at r.t. for 40 min, the reaction mixture was quenched with saturated aqueous ammonium chloride (20 mL), extracted into Et_2O (3 × 40 mL), washed with brine (40 mL), dried ($MgSO_4$), filtered, and concentrated *in vacuo*. Purification by flash column chromatography (100% EtOAc) afforded **S18** (0.53 g, 3.6 mmol, 90%) as a colourless oil.

To a stirred solution of 3-(pyridin-4-yl)but-3-en-1-ol (**S18**) (0.53 g, 3.6 mmol, 1.0 equiv.) in CH_2Cl_2 (20 mL) under N_2 at 0 °C was added $SOCl_2$ (0.64 g, 5.4 mmol, 1.5 equiv.) and the resulting solution was heated to reflux for 10 h. The reaction mixture was diluted with Et_2O (30 mL) and the reaction was quenched by slow addition of saturated aqueous $NaHCO_3$ (20 mL). The phases were separated, and the aqueous phase was extracted into Et_2O (3 × 30 mL). The organic extracts were combined, washed with H_2O (40 mL) and brine (40 mL), then dried ($MgSO_4$), filtered, and concentrated *in vacuo*. Purification by flash column chromatography (100% EtOAc) gave **1i** (362 mg, 2.16 mmol, 60%) as a colourless oil.

TLC: R_f = 0.20 (100% EtOAc, $KMnO_4$ stain).

^{1}H NMR (400 MHz, $CDCl_3$): δ_H 8.56 (d, *J* = 5.2 Hz, 2H), 7.25 (d, *J* = 8.0 Hz, 2H), 5.56 (s, 1H), 5.31 (s, 1H), 3.56 (t, *J* = 7.2 Hz, 2H), 2.93 (t, *J* = 7.2 Hz, 2H) ppm.

^{13}C NMR (101 MHz, $CDCl_3$): δ_C 150.1, 147.2, 142.3, 120.6, 117.9, 42.2, 37.5 ppm.

IR (film) ν_{max}: 2973, 2877, 1683, 1638, 1595, 1391, 1364, 1166, 1109, 993 cm^{-1}.

HRMS (ESI^+) calcd. for $C_9H_{10}ClNNa$ $[M+Na]^+$ 190.0394, found 190.0402.

2-(4-Chlorobut-1-en-2-yl)benzofuran (1j)

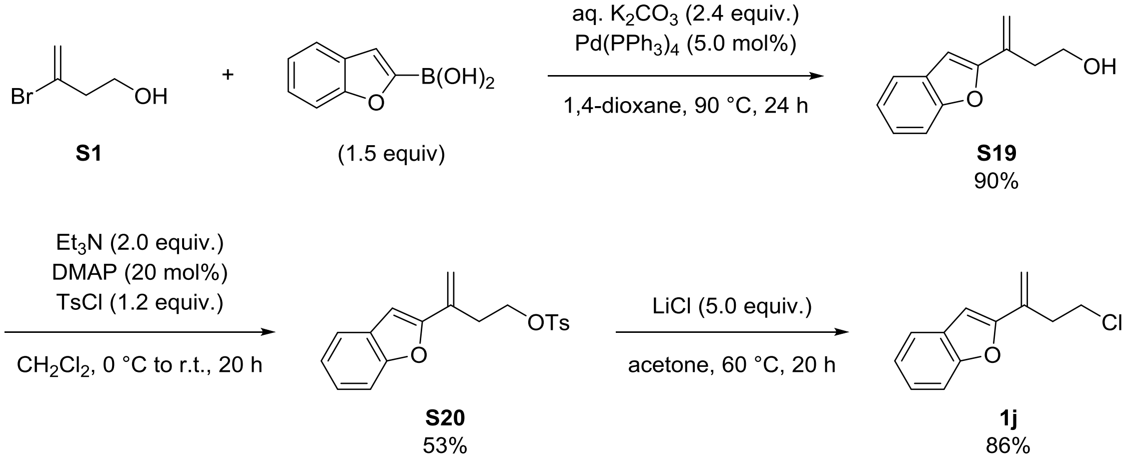

3-(Benzofuran-2-yl)but-3-en-1-ol (**S19**) was prepared following a modified literature procedure:[13] An oven-dried Schlenk flask was charged with $Pd(PPh_3)_4$ (116 mg, 0.100 mmol, 5.00 mol%), 3-bromobut-3-en-1-ol (**S1**) (755 mg, 5.00 mmol, 1.00 equiv.), 3 M aqueous K_2CO_3 solution (4.0 mL, 12 mmol, 2.4 equiv.), benzofuran-2-ylboronic acid (1.22 g, 7.50 mmol, 1.50 equiv.) and 1,4-dioxane (15 mL). The flask was evacuated and back filled with argon three times before heating to 90 °C and stirring for 24 h. The reaction was quenched with H_2O (10 mL) and extracted into EtOAc (3 × 20 mL). The organic phases were dried ($MgSO_4$), filtered, and concentrated *in vacuo*. Purification by flash column chromatography (30% EtOAc/hexane) gave **S19** (847 mg, 4.50 mmol, 90%) as a colourless oil.

Homoallyl chloride **1j** was prepared following a modified literature procedure:[14] Et_3N (1.0 g, 9.0 mmol, 2.0 equiv.) was added to 3-(benzofuran-2-yl)but-3-en-1-ol (**S19**) (847 mg, 4.50 mmol, 1.00 equiv.) in CH_2Cl_2 (30 mL) at r.t., followed by DMAP (110 mg, 0.900 mmol, 20.0 mol%) and *p*-toluenesulfonyl chloride (1.03 g, 5.40 mmol, 1.20 equiv.). The reaction was stirred for 10 h before quenching with saturated aqueous Na_2CO_3 (30 mL) and extracting into CH_2Cl_2 (3 × 30 mL). The organic phases were dried ($MgSO_4$), filtered, and concentrated *in vacuo*. Purification by flash column chromatography (20% EtOAc/hexane) afforded sulfonate **S20** (817 mg, 2.38 mmol, 53%) as a colourless oil.

To a solution of sulfonate **S20** (817 mg, 2.38 mmol, 1.00 equiv.) in acetone (10 mL) was added lithium chloride (505 mg, 12.0 mmol, 5.00 equiv.) and the reaction mixture was heated to reflux for 40 h. The reaction was allowed to cool to r.t., quenched with H_2O (30 mL), and extracted into CH_2Cl_2 (3 × 30 mL). The organic phases were dried ($MgSO_4$), filtered, and concentrated *in vacuo*. Purification by flash column chromatography (20% EtOAc/hexane) afforded **1j** (423 mg, 3.87 mmol, 86%) as a colourless oil.

TLC: R_f = 0.50 (10% EtOAc/hexane, $KMnO_4$ stain).

^{1}H NMR (400 MHz, $CDCl_3$): δ_H 7.56 (ddd, *J* = 7.6, 1.4, 0.7 Hz, 1H), 7.47 (ddd, *J* = 8.2, 0.9, 0.9 Hz, 1H), 7.30 (ddd, *J* = 8.1, 7.3, 1.4 Hz, 1H), 7.23 (td, *J* = 7.5, 1.1 Hz, 1H), 6.70 (s, 1H), 5.96 (d, *J* = 0.8 Hz, 1H), 5.31 (td, *J* = 1.2, 0.8 Hz,, 1H), 3.76 (t, *J* = 7.4 Hz, 2H), 2.95 (td, *J* = 7.4, 1.1 Hz, 2H) ppm.

^{13}C NMR (101 MHz, $CDCl_3$): δ_C 155.3, 154.7, 133.7, 128.7, 124.8, 122.9, 121.0, 115.1, 111.0, 102.8, 43.0, 36.5 ppm.

IR (film) ν_{max}: 3056, 2965, 2932, 1681, 1555, 1452, 1253, 1167, 929, 803 cm^{-1}.

HRMS (ESI^+) calcd. for $C_{12}H_{11}ClNaO$ $[M+Na]^+$ 229.0391, found 229.0391.

5. Product Characterisation

***tert*-Butyl 2-((1-(4,4,5,5-tetramethyl-1,3,2-dioxaborolan-2-yl)cyclopropyl)methyl)pyrrolidine-1-carboxylate** (**4a**)

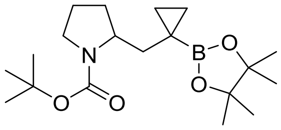

Prepared following General Procedure A using Boc-Pro-OH (65 mg, 0.30 mmol, 1.0 equiv.), 4CzIPN (2.4 mg, 0.0030 mmol, 1.0 mol%), Cs_2CO_3 (195 mg, 0.600 mmol, 2.00 equiv.), alkenyl boronic ester **1a** (96 µL, 0.45 mmol, 1.5 equiv.) and DMF (6.0 mL), which was irradiated with 1 × Kessil lamp for 20 h. Purification by flash column chromatography (10% EtOAc/pentane) gave the title compound (104 mg, 0.296 mmol, 99%) as a colourless oil.

TLC: R_f = 0.45 (10% EtOAc/pentane, $KMnO_4$ stain).

^{1}H NMR (400 MHz, $CDCl_3$): δ_H 3.95 – 3.86 (br. m, 1H), 3.35 – 3.29 (br. m, 2H), 2.03 – 1.73 (m, 4H), 1.63 – 1.57 (m, 1H), 1.45 (s, 9H), 1.29 – 1.25 (m, 1H), 1.20 (s, 6H), 1.19 (s, 6H), 0.75 – 0.57 (br. m, 2H), 0.51 – 0.31 (br. m, 2H) ppm.

^{13}C NMR (101 MHz, $CDCl_3$): δ_C 154.6, 83.0, 78.8 + 78.5 (rotameric peaks), 57.7 + 57.3 (rotameric peaks), 46.2 + 45.8 (rotameric peaks), 39.8 + 39.4 (rotameric peaks), 29.8, 28.7, 24.8, 24.6, 23.7 + 23.0 (rotameric peaks), 13.5 + 13.2 (rotameric peaks), 10.1, 1.8 (br.) ppm.

^{11}B NMR (128 MHz, $CDCl_3$): δ_B 33.3 (br. s, 1B) ppm.

IR (film) ν_{max}: 2975, 1691, 1389, 1139, 855 cm^{-1}.

HRMS (ESI^+) calcd. for $C_{19}H_{35}NBO_4$ $[M+H]^+$ 352.2657, found 352.2659.

***tert*-Butyl 2-((1-(methoxycarbonyl)cyclopropyl)methyl)pyrrolidine-1-carboxylate** (**4b**)

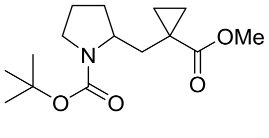

Prepared following General Procedure B using Boc-Pro-OH (65 mg, 0.30 mmol, 1.0 equiv.), 4CzIPN (2.4 mg, 0.0030 mmol, 1.0 mol%), Cs_2CO_3 (195 mg, 0.600 mmol, 2.00 equiv.), methyl 4-chloro-2-methylenebutanoate (**1b**) (89 mg, 0.60 mmol, 2.0 equiv.) and CH_2Cl_2 (6.0 mL), which was irradiated

with 1 × Kessil lamp for 15 h. Purification by flash column chromatography (20% EtOAc/pentane) gave the title compound (77 mg, 0.27 mmol, 91%) as a colourless oil.

TLC: R_f = 0.33 (20% EtOAc/pentane, $KMnO_4$ stain).

^{1}H NMR (500 MHz, DMSO-d_6, 100 ºC): δ_H 4.02 – 3.98 (m, 1H), 3.59 (s, 3H), 3.31 – 3.26 (m, 1H), 3.19 – 3.12 (m, 1H), 1.85 – 1.65 (m, 6H), 1.39 (s, 9H), 1.13 – 1.04 (m, 2H), 0.85 – 0.70 (m, 2H) ppm.

^{13}C NMR (126 MHz, DMSO-d_6, 100 °C): δ_C 174.8, 154.1, 78.5, 56.2, 51.7, 45.9, 37.7, 30.6, 28.7, 23.1, 21.6, 16.1, 14.3 ppm.

IR (film) ν_{max}: 3045, 2976, 1717, 1681, 1437, 1398, 1295, 1167, 1053, 1005, 820 cm^{-1}.

HRMS (ESI$^+$): calcd. for $C_{15}H_{25}NNaO_4$ [M+Na]$^+$ 306.1676, found 306.1692.

***tert*-Butyl 2-((1-cyanocyclopropyl)methyl)pyrrolidine-1-carboxylate** (**4c**)

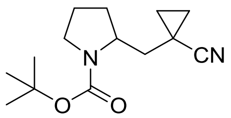

Prepared following General Procedure B using Boc-Pro-OH (65 mg, 0.30 mmol, 1.0 equiv.), 4CzIPN (2.4 mg, 0.0030 mmol, 1.0 mol%), Cs_2CO_3 (195 mg, 0.600 mmol, 2.00 equiv.), 4-chloro-2-methylenebutanenitrile (**1c**) (70 mg, 0.60 mmol, 2.0 equiv.) and CH_2Cl_2 (6.0 mL), which was irradiated with 1 × Kessil lamp for 15 h. Purification by flash column chromatography (30% EtOAc/pentane) gave the title compound (68 mg, 0.27 mmol, 90%) as a colourless oil.

TLC: R_f = 0.25 (30% EtOAc/pentane, $KMnO_4$ stain).

^{1}H NMR (500 MHz, DMSO-d_6, 100 ºC): δ_H 3.95 – 3.89 (m, 1H), 3.36 – 3.27 (m, 1H), 3.24 – 3.17 (m, 1H), 2.07 – 1.96 (m, 1H), 1.90 – 1.75 (m, 4H), 1.56 – 1.48 (m, 1H), 1.42 (s, 9H), 1.22 – 1.14 (m, 2H), 0.98 – 0.86 (m, 2H) ppm.

^{13}C NMR (126 MHz, DMSO-d_6, 100 °C): δ_C 153.5, 123.3, 78.5, 55.9, 45.8, 38.2, 29.9, 28.3, 22.9, 14.4, 12.9, 6.8 ppm.

IR (film) ν_{max}: 3044, 2977, 1684, 1396, 1283, 1224, 1025, 820 cm^{-1}.

HRMS (ESI$^+$): calcd. for $C_{14}H_{22}N_2NaO_2$ [M+Na]$^+$ 273.1573, found 273.1581.

***tert*-Butyl 2-((1-carbamoylcyclopropyl)methyl)pyrrolidine-1-carboxylate** (**4d**)

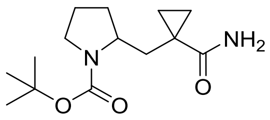

Prepared following General Procedure B using Boc-Pro-OH (65 mg, 0.30 mmol, 1.0 equiv.), 4CzIPN (2.4 mg, 0.0030 mmol, 1.0 mol%), Cs_2CO_3 (195 mg, 0.600 mmol, 2.00 equiv.), 4-chloro-2-methylenebutanamide (**1d**) (80 mg, 0.60 mmol, 2.0 equiv.) and CH_2Cl_2 (6.0 mL), which was irradiated with 1 × Kessil lamp for 15 h. Purification by flash column chromatography (90% EtOAc/pentane) gave the title compound (59 mg, 0.22 mmol, 73%) as a colourless oil.

TLC: R_f = 0.30 (EtOAc, $KMnO_4$ stain).

^{1}H NMR (500 MHz, DMSO-d_6, 100 °C): δ_H 6.83 (br. s, 2H), 3.94 – 3.84 (m, 1H), 3.31 – 3.22 (m, 1H), 3.24 – 3.15 (m, 1H), 2.21 – 1.97 (br. m, 1H), 1.96 – 1.87 (m, 1H), 1.86 – 1.79 (m, 1H), 1.79 – 1.70 (m, 2H), 1.54 – 1.34 (m, 10H), 1.03 – 0.97 (m, 1H), 0.95 – 0.90 (m, 1H), 0.65 – 0.60 (m, 1H), 0.57 – 0.51 (m, 1H) ppm.

^{13}C NMR (126 MHz, DMSO-d_6, 100 °C): δ_C 175.2, 154.0, 78.6, 56.1, 45.8, 38.0, 30.5, 28.3, 22.9, 22.0, 13.5, 13.1 ppm.

IR (film) ν_{max}: 3407, 2978, 1668, 1406, 1169, 819 cm^{-1}.

HRMS (ESI^+): calcd. for $C_{14}H_{24}N_2NaO_3$ $[M+Na]^+$ 291.1697, found 291.1682.

***tert*-Butyl 2-((1-(phenylsulfonyl)cyclopropyl)methyl)pyrrolidine-1-carboxylate** (**4e**)

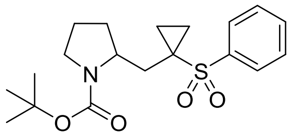

Prepared following General Procedure B using Boc-Pro-OH (65 mg, 0.30 mmol, 1.0 equiv.), 4CzIPN (2.4 mg, 0.0030 mmol, 1.0 mol%), Cs_2CO_3 (195 mg, 0.600 mmol, 2.00 equiv.), ((4-chlorobut-1-en-2-yl)sulfonyl)benzene (**1e**) (138 mg, 0.600 mmol, 2.00 equiv.) and CH_2Cl_2 (6.0 mL), which was irradiated with 1 × Kessil lamp for 15 h. Purification by flash column chromatography (50% EtOAc/pentane) gave the title compound (84 mg, 0.23 mmol, 77%) as a colourless oil.

TLC: R_f = 0.33 (50% EtOAc/pentane, $KMnO_4$ stain).

^{1}H NMR (500 MHz, DMSO-d_6, 100 °C): δ_H 7.94 – 7.88 (m, 2H), 7.78 – 7.66 (m, 1H), 7.68 – 7.61 (m, 2H), 3.78 – 3.70 (br. m, 1H), 3.25 – 3.10 (m, 2H), 2.16 (ddd, J = 14.6, 3.1, 2.7 Hz, 1H), 1.82 – 1.73 (m,

1H), 1.72 – 1.62 (m, 2H), 1.63 – 1.50 (m, 3H), 1.45 – 1.37 (m, 1H), 1.35 (s, 9H), 1.10 – 0.99 (m, 2H) ppm.

^{13}C NMR (126 MHz, DMSO-d_6, 100 °C): δ_C 153.3, 138.8, 134.1 + 133.5 (rotameric peaks), 129.5 + 129.2 (rotameric peaks), 128.6 + 128.4 (rotameric peaks), 78.7 + 78.4 (rotameric peaks), 54.8 + 54.5 (rotameric peaks), 46.0 + 45.8 (rotameric peaks), 41.9 + 39.3 (rotameric peaks), 33.9, 32.0 + 30.1 (rotameric peaks), 29.5 + 28.2 (rotameric peaks), 22.5, 11.4, 10.7 ppm.

IR (film) ν_{max}: 3050, 2982, 1688, 1446, 1394, 1373, 1240, 1043, 1029 cm^{-1}.

HRMS (ESI$^+$): calcd. for $C_{19}H_{27}NNaO_4S$ [M+Na]$^+$ 388.1553, found 388.1568.

***tert*-Butyl 2-((1-(dibutoxyphosphoryl)cyclopropyl)methyl)pyrrolidine-1-carboxylate (4f)**

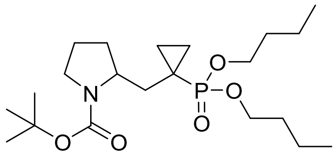

Prepared following General Procedure B using Boc-Pro-OH (65 mg, 0.30 mmol, 1.0 equiv.), 4CzIPN (2.4 mg, 0.0030 mmol, 1.0 mol%), Cs_2CO_3 (195 mg, 0.600 mmol, 2.00 equiv.), dibutyl (4-chlorobut-1-en-2-yl)phosphonate (**1f**) (170 mg, 0.600 mmol, 2.00 equiv.) and CH_2Cl_2 (6.0 mL), which was irradiated with 1 × Kessil lamp for 15 h. Purification by flash column chromatography (50% EtOAc/pentane) gave the title compound (104 mg, 0.249 mmol, 83%) as a colourless oil.

TLC: R_f = 0.20 (100% EtOAc, $KMnO_4$ stain).

^{1}H NMR (500 MHz, DMSO-d_6, 100 °C): δ_H 4.00 – 3.93 (m, 4H), 3.91 – 3.85 (m, 1H), 3.28 – 3.16 (m, 2H), 2.06 (ddd, J = 14.2, 11.6, 3.1 Hz, 1H), 1.94 – 1.85 (m, 2H), 1.82 – 1.72 (m, 2H), 1.66 – 1.58 (m, 4H), 1.47 – 1.23 (m, 14H), 1.03 – 0.86 (m, 8H), 0.73 – 0.65 (m, 2H) ppm.

^{13}C NMR (126 MHz, DMSO-d_6, 100 °C): δ_C 153.4, 78.1, 64.9 (dd, J = 10.3, 6.5 Hz), 55.6 (d, J = 2.8 Hz), 45.8, 37.1 (d, J = 4.9 Hz), 32.2 (dd, J = 5.7, 2.6 Hz), 29.9, 28.3, 22.6, 18.2 (d, J = 2.5 Hz), 13.2 (d, J = 1.6 Hz), 12.4 (d, J = 188.5 Hz), 10.8 (d, J = 2.0 Hz), 8.9 (d, J = 2.6 Hz) ppm.

IR (film) ν_{max}: 2961, 2934, 1690, 1456, 1391, 1230, 1169, 1020, 976, 901, 861 cm^{-1}.

HRMS (ESI$^+$): calcd. for $C_{21}H_{40}NNaO_5P$ [M+Na]$^+$ 440.2536, found 440.2536.

***tert*-Butyl 2-(2-(4,4,5,5-tetramethyl-1,3,2-dioxaborolan-2-yl)cyclopropyl)pyrrolidine-1-carboxylate** (**7a**)

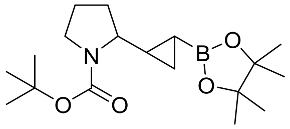

Prepared following a modified General Procedure A with Boc-Pro-OH (65 mg, 0.30 mmol, 1.0 equiv.), 4CzIPN (12 mg, 0.015 mmol, 5.0 mol%), K_2CO_3 (83 mg, 0.60 mmol, 2.0 equiv.), alkenyl boronic ester **8a** (118 µL, 0.600 mmol, 2.00 equiv.) and DMF (6.0 mL), which was irradiated with 1 × Kessil lamp for 16 h. Purification by flash column chromatography (15% EtOAc/pentane) gave the title compound (47 mg, 0.14 mmol, 47%) as a colourless oil. The d.r. was determined to be 56:39:5 by ^{1}H NMR.

TLC: R_f = 0.43 (15% EtOAc/pentane, $KMnO_4$ stain).

^{1}H NMR (400 MHz, $CDCl_3$): δ_H (56:39:5 ratio of diastereomers, only the major two diastereomers A and B are assigned) 3.51 – 3.14 (m, 3H), 2.01 – 1.67 (m, 4H), 1.49 – 1.44 (m, 9H), 1.23 – 1.15 (m, 12H + diastereomer B, m, 0.39H), 1.14 – 1.08 (diastereomer A, m, 0.56H), 1.08 – 1.01 (diastereomer B, m, 0.39H), 0.74 (diastereomer B, ddd, *J* = 7.8, 6.3, 3.6 Hz, 0.39H), 0.56 (diastereomer A, ddd, *J* = 8.1, 6.4, 3.7 Hz, 0.56H), 0.52 – 0.32 (diastereomers A, br. m, 0.56H), 0.15 – –0.01 (diastereomer A, m, 0.56H), –0.07 – –0.18 (diastereomer C, m, 0.05H), –0.23 – –0.39 (diastereomer B, m, 0.39H) ppm.

^{13}C NMR (101 MHZ, $CDCl_3$): δ_C (56:39:5 ratio of diastereomers, the minor diastereomer was not observed), 155.2 + 155.8 (diastereomeric peaks), 83.00 + 82.96 (diastereomeric peaks), 79.3 (br.), 61.4, 46.7, 32.1 (br.), 28.7 + 28.6 (diastereomeric peaks), 25.0 + 24.7 (diastereomeric peaks), 24.8 + 24.6 (diastereomeric peaks), 24.9 (minor), 23.3 +23.0 (diastereomeric peaks), 22.6 (major), 11.5 (br. minor), 7.7 (major) ppm. The carbon directly attached to boron was not detected due to the boron quadrupole.

^{11}B NMR (128 MHz, $CDCl_3$): δ_B 32.9 (br. s, 1B) ppm.

IR (film) ν_{max}: 2976, 1693, 1390 – 1367, 1167 – 1145 cm^{-1}.

HRMS (ESI$^+$) calcd. for $C_{18}H_{33}BNO_4$ [M+H]$^+$ 338.2500, found 338.2504.

***tert*-Butyl 2-(2-(methoxycarbonyl)cyclopropyl)pyrrolidine-1-carboxylate** (**7b**)

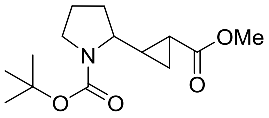

Prepared following General Procedure B using Boc-Pro-OH (65 mg, 0.30 mmol, 1.0 equiv.), 4CzIPN (2.4 mg, 0.0030 mmol, 1.0 mol%), Cs_2CO_3 (195 mg, 0.600 mmol, 2.00 equiv.), methyl (*E*)-4-chlorobut-2-enoate (**8b**) (81 mg, 0.60 mmol, 2.0 equiv.) and CH_2Cl_2 (6.0 mL), which was irradiated with 1 × Kessil lamp for 15 h. Purification by flash column chromatography (20% EtOAc/pentane) gave the title compound (71 mg, 0.26 mmol, 88%) as a colourless oil. The d.r. was determined to be 62:38 by high temperature NMR in DMSO-d_6.

TLC: R_f = 0.33 (20% EtOAc/pentane, $KMnO_4$ stain).

^{1}H NMR (500 MHz, DMSO-d_6, 100 ºC): 62:38 mixture of diastereomers: δ_H 3.60 (s, 3H) 3.60 – 3.56 (m, 0.38H), 3.46 – 3.40 (m, 0.62H), 3.35 – 3.20 (m, 2H), 1.98 – 1.63 (m, 4.62H), 1.59 – 1.53 (m, 0.38H), 1.49 – 1.40 (m, 10H), 1.08 – 1.04 (m, 0.76H), 0.99 – 0.93 (m, 0.62H), 0.86 – 0.80 (m, 0.62H) ppm.

^{13}C NMR (126 MHz, DMSO-d_6, 100 °C): δ_C Mixture of diastereomers: 173.5 (minor), 173.4 (major), 154.1 (minor), 154.0 (major), 78.6 (major), 78.5 (minor), 58.6 (minor), 58.0 (major), 51.24 (minor), 51.18 (major), 46.5 (minor), 46.4 (major), 30.8, 28.22 (minor), 28.16 (major), 23.0 (minor), 22.9 (major), 19.3 (undetermined), 16.9 (undetermined), 13.6 (undetermined), 11.4 (undetermined) ppm.

IR (film) ν_{max}: 3046, 2976, 2881, 1724, 1686, 1391, 1365, 1272, 1170, 1055, 1028, 819, cm^{-1}.

HRMS (ESI^+): calcd. for $C_{14}H_{23}NNaO_4$ $[M+Na]^+$ 292.1519, found 292.1542.

***tert*-Butyl 2-(2-((ethylthio)carbonyl)cyclopropyl)pyrrolidine-1-carboxylate** (**7c**)

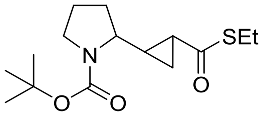

Prepared following General Procedure B using Boc-Pro-OH (65 mg, 0.30 mmol, 1.0 equiv.), 4CzIPN (2.4 mg, 0.0030 mmol, 1.0 mol%), Cs_2CO_3 (195 mg, 0.600 mmol, 2.00 equiv.), *S*-ethyl (*E*)-4-chlorobut-2-enethioate (**8c**) (99 mg, 0.60 mmol, 2.0 equiv.) and CH_2Cl_2 (6.0 mL), which was irradiated with 1 × Kessil lamp for 15 h. Purification by flash column chromatography (20% EtOAc/pentane) gave the title compound (59 mg, 0.20 mmol, 66%) as a colourless oil. The d.r. was determined to be 63:37 by high temperature NMR in DMSO-d_6.

TLC: R_f = 0.33 (10% EtOAc/hexane, $KMnO_4$ stain).

^{1}H NMR (500 MHz, DMSO-d_6, 100 ºC): 63:37 ratio of diastereomers: δ_H 3.67 – 3.59 (m, 0.37H), 3.51 – 3.44 (m, 0.63H), 3.36 – 3.20 (m, 2H), 2.90 – 2.81 (m, 2H), 2.32 – 2.24 (m, 0.63H), 2.04 – 1.96 (m, 0.37H), 1.99 – 1.75 (m, 3H), 1.75 – 1.62 (m, 1H), 1.61 – 1.53 (m, 1H), 1.45 (s, 5.67H), 1.43 (s, 3.33H), 1.27 – 1.09 (m, 4.37H), 0.99 – 0.91 (m, 0.63H) ppm.

^{13}C NMR (126 MHz, DMSO-d_6, 100 ºC): δ_C Mixture of diastereomers: 197.2 (minor), 197.1 (major), 154.01 (minor), 153.97 (major), 78.6 (major), 78.5 (minor), 58.6 (major), 57.9 (minor), 46.5 (minor), 46.3 (major), 30.8 (minor), 30.7 (major), 28.9 (minor), 28.8 (major), 28.6 (minor), 28.2 (major), 26.8, 22.95 (minor), 22.92 (major), 22.7 (minor), 22.6 (major), 15.5 (minor), 14.6 (major), 14.5 (minor), 13.6 (major) ppm.

IR (film) ν_{max}: 2972, 2931, 1683, 1389, 1365, 1167, 1105, 926, 822 cm^{-1}.

HRMS (ESI$^+$): calcd. for $C_{15}H_{25}NNaO_3S$ [M+Na]$^+$ 322.1447, found 322.1466.

***tert*-Butyl 2-((1-phenylcyclopropyl)methyl)pyrrolidine-1-carboxylate (4g)**

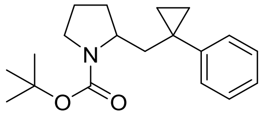

Prepared following General Procedure A using Boc-Pro-OH (65 mg, 0.30 mmol, 1.0 equiv.), 4CzIPN (2.4 mg, 0.0030 mmol, 1.0 mol%), Cs_2CO_3 (195 mg, 0.600 mmol, 2.00 equiv.), (4-chlorobut-1-en-2-yl)benzene (**1g**) (50 mg, 0.60 mmol, 2.0 equiv.) and DMF (6.0 mL), which was irradiated with 1 × Kessil lamp for 15 h. Purification by flash column chromatography (5% EtOAc/pentane) gave the title compound (77 mg, 0.26 mmol, 85%) as a colourless oil.

TLC: R_f = 0.33 (5% EtOAc/pentane, $KMnO_4$ stain).

^{1}H NMR (500 MHz, DMSO-d_6, 100 ºC): δ_H 7.36 – 7.29 (m, 2H), 7.30 – 7.22 (m, 2H), 7.18 – 7.13 (m, 1H), 3.65 – 3.58 (m, 1H), 3.24 – 3.17 (m, 1H), 3.15 – 3.08 (m, 1H), 2.28 – 2.22 (m, 1H), 1.76 – 1.58 (m, 4H), 1.44 – 1.39 (m, 1H), 1.37 – 1.36 (m, 9H), 0.90 – 0.84 (m, 1H), 0.80 – 0.71 (m, 2H), 0.71 – 0.65 (m, 1H) ppm.

^{13}C NMR (126 MHz, DMSO-d_6, 100 °C): δ_C 153.8, 145.1, 128.8, 128.5, 126.2, 78.5, 56.4, 46.2, 43.3, 30.1, 28.7, 23.7, 23.1, 14.0, 12.6 ppm.

IR (film) ν_{max}: 3046, 2977, 2877, 1682, 1446, 1396, 1365, 1277, 1170, 1027, 820 cm^{-1}.

HRMS (ESI$^+$): calcd. for $C_{19}H_{27}NNaO_2$ [M+Na]$^+$ 324.1934, found 324.1962.

***tert*-Butyl 2-((1-(naphthalen-2-yl)cyclopropyl)methyl)pyrrolidine-1-carboxylate (4h)**

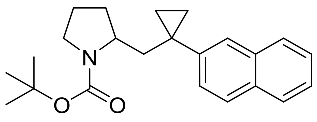

Prepared following General Procedure A using Boc-Pro-OH (65 mg, 0.30 mmol, 1.0 equiv.), 4CzIPN (2.4 mg, 0.0030 mmol, 1.0 mol%), Cs_2CO_3 (195 mg, 0.600 mmol, 2.00 equiv.), 2-(4-chlorobut-1-en-2-yl)naphthalene (**1h**) (130 mg, 0.600 mmol, 2.00 equiv.) and DMF (6.0 mL), which was irradiated with 1 × Kessil lamp for 15 h. Purification by flash column chromatography (20% EtOAc/pentane) gave the title compound (69 mg, 0.20 mmol, 65%) as a colourless oil.

TLC: R_f = 0.50 (10% EtOAc, $KMnO_4$ stain).

^{1}H NMR (500 MHz, DMSO-*d*$_6$, 100 °C): δ_H 7.86 – 7.76 (m, 4H), 7.56 – 7.50 (m, 1H), 7.49 – 7.39 (m, 2H), 3.70 – 3.62 (m, 1H), 3.25 – 3.16 (m, 1H), 3.15 – 3.08 (m, 1H), 2.36 (dd, *J* = 14.0, 2.6 Hz, 1H), 1.76 – 1.58 (m, 4H), 1.56 – 1.46 (m, 1H), 1.36 (s, 9H), 1.03 – 0.98 (m, 1H), 0.89 – 0.78 (m, 3H) ppm.

^{13}C NMR (126 MHz, DMSO-*d*$_6$, 100 °C): δ_C 153.4, 142.2, 133.2, 131.8, 127.5, 127.4, 127.3, 127.2, 126.3, 125.8, 125.2, 78.0, 56.1, 45.7, 42.8, 29.7, 28.3, 23.5, 22.7, 13.8, 12.3 ppm.

IR (film) ν_{max}: 2972, 2929, 1686, 1453, 1390, 1364, 1168, 1100, 818 cm^{-1}.

HRMS (ESI$^+$): calcd. for $C_{23}H_{29}NNaO_2$ $[M+Na]^+$ 374.2091, found 374.2096.

***tert*-Butyl 2-((1-(pyridin-4-yl)cyclopropyl)methyl)pyrrolidine-1-carboxylate (4i)**

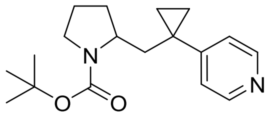

Prepared following General Procedure A using Boc-Pro-OH (65 mg, 0.30 mmol, 1.0 equiv.), 4CzIPN (2.4 mg, 0.0030 mmol, 1.0 mol%), Cs_2CO_3 (195 mg, 0.600 mmol, 2.00 equiv.), 4-(4-chlorobut-1-en-2-yl)pyridine (**1i**) (101 mg, 0.600 mmol, 2.00 equiv.) and DMF (6.0 mL), which was irradiated with 1 × Kessil lamp for 15 h. Purification by flash column chromatography (EtOAc) gave the title compound (64 mg, 0.21 mmol, 70%) as a colourless oil.

TLC: R_f = 0.20 (EtOAc, $KMnO_4$ stain).

^{1}H NMR (500 MHz, DMSO-*d*$_6$, 100 °C): δ_H 8.43 (d, *J* = 4.7 Hz, 2H), 7.29 (d, *J* = 4.7 Hz, 2H), 3.73 – 3.65 (m, 1H), 3.28 – 3.19 (m, 1H), 3.16 – 3.08 (m, 1H), 2.32 (dd, *J* = 14.1, 3.8 Hz, 1H), 1.82 – 1.61 (m,

4H), 1.53 – 1.44 (m, 1H), 1.38 (s, 9H), 1.04 – 0.96 (m, 1H), 0.93 – 0.86 (m, 1H), 0.88 – 0.80 (m, 1H), 0.81 – 0.72 (m, 1H) ppm.

^{13}C NMR (126 MHz, DMSO-*d*$_6$, 100 °C): δ_C 153.7, 153.4, 149.4, 122.7, 78.2, 55.7, 45.7, 41.0, 29.7, 28.3, 22.8, 22.0, 14.4, 13.8 ppm.

IR (film) ν_{max}: 2971, 1681, 1398, 1366, 1217, 1169, 820 cm^{-1}.

HRMS (ESI$^+$): calcd. for $C_{18}H_{26}N_2NaO_2$ [M+Na]$^+$ 325.1886, found 325.1889.

***tert*-Butyl 2-((1-(benzofuran-2-yl)cyclopropyl)methyl)pyrrolidine-1-carboxylate (4j)**

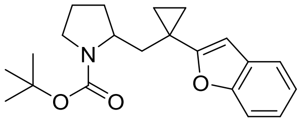

Prepared following General Procedure A using Boc-Pro-OH (65 mg, 0.30 mmol, 1.0 equiv.), 4CzIPN (2.4 mg, 0.0030 mmol, 1.0 mol%), Cs_2CO_3 (195 mg, 0.600 mmol, 2.00 equiv.), 2-(4-chlorobut-1-en-2-yl)benzofuran (**1j**) (124 mg, 0.600 mmol, 2.00 equiv.) and DMF (6.0 mL), which was irradiated with 1 × Kessil lamp for 15 h. Purification by flash column chromatography (20% EtOAc/pentane) gave the title compound (70 mg, 0.21 mmol, 68%) as a colourless oil.

TLC: R_f = 0.50 (10% EtOAc, $KMnO_4$ stain).

^{1}H NMR (500 MHz, DMSO-*d*$_6$, 100 °C): δ_H 7.51 – 7.47 (m, 1H), 7.43 – 7.39 (m, 1H), 7.22 – 7.14 (m, 2H), 6.62 (s, 1H), 3.93 – 3.85 (m, 1H), 3.30 – 3.22 (m, 1H), 3.20 – 3.14 (m, 1H), 2.27 (dd, *J* = 14.0, 3.4 Hz, 1H), 1.84 – 1.75 (m, 2H), 1.73 – 1.60 (m, 3H), 1.39 (s, 9H), 1.13 – 1.08 (m, 1H), 1.05 – 0.99 (m, 1H), 0.97 – 0.92 (m, 1H), 0.91 – 0.85 (m, 1H) ppm.

^{13}C NMR (126 MHz, DMSO-*d*$_6$, 100 °C): δ_C 161.7, 153.9, 153.5, 128.7, 123.1, 122.4, 120.2, 110.4, 101.3, 78.2, 55.9, 45.7, 39.4, 29.8, 28.3, 22.8, 17.4, 14.4, 12.8 ppm.

IR (film) ν_{max}: 2973, 2970, 1682, 1456, 1365, 1170, 1053, 820 cm^{-1}.

HRMS (ESI$^+$): calcd. for $C_{21}H_{27}NNaO_3$ [M+Na]$^+$ 364.1883, found 364.1914.

Benzyl 2-((1-(4,4,5,5-tetramethyl-1,3,2-dioxaborolan-2-yl)cyclopropyl)methyl)pyrrolidine-1-carboxylate (9a)

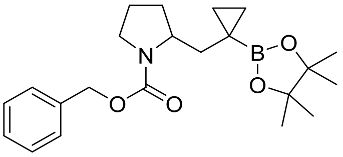

Prepared following General Procedure A using Z-Pro-OH (75 mg, 0.30 mmol, 1.0 equiv.), 4CzIPN (2.4 mg, 0.0030 mmol, 1.0 mol%), Cs_2CO_3 (195 mg, 0.600 mmol, 2.00 equiv.), alkenyl boronic ester **1a** (96 μL, 0.45 mmol, 1.5 equiv.) and DMF (6.0 mL), which was irradiated with 1 × Kessil lamp for 20 h. Purification by flash column chromatography (10% EtOAc/pentane) gave the title compound (94 mg, 0.25 mmol, 82%) as a colourless oil.

TLC: R_f = 0.50 (10% EtOAc/pentane, $KMnO_4$ stain).

^{1}H NMR (400 MHz, $CDCl_3$): δ_H 7.68 – 7.19 (m, 5H), 5.12 – 5.00 (m, 2H), 3.98 – 3.93 (br. m, 1H), 3.37 – 3.29 (br. m, 2H), 1.81 (br. m, 4H), 1.54 – 1.27 (m, 2H), 1.12 (br. s, 12H), 0.64 – 0.54 (br. m, 2H), 0.43 – 0.17 (br. m, 2H) ppm.

^{13}C NMR (101 MHz, $CDCl_3$): δ_C 155.1 + 154.8 (rotameric peaks), 137.4, 128.5, 127.9, 127.8, 83.1, 66.6 + 66.3 (rotameric peaks), 58.3 + 57.8 (rotameric peaks), 46.4 + 46.1 (rotameric peaks), 40.2 + 39.4 (rotameric peaks), 30.3 + 29.7 (rotameric peaks), 24.9, 24.6, 23.8 + 23.0 (rotameric peaks), 13.3 + 13.2 (rotameric peaks), 10.2, 1.9 (br.) ppm.

^{11}B NMR (128 MHz, $CDCl_3$): δ_B 33.4 (br. s, 1B) ppm.

IR (film) ν_{max}: 2975, 1698, 1411, 1139, 1111, 855 cm^{-1}.

HRMS (ESI^+) calcd. for $C_{22}H_{33}BNO_4$ $[M+H]^+$ 386.2501, found 386.2500.

***tert*-Butyl 2-((1-(4,4,5,5-tetramethyl-1,3,2-dioxaborolan-2-yl)cyclopropyl)methyl)piperidine-1-carboxylate (10a)**

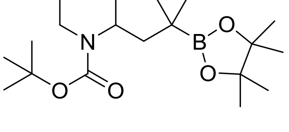

Prepared following General Procedure A with Boc-Pip-OH (69 mg, 0.30 mmol, 1.0 equiv.), 4CzIPN (2.4 mg, 0.0030 mmol, 1.0 mol%), Cs_2CO_3 (195 mg, 0.600 mmol, 2.00 equiv.), alkenyl boronic ester **1a** (96 μL, 0.45 mmol, 1.5 equiv.) and DMF (6.0 mL), which was irradiated with 1 × Kessil lamp for 20 h.

Purification by flash column chromatography (5% EtOAc/pentane) gave the title compound (97 mg, 0.26 mmol, 88%) as a white solid.

TLC: R_f = 0.48 (5% EtOAc/pentane, $KMnO_4$ stain).

Mpt: 77 – 78 °C (EtOAc).

^{1}H NMR (400 MHz, $CDCl_3$): δ_H 4.28 (br. s, 1H), 3.91 (br. d, *J* = 13.2 Hz, 1H), 2.81 (t, *J* = 12.9 Hz, 1H), 1.66 – 1.47 (m, 6H), 1.43 (s, 9H), 1.40 – 1.26 (m, 2H), 1.17 (s, 12H), 0.75 – 0.58 (m, 2H), 0.40 – 0.25 (m, 2H) ppm.

^{13}C NMR (101 MHZ, $CDCl_3$): δ_C 155.1, 83.0, 78.8, 51.2 (br.), 39.2 (br.), 35.8, 28.7, 28.4, 25.9, 24.9, 24.7, 19.5, 12.2, 11.3 (br.), 2.10 (br.) ppm.

^{11}B NMR (128 MHz, $CDCl_3$): δ_B 33.9 (br. s, 1B) ppm.

IR (film) ν_{max}: 2976 – 2930, 1686, 1414, 1364, 1140 cm^{-1}.

HRMS (ESI$^+$) calcd. for $C_{20}H_{36}BNNaO_4$ [M+Na]$^+$ 388.2633, found 388.2646.

***tert*-Butyl methyl(1-(1-(4,4,5,5-tetramethyl-1,3,2-dioxaborolan-2-yl)cyclopropyl)propan-2-yl)carbamate** (**11a**)

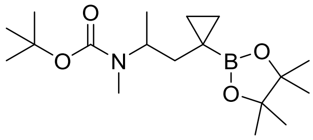

Prepared following General Procedure A using *N*-Me-Boc-Ala-OH (60 mg, 0.30 mmol, 1.0 equiv.), 4CzIPN (2.4 mg, 0.0030 mmol, 1.0 mol%), Cs_2CO_3 (195 mg, 0.600 mmol, 2.00 equiv.), alkenyl boronic ester **1a** (96 µL, 0.45 mmol, 1.5 equiv.) and DMF (6.0 mL), which was irradiated with 1 × Kessil lamp for 20 h. Purification by flash column chromatography (5% EtOAc/pentane) gave the title compound (88 mg, 0.26 mmol, 87%) as a colourless oil.

TLC: R_f = 0.28 (5% EtOAc/pentane, $KMnO_4$ stain).

^{1}H NMR (400 MHz, $CDCl_3$): δ_H 4.39 – 4.12 (br. m, 1H), 2.69 + 2.61 (rotameric peaks, 2 × br. s, 3H), 1.73 – 1.56 (m, 1H), 1.44 (s, 9H), 1.18 (s, 12H), 1.11 – 0.96 (m, 1H), 1.05 (d, *J* = 6.9 Hz, 3H), 0.71 – 0.57 (br. m, 2H), 0.31 – 0.23 (br. m, 2H) ppm.

^{13}C NMR (101 MHz, $CDCl_3$): δ_C 155.9, 83.0, 79.0 + 78.8 (rotameric peaks), 52.0 + 50.5 (rotameric peaks), 40.3 + 39.6 (rotameric peaks), 28.7, 28.4, 24.9, 18.6, 12.1 + 11.5 (rotameric peaks), 11.8 + 10.6 (rotameric peaks), 2.3 (br.) ppm.

^{11}B NMR (128 MHz, $CDCl_3$): δ_B 33.9 (br. s, 1B) ppm.

IR (film) ν_{max}: 2976, 1689, 1415, 1338, 1141, 854 cm^{-1}.

HRMS (ESI^+) calcd. for $C_{18}H_{35}BNO_4$ $[M+H]^+$ 340.2657, found 340.2660.

***tert*-Butyl (1-(1-(4,4,5,5-tetramethyl-1,3,2-dioxaborolan-2-yl)cyclopropyl)propan-2-yl)carbamate (12a)**

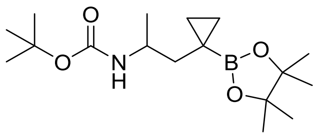

Prepared following General Procedure A with Boc-Ala-OH (57 mg, 0.30 mmol, 1.0 equiv.), 4CzIPN (4.7 mg, 0.0060 mmol, 2.0 mol%), Cs_2CO_3 (195 mg, 0.600 mmol, 2.00 equiv.), alkenyl boronic ester **1a** (96 μL, 0.45 mmol, 1.5 equiv.) and DMF (6.0 mL), which was irradiated with 1 × Kessil lamp for 20 h. Purification by flash column chromatography (10% EtOAc/pentane) gave the title compound (85 mg, 0.26 mmol, 87%) as a colourless oil.

TLC: R_f = 0.42 (10% EtOAc/pentane, $KMnO_4$ stain).

^{1}H NMR (400 MHz, $CDCl_3$): δ_H 5.35 (br. s, 1H), 3.65 – 3.53 (m, 1H), 1.73 (dd, *J* = 14.3, 10.2, Hz, 1H), 1.43 (s, 9H), 1.23 (s, 6H), 1.21 (s, 6H), 1.09 (d, *J* = 6.4 Hz, 3H), 0.93 (dd, *J* = 14.3, 4.3 Hz, 1H), 0.78 – 0.65 (m, 2H), 0.41 – 0.30 (m, 2H) ppm.

^{13}C NMR (101 MHZ, $CDCl_3$): δ_C 155.7, 83.5, 78.6, 47.5, 42.9, 28.7, 24.9, 24.7, 22.0, 13.8, 10.5 ppm. The carbon directly attached to boron was not detected due to the boron quadrupole.

^{11}B NMR (128 MHz, $CDCl_3$): δ_B 33.5 (br. s, 1B) ppm.

IR (film) ν_{max}: 3406, 2977, 1714, 1416, 1167, 1140 cm^{-1}.

HRMS (ESI^+) calcd. for $C_{17}H_{33}BNO_4$ $[M+H]^+$ 326.2500, found 326.2505.

***tert*-Butyl (3-methyl-1-(1-(4,4,5,5-tetramethyl-1,3,2-dioxaborolan-2-yl)cyclopropyl)butan-2-yl)carbamate (13a)**

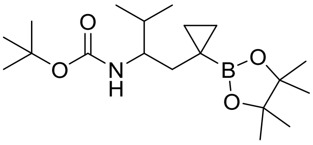

Prepared following General Procedure A with Boc-Val-OH (64 mg, 0.30 mmol, 1.0 equiv.), 4CzIPN (4.7 mg, 0.0060 mmol, 2.0 mol%), Cs_2CO_3 (195 mg, 0.600 mmol, 2.00 equiv.), alkenyl boronic ester **1a** (96 µL, 0.45 mmol, 1.5 equiv.) and DMF (6.0 mL), which was irradiated with 1 × Kessil lamp for 20 h. Purification by flash column chromatography (5% EtOAc/pentane) gave the title compound (65 mg, 0.18 mmol, 61%) as a colourless oil.

TLC: R_f = 0.34 (5% EtOAc/pentane, $KMnO_4$ stain).

^{1}H NMR (400 MHz, $CDCl_3$): δ_H 5.00 (d, *J* = 8.6 Hz, 1H), 3.42 (ddt, *J* = 12.1, 8.4, 4.1 Hz, 1H), 1.83 – 1.74 (m, 1H), 1.67 (dd, *J* = 14.3, 11.3 Hz, 1H), 1.42 (s, 9H), 1.22 (s, 6H), 1.21 (s, 6H), 0.88 (dd, *J* = 14.4, 4.0 Hz, 1H), 0.83 (d, *J* = 6.8 Hz, 3H), 0.82 (d, *J* = 6.9Hz, 3H), 0.75 – 0.64 (m, 2H), 0.40 – 0.27 (m, 2H) ppm.

^{13}C NMR (101 MHZ, $CDCl_3$): δ_C 156.0, 83.4, 78.5, 56.3, 36.4, 32.3, 28.6, 25.1, 24.7, 18.5, 18.2, 14.1, 9.9 ppm. The carbon directly attached to boron was not detected due to the boron quadrupole.

^{11}B NMR (128 MHz, $CDCl_3$): δ_B 34.3 (br. s, 1B) ppm.

IR (film) ν_{max}: 3417, 2976, 1716 – 1703, 1417, 1365, 1167, 1141 cm^{-1}.

HRMS (ESI$^+$) calcd. for $C_{19}H_{37}BNO_4$ [M+H]$^+$ 354.2814, found 354.2829.

***tert*-Butyl (3,3-dimethyl-1-(1-(4,4,5,5-tetramethyl-1,3,2-dioxaborolan-2-yl)cyclopropyl)butan-2-yl)carbamate (14a)**

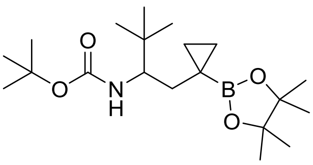

Prepared following General Procedure A with Boc-Tle-OH (69 mg, 0.30 mmol, 1.0 equiv.), 4CzIPN (4.7 mg, 0.0060 mmol, 2.0 mol%), Cs_2CO_3 (195 mg, 0.600 mmol, 2.00 equiv.), alkenyl boronic ester **1a** (96 µL, 0.45 mmol, 1.5 equiv.) and DMF (6.0 mL), which was irradiated with 1 × Kessil lamp for 20 h.

Purification by flash column chromatography (5% EtOAc/pentane) gave the title compound (59 mg, 0.16 mmol, 53%) as a colourless oil.

TLC: R_f = 0.40 (5% EtOAc/pentane, $KMnO_4$ stain).

^{1}H NMR (400 MHz, $CDCl_3$): δ_H (82:18 ratio of rotamers) 4.67 (d, *J* = 10.2 Hz, 0.82H), 4.33 (d, *J* = 10.4 Hz, 0.18H), 3.42 (ddd, *J* = 11.8, 10.1, 3.9 Hz, 0.82H), 3.24 (td, *J* = 11.4, 3.3 Hz, 0.18H), 1.58 (dd, *J* = 14.1, 11.7 Hz, 1H), 1.43 (s, 9H), 1.23 (s, 6H), 1.22 (s, 6H), 1.11 (dd, *J* = 14.1, 4.0 Hz, 1H), 0.85 (s, 9H), 0.75 – 0.62 (m, 2H), 0.43 – 0.24 (m, 2H) ppm.

^{13}C NMR (101 MHZ, $CDCl_3$): δ_C 156.2, 83.3, 78.5, 58.7, 35.3, 35.1, 28.7 + 28.6 (rotameric peaks), 26.6, 25.3 + 25.1 (rotameric peaks), 24.8, 14.0, 9.2 ppm. The carbon directly attached to boron was not detected due to the boron quadrupole.

^{11}B NMR (128 MHz, $CDCl_3$): δ_B 33.5 (br. s, 1B) ppm.

IR (film) ν_{max}: 3426, 2973, 1720 – 1702, 1418, 1365, 1169, 1141 cm^{-1}.

HRMS (ESI$^+$) calcd. for $C_{20}H_{38}BNNaO_4$ $[M+Na]^+$ 390.2790, found 390.2805.

***tert*-Butyl (2-(1-(4,4,5,5-tetramethyl-1,3,2-dioxaborolan-2-yl)cyclopropyl)ethyl)carbamate (15a)**

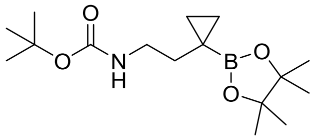

Prepared following General Procedure A using Boc-Gly-OH (53 mg, 0.30 mmol, 1.0 equiv.), 4CzIPN (4.7 mg, 0.0060 mmol, 2.0 mol%), Cs_2CO_3 (195 mg, 0.600 mmol, 2.00 equiv.), alkenyl boronic ester **1a** (96 µL, 0.45 mmol, 1.5 equiv.) and DMF (6.0 mL), which was irradiated with 1 × Kessil lamp for 62 h. Purification by flash column chromatography (5% EtOAc/pentane) gave the title compound (52 mg, 0.17 mmol, 55%) as a colourless oil.

TLC: R_f = 0.28 (5% EtOAc/pentane, $KMnO_4$ stain).

^{1}H NMR (400 MHz, $CDCl_3$): δ_H 5.34 – 5.28 (br. m, 1H), 3.17 – 3.13 (m, 2H), 1.43 (s, 9H), 1.38 (t, *J* = 6.5 Hz, 2H), 1.21 (s, 12H), 0.70 (q, *J* = 3.5 Hz, 2H), 0.34 (q, *J* = 3.5 Hz, 2H) ppm.

^{13}C NMR (101 MHz, $CDCl_3$): δ_C 156.2, 83.5, 78.7, 41.0, 35.7, 28.6, 24.8, 11.9 ppm. The carbon directly attached to boron was not detected due to the boron quadrupole.

^{11}B NMR (128 MHz, $CDCl_3$): δ_B 33.4 (br. s, 1B) ppm.

IR (film) ν_{max}: 3395, 2926, 1694, 1417, 1168, 1143, 856 cm^{-1}.

HRMS (ESI$^+$) calcd. for $C_{16}H_{31}BNO_4$ [M+H]$^+$ 312.2344, found 312.2345.

Methyl 1-(2-((*tert*-butoxycarbonyl)amino)ethyl)cyclopropane-1-carboxylate (**15b**)

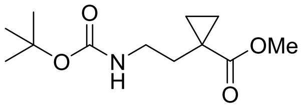

Prepared following General Procedure A using Boc-Gly-OH (35 mg, 0.20 mmol, 1.0 equiv.), 4CzIPN (1.6 mg, 0.0020 mmol, 1.0 mol%), Cs_2CO_3 (130 mg, 0.400 mmol, 2.00 equiv.), methyl 4-chloro-2-methylenebutanoate (**1b**) (59 mg, 0.40 mmol, 2.0 equiv.) and DMF (4.0 mL), which was irradiated with 1 × Kessil lamp for 15 h. Purification by flash column chromatography (50% EtOAc/hexane) gave the title compound (35 mg, 0.14 mmol, 72%) as a colourless oil.

TLC: R_f = 0.29 (20% EtOAc/hexane, $KMnO_4$ stain).

^{1}H NMR (400 MHz, DMSO-d_6): δ_H 6.73 (t, J = 5.5 Hz, 1H), 3.57 (s, 3H), 3.08 – 2.99 (m, 2H), 1.59 (t, J = 7.4 Hz, 2H), 1.36 (s, 9H), 1.08 – 1.04 (m, 2H), 0.77 – 0.71 (m, 2H) ppm.

^{13}C NMR (101 MHz, DMSO-d_6): δ_C 174.9, 155.9, 77.8, 52.1, 38.9, 33.9, 28.7, 21.5, 15.3 ppm.

IR (film) ν_{max}: 3425, 2975, 2980, 1704, 1529, 1451, 1365, 1162, 822 cm^{-1}.

HRMS (ESI$^+$): calcd. for $C_{12}H_{21}NNaO_4$ [M+Na]$^+$ 266.1363, found 266.1373.

***tert*-Butyl (2-methyl-1-(1-(4,4,5,5-tetramethyl-1,3,2-dioxaborolan-2-yl)cyclopropyl)propan-2-yl)carbamate** (**16a**)

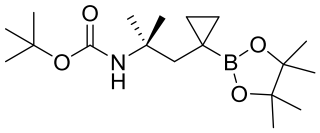

Prepared following General Procedure A using Boc-Aib-OH (61 mg, 0.30 mmol, 1.0 equiv.), 4CzIPN (4.7 mg, 0.0060 mmol, 2.0 mol%), Cs_2CO_3 (195 mg, 0.600 mmol, 2.00 equiv.), alkenyl boronic ester **1a** (96 µL, 0.45 mmol, 1.5 equiv.) and DMF (6.0 mL), which was irradiated with 1 × Kessil lamp for 24 h. Purification by flash column chromatography (5% EtOAc/pentane) gave the title compound (59 mg, 0.17 mmol, 58%) as a colourless oil.

TLC: R_f = 0.48 (5% EtOAc/pentane, $KMnO_4$ stain).

^{1}H NMR (400 MHz, $CDCl_3$): δ_H 5.97 (br. s, 1H), 1.43 (s, 9H + 2H), 1.33 (s, 6H), 1.23 (s, 12H), 0.73 (q, J = 3.6 Hz, 2H), 0.38 (q, J = 3.6 Hz, 2H) ppm.

^{13}C NMR (101 MHz, $CDCl_3$): δ_C 155.0, 83.6, 78.2, 54.2, 49.5, 28.8, 27.0, 24.6, 12.2 ppm. The carbon directly attached to boron was not detected due to the boron quadrupole.

^{11}B NMR (128 MHz, $CDCl_3$): δ_B 33.9 (br. s, 1B) ppm.

IR (film) ν_{max}: 3391, 2977, 1720, 1520, 1416, 1168, 1066, 851 cm^{-1}.

HRMS (ESI^+) calcd. for $C_{18}H_{35}BNO_4$ $[M+H]^+$ 340.2657, found 340.2667.

Methyl 1-(2-((*tert*-butoxycarbonyl)amino)-2-methylpropyl)cyclopropane-1-carboxylate (16b)

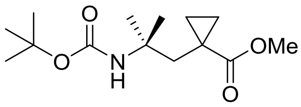

Prepared following General Procedure A using Boc-Aib-OH (61 mg, 0.30 mmol, 1.0 equiv.), 4CzIPN (2.4 mg, 0.0300 mmol, 1.0 mol%), Cs_2CO_3 (195 mg, 0.600 mmol, 2.00 equiv.), methyl 4-chloro-2-methylenebutanoate (**1b**) (89 mg, 0.60 mmol, 2.0 equiv.) and DMF (6.0 mL), which was irradiated with 1 × Kessil lamp for 15 h. Purification by flash column chromatography (30% EtOAc/hexane) gave the title compound (73 mg, 0.27 mmol, 90%) as a colourless oil.

TLC: R_f = 0.37 (30% EtOAc/hexane, $KMnO_4$ stain).

^{1}H NMR (400 MHz, $CDCl_3$): δ_H 4.97 (s, 1H), 3.63 (s, 3H), 1.99 (s, 2H), 1.39 (s, 9H), 1.27 (s, 6H), 1.19 – 1.16 (m, 2H), 0.80 – 0.75 (m, 2H) ppm.

^{13}C NMR (101 MHz, $CDCl_3$): δ_C 175.8, 154.4, 78.5, 53.6, 51.8, 43.0, 28.5, 27.7, 21.0, 14.9 ppm.

IR (film) ν_{max}: 3376, 2974, 2934, 1710, 1513, 1454, 1364, 1279, 1161, 1073, 869 cm^{-1}.

HRMS (ESI^+): calcd. for $C_{14}H_{25}NNaO_4$ $[M+Na]^+$ 294.1676, found 294.1689.

***tert*-Butyl (1-phenyl-3-(1-(4,4,5,5-tetramethyl-1,3,2-dioxaborolan-2-yl)cyclopropyl)propan-2-yl)carbamate (17a)**

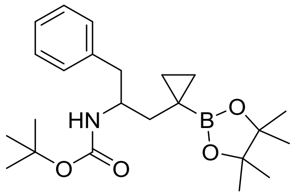

Prepared following General Procedure A with Boc-Phe-OH (80 mg, 0.30 mmol, 1.0 equiv.), 4CzIPN (4.7 mg, 0.0060 mmol, 2.0 mol%), Cs_2CO_3 (195 mg, 0.600 mmol, 2.00 equiv.), alkenyl boronic ester **1a** (96 µL, 0.45 mmol, 1.5 equiv.) and DMF (6.0 mL), which was irradiated with 1 × Kessil lamp for 20 h. Purification by flash column chromatography (10% EtOAc/pentane) gave the title compound (109 mg, 0.27 mmol, 91%) as a colourless oil.

TLC: R_f = 0.32 (10% EtOAc/pentane, $KMnO_4$ stain).

^{1}H NMR (400 MHz, $CDCl_3$): δ_H 7.30 – 7.21 (m, 2H), 7.21 – 7.12 (m, 3H), 5.38 (br. d, *J* = 7.4 Hz, 1H), 3.72 (dtt *J* = 11.3, 7.5, 3.7 Hz, 1H), 2.96 (dd, *J* = 13.3, 4.7 Hz, 1H), 2.58 (dd, *J* = 13.3, 8.3 Hz, 1H), 1.74 (dd, *J* = 14.3, 11.0 Hz, 1H), 1.43 (s, 9H), 1.23 (s, 6H), 1.21 (s, 6H), 0.83 (dd, *J* = 14.4, 3.6 Hz, 1H), 0.74 – 0.59 (m, 2H), 0.34 – 0.15 (m, 2H) ppm.

^{13}C NMR (101 MHZ, $CDCl_3$): δ_C 155.7, 139.1, 129.6, 128.2, 126.0, 83.5, 78.7, 53.1, 42.3, 39.5, 28.6, 25.0, 24.6, 14.1, 10.1 ppm. The carbon directly attached to boron was not detected due to the boron quadrupole.

^{11}B NMR (128 MHz, $CDCl_3$): δ_B 34.7 (br. s, 1B) ppm.

IR (film) ν_{max}: 3407, 2977, 1712, 1505, 1417, 1365, 1167, 1139 cm^{-1}.

HRMS (ESI^+) calcd. for $C_{23}H_{37}BNO_4$ $[M+H]^+$ 402.2814, found 402.2815.

***tert*-Butyl (1-(1-benzyl-1H-imidazol-4-yl)-3-(1-(4,4,5,5-tetramethyl-1,3,2-dioxaborolan-2-yl)cyclopropyl)propan-2-yl)carbamate** (**18a**)

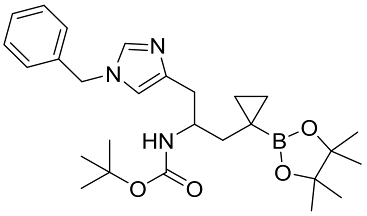

Prepared following General Procedure A using Boc-His(Bzl)-OH (103 mg, 0.300 mmol, 1.00 equiv.), 4CzIPN (4.7 mg, 0.0060 mmol, 2.0 mol%), Cs_2CO_3 (195 mg, 0.600 mmol, 2.00 equiv.), alkenyl boronic ester **1a** (96 μL, 0.45 mmol, 1.5 equiv.) and DMF (6.0 mL), which was irradiated with 1 × Kessil lamp for 24 h. Purification by flash column chromatography (10% $MeOH/CH_2Cl_2$) gave the title compound (130 mg, 0.270 mmol, 90%) as a colourless oil.

TLC: R_f = 0.50 (10% $MeOH/CH_2Cl_2$, $KMnO_4$ stain).

^{1}H NMR (400 MHz, $CDCl_3$): δ_H 7.40 (br. s, 1H), 7.33 – 7.25 (m, 3H), 7.13 – 7.07 (m, 2H), 6.69 (br. s, 1H), 5.39 (br. d, *J* = 7.5 Hz, 1H), 5.00 (s, 2H), 3.77 – 3.68 (m, 1H), 2.79 (dd, *J* = 14.4, 4.6 Hz, 1H), 2.67 (dd, *J* = 14.2, 7.1 Hz, 1H), 1.63 (dd, *J* = 14.0, 10.3 Hz, 1H), 1.38 (s, 9H), 1.22 – 1.13 (m, 1H), 1.18 (s, 6H), 1.17 (s, 6H), 0.67 – 0.58 (br. m, 2H), 0.29 – 0.22 (br. m, 2H) ppm.

^{13}C NMR (101 MHz, $CDCl_3$): δ_C 155.6, 140.2, 136.4, 136.2, 129.0, 128.2, 127.3, 116.8, 83.1, 78.4, 51.8, 50.9, 40.4, 34.3, 28.6, 24.9, 24.7, 13.3, 10.4, 2.4 (br.) ppm.

^{11}B NMR (128 MHz, $CDCl_3$): δ_B 34.3 (br. s, 1B) ppm.

IR (film) ν_{max}: 3404, 2977, 1699, 1498, 1165, 1139 cm^{-1}.

HRMS (ESI^+) calcd. for $C_{27}H_{40}BN_3NaO_4$ $[M+Na]^+$ 504.3009, found 504.2993.

Methyl 1-(3-(1-benzyl-1*H*-imidazol-4-yl)-2-((*tert*-butoxycarbonyl)amino)propyl)cyclopropane-1-carboxylate (**18b**)

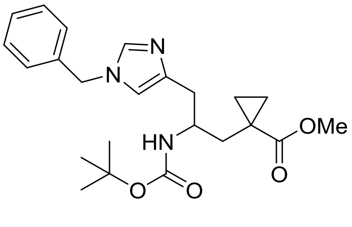

Prepared following General Procedure A using Boc-His(Bzl)-OH (69 mg, 0.20 mmol, 1.0 equiv.), 4CzIPN (1.6 mg, 0.0020 mmol, 1.0 mol%), Cs_2CO_3 (130 mg, 0.400 mmol, 2.00 equiv.), methyl 4-chloro-2-methylenebutanoate (**1b**) (59 mg, 0.40 mmol, 2.0 equiv.) and DMF (4.0 mL), which was

irradiated with 1 × Kessil lamp for 15 h. Purification by flash column chromatography (10% MeOH/EtOAc) gave the title compound (73 mg, 0.18 mmol, 88%) as a colourless oil.

TLC: R_f = 0.30 (10% MeOH/EtOAc, $KMnO_4$ stain).

^{1}H NMR (400 MHz, $CDCl_3$): δ_H 7.43 (s, 1H), 7.38 – 7.30 (m, 3H), 7.16 – 7.11 (m, 2H), 6.72 (s, 1H), 5.25 (d, *J* = 8.2 Hz, 1H), 5.05 (s, 2H), 4.14 – 4.03 (m, 1H), 3.64 (s, 3H), 2.84 – 2.66 (m, 2H), 1.88 – 1.77 (m, 1H), 1.66 – 1.55 (m, 1H), 1.39 (s, 9H), 1.29 – 1.11 (m, 2H), 0.85 – 0.76 (m, 1H), 0.70 – 0.61 (m, 1H) ppm.

^{13}C NMR (101 MHz, $CDCl_3$): δ_C 175.7, 155.4, 139.7, 136.6, 136.2, 128.9, 128.1, 127.1, 116.8, 78.5, 51.7, 50.7, 49.5, 38.4, 33.6, 28.4, 21.3, 16.1, 14.9 ppm.

IR (film) ν_{max}: 3362, 2974, 2921, 1702, 1498, 1437, 1364, 1161, 910 cm^{-1}.

HRMS (ESI^+): calcd. for $C_{23}H_{31}N_3NaO_4$ $[M+Na]^+$ 436.2207, found 436.2207.

***tert*-Butyl (4-(methylthio)-1-(1-(4,4,5,5-tetramethyl-1,3,2-dioxaborolan-2-yl)cyclopropyl)butan-2-yl)carbamate (19a)**

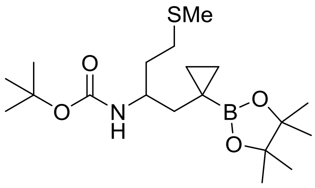

Prepared following General Procedure A with Boc-Met-OH (75 mg, 0.30 mmol, 1.0 equiv.), 4CzIPN (4.7 mg, 0.0060 mmol, 2.0 mol%), Cs_2CO_3 (195 mg, 0.600 mmol, 2.00 equiv.), alkenyl boronic ester **1a** (96 µL, 0.45 mmol, 1.5 equiv.) and DMF (6.0 mL), which was irradiated with 1 × Kessil lamp for 24 h. Purification by flash column chromatography (10% EtOAc/pentane) gave the title compound (92 mg, 0.24 mmol, 79%) as a colourless oil.

TLC: R_f = 0.43 (10% EtOAc/pentane, $KMnO_4$ stain).

^{1}H NMR (400 MHz, $CDCl_3$): δ_H 5.19 (d, *J* = 8.1 Hz, 1H), 3.66 – 3.54 (m, 1H), 2.47 (t, *J* = 8.0 Hz, 2H), 2.07 (s, 3H), 1.77 – 1.64 (m, 3H), 1.41 (s, 9H), 1.22 (s, 6H), 1.20 (s, 6H), 1.01 (dd, *J* = 14.5, 4.2 Hz, 1H), 0.75 – 0.66 (m, 2H), 0.40 – 0.30 (m, 2H) ppm.

^{13}C NMR (101 MHZ, $CDCl_3$): δ_C 155.8, 83.5, 78.8, 51.0, 40.7, 36.0, 30.8, 28.6, 25.0, 24.6, 15.7, 13.9, 10.3 ppm. The carbon directly attached to boron was not detected due to the boron quadrupole.

^{11}B NMR (128 MHz, $CDCl_3$): δ_B 34.5 (br. s, 1B) ppm.

IR (film) ν_{max}: 3405, 2977, 2918, 1713, 1417, 1365, 1167, 1139 cm^{-1}.

HRMS (ESI$^+$) calcd. for $C_{19}H_{37}BNO_4S$ [M+H]$^+$ 386.2534, found 386.2536.

Methyl 1-(2-((*tert*-butoxycarbonyl)amino)-4-(methylthio)butyl)cyclopropane-1-carboxylate (**19b**)

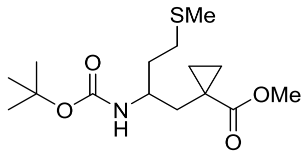

Prepared following General Procedure A using Boc-Met-OH (75 mg, 0.30 mmol, 1.0 equiv.), 4CzIPN (2.4 mg, 0.0030 mmol, 1.0 mol%), Cs_2CO_3 (195 mg, 0.60 mmol, 2.0 equiv.), methyl 4-chloro-2-methylenebutanoate (**1b**) (89 mg, 0.60 mmol, 2.0 equiv.) and DMF (6.0 mL), which was irradiated with 1 × Kessil lamp for 15 h. Purification by flash column chromatography (30% EtOAc/hexane) gave the title compound (86 mg, 0.27 mmol, 90%) as a colourless oil.

TLC: R_f = 0.25 (30% EtOAc/hexane, $KMnO_4$ stain).

^{1}H NMR (400 MHz, $CDCl_3$): δ_H 4.56 (d, J = 9.5 Hz, 1H), 3.91 – 3.77 (m, 1H), 3.64 (s, 3H), 2.60 – 2.43 (m, 2H), 2.08 (s, 3H), 1.84 – 1.59 (m, 4H), 1.40 (s, 9H), 1.29 – 1.17 (m, 2H), 0.86 – 0.65 (m, 2H) ppm.

^{13}C NMR (101 MHz, $CDCl_3$): δ_C 175.7, 155.5, 78.9, 51.8, 49.5, 38.9, 35.5, 30.7, 28.4, 21.3, 16.7, 15.5, 14.5 ppm.

IR (film) ν_{max}: 3372, 2976, 2918, 1700, 1512, 1437, 1365, 1165, 1047, 1027, 912 cm^{-1}.

HRMS (ESI$^+$): calcd. for $C_{15}H_{27}NNaO_4S$ [M+Na]$^+$ 340.1553, found 340.1578.

Benzyl 4-((*tert*-butoxycarbonyl)amino)-5-(1-(4,4,5,5-tetramethyl-1,3,2-dioxaborolan-2-yl)cyclopropyl)pentanoate (**20a**)

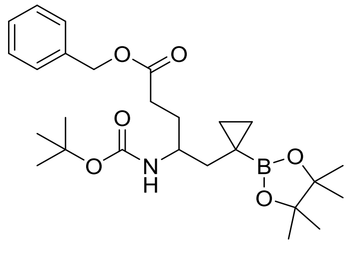

Prepared following General Procedure A using Boc-Glu(OBzl)-OH (101 mg, 0.300 mmol, 1.00 equiv.), 4CzIPN (4.7 mg, 0.0060 mmol, 2.0 mol%), Cs_2CO_3 (195 mg, 0.600 mmol, 2.00 equiv.), alkenyl boronic

ester **1a** (96 μL, 0.45 mmol, 1.5 equiv.) and DMF (6.0 mL), which was irradiated with 1 × Kessil lamp for 24 h. Purification by flash column chromatography (10% EtOAc/pentane) gave the title compound (78 mg, 0.17 mmol, 55%) as a colourless oil.

TLC: R_f = 0.15 (10% EtOAc/pentane, $KMnO_4$ stain).

^{1}H NMR (400 MHz, $CDCl_3$): δ_H 7.36 – 7.28 (m, 5H), 5.16 – 5.07 (m, 3H), 3.64 – 3.53 (br. m, 1H), 2.40 (t, *J* = 7.9 Hz, 2H), 1.88 – 1.79 (m, 1H), 1.74 – 1.65 (m, 1H), 1.48 – 1.38 (m, 1H), 1.42 (s, 9H), 1.22 (s, 6H), 1.20 (s, 6H), 1.02 (dd, *J* = 14.1, 3.6 Hz, 1H), 0.74 – 0.67 (m, 2H), 0.37 – 0.31 (m, 2H) ppm.

^{13}C NMR (101 MHz, $CDCl_3$): δ_C 173.6, 155.9, 136.2, 128.6, 128.3, 128.2, 83.5, 78.8, 66.3, 50.9, 40.8, 31.4, 31.1, 28.6, 25.0, 24.6, 13.8, 10.3, 1.8 (br.) ppm.

^{11}B NMR (128 MHz, $CDCl_3$): δ_B 34.3 (br. s, 1B) ppm.

IR (film) ν_{max}: 3404, 2977, 1712, 1417, 1246, 1165, 1138, 855 cm^{-1}.

HRMS (ESI^+) calcd. for $C_{26}H_{41}BNO_6$ $[M+H]^+$ 474.3026, found 474.3024.

***tert*-Butyl (5-amino-5-oxo-1-(1-(4,4,5,5-tetramethyl-1,3,2-dioxaborolan-2-yl)cyclopropyl)pentan-2-yl)carbamate** (**21a**)

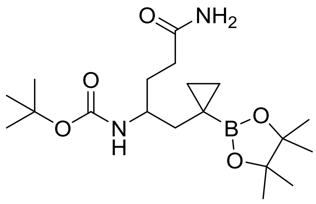

Prepared following General Procedure A with Boc-Gln-OH (74 mg, 0.30 mmol, 1.0 equiv.), 4CzIPN (4.7 mg, 0.0060 mmol, 2.0 mol%), Cs_2CO_3 (195 mg, 0.600 mmol, 2.00 equiv.), alkenyl boronic ester **1a** (96 μL, 0.45 mmol, 1.5 equiv.) and DMF (6.0 mL), which was irradiated with 1 × Kessil lamp for 24 h. Purification by flash column chromatography (100% EtOAc) gave the title compound (94 mg, 0.25 mmol, 82%) as a white solid.

TLC: R_f = 0.20 (100% EtOAc, $KMnO_4$ stain).

Mpt: 165 – 166 °C (EtOAc).

^{1}H NMR (400 MHz, $CDCl_3$): δ_H 7.09 (br. s, 1H), 5.45 (br. d, *J* = 8.2 Hz, 1H), 5.30 (br. s, 1H), 3.66 – 3.51 (m, 1H), 2.30 (ddd, *J* = 14.4, 8.9, 5.5 Hz, 1H), 2.19 (ddd, *J* = 14.5, 7.1, 5.3 Hz, 1H), 1.78 – 1.62 (m, 3H), 1.44 (s, 9H), 1.23 (s, 6H), 1.21 (s, 6H), 1.00 (dd, *J* = 14.4, 3.9 Hz, 1H), 0.78 – 0.67 (m, 2H), 0.42 – 0.29 (m, 2H) ppm.

^{13}C NMR (101 MHz, $CDCl_3$): δ_C 176.0, 157.0, 83.6, 79.3, 50.7, 41.0, 33.0, 32.8, 28.6, 25.1, 24.5, 14.3, 10.1 ppm. The carbon directly attached to boron was not detected due to the boron quadrupole.

^{11}B NMR (128 MHz, $CDCl_3$): δ_B 33.5 (br. s, 1B) ppm.

IR (film) ν_{max}: 3345, 3174, 2977, 2922, 1681, 1417, 1168, 1141 cm^{-1}.

HRMS (ESI$^+$) calcd. for $C_{19}H_{36}BN_2O_5$ [M+H]$^+$ 383.2715, found 383.2734.

Benzyl (2-oxo-2-((1-phenyl-3-(1-(4,4,5,5-tetramethyl-1,3,2-dioxaborolan-2-yl)cyclopropyl)propan-2-yl)amino)ethyl)carbamate (**22a**)

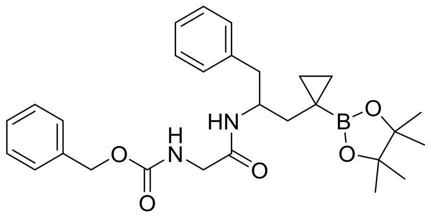

Prepared following General Procedure A with Z-Gly-Phe-OH (107 mg, 0.300 mmol, 1.00 equiv.), 4CzIPN (4.7 mg, 0.0060 mmol, 2.0 mol%), Cs_2CO_3 (195 mg, 0.600 mmol, 2.00 equiv.), alkenyl boronic ester **1a** (96 µL, 0.45 mmol, 1.5 equiv.) and DMF (6.0 mL), which was irradiated with 1 × Kessil lamp for 24 h. Purification by flash column chromatography (40% EtOAc/pentane) gave the title compound (56 mg, 0.11 mmol, 38%) as a colourless oil.

TLC: R_f = 0.31 (40% EtOAc/pentane, PMA stain).

^{1}H NMR (400 MHz, $CDCl_3$): δ_H 7.43 – 7.29 (m, 5H), 7.27 – 7.22 (m, 2H), 7.21 – 7.11 (m, 3H), 6.39 (br. d, *J* = 7.7 Hz, 1H), 5.45 (br. s, 1H), 5.12 (s, 2H), 4.26 – 4.11 (m, 1H), 3.80 (d, *J* = 5.1 Hz, 2H), 2.87 (dd, *J* = 13.5, 5.7 Hz, 1H), 2.71 (dd, *J* = 13.5, 7.3 Hz, 1H), 1.48 (dd, *J* = 14.5, 10.0 Hz, 1H), 1.28 – 1.25 (m, 1H), 1.23 (s, 6H), 1.19 (s, 6H), 0.80 – 0.59 (m, 2H), 0.34 – 0.17 (m, 2H) ppm.

^{13}C NMR (101 MHZ, $CDCl_3$): δ_C 167.9, 156.4, 138.5, 136.5, 129.5, 128.7, 128.4, 128.3, 128.2, 126.4, 83.6, 67.1, 51.7, 44.6, 41.3, 39.9, 25.3, 24.4, 14.1, 10.8 ppm. The carbon directly attached to boron was not detected due to the boron quadrupole.

^{11}B NMR (128 MHz, $CDCl_3$): δ_B 33.7 (br. s, 1B) ppm.

IR (film) ν_{max}: 3318, 2977, 2929, 1715, 1662, 1416, 1137 cm^{-1}.

HRMS (ESI$^+$) calcd. for $C_{23}H_{37}BNO_4$ [M+H]$^+$ 402.2814, found 402.2815.

Methyl 1-(2-(2-(((benzyloxy)carbonyl)amino)acetamido)-3-phenylpropyl)cyclopropane-1-carboxylate (**22b**)

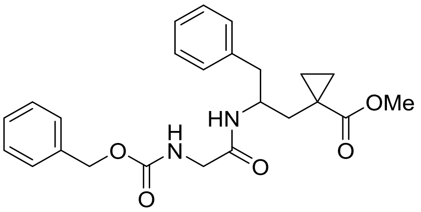

Prepared following General Procedure A using Z-Gly-Phe-OH (71 mg, 0.20 mmol, 1.0 equiv.), 4CzIPN (1.6 mg, 0.0020 mmol, 1.0 mol%), Cs_2CO_3 (130 mg, 0.400 mmol, 2.00 equiv.), methyl 4-chloro-2-methylenebutanoate (**1b**) (59 mg, 0.40 mmol, 2.0 equiv.) and DMF (4.0 mL), which was irradiated with 1 × Kessil lamp for 15 h. Purification by flash column chromatography (50% EtOAc/hexane) gave the title compound (53 mg, 0.12 mmol, 62%) as a colourless oil.

TLC: R_f = 0.28 (50% EtOAc/hexane, $KMnO_4$ stain).

^{1}H NMR (400 MHz, $CDCl_3$): δ_H δ 7.39 – 7.29 (m, 5H), 7.28 – 7.23 (m, 2H), 7.23 – 7.11 (m, 3H), 6.49 (d, *J* = 8.2 Hz, 1H), 5.45 (br. s, 1H), 5.12 (s, 2H), 4.33 – 4.19 (m, 1H), 3.76 (d, *J* = 5.6 Hz, 2H), 3.58 (s, 3H), 2.87 (dd, *J* = 13.6, 5.8 Hz, 1H), 2.70 (dd, *J* = 13.5, 7.4 Hz, 1H), 1.96 (dd, *J* = 14.9, 10.8 Hz, 1H), 1.45 (dd, *J* = 14.9, 3.7 Hz, 1H), 1.28 – 1.13 (m, 2H), 0.69 – 0.59 (m, 2H) ppm.

^{13}C NMR (101 MHz, $CDCl_3$): δ_C 176.4, 168.2, 156.4, 137.8, 136.2, 129.3, 128.5, 128.3, 128.1, 128.0, 126.4, 67.0, 52.0, 50.4, 44.6, 41.4, 37.1, 21.6, 17.5, 14.1 ppm.

IR (film) ν_{max}: 3321, 2987, 2922, 1715, 1658, 1521, 1453, 1347, 1240, 1147, 1047 cm^{-1}.

HRMS (ESI^+): calcd. for $C_{24}H_{28}N_2NaO_5$ $[M+Na]^+$ 447.1890, found 447.1890.

Benzyl ((2*S*)-1-((4-methyl-1-oxo-1-(1-(4,4,5,5-tetramethyl-1,3,2-dioxaborolan-2-yl)cyclopropyl)pentan-2-yl)amino)-1-oxo-3-phenylpropan-2-yl)carbamate (**23a**)

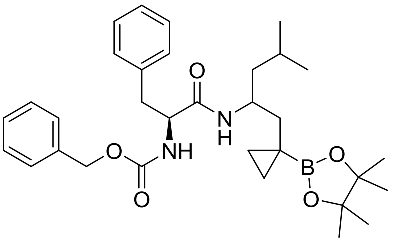

Prepared following General Procedure A using Z-Phe-Leu-OH (124 mg, 0.300 mmol, 1.00 equiv.), 4CzIPN (4.7 mg, 0.0060 mmol, 2.0 mol%), Cs_2CO_3 (195 mg, 0.600 mmol, 2.00 equiv.), alkenyl boronic ester **1a** (96 µL, 0.45 mmol, 1.5 equiv.) and DMF (6.0 mL), which was irradiated with 1 × Kessil lamp

for 24 h. Purification by flash column chromatography (15% EtOAc/pentane) gave the title compound (98 mg, 0.18 mmol, 61%) as a colourless oil. The d.r. was determined to be 50:50 by high temperature NMR in DMSO-d_6.

TLC: R_f = 0.15 (15% EtOAc/pentane, $KMnO_4$ stain).

^{1}H NMR (500 MHz, DMSO-d_6, 100°C): 1:1 ratio of diastereomers: δ_H 7.33 – 7.19 (m, 10H), 7.07 (br. m, 1H), 6.86 – 6.74 (br. m, 1H), 4.98 (s, 2H), 4.27 – 4.21 (m, 1H), 4.10 – 4.00 (m, 1H), 3.05 – 3.01 (m, 1H), 2.84 – 2.79 (m, 1H), 1.66 – 1.42 (m, 2H), 1.31 – 1.22 (m, 2H), 1.19 (s, 12H), 1.03 – 0.92 (m, 1H), 0.89 – 0.82 (m, 6H), 0.60 – 0.54 + 0.52 – 0.46 (diastereomeric peaks, 2 × m, 2H), 0.44 – 0.37 + 0.24 – 0.18 (diastereomeric peaks, 2 × m, 2H) ppm.

^{13}C NMR (126 MHz, DMSO-d_6, 100°C): 1:1 ratio of diastereomers: δ_C 169.5 + 169.4 (diastereomeric peaks), 155.0 + 154.9 (diastereomeric peaks), 137.6 + 137.5 (diastereomeric peaks), 136.6, 128.59 + 128.57 (diastereomeric peaks), 127.6, 127.4, 127.0, 126.8, 125.57 + 125.55 (diastereomeric peaks), 82.23 + 82.22 (diastereomeric peaks), 64.89 + 64.88 (diastereomeric peaks), 55.9, 46.91 + 46.85 (diastereomeric peaks), 43.9 + 43.7 (diastereomeric peaks), 41.3 + 41.2 (diastereomeric peaks), 37.7 + 37.4 (diastereomeric peaks), 24.09 + 24.08 (diastereomeric peaks), 23.87 + 23.85 (diastereomeric peaks), 23.80 + 23.79 (diastereomeric peaks), 22.7 + 22.6 (diastereomeric peaks), 21.4 + 21.3 (diastereomeric peaks), 10.8 + 10.7 (diastereomeric peaks), 9.9 + 9.7 (diastereomeric peaks) ppm. The carbon directly attached to boron was not detected due to the boron quadrupole.

^{11}B NMR (128 MHz, $CDCl_3$): δ_B 33.9 (br. s, 1B) ppm.

IR (film) ν_{max}: 3417, 3315, 3064, 2955, 1652, 1415, 1258, 1140, 1027, 909, 854 cm^{-1}.

HRMS (ESI$^+$) calcd. for $C_{32}H_{46}BN_2O_5$ [M+H]$^+$ 549.3500, found 549.3500.

4,4,5,5-Tetramethyl-2-(1-((tetrahydrofuran-2-yl)methyl)cyclopropyl)-1,3,2-dioxaborolane (24a)

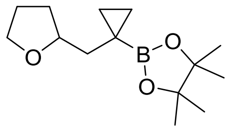

Prepared following General Procedure A with tetrahydro-2-furoic acid (29 μL, 0.30 mmol, 1.0 equiv.), 4CzIPN (4.7 mg, 0.0060 mmol, 2.0 mol%), Cs_2CO_3 (195 mg, 0.600 mmol, 2.00 equiv.), alkenyl boronic ester **1a** (96 μL, 0.45 mmol, 1.5 equiv.) and DMF (6.0 mL), which was irradiated with 1 × Kessil lamp for 24 h. Purification by flash column chromatography (7% EtOAc/pentane) gave the title compound (58 mg, 0.23 mmol, 76%) as a colourless oil.

TLC: R_f = 0.31 (7% EtOAc/pentane, $KMnO_4$ stain).

^{1}H NMR (400 MHz, $CDCl_3$): δ_H 3.94 (dddd, *J* = 6.3, 6.3, 6.3, 7.3 Hz, 1H), 3.82 (ddd, *J* = 8.1, 7.2, 6.6 Hz, 1H), 3.70 (ddd, *J* = 8.0, 8.0, 5.8 Hz, 1H), 2.02 – 1.74 (m, 3H), 1.63 (dd, *J* = 13.6, 7.0 Hz, 1H), 1.50 (dddd, *J* = 11.6, 8.5, 7.6, 7.6 Hz, 1H), 1.29 (dd, *J* = 13.6, 7.3 Hz, 1H), 1.20 (s, 6H), 1.20 (s, 6H), 0.72 – 0.61 (m, 2H), 0.46 – 0.28 (m, 2H) ppm.

^{13}C NMR (101 MHZ, $CDCl_3$): δ_C 83.0, 79.7, 67.4, 41.9, 31.6, 25.9, 24.8, 24.7, 11.12, 11.07 ppm. The carbon directly attached to boron was not detected due to the boron quadrupole.

^{11}B NMR (128 MHz, $CDCl_3$): δ_B 33.3 (br. s, 1B) ppm.

IR (film) ν_{max}: 2976 – 2868, 1416, 1315, 1143, 1066 cm^{-1}.

HRMS (ESI^+) calcd. for $C_{14}H_{26}BO_3$ $[M+H]^+$ 253.1972, found 253.1978.

Methyl 1-((tetrahydrofuran-2-yl)methyl)cyclopropane-1-carboxylate (**24b**)

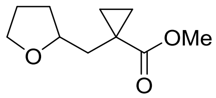

Prepared following General Procedure A using tetrahydro-2-furoic acid (23 mg, 0.20 mmol, 1.0 equiv.), 4CzIPN (1.6 mg, 0.0020 mmol, 1.0 mol%), Cs_2CO_3 (130 mg, 0.400 mmol, 2.00 equiv.), methyl 4-chloro-2-methylenebutanoate (**1b**) (59 mg, 0.40 mmol, 2.0 equiv.) and DMF (4.0 mL), which was irradiated with 1 × Kessil lamp for 15 h. Purification by flash column chromatography (10% EtOAc/pentane) gave the title compound (28 mg, 0.15 mmol, 77%) as a colourless oil.

TLC: R_f = 0.28 (10% EtOAc/pentane, $KMnO_4$ stain).

^{1}H NMR (400 MHz, $CDCl_3$): δ_H 4.06 – 3.94 (m, 1H), 3.84 – 3.74 (m, 1H), 3.73 – 3.62 (m, 1H), 3.62 (s, 3H), 2.06 – 1.93 (m, 1H), 1.91 (dd, *J* = 14.4, 4.9 Hz, 1H), 1.90 – 1.75 (m, 2H), 1.65 (dd, *J* = 14.4, 7.5 Hz, 1H), 1.49 – 1.35 (m, 1H), 1.27 – 1.12 (m, 2H), 0.93 – 0.87 (m, 1H), 0.77 – 0.67 (m, 1H) ppm.

^{13}C NMR (101 MHz, $CDCl_3$): δ_C 175.6, 78.1, 67.4, 51.7, 39.1, 31.9, 25.5, 21.3, 15.4, 15.0 ppm.

IR (film) ν_{max}: 2951, 2870, 1721, 1436, 1347, 1193, 1150, 1063, 883 cm^{-1}.

HRMS (ESI^+): calcd. for $C_{10}H_{16}NaO_3$ $[M+Na]^+$ 207.0992, found 207.0994.

4,4,5,5-Tetramethyl-2-(1-(2-methylheptyl)cyclopropyl)-1,3,2-dioxaborolane (**25a**)

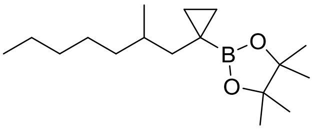

Prepared following General Procedure A using 2-methylheptanoic acid (43 mg, 0.30 mmol, 1.0 equiv.), 4CzIPN (4.7 mg, 0.0060 mmol, 1.0 mol%), Cs_2CO_3 (195 mg, 0.600 mmol, 2.00 equiv.), alkenyl boronic ester **1a** (96 µL, 0.45 mmol, 1.5 equiv.) and DMF (6.0 mL), which was irradiated with 1 × Kessil lamp for 24 h. Purification by flash column chromatography (2% Et_2O/pentane) gave the title compound (52 mg, 0.19 mmol, 62%) as a colourless oil.

TLC: R_f = 0.31 (2% Et_2O/pentane, $KMnO_4$ stain).

^{1}H NMR (400 MHz, $CDCl_3$): δ_H 1.67 – 1.57 (m, 1H), 1.36 – 1.19 (m, 8H), 1.19 (s, 12H), 1.07 – 0.98 (m, 2H), 0.88 (t, *J* = 7.0 Hz, 3H), 0.86 (d, *J* = 6.7 Hz, 3H), 0.69 – 0.60 (m, 2H), 0.31 – 0.23 (m, 2H) ppm.

^{13}C NMR (101 MHz, $CDCl_3$): δ_C 82.9, 44.2, 37.4, 33.5, 32.4, 27.0, 24.74, 24.68, 22.9, 20.1, 14.3, 12.4, 11.3 ppm. The carbon directly attached to boron was not detected due to the boron quadrupole.

^{11}B NMR (128 MHz, $CDCl_3$): δ_B 33.4 (br. s, 1B) ppm.

IR (film) ν_{max}: 2923, 2852, 1457, 1415, 1144, 855 cm^{-1}.

HRMS (ESI$^+$) calcd. for $C_{17}H_{33}BNaO_2$ [M+Na]$^+$ 303.2469, found 303.2454.

Methyl 1-(2-methylheptyl)cyclopropane-1-carboxylate (**25b**)

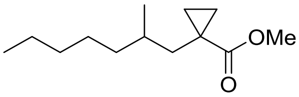

Prepared following General Procedure C using 2-methylheptanoic acid (29 mg, 0.20 mmol, 1.0 equiv.), 4CzIPN (7.9 mg, 0.010 mmol, 5.0 mol%), Cs_2CO_3 (130 mg, 0.400 mmol, 2.00 equiv.), methyl 4-chloro-2-methylenebutanoate (**1b**) (36 mg, 0.24 mmol, 1.2 equiv.) and DMF (4.0 mL), which was irradiated with 1 × Kessil lamp for 20 h. Purification by flash column chromatography (5% EtOAc/pentane) gave the title compound (32 mg, 0.15 mmol, 76%) as a colourless oil.

TLC: R_f = 0.44 (5% EtOAc/pentane, $KMnO_4$ stain).

^{1}H NMR (400 MHz, $CDCl_3$): δ_H 3.63 (s, 3H), 1.80 – 1.68 (m, 2H), 1.34 – 1.19 (m, 9H), 1.18 – 1.11 (m, 1H), 1.10 – 1.02 (m, 1H), 0.91 – 0.84 (m, 6H), 0.73 – 0.67 (m, 1H), 0.66 – 0.60 (m, 1H) ppm.

^{13}C NMR (101 MHz, $CDCl_3$): δ_C 175.9, 51.6, 41.1, 37.3, 32.1, 32.0, 26.6, 22.7, 22.1, 19.8, 16.0, 15.3, 14.1 ppm.

IR (film) ν_{max}: 2954, 2926, 2857, 1728, 1457, 1437, 1343, 1195, 1157, 1029 cm^{-1}.

HRMS (ESI$^+$): calcd. for $C_{13}H_{24}NaO_2$ $[M+Na]^+$ 235.1669, found 235.1654.

Methyl 1-(((1*S*,2*R*)-2-phenylcyclopropyl)methyl)cyclopropane-1-carboxylate (**26b**)

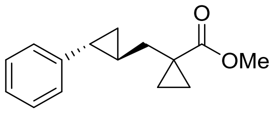

Prepared following General Procedure C using *trans*-1-carboxy-2-phenylcyclopropane (32 mg, 0.20 mmol, 1.0 equiv.), 4CzIPN (7.9 mg, 0.010 mmol, 5.0 mol%), Cs_2CO_3 (130 mg, 0.400 mmol, 2.00 equiv.), methyl 4-chloro-2-methylenebutanoate (**1b**) (36 mg, 0.24 mmol, 1.2 equiv.) and DMF (4.0 mL), which was irradiated with 1 × Kessil lamp for 20 h. Purification by flash column chromatography (10% EtOAc/hexane) gave the title compound (9.2 mg, 0.040 mmol, 20%) as a colourless oil. The d.r. was determined to be >95:5 by ^{1}H NMR.

TLC: R_f = 0.37 (10% EtOAc/hexane, $KMnO_4$ stain).

^{1}H NMR (400 MHz, $CDCl_3$): δ_H 7.28 – 7.19 (m, 2H), 7.18 – 7.08 (m, 1H), 7.06 – 6.99 (m, 2H), 3.65 (s, 3H), 1.78 – 1.63 (m, 3H), 1.24 – 1.17 (m, 2H), 1.19 – 1.08 (m, 1H), 0.94 – 0.88 (m, 1H), 0.86 – 0.76 (m, 3H) ppm.

^{13}C NMR (101 MHz, $CDCl_3$): δ_C 175.8, 143.5, 128.2, 125.6, 125.3, 51.7, 37.6, 23.4, 23.1, 21.6, 15.5, 15.0, 14.9 ppm.

IR (film) ν_{max}: 2997, 2952, 1721, 1604, 1497, 1436, 1354, 1192, 1157, 1028, 879 cm^{-1}.

HRMS (ESI$^+$): calcd. for $C_{15}H_{18}NaO_2$ $[M+Na]^+$ 253.1199, found 253.1217.

Methyl 1-phenethylcyclopropane-1-carboxylate (**27b**)

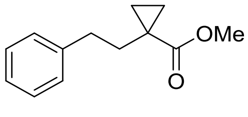

Prepared following General Procedure A using phenylacetic acid (41 mg, 0.30 mmol, 1.0 equiv.), 4CzIPN (2.4 mg, 0.0030 mmol, 1.0 mol%), Cs_2CO_3 (195 mg, 0.600 mmol, 2.00 equiv.), methyl 4-

chloro-2-methylenebutanoate (**1b**) (89 mg, 0.60 mmol, 2.0 equiv.) and DMF (6.0 mL), which was irradiated with 1 × Kessil lamp for 15 h. Purification by flash column chromatography (10% EtOAc/pentane) gave the title compound (37 mg, 0.18 mmol, 60%) as a colourless oil.

TLC: R_f = 0.50 (10% EtOAc/pentane, $KMnO_4$ stain).

^{1}H NMR (400 MHz, $CDCl_3$): δ_H 7.31 – 7.24 (m, 2H), 7.21 – 7.15 (m, 3H), 3.67 (s, 3H), 2.82 – 2.72 (m, 2H), 1.86 – 1.80 (m, 2H), 1.23 – 1.21 (m, 2H), 0.71 – 0.66 (m, 2H) ppm.

^{13}C NMR (101 MHz, $CDCl_3$): δ_C 175.6, 142.3, 128.4, 128.3, 125.7, 51.8, 36.3, 34.1, 23.3, 15.8 ppm.

IR (film) ν_{max}: 3027, 2951, 1720, 1496, 1453, 1354, 1196, 907 cm^{-1}.

HRMS (ESI$^+$): calcd. for $C_{13}H_{16}NaO_2$ $[M+Na]^+$ 227.1043, found 227.1050.

Methyl 1-(3-phenylpropyl)cyclopropane-1-carboxylate (**28b**)

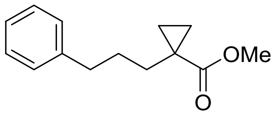

Prepared following General Procedure C using 3-phenylpropanoic acid (45 mg, 0.30 mmol, 1.0 equiv.), 4CzIPN (12 mg, 0.015 mmol, 5.0 mol%), Cs_2CO_3 (195 mg, 0.600 mmol, 2.00 equiv.), methyl 4-chloro-2-methylenebutanoate (**1b**) (54 mg, 0.36 mmol, 1.2 equiv.) and DMF (6.0 mL), which was irradiated with 1 × Kessil lamp for 20 h. Purification by flash column chromatography (10% EtOAc/pentane) gave the title compound (43 mg, 0.20 mmol, 66%) as a colourless oil.

TLC: R_f = 0.50 (10% EtOAc/pentane, $KMnO_4$ stain).

^{1}H NMR (400 MHz, $CDCl_3$): δ_H 7.29 – 7.24 (m, 2H), 7.20 – 7.14 (m, 3H), 3.63 (s, 3H), 2.61 (t, J = 7.8 Hz, 2H), 1.83 – 1.72 (m, 2H), 1.59 – 1.53 (m, 2H), 1.20 – 1.15 (m, 2H), 0.67 – 0.63 (m, 2H) ppm.

^{13}C NMR (101 MHz, $CDCl_3$): δ_C 175.7, 142.3, 128.3, 128.2, 125.6, 51.65, 51.62, 35.9, 33.6, 29.3, 23.4, 15.6 ppm.

IR (film) ν_{max}: 3025, 2948, 2859, 1721, 1496, 1452, 1436, 1353, 1193, 1167, 1029 cm^{-1}.

HRMS (ESI$^+$): calcd. for $C_{14}H_{18}NaO_2$ $[M+Na]^+$ 241.1199, found 241.1189.

2-(1-(((1*S*,4a*S*,10a*S*)-7-Isopropyl-1,4a-dimethyl-1,2,3,4,4a,9,10,10a-octahydrophenanthren-1-yl)methyl)cyclopropyl)-4,4,5,5-tetramethyl-1,3,2-dioxaborolane (**29a**)

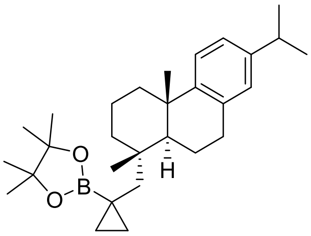

Prepared following General Procedure A with dehydroabietic acid (94 mg, 0.30 mmol, 1.0 equiv.), 4CzIPN (4.7 mg, 0.0060 mmol, 2.0 mol%), Cs_2CO_3 (195 mg, 0.600 mmol, 2.00 equiv.), alkenyl boronic ester **1a** (96 µL, 0.45 mmol, 1.5 equiv.) and DMF (6.0 mL), which was irradiated with 1 × Kessil lamp for 24 h. Purification by preparative TLC (2% EtOAc/pentane) gave the title compound (95 mg, 0.22 mmol, 73%) as a colourless oil. The d.r. was determined to be >95:5 by ^{1}H and ^{13}C NMR.

TLC: R_f = 0.46 (2% EtOAc/pentane, $KMnO_4$ stain).

Optical rotation: $[\alpha]_D^{23}$ +10 (*c* 1.0, $CHCl_3$).

^{1}H NMR (400 MHz, $CDCl_3$): δ_H 7.16 (d, *J* = 8.2 Hz, 1H), 6.98 (dd, *J* = 8.1, 2.0 Hz, 1H), 6.88 (d, *J* = 2.0 Hz, 1H), 2.96 – 2.84 (m, 1H), 2.84 (h, *J* = 7.0 Hz, 1H), 2.32 – 2.22 (m, 1H), 1.86 (ddt, *J* = 13.1, 6.8, 2.4 Hz, 1H), 1.78 – 1.56 (m, 6H), 1.46 (dd, *J* = 12.4, 2.3 Hz, 1H), 1.44 – 1.34 (m, 1H), 1.23 (d, *J* = 6.9 Hz, 6H), 1.21 (s, 3H), 1.22 – 1.19 (m, 1H), 1.17 (s, 6H), 1.16 (s, 6H), 0.97 (s, 3H), 0.69 – 0.57 (m, 2H), 0.39 – 0.28 (m, 2H) ppm.

^{13}C NMR (101 MHz, $CDCl_3$): δ_C 148.2, 145.5, 135.1, 126.9, 124.1, 123.8, 82.9, 50.8, 48.8, 39.4, 38.8, 38.3, 37.8, 33.6, 30.3, 25.7, 24.7, 24.5, 24.2, 24.1, 20.9, 19.2, 19.1, 11.4, 11.2 ppm. The carbon directly attached to boron was not detected due to the boron quadrupole

^{11}B NMR (128 MHz, $CDCl_3$): δ_B 35.1 (br. s, 1B) ppm.

IR (film) ν_{max}: 2958 – 2866, 1413, 1302, 1142 cm^{-1}.

HRMS (ESI^+) calcd. for $C_{29}H_{45}BNaO_2$ $[M+Na]^+$ 459.3410, found 459.3403.

Methyl 1-(((1*S*,4a*S*,10a*S*)-7-isopropyl-1,4a-dimethyl-1,2,3,4,4a,9,10,10a-octahydrophenanthren-1-yl)methyl)cyclopropane-1-carboxylate (**29b**)

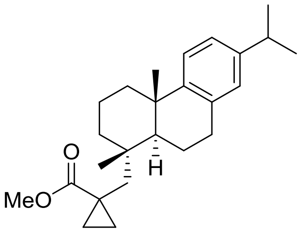

Prepared following General Procedure A using dehydroabietic acid (60 mg, 0.20 mmol, 1.0 equiv.), 4CzIPN (1.6 mg, 0.0020 mmol, 1.0 mol%), Cs_2CO_3 (130 mg, 0.400 mmol, 2.00 equiv.), methyl 4-chloro-2-methylenebutanoate (**1b**) (59 mg, 0.40 mmol, 2.0 equiv.) and DMF (4.0 mL), which was irradiated with 1 × Kessil lamp for 20 h. Purification by flash column chromatography (10% EtOAc/hexane) gave the title compound (63 mg, 0.17 mmol, 85%) as a colourless oil. The d.r. was determined to be 78:22 by ^{13}C NMR.

TLC: R_f = 0.50 (EtOAc /hexane, $KMnO_4$ stain).

Optical rotation: $[\alpha]_D^{23}$ +30 (*c* 1.0, $CHCl_3$).

^{1}H NMR (400 MHz, $CDCl_3$): δ_H 7.17 (d, *J* = 8.1 Hz, 1H), 7.00 (d, *J* = 8.2 Hz, 1H), 6.91 (s, 1H), 3.65 (minor, s, 0.66H), 3.64 (major, s, 2.34H), 3.00 – 2.78 (m, 3H), 2.28 (d, *J* = 12.5 Hz, 1H), 2.16 (d, *J* = 14.7 Hz, 1H), 1.97 – 1.88 (m, 1H), 1.79 – 1.56 (m, 4H), 1.46 – 1.36 (m, 3H), 1.24 (d, *J* = 6.9 Hz, 6H), 1.22 (s, 3H), 1.14 – 1.05 (m, 1H), 0.98 (s, 3H), 0.92 – 0.82 (m, 2H), 0.82 – 0.66 (m, 2H) ppm.

^{13}C NMR (126 MHz, DMSO-d_6, 100 °C): δ_C Mixture of diastereomers: 174.82 (minor), 174.79 (major), 147.6, 144.9, 134.2, 126.3, 123.6, 123.5, 56.3 (minor), 53.7 (minor), 51.3 (major), 49.9 (major), 47.00 (minor), 46.9 (major), 43.3 (minor), 38.9 (minor), 38.71 (major), 38.3 (major), 38.2 (minor), 38.0 (major), 37.4 (major), 36.2 (minor), 35.3 (minor), 32.8 (major), 32.3 (minor), 29.6 (major), 25.3 (major), 24.6 (minor), 23.7 (major), 21.4 (minor), 21.1 (major), 20.5 (minor), 20.1 (major), 19.6 (minor), 18.8 (major), 18.5 (major), 18.1 (minor), 15.0 (minor), 14.5 (major), 14.4 (minor), 13.7 (major), 13.6 (minor) ppm.

IR (film) ν_{max}: 2969, 2901, 1720, 1660, 1497, 1439, 1382, 1196, 1165, 924, 820 cm^{-1}.

HRMS (ESI^+): calcd. for $C_{25}H_{36}NaO_2$ $[M+Na]^+$ 391.2608, found 391.2608.

2-(1-(5-(2,5-Dimethylphenoxy)-2,2-dimethylpentyl)cyclopropyl)-4,4,5,5-tetramethyl-1,3,2-dioxaborolane (**30a**)

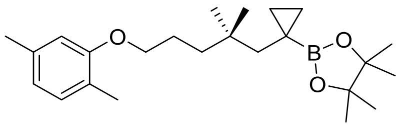

Prepared following General Procedure A using gemfibrozil (50 mg, 0.20 mmol, 1.0 equiv.), 4CzIPN (3.2 mg, 0.0040 mmol, 2.0 mol%), Cs_2CO_3 (130 mg, 0.400 mmol, 2.00 equiv.), alkenyl boronic ester **1a** (65 mg, 0.30 mmol, 1.5 equiv.) and DMF (4.0 mL), which was irradiated with 1 × Kessil lamp for 20 h. Purification by flash column chromatography (10% EtOAc/hexane) gave the title compound (46 mg, 0.12 mmol, 60%) as a colourless oil.

TLC: R_f = 0.30 (10% EtOAc/hexane, $KMnO_4$ stain).

^{1}H NMR (400 MHz, $CDCl_3$): δ_H 6.99 (d, *J* = 7.4 Hz, 1H), 6.64 (d, *J* = 7.5 Hz, 1H), 6.61 (s, 1H), 3.89 (t, *J* = 6.6 Hz, 2H), 2.30 (s, 3H), 2.18 (s, 3H), 1.78 – 1.69 (m, 2H), 1.43 – 1.36 (m, 2H), 1.30 (s, 2H), 1.18 (s, 12H), 0.94 (s, 6H), 0.65 – 0.62 (m, 2H), 0.36 – 0.32 (m, 2H) ppm.

^{13}C NMR (101 MHz, $CDCl_3$): δ_C 157.1, 136.3, 130.2, 123.6, 120.5, 111.9, 82.8, 68.8, 48.5, 39.4, 34.9, 27.7, 24.5, 24.4, 21.4, 15.8, 11.1 ppm. The carbon directly attached to boron was not detected due to the boron quadrupole.

^{11}B NMR (101 MHz, $CDCl_3$): δ_B 33.1 (br. s, 1B) ppm.

IR (film) ν_{max}: 2974, 2955, 1508, 1412, 1370, 1302, 1264, 1144, 1129, 1048, 909, 848 cm^{-1}.

HRMS (ESI$^+$): calcd. for $C_{24}H_{40}BO_3$ $[M+H]^+$ 387.3065, found 387.3083.

Methyl 1-(5-(2,5-dimethylphenoxy)-2,2-dimethylpentyl)cyclopropane-1-carboxylate (**30b**)

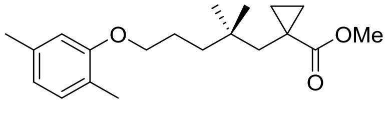

Prepared following General Procedure C using gemfibrozil (75 mg, 0.30 mmol, 1.0 equiv.), 4CzIPN (12 mg, 0.015 mmol, 5.0 mol%), Cs_2CO_3 (195 mg, 0.600 mmol, 2.00 equiv.), 4-chloro-2-methylenebutanamide (**1b**) (54 mg, 0.36 mmol, 1.2 equiv.) and DMF (6.0 mL), which was irradiated with 1 × Kessil lamp for 20 h. Purification by flash column chromatography (10% EtOAc/hexane) gave the title compound (91 mg, 0.29 mmol, 95%) as a colourless oil.

TLC: R_f = 0.45 (10% EtOAc/hexane, $KMnO_4$ stain).

^{1}**H NMR** (400 MHz, $CDCl_3$): δ_H 6.99 (d, *J* = 7.4 Hz, 1H), 6.65 (d, *J* = 7.5 Hz, 1H), 6.62 (s, 1H), 3.90 (t, *J* = 6.5 Hz, 2H), 3.63 (s, 3H), 2.30 (s, 3H), 2.18 (s, 3H), 1.79 – 1.71 (m, 2H), 1.67 (s, 2H), 1.41 – 1.36 (m, 2H), 1.19 – 1.15 (m, 2H), 0.93 (s, 6H), 0.74 – 0.70 (m, 2H) ppm.

^{13}**C NMR** (101 MHz, $CDCl_3$): δ_C 175.7, 157.1, 136.4, 130.2, 123.5, 120.6, 112.0, 68.6, 51.6, 44.9, 39.6, 34.8, 27.6, 24.3, 21.43, 21.38, 15.8, 14.7 ppm.

IR (film) ν_{max}: 2950, 2928, 1724, 1614, 1585, 1508, 1436, 1366, 1263, 1157, 1128, 1048, 802 cm^{-1}.

HRMS (ESI^+): calcd. for $C_{20}H_{30}NaO_3$ $[M+Na]^+$ 341.2087, found 341.2097.

2,5,7,8-Tetramethyl-2-((1-(4,4,5,5-tetramethyl-1,3,2-dioxaborolan-2-yl)cyclopropyl)methyl)chroman-6-ol (**31a**)

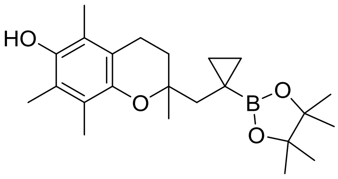

Prepared following General Procedure A using Trolox (50 mg, 0.20 mmol, 1.0 equiv.), 4CzIPN (3.2 mg, 0.0040 mmol, 2.0 mol%), Cs_2CO_3 (130 mg, 0.400 mmol, 2.00 equiv.), alkenyl boronic ester **1a** (65 mg, 0.30 mmol, 1.5 equiv.) and DMF (4.0 mL), which was irradiated with 1 × Kessil lamp for 20 h. Purification by flash column chromatography (10% EtOAc/hexane) gave the title compound (46 mg, 0.12 mmol 60%) as a colourless oil.

TLC: R_f = 0.32 (10% EtOAc/hexane, $KMnO_4$ stain).

^{1}**H NMR** (400 MHz, $CDCl_3$): δ_H 4.19 (s, 1H), 2.65 – 2.52 (m, 2H), 2.16 (s, 3H), 2.12 (s, 3H), 2.11 (s, 3H), 1.88 – 1.78 (m, 2H), 1.53 (d, *J* = 14.4 Hz, 1H), 1.27 (s, 3H), 1.21 (s, 6H), 1.21 (s, 6H), 0.96 – 0.83 (m, 1H), 0.77 – 0.73 (m, 1H), 0.65 – 0.57 (m, 2H), 0.50 – 0.45 (m, 1H) ppm.

^{13}**C NMR** (101 MHz, $CDCl_3$): δ_C 145.6, 144.4, 122.5, 121.0, 118.4, 117.1, 82.9, 75.9, 45.6, 32.0, 24.6, 24.5, 24.0, 20.8, 12.2, 12.0, 11.5, 11.3, 10.8 ppm. The carbon directly attached to boron was not detected due to the boron quadrupole.

^{11}**B NMR** (101 MHz, $CDCl_3$): δ_B 33.2 (br. s, 1B) ppm.

IR (film) ν_{max}: 3447, 2975, 2824, 1449, 1414, 1371, 1305, 1259, 1141, 1084, 854 cm^{-1}.

HRMS (ESI^+): calcd. for $C_{23}H_{35}BNaO_4$ $[M+Na]^+$ 409.2521, found 409.2531.

Methyl 1-((6-hydroxy-2,5,7,8-tetramethylchroman-2-yl)methyl)cyclopropane-1-carboxylate (31b)

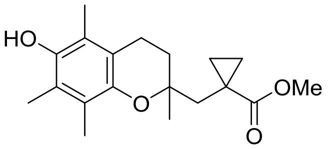

Prepared following General Procedure A using Trolox (75 mg, 0.30 mmol, 1.0 equiv.), 4CzIPN (2.4 mg, 0.0030 mmol, 1.0 mol%), Cs_2CO_3 (195 mg, 0.600 mmol, 2.00 equiv.), methyl 4-chloro-2-methylenebutanoate (**1b**) (89 mg, 0.60 mmol, 2.0 equiv.) and DMF (6.0 mL), which was irradiated with 1 × Kessil lamp for 20 h. Purification by flash column chromatography (20% EtOAc/hexane) gave the title compound (72 mg, 0.23 mmol, 75%) as a colourless oil.

TLC: R_f = 0.31 (20% EtOAc/hexane, $KMnO_4$ stain).

^{1}H NMR (400 MHz, $CDCl_3$): δ_H 4.20 (s, 1H), 3.64 (s, 3H), 2.66 – 2.55 (m, 2H), 2.16 (d, *J* = 15.0 Hz, 1H), 2.15 (s, 3H), 2.10 (s, 3H), 2.08 (s, 3H), 1.97 (d, *J* = 15.2 Hz, 1H), 1.90 – 1.73 (m, 2H), 1.33 – 1.25 (m, 1H), 1.23 (s, 3H), 1.20 – 1.13 (m, 1H), 1.08 – 1.01 (m, 1H), 0.96 – 0.86 (m, 1H) ppm.

^{13}C NMR (101 MHz, $CDCl_3$): δ_C 176.0, 145.2, 144.6, 122.4, 121.1, 118.5, 117.0, 75.5, 51.8, 43.0, 31.9, 23.5, 20.6, 20.4, 15.7, 14.7, 12.2, 12.0, 11.3 ppm.

IR (film) ν_{max}: 3478, 2973, 2951, 1710, 1437, 1420, 1262, 1086, 907 cm^{-1}.

HRMS (ESI^+): calcd. for $C_{19}H_{27}O_4$ $[M+H]^+$ 319.1904, found 319.1903.

Methyl 1-((4*R*)-4-((3*R*,7*R*,8R,9*S*,10*S*,12*S*,13*R*,14*S*,17*R*)-3,7,12-trihydroxy-10,13-dimethylhexadecahydro-1H-cyclopenta[a]phenanthren-17-yl)pentyl)cyclopropane-1-carboxylate (32b)

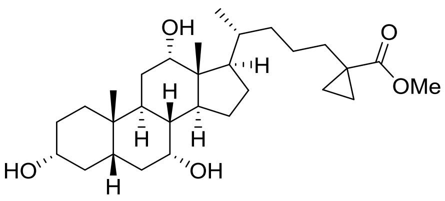

Prepared following General Procedure C using cholic acid (123 mg, 0.30 mmol, 1.0 equiv.), 4CzIPN (12 mg, 0.015 mmol, 5.0 mol%), Cs_2CO_3 (195 mg, 0.600 mmol, 2.00 equiv.), methyl 4-chloro-2-methylenebutanoate (**1b**) (53 mg, 0.36 mmol, 1.2 equiv.) and DMF (6.0 mL), which was irradiated with 1 × Kessil lamp for 20 h. Purification by flash column chromatography (100% EtOAc) gave the title compound (107 mg, 0.224 mmol, 75%) as a colourless oil.

TLC: R_f = 0.28 (100% EtOAc, $KMnO_4$ stain).

Optical rotation: $[\alpha]_D^{23}$ +25.0 (*c* 1.0, $CHCl_3$).

^{1}H NMR (400 MHz, $CDCl_3$): δ_H 4.00 – 3.93 (m, 1H), 3.86 – 3.79 (m, 1H), 3.63 (s, 3H), 3.48 – 3.36 (m, 1H), 2.90 – 2.31 (br. m, 3H), 2.29 – 2.14 (m, 2H), 1.97 – 1.79 (m, 3H), 1.80 – 1.60 (m, 5H), 1.61 – 1.45 (m, 6H), 1.43 – 1.19 (m, 7H), 1.20 – 1.12 (m, 2H), 1.12 – 1.00 (m, 2H), 0.99 – 0.93 (m, 1H), 0.96 (d, *J* = 6.5 Hz, 3H), 0.87 (s, 3H), 0.72 – 0.62 (m, 2H), 0.66 (s, 3H) ppm.

^{13}C NMR (101 MHz, $CDCl_3$): δ_C 175.9, 73.1, 71.9, 68.4, 51.6, 51.6, 47.5, 46.4, 41.7, 41.5, 39.5, 35.9, 35.5, 35.3, 34.7, 34.6, 34.4, 30.5, 28.1, 27.6, 26.4, 24.2, 23.5, 23.2, 22.5, 17.6, 15.7, 15.5, 12.5 ppm.

IR (film) ν_{max}: 3391, 2934, 2865, 1721, 1662, 1436, 1354, 1265, 1196, 1153, 1076, 1044, 912 cm^{-1}.

HRMS (ESI$^+$): calcd. for $C_{29}H_{48}NaO_5$ $[M+Na]^+$ 499.3394, found 499.3385.

Methyl 1-(5-((3a*S*,4*S*,6a*R*)-2-oxohexahydro-1H-thieno[3,4-*d*]imidazol-4-yl)pentyl)cyclopropane-1-carboxylate (**33b**)

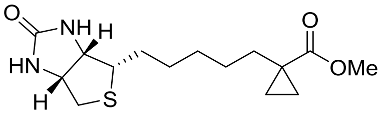

Prepared following General Procedure C using D-(+)-biotin (49 mg, 0.20 mmol, 1.0 equiv.), 4CzIPN (7.9 mg, 0.0100 mmol, 5.0 mol%), Cs_2CO_3 (130 mg, 0.400 mmol, 2.00 equiv.), methyl 4-chloro-2-methylenebutanoate (**1b**) (36 mg, 0.24 mmol, 1.2 equiv.) and DMF (4.0 mL), which was irradiated with 1 × Kessil lamp for 20 h. Purification by flash column chromatography (10% MeOH/CH_2Cl_2) gave the title compound (9.9 mg, 0.032 mmol, 16%) as a colourless oil.

TLC: R_f = 0.59 (10% MeOH/CH_2Cl_2, $KMnO_4$ stain).

Optical rotation: $[\alpha]_D^{23}$ +50.0 (*c* 1.0, $CHCl_3$).

^{1}H NMR (400 MHz, $CDCl_3$): δ_H 5.18 (s, 1H), 5.06 (s, 1H), 4.55 – 4.46 (m, 1H), 4.35 – 4.27 (m, 1H), 3.64 (s, 3H), 3.15 (td, *J* = 5.9, 7.5 Hz, 1H), 2.92 (dd, *J* = 12.8, 5.0 Hz, 1H), 2.73 (d, *J* = 12.8 Hz, 1H), 1.72 – 1.60 (m, 2H), 1.55 – 1.37 (m, 6H), 1.36 – 1.28 (m, 2H), 1.20 – 1.16 (m, 2H), 0.69 – 0.64 (m, 2H) ppm.

^{13}C NMR (101 MHz, $CDCl_3$): δ_C 175.9, 163.1, 61.9, 60.1, 55.5, 51.7, 40.6, 33.9, 29.6, 28.9, 28.6, 27.4, 23.5, 15.6, 15.5 ppm.

IR (film) ν_{max}: 3245, 2923, 2854, 1703, 1453, 1436, 1353, 1260, 1195, 1160, 1027, 908 cm^{-1}.

HRMS (ESI$^+$): calcd. for $C_{15}H_{24}N_2NaO_3S$ $[M+Na]^+$ 335.1400, found 335.1412.

6. Scale-up Reaction

The synthesis of cyclopropane **4b** was carried out on a 2.0 mmol scale.

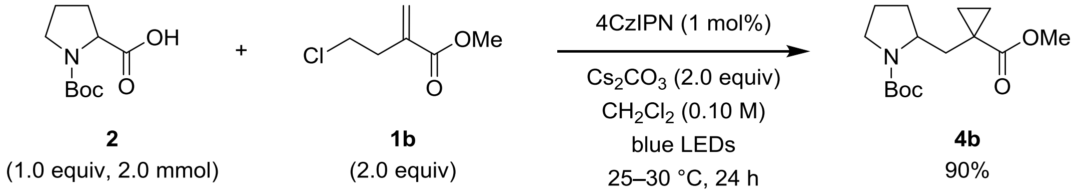

To a 50 mL Schlenk tube (3.5 mL diameter) equipped with a magnetic stir bar was added the Boc-Pro-OH (430 mg, 2.00 mmol, 1.00 equiv.), 4CzIPN (16 mg, 0.020 mmol, 1.0 mol%) and Cs_2CO_3 (1.29 g, 4.00 mmol, 2.00 equiv. Anhydrous CH_2Cl_2 (20 mL, 0.1 M) was then added followed by methyl 4-chloro-2-methylenebutanoate (**1b**) (594 mg, 4.00 mmol, 2.00 equiv.). The vial was sealed with a septum and the reaction mixture degassed by sparging with nitrogen for 10 min. The nitrogen inlet was removed, and the vial further sealed with parafilm. The reaction mixture was stirred at 800 rpm and irradiated with a 40 W Kessil LED lamp with fan cooling for 24 h. The organic phase was washed with H_2O (40 mL), brine (40 mL), dried ($MgSO_4$), filtered, and concentrated *in vacuo*. Purification by flash column chromatography (20% EtOAc/pentane) gave the title compound (510 mg, 1.80 mmol, 90%) as a colourless oil.

7. Studies into the Formation of Larger Rings

7.1. Substrate Synthesis

General Procedure D (for the synthesis of chloroalkyl alkenes **34a**–**36a**):

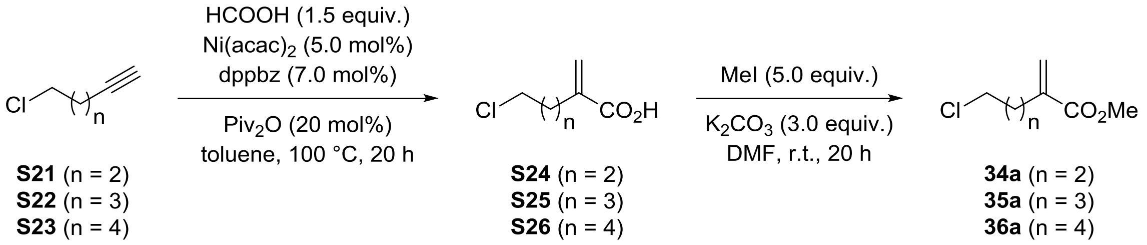

Alkenyl carboxylic acids **S24**–**S26** were prepared following a modified literature procedure:[6] A Schlenk tube containing a magnetic stir bar was charged with Ni(acac)$_2$ (5.0 mol%) and 1,2-bis(diphenylphosphino)benzene (7.0 mol%). The tube was evacuated and back-filled with N_2 three times. Toluene (0.3 M) was added, followed by chloroalkyl alkyne (**S21**, **S22** or **S23**) (1.0 equiv.), formic acid (1.5 equiv.) and pivalic anhydride (20 mol%). The reaction mixture was heated to 100 °C for 24 h before cooling to r.t. and concentrating *in vacuo*. The residue was purified by flash column chromatography (50% EtOAc/hexane) to give the corresponding alkenyl carboxylic acid.

Methyl esters **34a**–**36a** were prepared following a modified literature procedure:[15] To a solution of alkenyl carboxylic acid (**S24**, **S25** or **S26**) (1.0 equiv.) in DMF (0.5 M) at r.t. was added K_2CO_3 (3.0 equiv.). The mixture was cooled to 0 °C before MeI (5.0 equiv.) was added. The resulting mixture was vigorously stirred at r.t. overnight. H_2O (30 mL) was added and the mixture was extracted into CH_2Cl_2 (3 × 30 mL). The combined organic layers were washed with H_2O (3 × 50 mL), saturated aqueous $NaHCO_3$ (30 mL), brine (30 mL), dried ($MgSO_4$), filtered, and concentrated *in vacuo*. The residue was purified by flash column chromatography (10% EtOAc/hexane) to give the corresponding methyl ester.

General Procedure E (for the synthesis of bromoalkyl alkenes **34b**–**36b**):

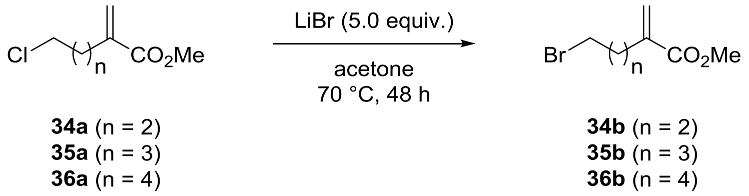

Alkyl bromides **34b**–**36b** were prepared following a modified literature procedure:[16] A solution of alkyl chloride (**34a**, **35a** or **36a**) (1.0 equiv.) and LiBr (5.0 equiv.) in acetone (0.5 M) was heated to reflux for 48 h. After cooling to r.t., H_2O (20 mL) was added and the mixture extracted into CH_2Cl_2 (3 × 40 mL).

The combined organic layers were washed with brine (30 mL), dried ($MgSO_4$), filtered, and concentrated *in vacuo*. The residue was purified by flash column chromatography (10% EtOAc/hexane) to give the corresponding alkyl bromide.

General Procedure F (for the synthesis of iodoalkyl alkenes **34c**–**36c**):

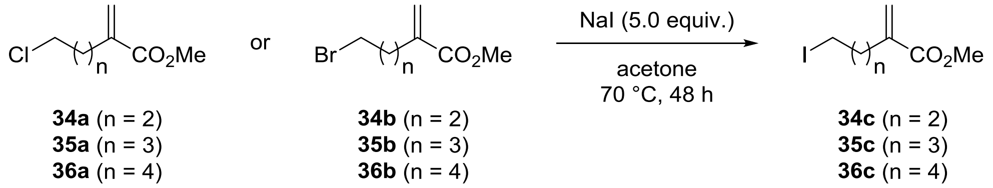

Alkyl iodides **34c**–**36c** were prepared following a modified literature procedure:[17] A solution of alkyl chloride (**34a**, **35a** or **36a**) or alkyl bromide (**34b**, **35b** or **36b**) (1.0 equiv.) and NaI (5.0 equiv.) in acetone (0.5 M) was heated to reflux for 48 h. After cooling to r.t., H_2O (20 mL) was added and the mixture extracted into CH_2Cl_2 (3 × 40 mL). The combined organic layers were washed with brine (30 mL), dried ($MgSO_4$), filtered, and concentrated *in vacuo*. The residue was purified by flash column chromatography (10% EtOAc/hexane) to give the corresponding alkyl iodide.

Methyl 5-chloro-2-methylenepentanoate (**34a**)

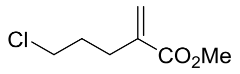

Prepared following General Procedure D using 5-chloropent-1-yne (**S21**) (1.00 g, 10.0 mmol). Purification by flash column chromatography (10% EtOAc/hexane) gave the title compound (1.04 g, 6.40 mmol, 64% over 2 steps) as a colourless oil.

TLC: R_f = 0.50 (10% EtOAc/hexane, $KMnO_4$ stain).

^{1}H NMR (400 MHz, $CDCl_3$): δ_H 6.20 (s, 1H), 5.61 (s, 1H), 3.75 (s, 3H), 3.54 (t, J = 6.4 Hz, 2H), 2.47 (t, J = 7.5 Hz, 2H), 2.04 – 1.90 (m, 2H) ppm.

^{13}C NMR (101 MHz, $CDCl_3$): δ_C 167.3, 138.9, 126.0, 51.9, 44.2, 31.1, 29.2 ppm.

IR (film) ν_{max}: 2953, 1717, 1631, 1438, 1264, 1198, 1167, 996, 946, 816 cm^{-1}.

HRMS (ESI^+) calcd. for $C_7H_{11}ClNaO_2$ $[M+Na]^+$ 185.0340, found 185.0337.

Methyl 5-bromo-2-methylenepentanoate (**34b**)

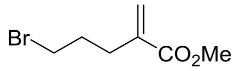

Prepared following General Procedure E using methyl 5-chloro-2-methylenepentanoate (**34a**) (650 mg, 4.00 mmol). Purification by flash column chromatography (10% EtOAc/hexane) gave the title compound (0.75 g, 3.6 mmol, 90%) as a colourless oil.

TLC: R_f = 0.50 (10% EtOAc/hexane, $KMnO_4$ stain).

^{1}H NMR (400 MHz, $CDCl_3$): δ_H 6.20 (s, 1H), 5.62 (s, 1H), 3.76 (s, 3H), 3.41 (t, *J* = 6.6 Hz, 2H), 2.47 (t, *J* = 7.4 Hz, 2H), 2.12 – 1.97 (m, 2H) ppm.

^{13}C NMR (101 MHz, $CDCl_3$): δ_C 167.3, 138.8, 126.1, 51.9, 32.9, 31.2, 30.5 ppm.

IR (film) ν_{max}: 2951, 1717, 1630, 1437, 1246, 1197, 1164, 992, 945, 816 cm^{-1}.

HRMS (ESI^+) calcd. for $C_7H_{11}BrNaO_2$ $[M+Na]^+$ 228.9835, found 228.9838.

Methyl 5-iodo-2-methylenepentanoate (**34c**)

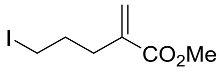

Prepared following General Procedure F using methyl 5-bromo-2-methylenepentanoate (**34b**) (414 mg, 2.00 mmol). Purification by flash column chromatography (10% EtOAc/hexane) gave the title compound (0.46 g, 1.8 mmol, 90%) as a colourless oil.

TLC: R_f = 0.50 (10% EtOAc/hexane, $KMnO_4$ stain).

^{1}H NMR (400 MHz, $CDCl_3$): δ_H 6.19 (s, 1H), 5.62 (s, 1H), 3.75 (s, 3H), 3.18 (t, *J* = 6.7 Hz, 2H), 2.42 (t, *J* = 7.1 Hz, 3H), 2.08 – 1.90 (m, 2H) ppm.

^{13}C NMR (101 MHz, $CDCl_3$): δ_C 167.2, 138.6, 126.1, 51.9, 32.7, 31.9, 5.9 ppm.

IR (film) ν_{max}: 2949, 1717, 1629, 1436, 1264, 1219, 1194, 1167, 944, 816 cm^{-1}.

HRMS (ESI^+) calcd. for $C_7H_{11}INaO_2$ $[M+Na]^+$ 276.9696, found 276.9692.

Methyl 6-chloro-2-methylenehexanoate (35a)

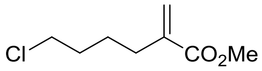

Prepared following General Procedure D using 6-chloropent-1-yne (**S22**) (1.17 g, 10.0 mmol). Purification by flash column chromatography (10% EtOAc/hexane) gave the title compound (1.15 g, 6.50 mmol, 65% over 2 steps) as a colourless oil.

TLC: R_f = 0.50 (10% EtOAc/hexane, $KMnO_4$ stain).

^{1}H NMR (400 MHz, $CDCl_3$): δ_H 6.16 (s, 1H), 5.56 (s, 1H), 3.75 (s, 3H), 3.55 (t, *J* = 6.6 Hz, 2H), 2.33 (t, *J* = 7.6 Hz, 2H), 1.88 – 1.75 (m, 2H), 1.69 – 1.54 (m, 2H) ppm.

^{13}C NMR (101 MHz, $CDCl_3$): δ_C 167.6, 140.0, 125.1, 51.8, 44.7, 32.0, 31.1, 25.6 ppm.

IR (film) ν_{max}: 2951, 1717, 1630, 1437, 1291, 1196, 1165, 91, 945, 817 cm^{-1}.

HRMS (ESI^+) calcd. for $C_8H_{13}ClNaO_2$ $[M+Na]^+$ 199.0496, found 199.0505.

Methyl 6-bromo-2-methylenehexanoate (35b)

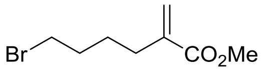

Prepared following General Procedure E using methyl 6-chloro-2-methylenehexanoate (**35a**) (706 mg, 4.00 mmol). Purification by flash column chromatography (10% EtOAc/hexane) gave the title compound (0.80 g, 3.6 mmol, 90%) as a colourless oil.

TLC: R_f = 0.50 (10% EtOAc/hexane, $KMnO_4$ stain).

^{1}H NMR (400 MHz, $CDCl_3$): δ_H 6.14 (s, 1H), 5.54 (s, 1H), 3.74 (s, 3H), 3.40 (t, *J* = 6.7 Hz, 2H), 2.32 (t, *J* = 7.6 Hz, 2H), 1.92 – 1.82 (m, 2H), 1.67 – 1.55 (m, 2H) ppm.

^{13}C NMR (101 MHz, $CDCl_3$): δ_C 167.5, 139.9, 125.0, 51.8, 33.4, 32.2, 31.0, 26.9 ppm.

IR (film) ν_{max}: 2950, 1717, 1630, 1436, 1254, 1195, 1162, 944, 817 cm^{-1}.

HRMS (ESI^+) calcd. for $C_8H_{13}BrNaO_2$ $[M+Na]^+$ 242.9991, found 242.9996.

Methyl 6-iodo-2-methylenehexanoate (**35c**)

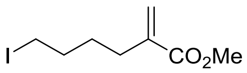

Prepared following General Procedure F using methyl 6-chloro-2-methylenehexanoate (**35a**) (309 mg, 1.75 mmol). Purification by flash column chromatography (10% EtOAc/hexane) gave the title compound (413 mg, 1.54 mmol, 88%) as a yellow oil.

TLC: R_f = 0.50 (10% EtOAc/hexane, $KMnO_4$ stain).

^{1}H NMR (400 MHz, $CDCl_3$): δ_H 6.15 (s, 1H), 5.55 (s, 1H), 3.75 (s, 3H), 3.19 (t, *J* = 7.0 Hz, 2H), 2.32 (t, *J* = 7.6 Hz, 2H), 1.89 – 1.80 (m, 2H), 1.62 – 1.54 (m, 2H) ppm.

^{13}C NMR (101 MHZ, $CDCl_3$): δ_C 167.5, 139.9, 125.1, 51.8, 32.9, 30.8, 29.2, 6.4 ppm.

IR (film) ν_{max}: 2948, 1717, 1630, 1436, 1277, 1193, 1165, 943, 816 cm^{-1}.

HRMS (ESI$^+$) calcd. for $C_8H_{13}INaO_2$ [M+Na]$^+$ 290.9852, found 290.9857.

Methyl 7-bromo-2-methyleneheptanoate (**36b**)

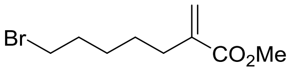

7-Chlorohept-1-yne (**S23**) was reacted according to General Procedure D to generate methyl 7-chloro-2-methyleneheptanoate (**36a**) (763 mg, 4.00 mmol), which was further reacted according to General Procedure E. Purification by flash column chromatography (10% EtOAc/hexane) gave the title compound (0.85 g, 3.6 mol, 90%) as a colourless oil.

TLC: R_f = 0.50 (10% EtOAc/hexane, $KMnO_4$ stain).

^{1}H NMR (400 MHz, $CDCl_3$): δ_H 6.13 (s, 1H), 5.52 (s, 1H), 3.73 (s, 3H), 3.39 (t, *J* = 6.8 Hz, 2H), 2.30 (t, *J* = 6.8 Hz, 2H), 1.92 – 1.82 (m, 2H), 1.53 – 1.40 (m, 4H) ppm.

^{13}C NMR (101 MHz, $CDCl_3$): δ_C 167.6, 140.3, 124.8, 51.7, 33.7, 32.5, 31.7, 27.6, 27.5 ppm.

IR (film) ν_{max}: 2940, 1718, 1630, 1436, 1194, 1161, 994, 944, 816 cm^{-1}.

HRMS (ESI$^+$) calcd. for $C_9H_{15}BrNaO_2$ [M+Na]$^+$ 257.0148, found 257.0154.

Methyl 6-iodo-2-methylenehexanoate (**36c**)

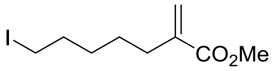

Prepared following General Procedure F using methyl 7-bromo-2-methyleneheptanoate (**36b**) (292 mg, 1.24 mmol). Purification by flash column chromatography (10% EtOAc/hexane) gave the title compound (315 mg, 1.12 mmol, 90%) as a yellow oil.

TLC: R_f = 0.50 (10% EtOAc/hexane, $KMnO_4$ stain).

^{1}H NMR (400 MHz, $CDCl_3$): δ_H 6.13 (s, 1H), 5.53 (s, 1H), 3.74 (s, 3H), 3.18 (t, *J* = 7.0 Hz, 2H), 2.30 (t, *J* = 7.3 Hz, 2H), 1.89 – 1.78 (m, 2H), 1.53 – 1.37 (m, 4H) ppm.

^{13}C NMR (101 MHZ, $CDCl_3$): δ_C 167.6, 140.3, 124.8, 124.8, 51.8, 33.2, 31.7, 30.0, 27.3, 6.9 ppm.

IR (film) ν_{max}: 2932, 1717, 1630, 1436, 1273, 1202, 1163, 943, 816 cm^{-1}.

HRMS (ESI^+) calcd. for $C_9H_{15}INaO_2$ $[M+Na]^+$ 305.0009, found 305.0007.

7.2. Attempted Cyclobutane Synthesis

***tert*-Butyl 2-(5-chloro-2-(methoxycarbonyl)pentyl)pyrrolidine-1-carboxylate (40a)**

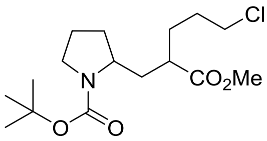

Prepared following General Procedure B using Boc-Pro-OH (43 mg, 0.20 mmol, 1.0 equiv.), 4CzIPN (1.6 mg, 0.0020 mmol, 1.0 mol%), Cs_2CO_3 (130 mg, 0.400 mmol, 2.00 equiv.), methyl 5-chloro-2-methylenepentanoate (**34a**) (65 mg, 0.40 mmol, 2.0 equiv.) and CH_2Cl_2 (4.0 mL), which was irradiated with 1 × Kessil lamp for 15 h. Purification by flash column chromatography (10% EtOAc/hexane) gave the title compound (43 mg, 0.13 mmol, 65%) as a colourless oil. The d.r. could not be determined by ^{1}H NMR but is expected to be approximately 1:1.

TLC: R_f = 0.33 (20% EtOAc/pentane, $KMnO_4$ stain).

^{1}H NMR (400 MHz, DMSO-d_6): Mixture of diastereomers: δ_H 3.77 – 3.51 (m, 3H), 3.60 (s, 3H), 3.27 – 3.19 (m, 1H), 3.19 – 3.09 (m, 1H), 2.45 – 2.31 (m, 1H), 1.99 – 1.50 (m, 9H), 1.43 – 1.34 (m, 1H), 1.38 (s, 9H) ppm.

^{13}C NMR (101 MHz, DMSO-d_6): δ_C 175.8 + 175.6 (diastereomeric peaks), 154.1 + 153.9 (diastereomeric peaks), 78.7, 55.7 + 55.5 (diastereomeric peaks), 51.84 + 51.78 (diastereomeric peaks), 46.4 + 46.1 (diastereomeric peaks), 45.5, 42.1 + 41.7 (diastereomeric peaks), 37.2 + 36.8 + 36.6 (diastereomeric/rotameric peaks), 30.4, 30.1 + 29.5 (diastereomeric peaks), 28.6, 23.7 + 22.8 (diastereomeric peaks) ppm.

IR (film) ν_{max}: 3451, 2970, 1734, 1690, 1392, 1249, 1167, 1105 cm^{-1}.

HRMS (ESI$^+$) calcd. for $C_{16}H_{28}ClNNaO_4$ $[M+Na]^+$ 356.1599, found 356.1614.

***tert*-Butyl 2-(5-bromo-2-(methoxycarbonyl)pentyl)pyrrolidine-1-carboxylate (40b)**

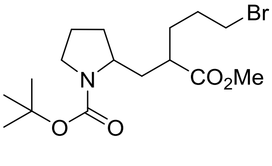

Prepared following General Procedure B using Boc-Pro-OH (43 mg, 0.20 mmol, 1.0 equiv.), 4CzIPN (1.6 mg, 0.0020 mmol, 1.0 mol%), Cs_2CO_3 (130 mg, 0.400 mmol, 2.00 equiv.), methyl 5-bromo-2-methylenepentanoate (**34b**) (83 mg, 0.40 mmol, 2.0 equiv.) and CH_2Cl_2 (4.0 mL), which was irradiated

with 1 × Kessil lamp for 15 h. Purification by flash column chromatography (10% EtOAc/hexane) gave the title compound (47 mg, 0.12 mmol, 62%) as a colourless oil. The d.r. could not be determined by ^{1}H NMR but is expected to be approximately 1:1.

TLC: R_f = 0.33 (20% EtOAc/hexane, $KMnO_4$ stain).

^{1}H NMR (400 MHz, $CDCl_3$): Mixture of diastereomers: δ_H 3.92 – 3.64 (m, 1H), 3.67 (s, 3 H), 3.54 – 3.19 (m, 2H), 3.38 (t, *J* = 6.7 Hz, 2H), 2.51 – 2.34 (m, 1H), 2.14 – 1.52 (m, 9H), 1.51 – 1.34 (m, 1H), 1.45 (s, 9 H) ppm.

^{13}C NMR (101 MHz, $CDCl_3$): δ_C 175.9 + 175.6 (diastereomeric peaks), 154.5, 79.4 + 79.0 (diastereomeric peaks), 55.8 + 55.3 (diastereomeric peaks), 51.6, 46.3 + 46.0 (diastereomeric peaks), 45.9 + 44.5 (diastereomeric peaks), 42.2, 37.4 + 37.1 (diastereomeric peaks), 33.1, 31.6, 30.6 + 30.4 (diastereomeric peaks), 28.5, 23.6 + 23.0 (diastereomeric peaks) ppm.

IR (film) ν_{max}: 3451, 2970, 1734, 1690, 1392, 1365, 1249, 1167, 1105 cm^{-1}.

HRMS (ESI^+) calcd. for $C_{16}H_{28}BrNNaO_4$ $[M+Na]^+$ 400.1094, found 400.1082.

***tert*-Butyl 2-(5-iodo-2-(methoxycarbonyl)pentyl)pyrrolidine-1-carboxylate** (**40c**)

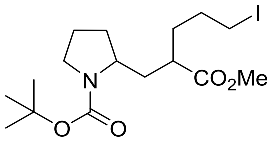

Prepared following General Procedure B using Boc-Pro-OH (43 mg, 0.20 mmol, 1.0 equiv.), 4CzIPN (1.6 mg, 0.0020 mmol, 1.0 mol%), Cs_2CO_3 (130 mg, 0.400 mmol, 2.00 equiv.), methyl 5-iodo-2-methylenepentanoate (**34c**) (102 mg, 0.400 mmol, 2.00 equiv.) and CH_2Cl_2 (4.0 mL), which was irradiated with 1 × Kessil lamp for 15 h. Purification by flash column chromatography (10% EtOAc/hexane) gave the title compound (31 mg, 0.073 mmol, 36%) as a colourless oil. The d.r. could not be determined by ^{1}H NMR but is expected to be approximately 1:1.

TLC: R_f = 0.33 (20% EtOAc/hexane, $KMnO_4$ stain).

^{1}H NMR (400 MHz, $CDCl_3$): Mixture of diastereomers: δ_H 3.92 – 3.60 (m, 1H), 3.67 (s, 3 H), 3.47 – 3.19 (m, 2H), 3.15 (t, *J* = 6.7 Hz, 2H), 2.52 – 2.31 (m, 1H), 2.15 – 1.53 (m, 9H), 1.53 – 1.29 (m, 1H), 1.45 (s, 9H) ppm.

^{13}C NMR (101 MHz, $CDCl_3$): δ_C 175.9 + 175.5 (diastereomeric peaks), 154.8 + 154.5 (diastereomeric peaks), 79.4 + 79.0 (diastereomeric peaks), 55.8 + 55.4 (diastereomeric peaks), 51.6, 46.4, 46.0 + 45.9 (diastereomeric peaks), 42.0, 37.4 + 37.1 (diastereomeric peaks), 33.9 + 33.5 + 33.0

(diastereomeric/rotameric peaks), 31.1 + 30.6 (diastereomeric peaks), 28.5, 23.7 + 23.0 (diastereomeric peaks), 6.0 ppm.

IR (film) ν_{max}: 3448, 2970, 1733, 1690, 1392, 165, 1167, 1105 cm^{-1}.

HRMS (ESI^{+}) calcd. for $C_{16}H_{28}INNaO_4$ [M+Na]$^{+}$ 448.0955, found 448.0933.

7.3. Cyclopentane Synthesis

***tert*-Butyl 2-(6-chloro-2-(methoxycarbonyl)hexyl)pyrrolidine-1-carboxylate (41a)**

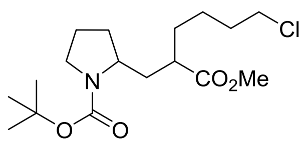

Prepared following General Procedure B using Boc-Pro-OH (43 mg, 0.20 mmol, 1.0 equiv.), 4CzIPN (1.6 mg, 0.0020 mmol, 1.0 mol%), Cs_2CO_3 (130 mg, 0.400 mmol, 2.00 equiv.), methyl 6-chloro-2-methylenehexanoate (**35a**) (71 mg, 0.400 mmol, 2.00 equiv.) and CH_2Cl_2 (4.0 mL), which was irradiated with 1 × Kessil lamp for 15 h. Purification by flash column chromatography (10% EtOAc/hexane) gave the title compound (53 mg, 0.15 mmol, 76%) as a colourless oil. The d.r. could not be determined by ^{1}H NMR but is expected to be approximately 1:1.

TLC: R_f = 0.33 (20% EtOAc/hexane, $KMnO_4$ stain).

^{1}H NMR (400 MHz, $CDCl_3$): Mixture of diastereomers: δ_H 3.93 – 3.61 (m, 1H), 3.66 (s, 3H), 3.51 (t, *J* = 6.7 Hz, 2H), 3.44 – 3.17 (m, 2H), 2.52 – 2.30 (m, 1H), 2.13 – 1.53 (m, 9H), 1.51 – 1.30 (m, 3H), 1.45 (s, 9H) ppm.

^{13}C NMR (101 MHz, $CDCl_3$): δ_C 176.1 + 175.9 (diastereomeric peaks), 154.5, 79.3 + 79.0 (diastereomeric peaks), 55.9 + 55.4 (diastereomeric peaks), 51.5, 46.3 + 45.9 (diastereomeric peaks), 44.7, 42.8 + 42.6 (diastereomeric peaks), 37.3 + 36.8 (diastereomeric peaks), 32.3, 31.7, 30.5, 28.5, 24.7, 23.6 + 23.0 (diastereomeric peaks) ppm.

IR (film) ν_{max}: 3384, 2954, 1733, 1690, 1391, 1365, 1165, 1106 cm^{-1}.

HRMS (ESI$^+$) calcd. for $C_{17}H_{30}ClNNaO_4$ $[M+Na]^+$ 370.1756, found 370.1753.

***tert*-Butyl 2-(6-bromo-2-(methoxycarbonyl)hexyl)pyrrolidine-1-carboxylate (41b)**

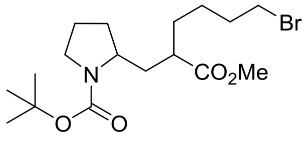

Prepared following General Procedure B using Boc-Pro-OH (43 mg, 0.20 mmol, 1.0 equiv.), 4CzIPN (1.6 mg, 0.0020 mmol, 1.0 mol%), Cs_2CO_3 (130 mg, 0.400 mmol, 2.00 equiv.), methyl 6-bromo-2-methylenehexanoate (**35b**) (88 mg, 0.40 mmol, 2.0 equiv.) and CH_2Cl_2 (4.0 mL), which was irradiated with 1 × Kessil lamp for 15 h. Purification by flash column chromatography (10% EtOAc/hexane) gave

the title compound (20 mg, 0.051 mmol, 25%) as a colourless oil. The d.r. could not be determined by ^{1}H NMR but is expected to be approximately 1:1.

TLC: R_f = 0.33 (20% EtOAc/hexane, $KMnO_4$ stain).

^{1}H NMR (400 MHz, $CDCl_3$): Mixture of diastereomers: δ_H 3.94 – 3.62 (m, 1H), 3.67 (s, 3H), 3.38 (t, *J* = 6.7 Hz, 2H), 3.52 – 3.20 (m, 2H), 2.54 – 2.33 (m, 1H), 2.11 – 1.54 (m, 9H), 1.51 – 1.30 (m, 3H), 1.45 (s, 9H) ppm.

^{13}C NMR (101 MHz, $CDCl_3$): δ_C 176.2 + 175.8 (diastereomeric peaks), 154.5, 79.3 + 79.0 (diastereomeric peaks), 55.9 + 55.4 (diastereomeric peaks), 51.5, 46.3 + 45.9 (diastereomeric peaks), 42.8 + 42.5 (diastereomeric peaks), 37.3 + 36.8 (diastereomeric peaks), 33.4, 32.5, 32.3 + 31.6 (diastereomeric peaks), 30.6, 28.5, 25.9, 23.6 + 23.0 (diastereomeric peaks) ppm.

IR (film) ν_{max}: 3384, 2951, 1733, 1689, 1301, 1365, 1253, 1165, 1105 cm^{-1}.

HRMS (ESI^+) calcd. for $C_{17}H_{30}BrNNaO_4$ $[M+Na]^+$ 414.1250, found 414.1238.

***tert*-Butyl 2-((1-(methoxycarbonyl)cyclopentyl)methyl)pyrrolidine-1-carboxylate (38)**

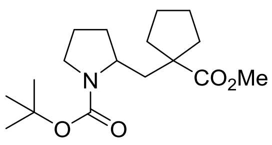

Prepared following General Procedure B using Boc-Pro-OH (43 mg, 0.20 mmol, 1.0 equiv.), 4CzIPN (1.6 mg, 0.0020 mmol, 1.0 mol%), Cs_2CO_3 (130 mg, 0.400 mmol, 2.00 equiv.), methyl 6-iodo-2-methylenehexanoate (**35c**) (107 mg, 0.400 mmol, 2.00 equiv.) and CH_2Cl_2 (4.0 mL), which was irradiated with 1 × Kessil lamp for 15 h. Purification by flash column chromatography (10% EtOAc/hexane) gave the title compound (54 mg, 0.17 mmol, 86%) as a colourless oil.

TLC: R_f = 0.30 (20% EtOAc/hexane, $KMnO_4$ stain).

^{1}H NMR (400 MHz, $CDCl_3$): δ_H 3.89 – 3.71 (m, 1H), 3.65 (s, 3H), 3.46 – 3.17 (m, 2H), 2.29 – 2.16 (m, 1H), 2.13 – 1.87 (m, 2H), 1.85 – 1.71 (m, 4H), 1.69 – 1.50 (m, 6H), 1.49 – 1.36 (m, 1H), 1.45 (s, 9H) ppm.

^{13}C NMR (101 MHz, $CDCl_3$): δ_C 178.2, 154.3, 79.3 + 78.9 (rotameric peaks), 55.0, 52.7, 51.7, 46.0 + 45.7 (rotameric peaks), 43.3, 38.5, 34.0, 30.6 + 30.3 (rotameric peaks), 28.6, 24.7, 24.4, 23.7 + 23.0 (rotameric peaks) ppm.

IR (film) ν_{max}: 3385, 2973, 1729, 1691, 1390, 1365, 1162, 1110 cm^{-1}.

HRMS (ESI^+) calcd. for $C_{17}H_{29}NNaO_4$ $[M+Na]^+$ 334.1989, found 334.1981.

7.4. Attempted Cyclohexane Synthesis

***tert*-Butyl 2-(7-bromo-2-(methoxycarbonyl)heptyl)pyrrolidine-1-carboxylate** (**42b**)

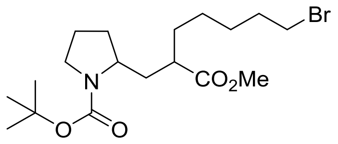

Prepared following General Procedure B using Boc-Pro-OH (43 mg, 0.20 mmol, 1.0 equiv.), 4CzIPN (1.6 mg, 0.0020 mmol, 1.0 mol%), Cs_2CO_3 (130 mg, 0.400 mmol, 2.00 equiv.), methyl 7-bromo-2-methyleneheptanoate (**36b**) (94 mg, 0.40 mmol, 2.0 equiv.) and CH_2Cl_2 (4.0 mL), which was irradiated with 1 × Kessil lamp for 15 h. Purification by flash column chromatography (10% EtOAc/hexane) gave the title compound (74 mg, 0.18 mmol, 91%) as a colourless oil. The d.r. could not be determined by ^{1}H NMR but is expected to be approximately 1:1.

TLC: R_f = 0.33 (20% EtOAc/hexane, $KMnO_4$ stain).

^{1}H NMR (400 MHz, $CDCl_3$): Mixture of diastereomers: δ_H 3.95 – 3.60 (m, 1H), 3.67 (s, 3H), 3.47 – 3.17 (m, 2H), 3.39 (t, *J* = 6.7 Hz, 2H), 2.53 – 2.28 (m, 1H), 2.14 – 1.56 (m, 9H), 1.52 – 1.35 (m, 3H), 1.46 (s, 9H), 1.34 – 1.22 (m, 2H) ppm.

^{13}C NMR (101 MHz, $CDCl_3$): δ_C 176.4 + 176.0 (diastereomeric peaks), 154.6, 79.3 + 79.0 (diastereomeric peaks), 55.9 + 55.4 (diastereomeric peaks), 51.5, 46.4 + 45.9 (diastereomeric peaks), 42.9, 37.4 + 36.8 (diastereomeric peaks), 33.7, 33.0, 32.5, 30.6, 28.5, 28.0, 26.5, 23.6 + 23.0 (diastereomeric peaks) ppm.

IR (film) ν_{max}: 3385, 2934, 1733, 1689, 1390, 1365, 1163, 1104 cm^{-1}.

HRMS (ESI$^+$) calcd. for $C_{18}H_{32}BrNNaO_4$ $[M+Na]^+$ 428.1407, found 428.1396.

***tert*-Butyl 2-(7-iodo-2-(methoxycarbonyl)heptyl)pyrrolidine-1-carboxylate** (**42c**)

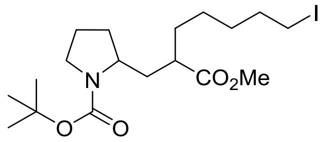

Prepared following General Procedure B using Boc-Pro-OH (43 mg, 0.20 mmol, 1.0 equiv.), 4CzIPN (1.6 mg, 0.0020 mmol, 1.0 mol%), Cs_2CO_3 (130 mg, 0.400 mmol, 2.00 equiv.), methyl 7-iodo-2-methyleneheptanoate (**36c**) (113 mg, 0.400 mmol, 2.00 equiv.) and CH_2Cl_2 (4.0 mL), which was irradiated with 1 × Kessil lamp for 15 h. Purification by flash column chromatography (10%

EtOAc/hexane) gave the title compound (76 mg, 0.17 mmol, 84%) as a colourless oil. The d.r. could not be determined by ^{1}H NMR but is expected to be approximately 1:1.

TLC: R_f = 0.33 (20% EtOAc/hexane, $KMnO_4$ stain).

^{1}H NMR (400 MHz, $CDCl_3$): Mixture of diastereomers: δ_H 3.91 – 3.57 (m, 1H), 3.66 (s, 3H), 3.46 – 3.19 (m, 2H), 3.15 (t, *J* = 7.0 Hz, 2H), 2.50 – 2.26 (m, 1H), 2.12 – 1.73 (m, 6H), 1.67 – 1.54 (m, 3H), 1.44 (s, 9H), 1.42 – 1.21 (m, 5H) ppm.

^{13}C NMR (101 MHz, $CDCl_3$): δ_C 176.3 + 176.0 (diastereomeric peaks), 154.5, 79.3 + 78.9 (diastereomeric peaks), 55.9 + 55.4 (diastereomeric peaks), 51.5 + 51.4 (diastereomeric peaks), 46.3 + 45.8 (diastereomeric peaks), 42.9 + 42.6 (diastereomeric peaks), 37.4 + 36.8 (diastereomeric peaks), 33.2, 32.9 + 32.3 (diastereomeric peaks), 30.5, 30.2, 28.5, 26.3, 23.6 + 22.9 (diastereomeric peaks), 6.9 ppm.

IR (film) ν_{max}: 3385, 2932, 1733, 1689, 1390, 1365, 1163, 1103 cm^{-1}.

HRMS (ESI^+) calcd. for $C_{18}H_{32}INNaO_4$ $[M+Na]^+$ 476.1268, found 476.1251.

8. Mechanistic Studies

8.1. Ring Opening of Pinonic Acid

Methyl (*R*)-1-(3-acetyl-2,2-dimethylhex-5-en-1-yl)cyclopropane-1-carboxylate (**43**)

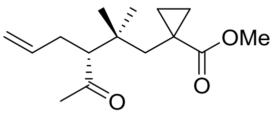

Prepared following General Procedure C using *cis*-pinonic acid (37 mg, 0.20 mmol, 1.0 equiv.), 4CzIPN (7.9 mg, 0.010 mmol, 5.0 mol%), Cs_2CO_3 (130 mg, 0.400 mmol, 2.00 equiv.), methyl 4-chloro-2-methylenebutanoate (**1b**) (36 mg, 0.24 mmol, 1.2 equiv.) and DMF (4.0 mL), which was irradiated with 1 × Kessil lamp for 20 h. Purification by flash column chromatography (10% EtOAc/hexane) gave the title compound (13 mg, 0.051 mmol, 25%) as a colourless oil.

TLC: R_f = 0.33 (10% EtOAc/hexane, $KMnO_4$ stain).

^{1}H NMR (400 MHz, $CDCl_3$): δ_H 5.66 (dddd, *J* = 17.1, 10.1, 7.9, 6.1 Hz, 1H), 5.04 – 4.94 (m, 2H), 3.64 (s, 3H), 2.55 (dd, *J* = 11.5, 2.9 Hz, 1H), 2.39 – 2.29 (m, 1H), 2.26 – 2.19 (m, 1H), 2.12 (s, 3H), 1.81 (d, *J* = 14.7 Hz, 1H), 1.71 (d, *J* = 14.7 Hz, 1H), 1.21 – 1.16 (m, 2H), 0.99 (s, 3H), 0.98 (s, 3H), 0.75 – 0.70 (m, 2H) ppm.

^{13}C NMR (101 MHz, $CDCl_3$): δ_C 213.0, 175.6, 136.3, 116.6, 61.9, 51.7, 42.6, 38.0, 35.0, 32.4, 26.3, 25.0, 21.2, 14.84, 14.80 ppm.

IR (film) ν_{max}: 3079, 2969, 2899, 1724, 1710, 1640, 1437, 1358, 1194, 1167, 1066, 915 cm^{-1}.

HRMS (ESI$^+$): calcd. for $C_{15}H_{24}NaO_3$ [M+Na]$^+$ 275.1618, found 275.1636.

8.2. Reaction of Allyl Acetate Substrate

Methyl 2-(acetoxy(phenyl)methyl)acrylate (**44**)

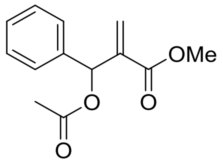

Substrate **44** was prepared following a literature procedure.[18] All recorded spectroscopic data matched those previously reported in the literature.

^{1}H NMR (400 MHz, $CDCl_3$): δ_H 7.41 – 7.28 (m, 5H), 6.71 (s, 1H), 6.42 (s, 1H), 5.88 (s, 1H), 3.72 (s, 3H), 2.12 (s, 3H) ppm.

^{13}C NMR (101 MHz, $CDCl_3$): δ_C 169.3, 165.3, 139.6, 137.7, 128.4, 128.3, 127.6, 125.7, 73.0, 51.9, 21.0 ppm.

***tert*-Butyl (*E*)-2-(2-(methoxycarbonyl)-3-phenylallyl)pyrrolidine-1-carboxylate** (*E*-**45**)

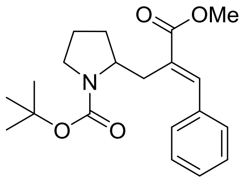

Prepared following a modified General Procedure A using Boc-Pro-OH (43 mg, 0.20 mmol, 1.0 equiv.), 4CzIPN (1.6 mg, 0.0030 mmol, 1.0 mol%), Cs_2CO_3 (130 mg, 0.400 mmol, 2.00 equiv.), allyl acetate **44** (56 mg, 0.24 mmol, 1.2 equiv.) and DMF (4.0 mL), which was irradiated with 1 × Kessil lamp for 15 h. Purification by flash column chromatography (20% EtOAc/ hexane) gave the title compound (63 mg, 0.18 mmol, 91%, *E*/*Z* = 71:29) as a colourless oil.

TLC: R_f = 0.56 (20% EtOAc/hexane, $KMnO_4$ stain).

^{1}H NMR (500 MHz, DMSO-d_6, 100 ºC): δ_H 7.65 (s, 1H), 7.46 – 7.40 (m, 4H), 7.38 – 7.34 (m, 1H), 4.14 – 4.08 (m, 1H), 3.77 (s, 3H), 3.27 – 3.20 (m, 1H), 3.05 – 2.99 (m, 1H), 2.87 (dd, *J* = 13.5, 7.6 Hz, 1H), 2.54 (dd, *J* = 13.5, 6.7 Hz, 1H), 1.82 – 1.74 (m, 1H), 1.73 – 1.60 (m, 2H), 1.55 – 1.49 (m, 1H), 1.38 (s, 9H) ppm.

^{13}C NMR (126 MHz, DMSO-d_6, 100 °C): δ_C 168.0, 153.7, 139.8, 135.4, 130.7, 128.9, 128.5, 128.3, 78.3, 55.8, 51.6, 45.7, 31.7, 30.0, 28.2, 22.5 ppm.

IR (film) ν_{max}: 2975, 2249, 2124, 1717, 1682, 1550, 1394, 820 cm^{-1}.

HRMS (ESI$^+$): calcd. for $C_{20}H_{27}NNaO_4$ [M+Na]$^+$ 368.1832, found 368.1847.

***tert*-Butyl (*Z*)-2-(2-(methoxycarbonyl)-3-phenylallyl)pyrrolidine-1-carboxylate (*Z*-45)**

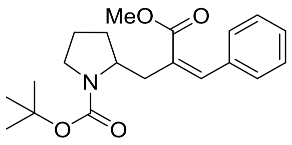

TLC: R_f = 0.50 (20% EtOAc/hexane, $KMnO_4$ stain).

^{1}H NMR (500 MHz, DMSO-d_6, 100 °C): δ_H 7.35 – 7.30 (m, 2H), 7.29 – 7.22 (m, 3H), 6.70 (s, 1H), 3.95 – 3.89 (m, 1H), 3.64 – 3.59 (m, 1H), 3.35 – 3.28 (m, 3H), 3.26 – 3.19 (m, 1H), 2.80 – 2.74 (m, 1H), 2.50 – 2.44 (m, 1H), 1.95 – 1.83 (m, 2H), 1.81 – 1.73 (m, 2H), 1.43 (s, 9H) ppm.

^{13}C NMR (126 MHz, DMSO-d_6, 100 °C): δ_C 169.2, 153.6, 135.8, 134.4, 131.5, 128.1, 127.9, 127.7, 78.3, 56.1, 51.3, 46.1, 29.1, 28.3, 28.2, 22.7 ppm.

IR (film) ν_{max}: 2973, 2250, 2125, 1718, 1684, 1550, 1395, 821 cm^{-1}.

HRMS (ESI$^+$): calcd. for $C_{20}H_{27}NNaO_4$ [M+Na]$^+$ 368.1832, found 368.1838.

8.3. Synthesis and Reactions of Homoallyl Tosylates

3-(4,4,5,5-Tetramethyl-1,3,2-dioxaborolan-2-yl)but-3-en-1-yl 4-methylbenzenesulfonate (46a)

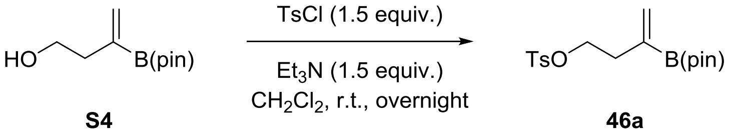

S4 **46a** 70%

Et_3N (468 mg, 4.5 mmol, 1.5 equiv.) was added to 3-(4,4,5,5-tetramethyl-1,3,2-dioxaborolan-2-yl)but-3-en-1-ol (**S4**) (594 mg, 3.00 mmol, 1.00 equiv.) in CH_2Cl_2 (20 mL) at r.t., followed by *p*-toluenesulfonyl chloride (858 mg, 4.5 mmol, 1.5 equiv.). The reaction was stirred overnight, quenched with saturated Na_2CO_3 (10 mL), and extracted into CH_2Cl_2 (3 × 10 mL). The combined organic phases were dried ($MgSO_4$), filtered, and concentrated *in vacuo*. The residue was purified by flash column chromatography (20% EtOAc/hexane) to afford the product **46a** (740 mg, 2.10 mmol, 70%) as a colourless oil.

TLC: R_f = 0.32 (20% EtOAc/hexane, $KMnO_4$ stain).

^{1}H NMR (400 MHz, $CDCl_3$): δ_H 7.77 (d, *J* = 8.2 Hz, 2H), 7.31 (d, *J* = 7.8 Hz, 2H), 5.84 (d, *J* = 3.1 Hz, 1H), 5.63 (d, *J* = 1.9 Hz, 1H), 4.11 (t, *J* = 6.9 Hz, 2H), 2.45 (t, *J* = 6.8 Hz, 2H), 2.42 (s, 3H), 1.19 (s, 12H) ppm.

^{13}C NMR (101 MHz, $CDCl_3$): δ_C 144.4, 133.3, 133.0, 129.7, 127.9, 127.9, 83.6, 69.7, 34.9, 24.7, 21.5 ppm.

^{11}B NMR (101 MHz, $CDCl_3$): δ_B 28.9 (br. s, 1B) ppm.

IR (film) ν_{max}: 3067, 2978, 1620, 1598, 1433, 1361, 1314, 1176, 967 cm^{-1}.

HRMS (ESI$^+$): calcd. for $C_{17}H_{25}BNaO_5S$ $[M+Na]^+$ 375.1408, found 375.1422.

Methyl 2-methylene-4-(tosyloxy)butanoate (46b)

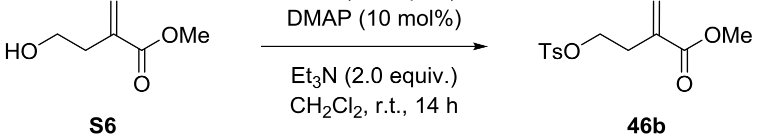

S6 **46b** 70%

To alcohol **S6** (1.3 g, 10 mmol, 1.0 equiv.) in CH_2Cl_2 (40 mL) at r.t. was added triethylamine (2.1 g, 20 mol, 2.0 equiv.), DMAP (0.12 g, 1.0 mol, 10 mol%) and *p*-toluenesulfonyl chloride (2.3 g, 12 mmol, 1.2 equiv.). After 14 h, aqueous 1 M HCl (10 mL) was added and the phases were separated. The aqueous

phase was extracted into Et_2O (3 × 20 mL) and the combined organic phases dried ($MgSO_4$), filtered, and concentrated *in vacuo*. Purification by flash column chromatography (10% EtOAc/hexane) gave sulfonate **46b** (2.0 g, 7.0 mmol, 70%) as a colourless oil.

TLC: R_f = 0.30 (10% EtOAc/hexane, $KMnO_4$ stain).

^{1}H NMR (400 MHz, $CDCl_3$): δ_H 7.76 (d, *J* = 8.3 Hz, 2H), 7.34 (d, *J* = 8.0 Hz, 2H), 6.23 (d, *J* = 1.0 Hz, 1H), 5.64 (dt, *J* = 1.2, 1.0 Hz, 1H), 4.18 (t, *J* = 6.4 Hz, 2H), 3.69 (s, 3H), 2.65 (td, *J* = 6.4, 1.2 Hz, 2H), 2.44 (s, 3H) ppm.

^{13}C NMR (101 MHz, $CDCl_3$): δ_C 166.4, 144.6, 134.7, 132.8, 129.7, 128.4, 127.7, 68.2, 51.7, 31.7, 21.4 ppm.

IR (film) ν_{max}: 2987, 2956, 2901, 1715, 1632, 1597, 1439, 1356, 1174, 1096, 969, 907, 814 cm^{-1}.

HRMS (ESI^+): calcd. for $C_{13}H_{16}NaO_5S$ $[M+Na]^+$ 307.0611, found 307.0614.

Reaction of tosylate 46a with Boc-Pro-OH:

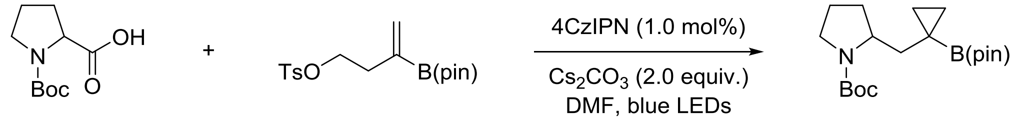

2 **46a** **4a** 84%

Tosylate **46a**: Cyclopropyl boronic ester **4a** was prepared following General Procedure A, using Boc-Pro-OH (65 mg, 0.30 mmol, 1.0 equiv.), 4CzIPN (2.4 mg, 0.0030 mmol, 1.0 mol%), Cs_2CO_3 (195 mg, 0.600 mmol, 2.00 equiv.), alkenyl boronic ester **46a** (159 mg, 0.450 mmol, 1.50 equiv.) and DMF (6.0 mL), which was irradiated with 1 × Kessil lamp for 20 h. Purification by flash column chromatography (10% EtOAc/pentane) gave the title compound (89 mg, 0.25 mmol, 84%) as a colourless oil. See above (Section 4) for spectroscopic and analytical data.

Reaction of tosylate 46b with Boc-Pro-OH:

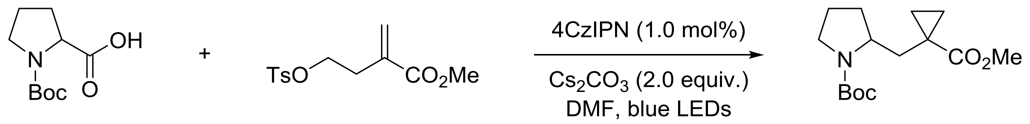

2 **46b** **4b** 94%

Tosylate **46b**: Cyclopropyl carboxylate ester **4b** was prepared following General Procedure A, using Boc-Pro-OH (43 mg, 0.20 mmol, 1.0 equiv.), 4CzIPN (1.6 mg, 0.0020 mmol, 1.0 mol%), Cs_2CO_3 (130

mg, 0.400 mmol, 2.00 equiv.), alkenyl carboxylate ester **46b** (114 mg, 0.400 mmol, 2.00 equiv.) and DMF (4.0 mL), which was irradiated with 1 × Kessil lamp for 20 h. Purification by flash column chromatography (10% EtOAc/pentane) gave the title compound (53 mg, 0.19 mmol, 94%) as a colourless oil. See above (Section 4) for spectroscopic and analytical data.

8.4. Quantum Yield Measurement

The quantum yield was measured for the reaction of Boc-Pro-OH (**2**) with (4-chlorobut-1-en-2-yl)boronic acid pinacol ester (**1a**). The reaction was performed in a quartz cuvette (path length: l = 1.0 cm) positioned 5 cm away from a single 0.1 W blue LED (λ_{max} = 450 nm).

Under the standard conditions, where the carboxylic acid is deprotonated *in situ* using Cs_2CO_3, the reaction mixture is heterogeneous. This will have an impact on light penetration and potentially affect the accuracy of the quantum yield measurements. Therefore, to obtain a more accurate measurement, the reaction was performed using the preformed cesium carboxylate **S27**, which provided a homogeneous reaction mixture. The use of a preformed cesium carboxylate did not affect the efficiency of the reaction, as submitting Boc-Pro-OCs (**S27**) (0.10 mmol) to the standard reaction conditions (General Procedure A, omitting Cs_2CO_3) led to the formation of product **4a** in 97% GC yield after irradiation with a 40 W Kessil lamp for 12 h.

Determination of the Photon Flux:

The photon flux of the LED setup was determined using standard ferrioxalate actinometry.[19]

A 0.15 M ferrioxalate solution was prepared by dissolving 2.21 g of potassium ferrioxalate trihydrate in 30 mL of 0.05 M aq. H_2SO_4. A buffered 5.5 mM phenanthroline solution was prepared by dissolving 50 mg of 1,10-phenanthroline 11.25 g of NaOAc•3H_2O in 50 mL of 0.5 M aq. H_2SO_4. Both solutions were stored in amber bottles in the dark.

Whilst working under red light, 2.0 mL of the 0.15 M ferrioxalate solution was added to a quartz cuvette (l = 1.0 cm). The cuvette was placed 5 cm from a single 0.1 W blue LED and irradiated for specific time internals of between 15 and 60 s. After irradiation, 1.0 mL of the phenanthroline solution was added to the cuvette. The mixture was left to stand for approximately 30 min before the absorbance at λ = 510 nm was measured by UV/Vis spectroscopy. The absorbance of a non-irradiated sample was also measured.

The number of moles of Fe^{2+} formed was calculated using:

$$\text{mol Fe}^{2+} = \frac{V \bullet \Delta A}{l \bullet \varepsilon}$$

Where V is the total volume of the solution after the addition of 1,10-phenanthroline (0.0030 L), ΔA is the difference in absorbance at λ = 510 nm between the irradiated and non-irradiated ferrioxalate solutions, l is the optical path length of the irradiation cell (1.0 cm), and ε is the molar absorptivity of the $Fe(phen)_3^{2+}$ complex at λ = 510 nm (11,100 L mol^{-1} cm^{-1}).

The moles of Fe^{2+} were plotted as a function of time:

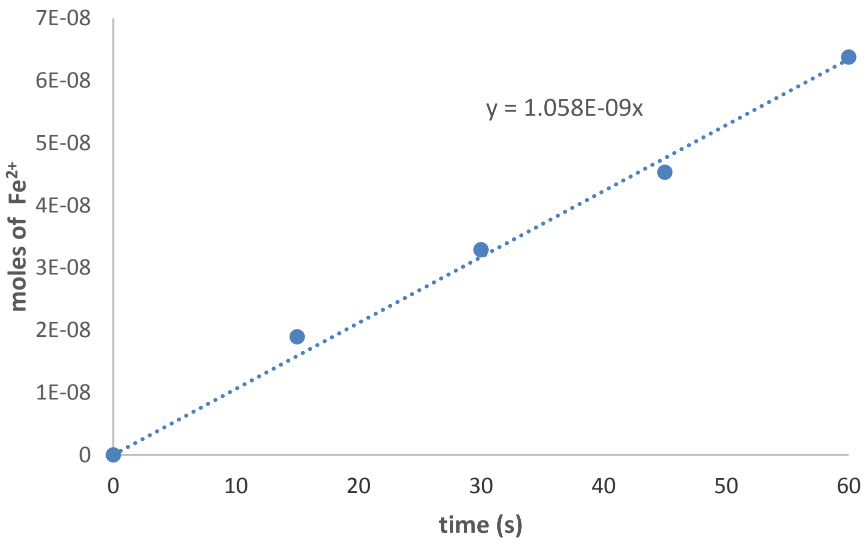

Figure S2. Moles of Fe^{2+} formed vs. time of irradiation for determination of the photon flux.

The photon flux was then calculated using:

$$\text{photon flux} = \frac{\text{mol Fe}^{2+}}{\Phi \bullet \text{t} \bullet \text{f}}$$

Where Φ is the quantum yield of the ferrioxalate actinometer (1.0 at λ = 450 nm),[19] t is the time, and f is the fraction of absorbed light at λ = 450 nm, where $f = 1 - 10^{-A}$. The absorbance (A) of the ferrioxalate solution at λ = 450 nm was measured by UV/Vis spectroscopy to be 1.708, therefore f = 0.9804.

$$\text{photon flux} = \frac{1.06 \times 10^{-9}}{1.00 \bullet 0.980} = 1.08 \times 10^{-9} \text{ einstein s}^{-1}$$

Determination of the Quantum Yield:

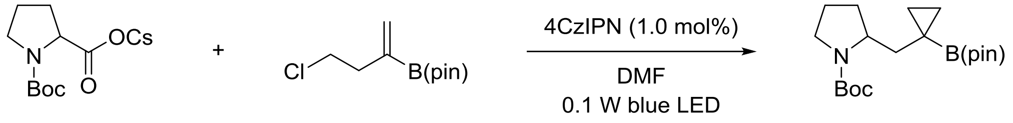

S27 **1a** (1.5 equiv.) **4a** 9.4% (after 4 h) 31.1% (after 12 h)

In a nitrogen-filled glovebox, a 4.5 mL quartz cuvette (path length: l = 1.0 cm) was charged with 4CzIPN (0.8 mg, 0.001 mmol) and Boc-Pro-OCs (**S27**)[20] (35 mg, 0.10 mmol, 1.0 equiv.). The cuvette was sealed with a septum and removed from the glovebox before the sequential addition of DMF (2.0 mL) and

alkenyl boronic ester **1a** (32 mg, 0.15 mmol, 1.5 equiv.). The reaction mixture was degassed by sparging with nitrogen for 10 min. The nitrogen inlet was removed and the vial further sealed with parafilm. The reaction was positioned 5 cm away from a single 0.1 W blue LED, stirred and irradiated for 12 h. The yield was determined by GC analysis, using 1,2,4-trimethoxybenzene as an internal standard, to be 31.1% (3.11×10^{-5} mol).

The quantum yield (Φ) was then calculated using:

$$\Phi = \frac{\text{mol product}}{\text{photon flux} \bullet \text{t} \bullet \text{f}}$$

Where t is the time (43200 s) and f is the fraction of light absorbed by 4CzIPN at λ = 450 nm (for a 5.0×10^{-4} M solution in DMF, this was determined by UV/Vis spectroscopy to be 0.969).

$$\Phi = \frac{3.11 \times 10^{-5}}{1.08 \times 10^{-9} \bullet 43200 \bullet 0.969} = 0.688$$

The reaction was repeated with an irradiation time of 4 h (14400 s) to give 9.4% (9.40×10^{-6} mol) yield.

$$\Phi = \frac{9.40 \times 10^{-6}}{1.08 \times 10^{-9} \bullet 14400 \bullet 0.969} = 0.624$$

Average quantum yield (Φ) of the two experiments = 0.656

9. References

[1] Luo, J.; Zhang, J. *ACS Catal.* **2016**, *6*, 873.

[2] Bunch, L.; Nielsen, B.; Jensen, A. A.; Bräuner-Osborne, H. *J. Med. Chem.* **2006**, *49*, 172.

[3] Takagi, J.; Takahashi, K.; Ishiyama, T.; Miyaura, N. *J. Am. Chem. Soc.* **2002**, *124*, 8001.

[4] Lachia, M.; Iriart, S.; Baalouch, M.; Mesmaeker, A. D.; Beaudegnies, R. *Tetrahedron Lett.* **2011**, *52,* 3219.

[5] Powell, K. J.; Han, L.-C.; Sharma, P.; Moses, J. E. *Org. Lett.* **2014**, *16*, 2158.

[6] Fu, M.-C.; Shang, Rui.; Cheng, W.-M.; Fu, Yao. *ACS Catal.* **2016**, *6*, 2501.

[7] Morita, M.; Drouin, L.; Motoki, R.; Kimura, Y.; Fujimori, I.; Kanai, M.; Shibasaki, M. *J. Am. Chem. Soc.* **2009**, *131,* 3858.

[8] Wang, H.; Lu, Q.; Chiang, C.-W.; Luo, Y.; Zhou, J.; Wang, G.; Lei, A. *Angew. Chem. Int. Ed.* **2017**, *56*, 595.

[9] Ananikov, V. P.; Khemchyan, L. L.; Beletskaya, I. P. *Synlett* **2009**, *2009*, 2375.

[10] Hartog, T. Rudolph, A. Maciá, B. Minnaard, A, J. Feringa, B. L. *J. Am. Chem. Soc.*, **2010**, *132*, 14349.

[11] Jung, H. H.; Kim, K. S.; Kim, N.; Oh, C. H. *Angew. Chem. Int. Ed.* **2003**, *42*, 805.

[12] Vara, B. A.; Struble, T. J.; Wang, W.; Dobish, M. C.; Johnston, J. N. *J. Am. Chem. Soc.* **2015**, *137*, 7302.

[13] Janssen-Müller, D.; Schedler, M.; Fleige, M.; Daniliuc, C. G.; Glorius, F. *Angew. Chem. Int. Ed.* **2015**, *54*, 12492.

[14] Braddock, D. C.; Pouwer, R. H.; Burton, J. W.; Broadwith, P. *J. Org. Chem.* **2009**, *74,* 6042.

[15] Douelle, F.; Capes, A. S.; Greaney, M. F. *Org. Lett.* **2007**, *9*, 1931.

[16] Painter, T. O.; Thornton, P. D.; Orestano, M.; Santini, C.; Organ, M. G.; Aubé, J. *Chem. Eur. J.* **2011**, *17*, 9595.

[17] Snider, B. B.; Hawryluk, N. A. *Org. Lett.* **2001**, *3*, 569.

[18] Novak, A.; Calhorda, M. J.; Costa, P. J.; Woodward, S. *Eur. J. Org. Chem.* **2009**, 898.

[19] a) Montalti, M.; Credi, A.; Prodi, L.; Gandolfi, M. T. *Handbook of Photochemistry*, 3rd ed.; CRC/Taylor & Francis: Boca Raton, FL, 2006; b) Cismesia, M. A.; Yoon, T. P. *Chem. Sci.* **2015**, *6*, 5426.

[20] Noble, A.; Mega, R. S.; Pflästerer, D.; Myers, E. L.; Aggarwal, V. K. *Angew. Chem. Int. Ed.*, **2018**, *57*, 2155.

10. NMR Spectra

^{1}H NMR (400 MHz, $CDCl_3$)

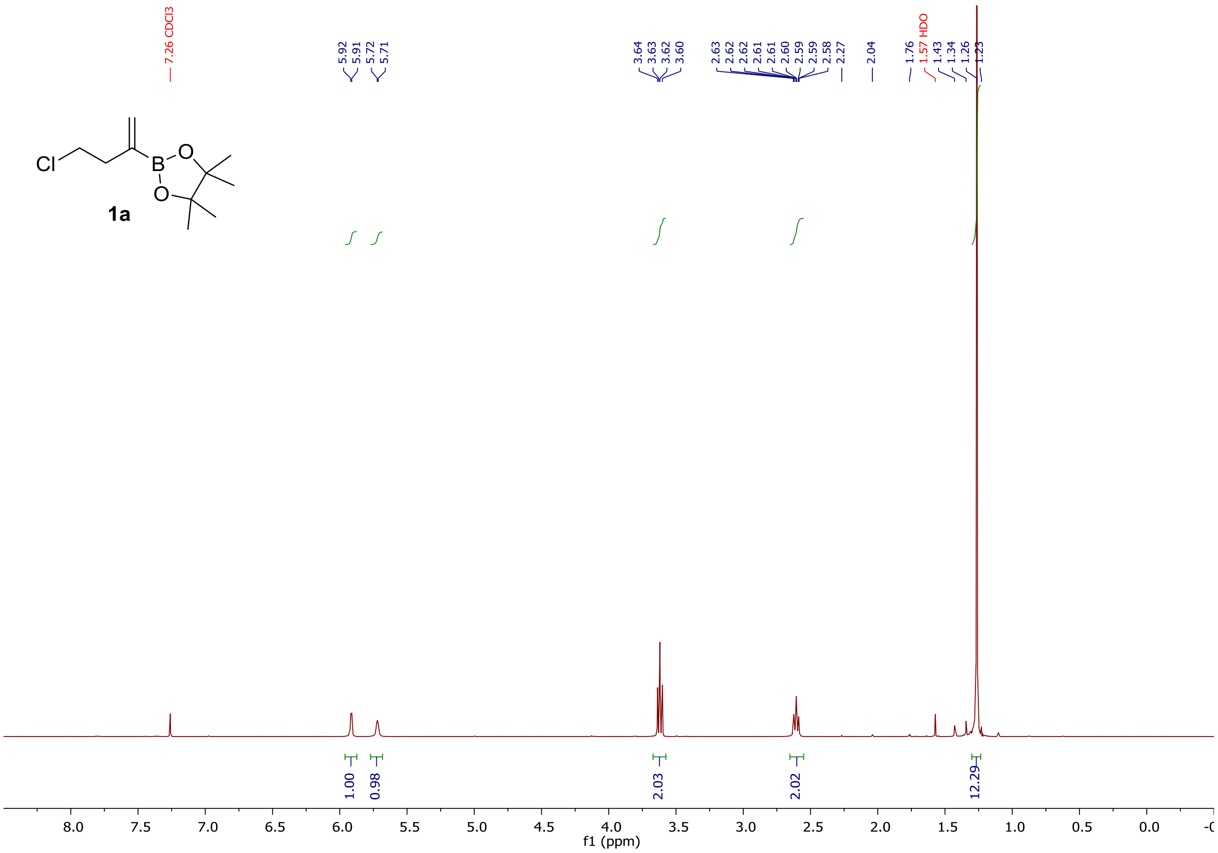

^{13}C NMR (101 MHz, $CDCl_3$)

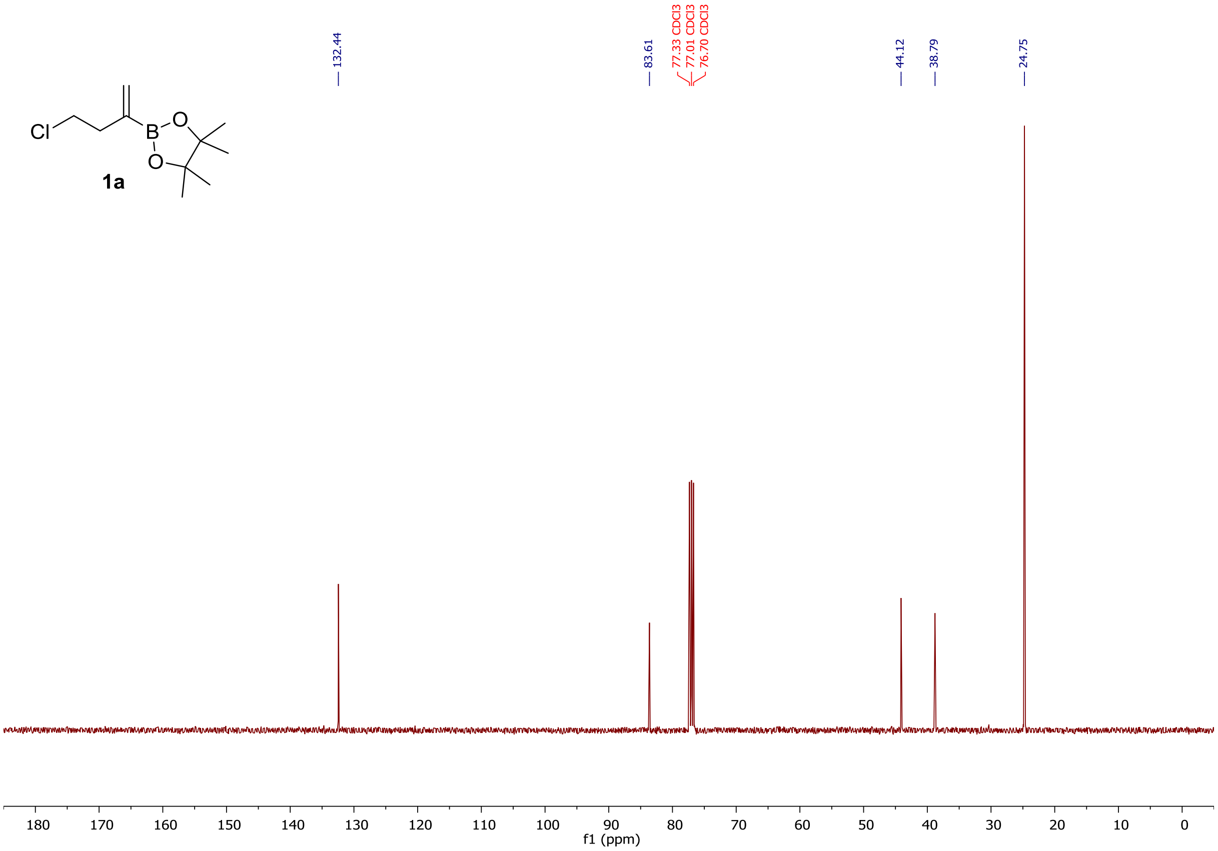

^{1}H NMR (400 MHz, $CDCl_3$)

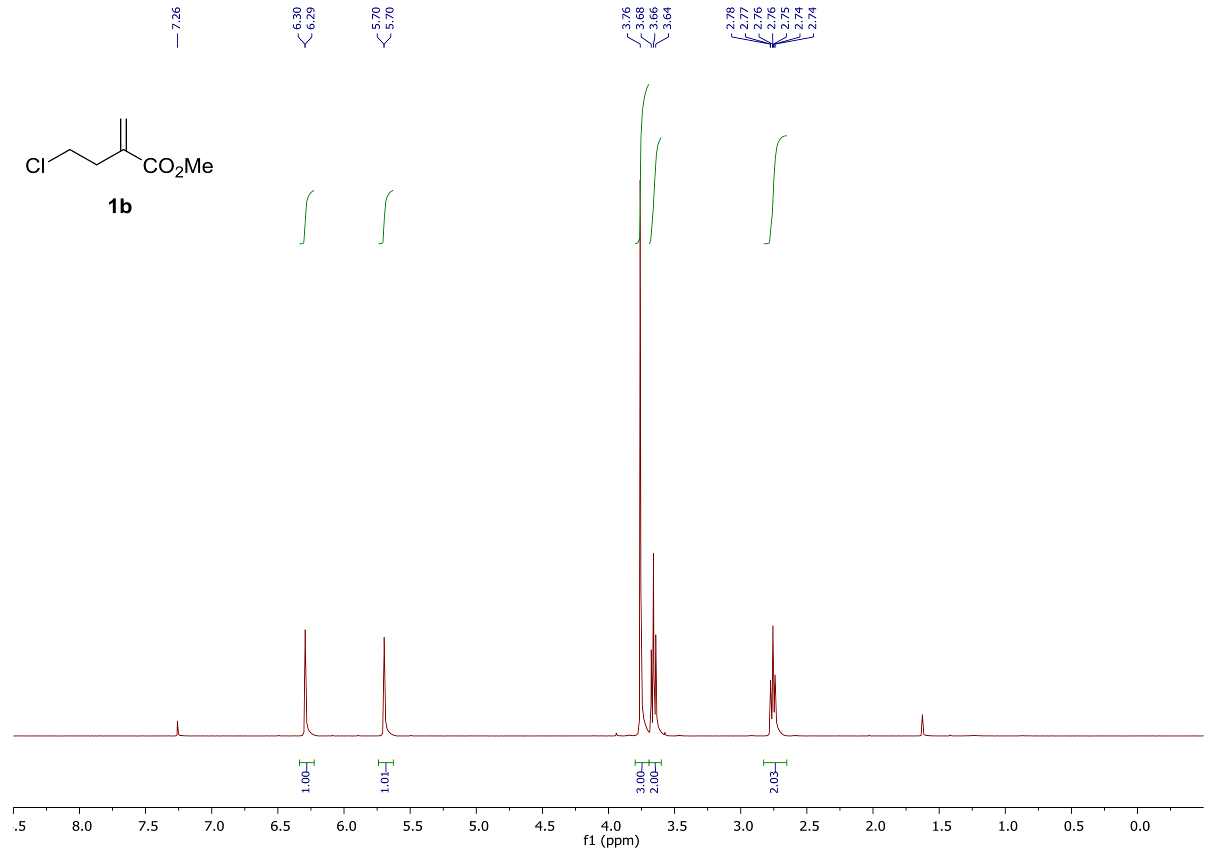

^{13}C NMR (101 MHz, $CDCl_3$)

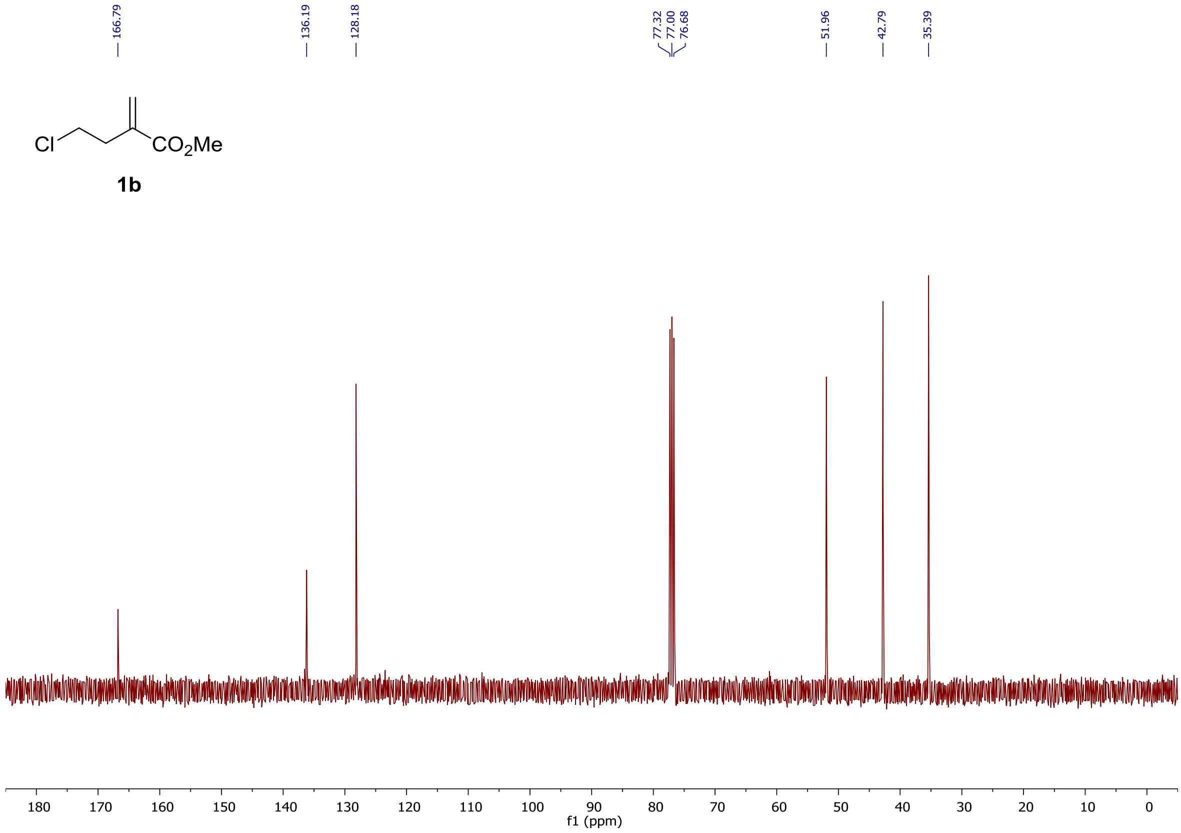

^{1}H NMR (400 MHz, $CDCl_3$)

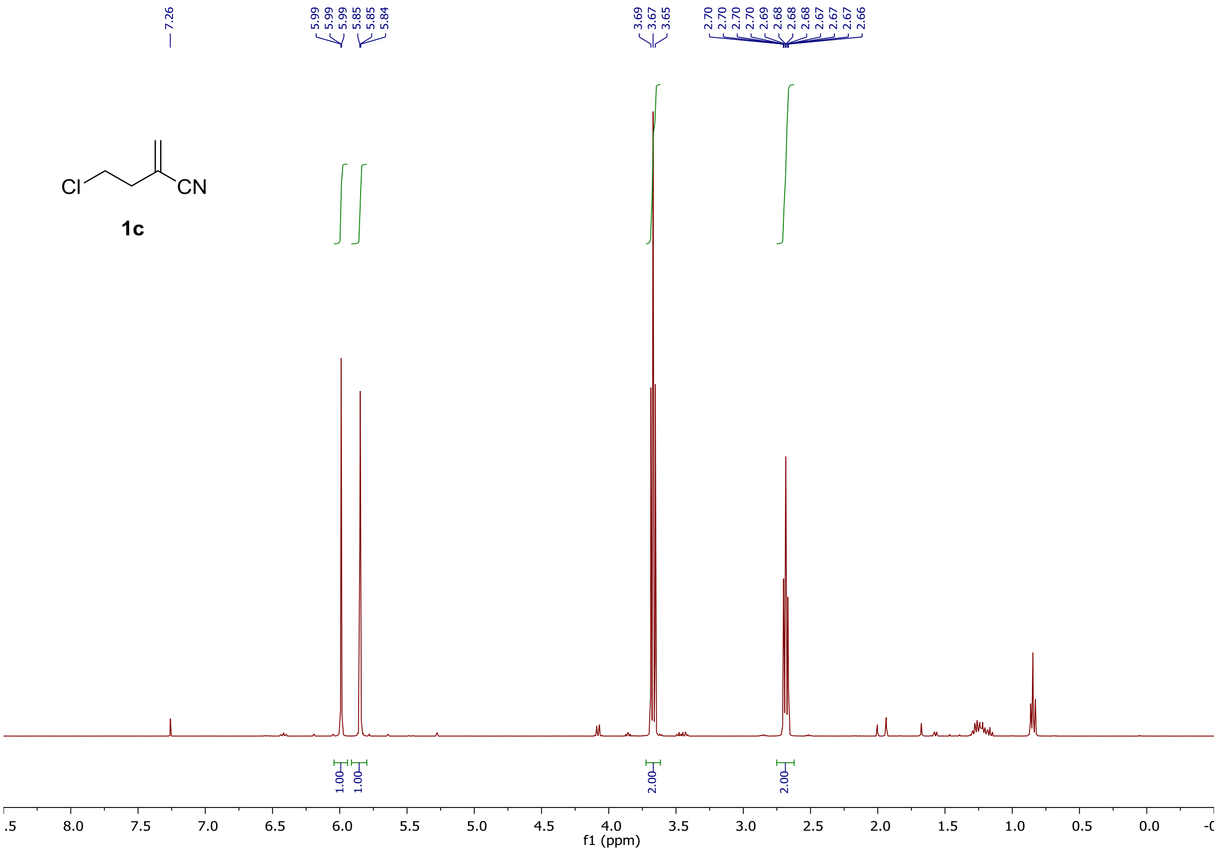

^{13}C NMR (101 MHz, $CDCl_3$)

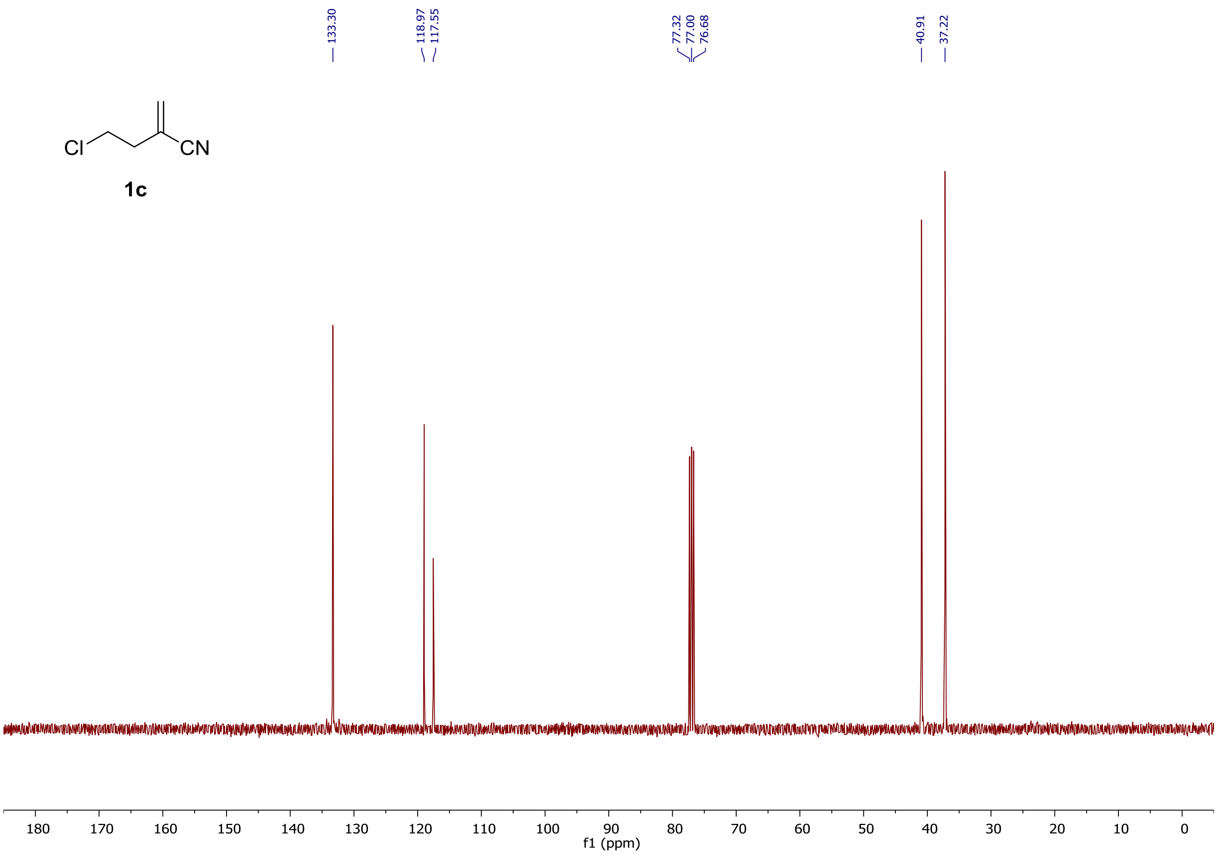

^{1}H NMR (400 MHz, $CDCl_3$)

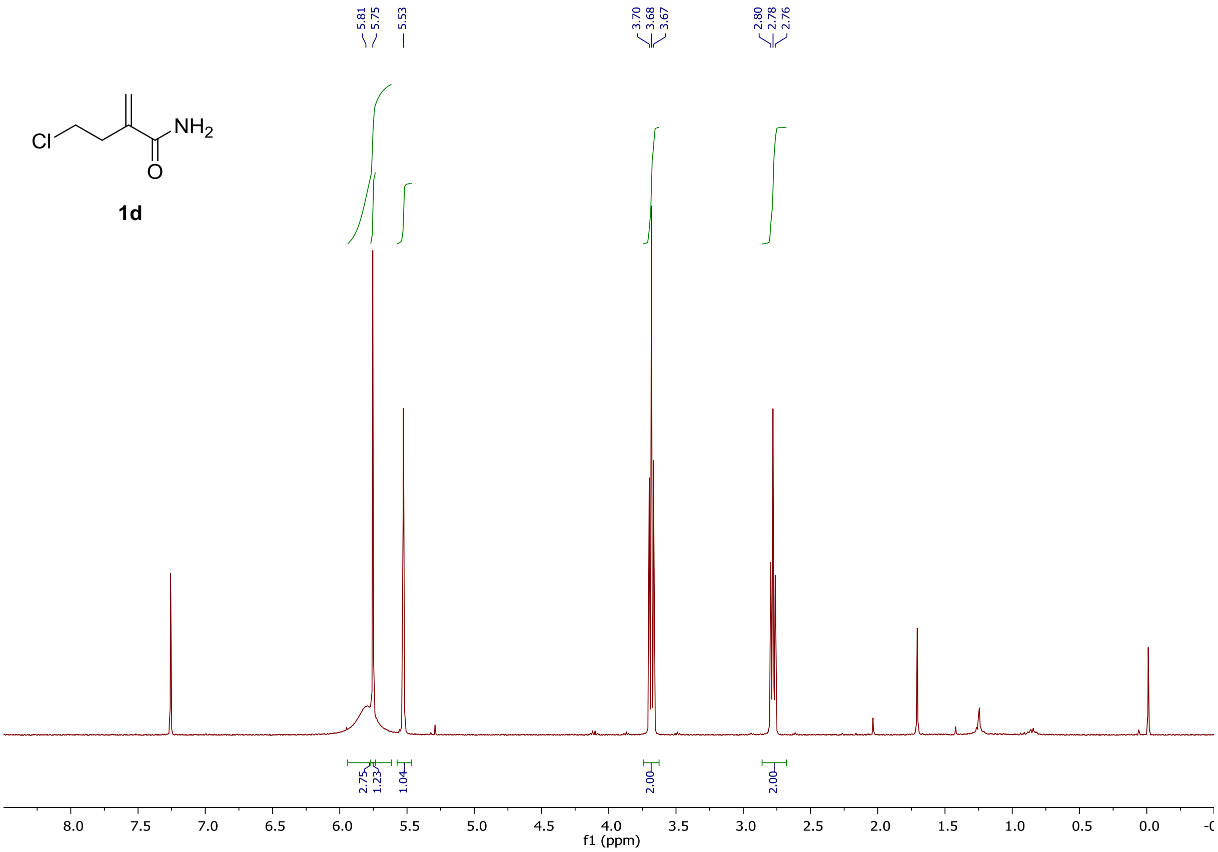

^{13}C NMR (101 MHz, $CDCl_3$)

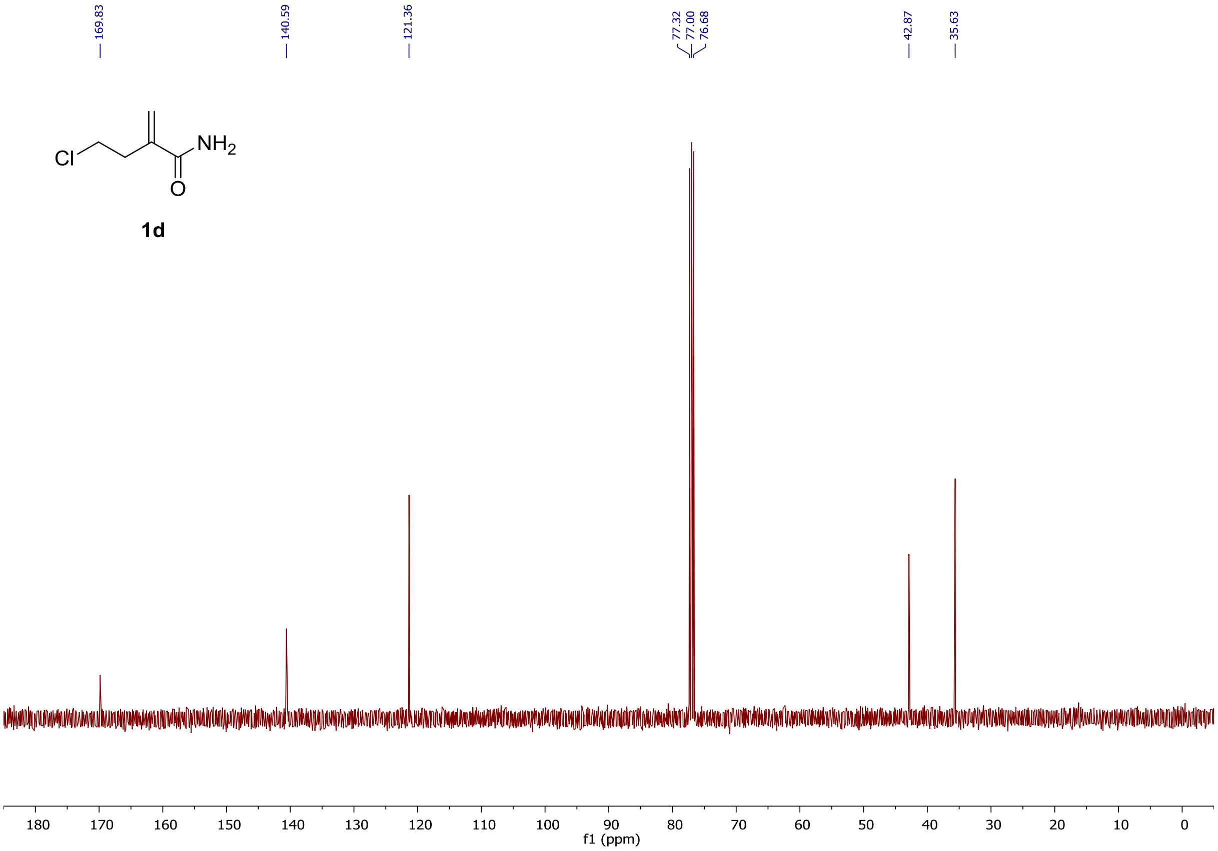

^{1}H NMR (400 MHz, $CDCl_3$)

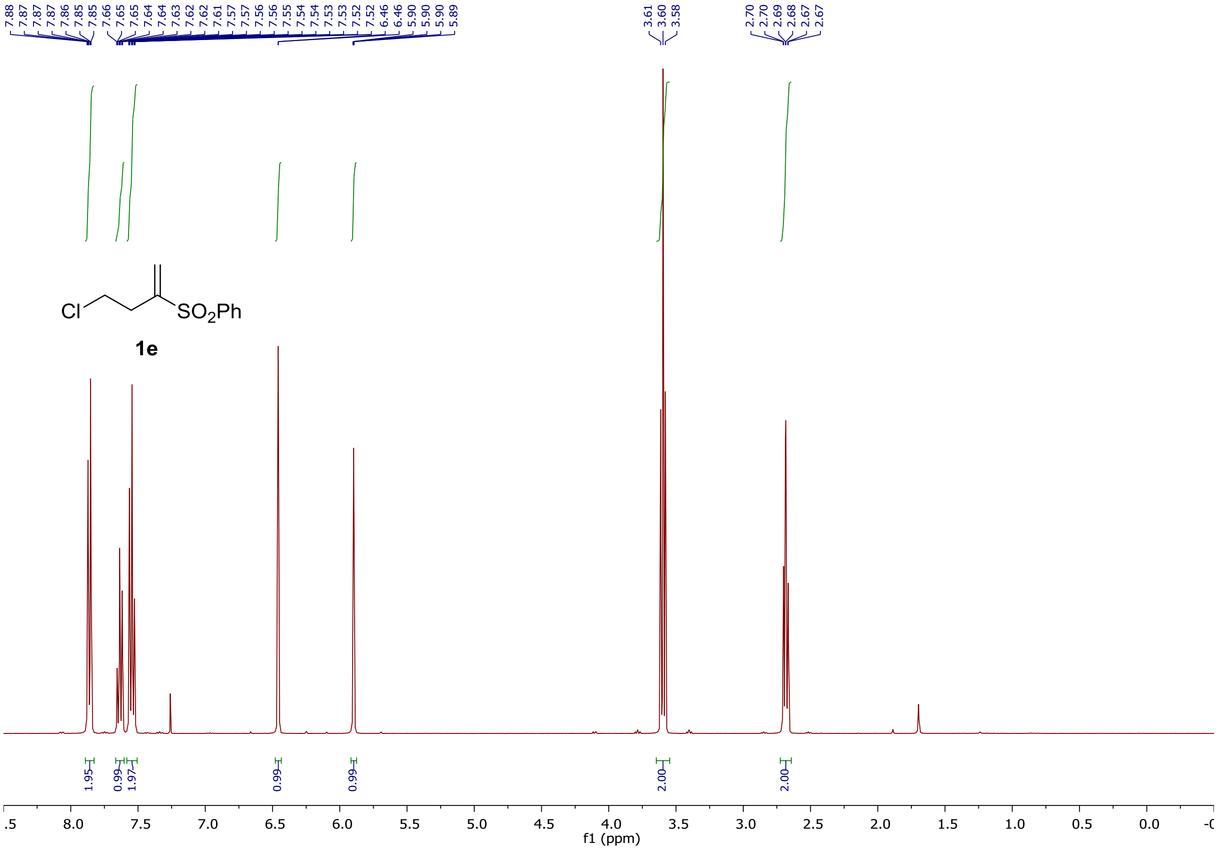

^{13}C NMR (101 MHz, $CDCl_3$)

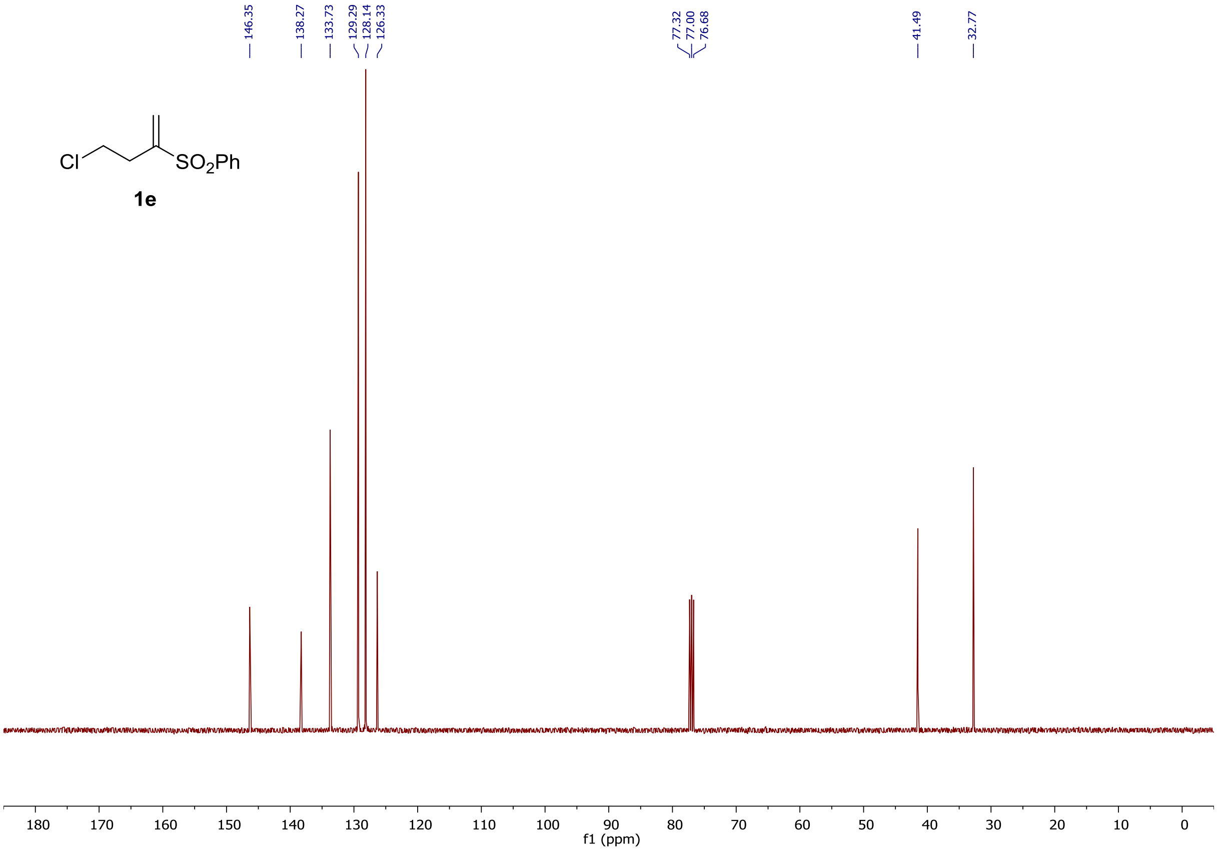

^{1}H NMR (400 MHz, $CDCl_3$)

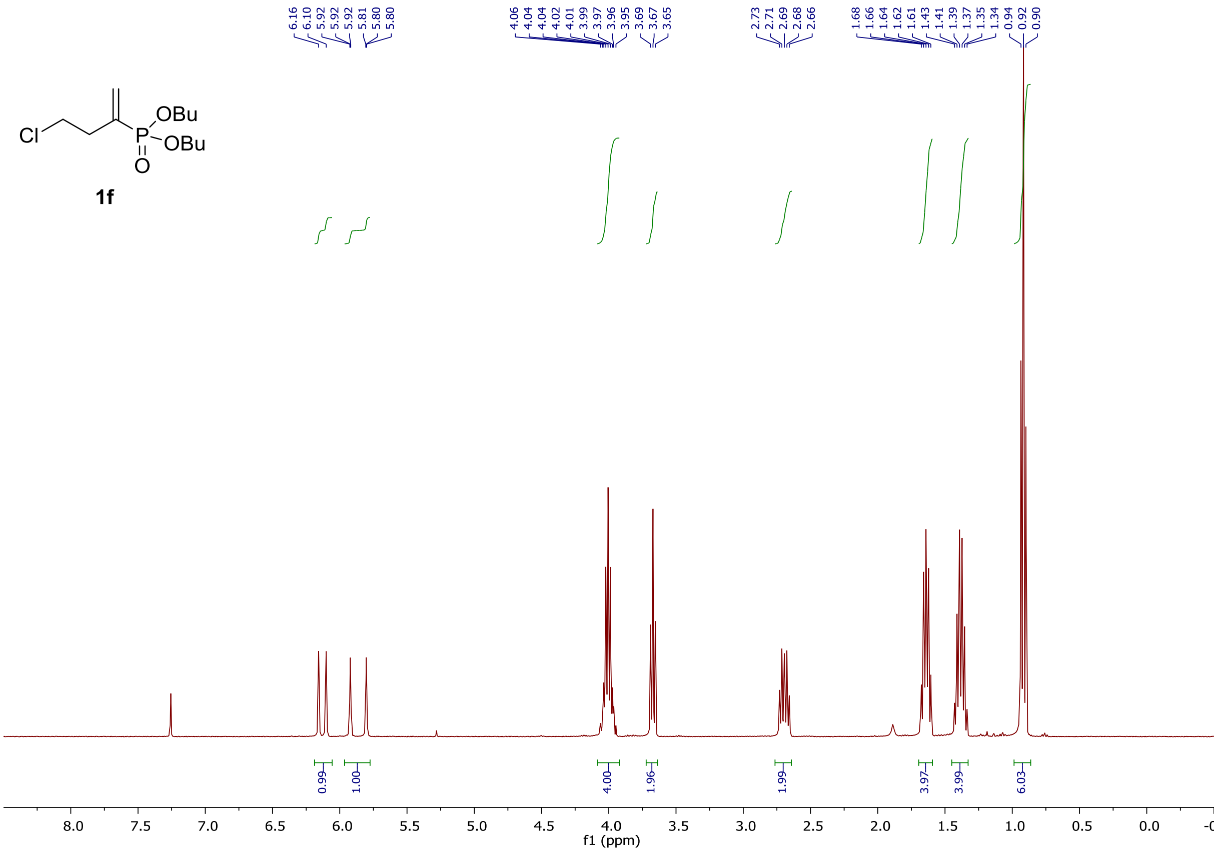

^{13}C NMR (101 MHz, $CDCl_3$)

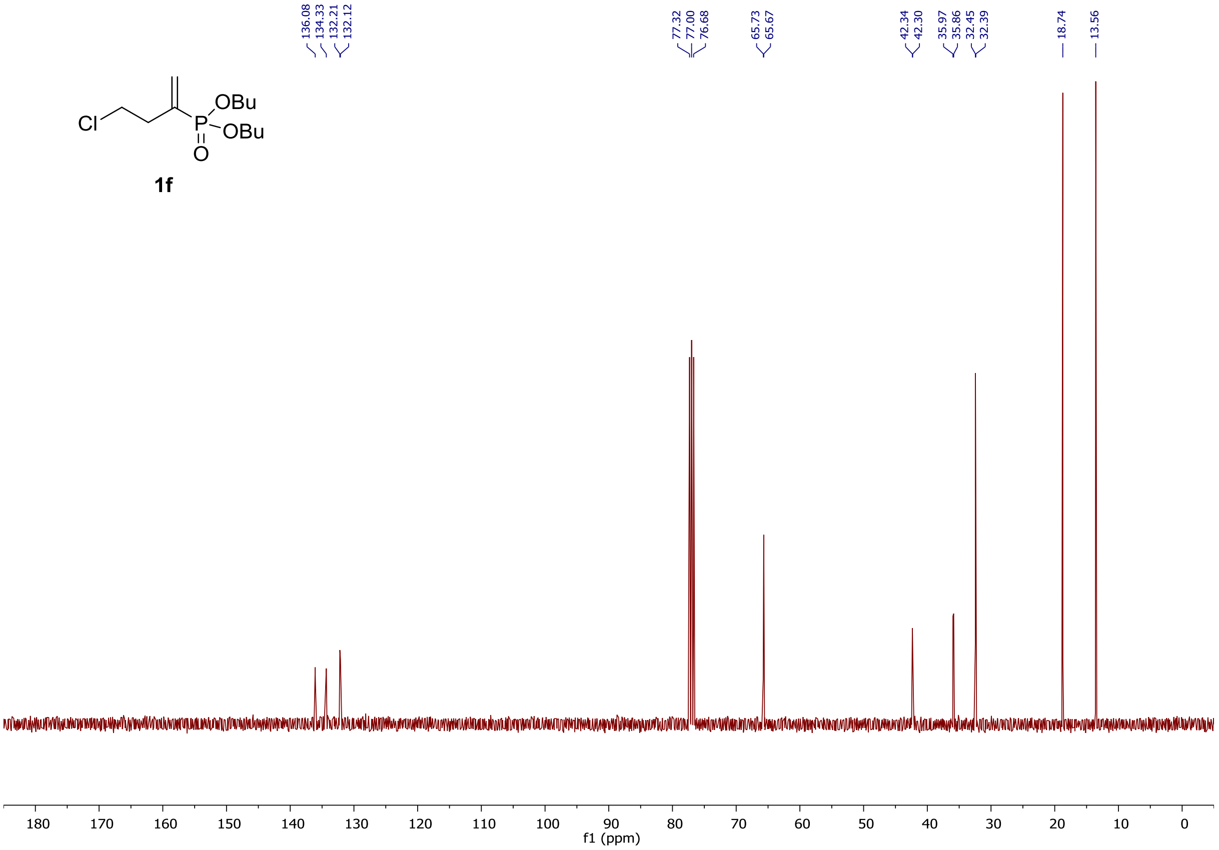

^{1}H NMR (400 MHz, $CDCl_3$)

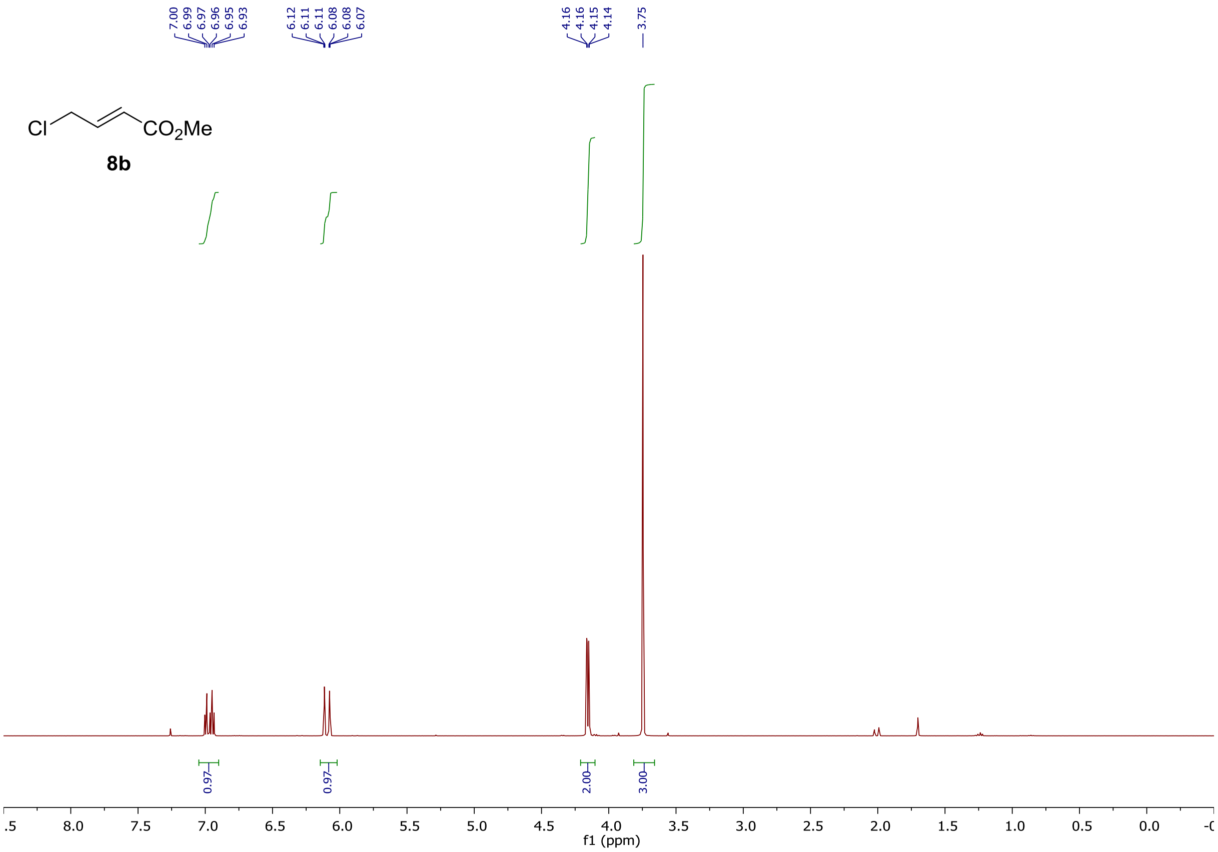

^{13}C NMR (101 MHz, $CDCl_3$)

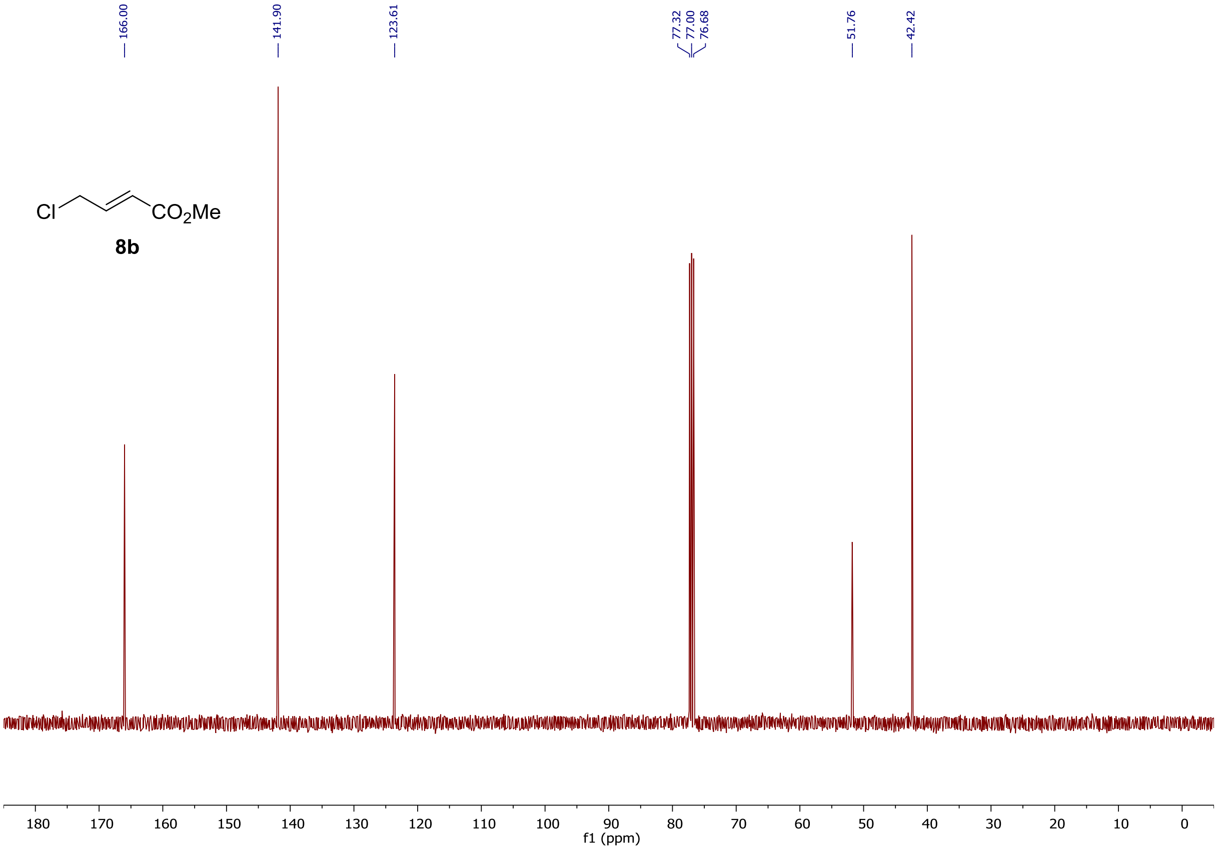

^{1}H NMR (400 MHz, $CDCl_3$)

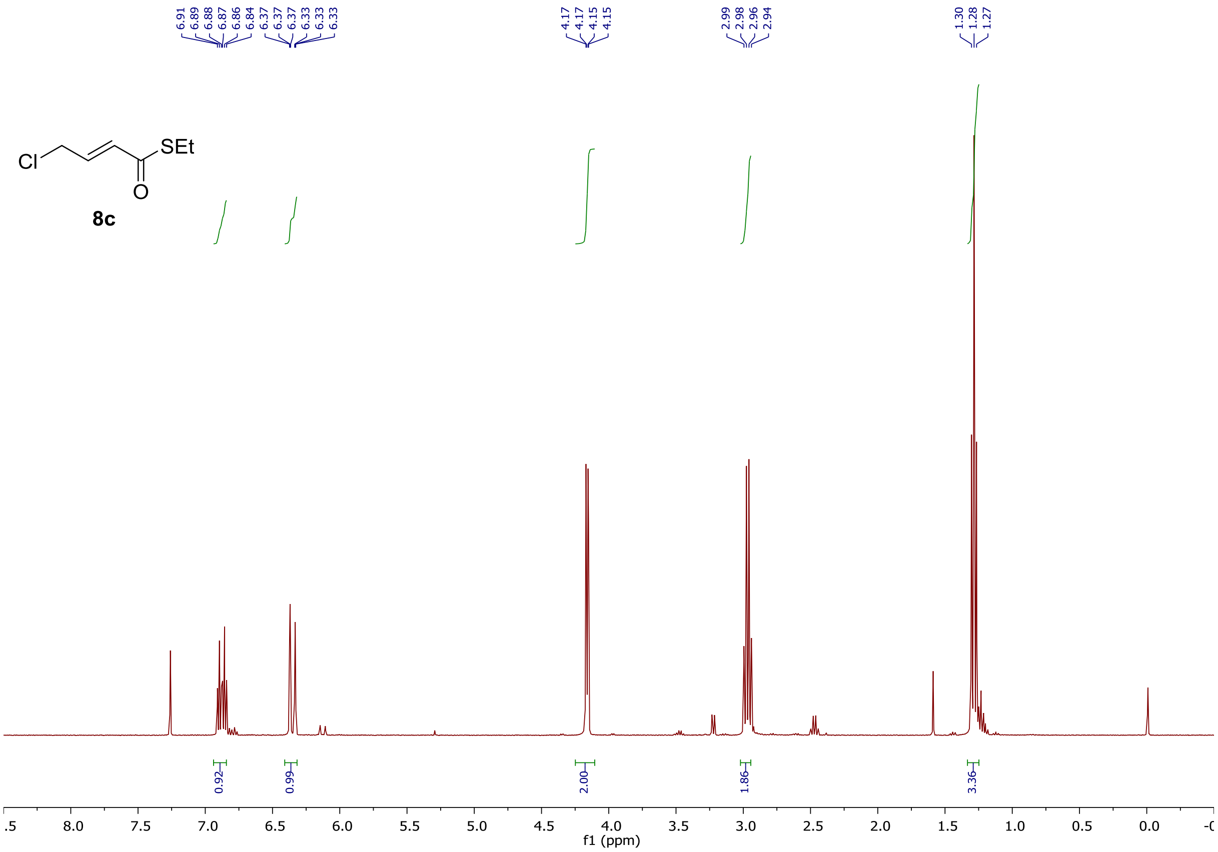

^{13}C NMR (101 MHz, $CDCl_3$)

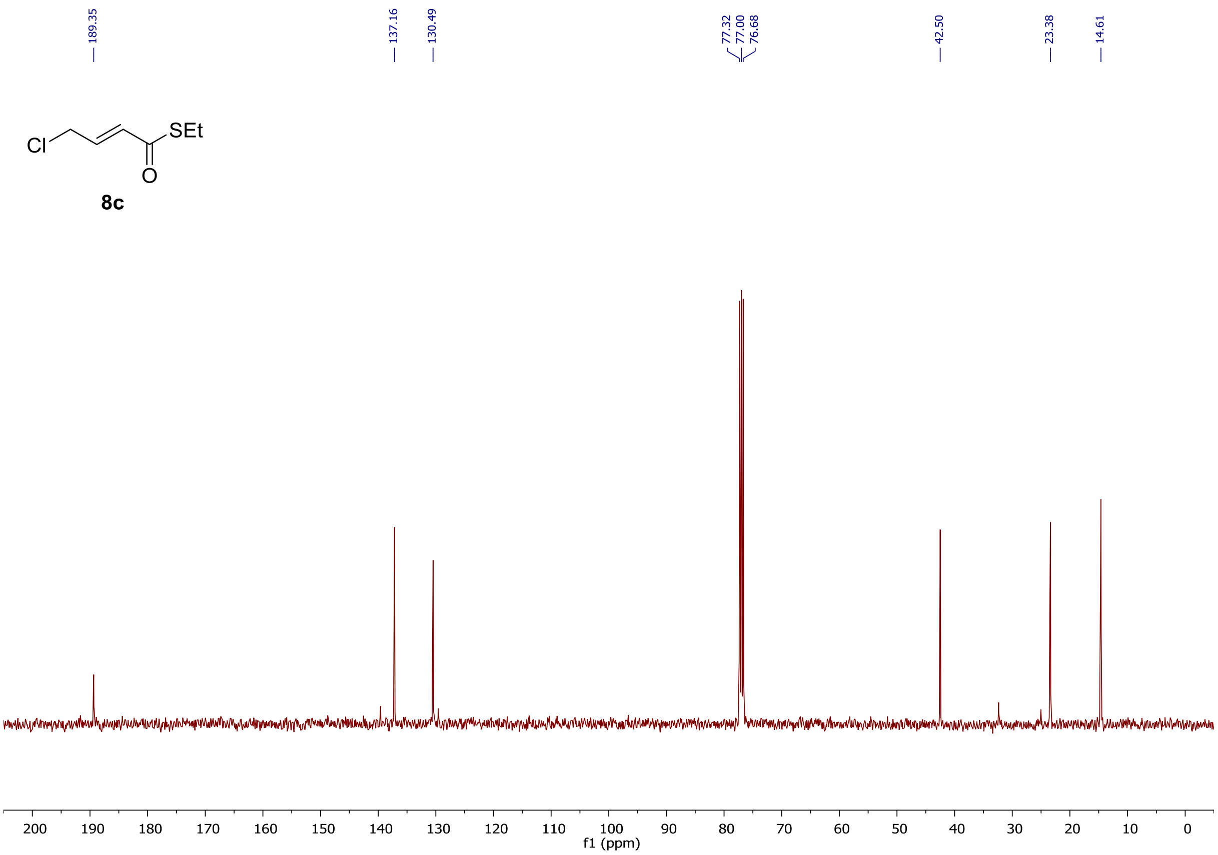

^{1}H NMR (400 MHz, $CDCl_3$)

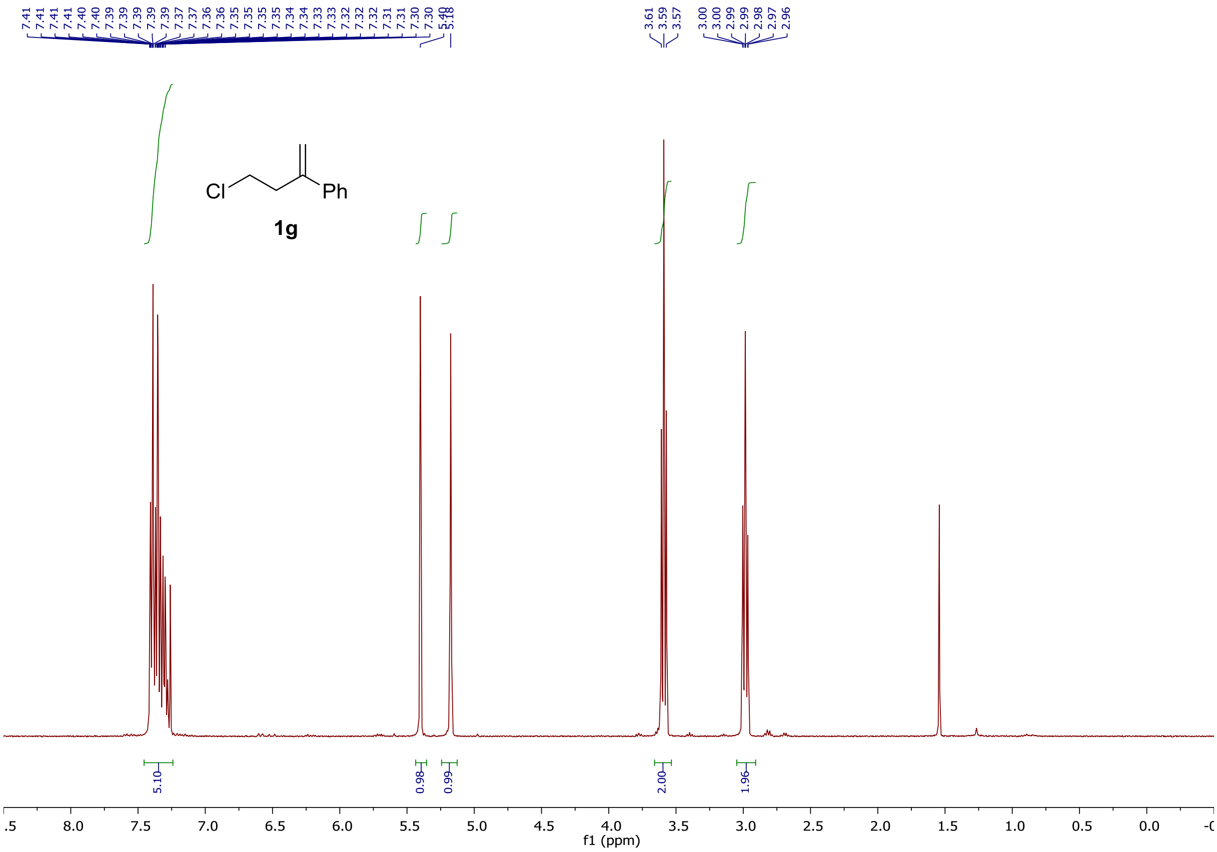

^{13}C NMR (101 MHz, $CDCl_3$)

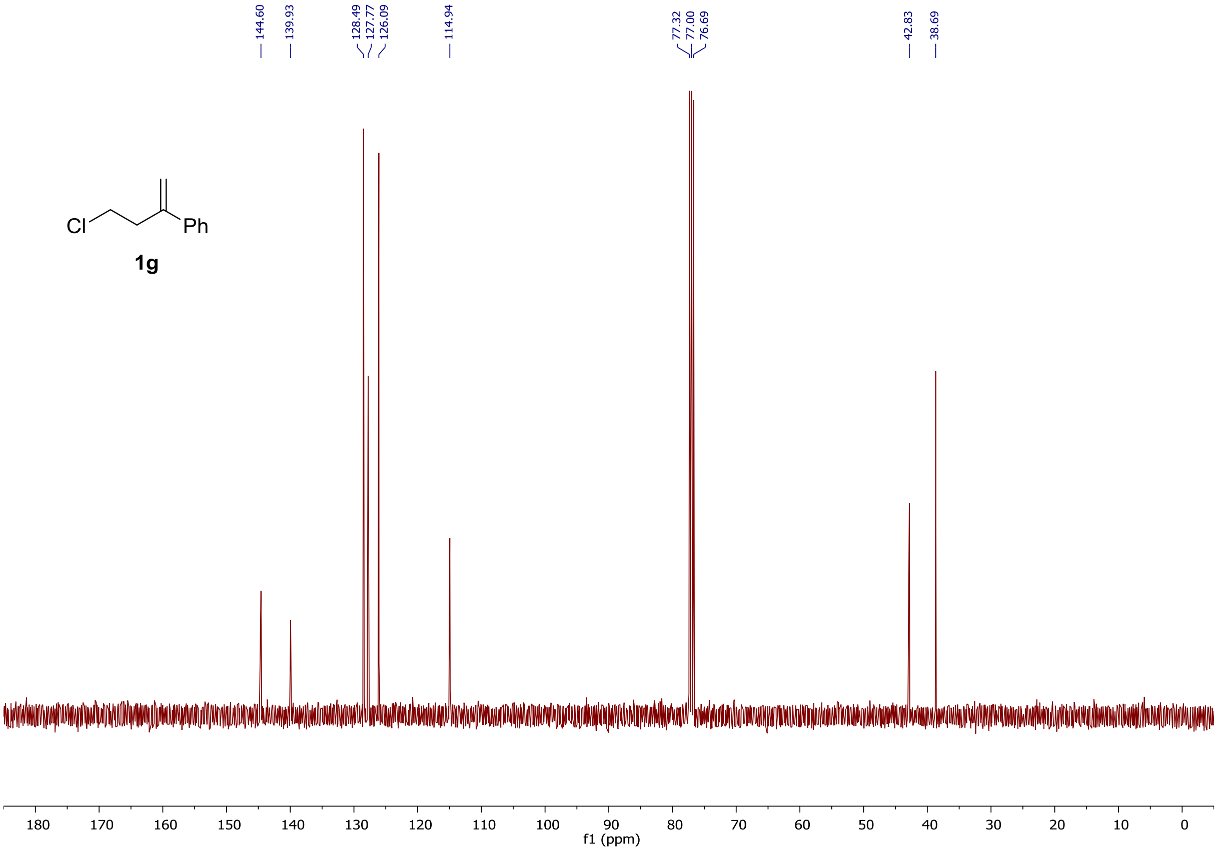

^{1}H NMR (400 MHz, $CDCl_3$)

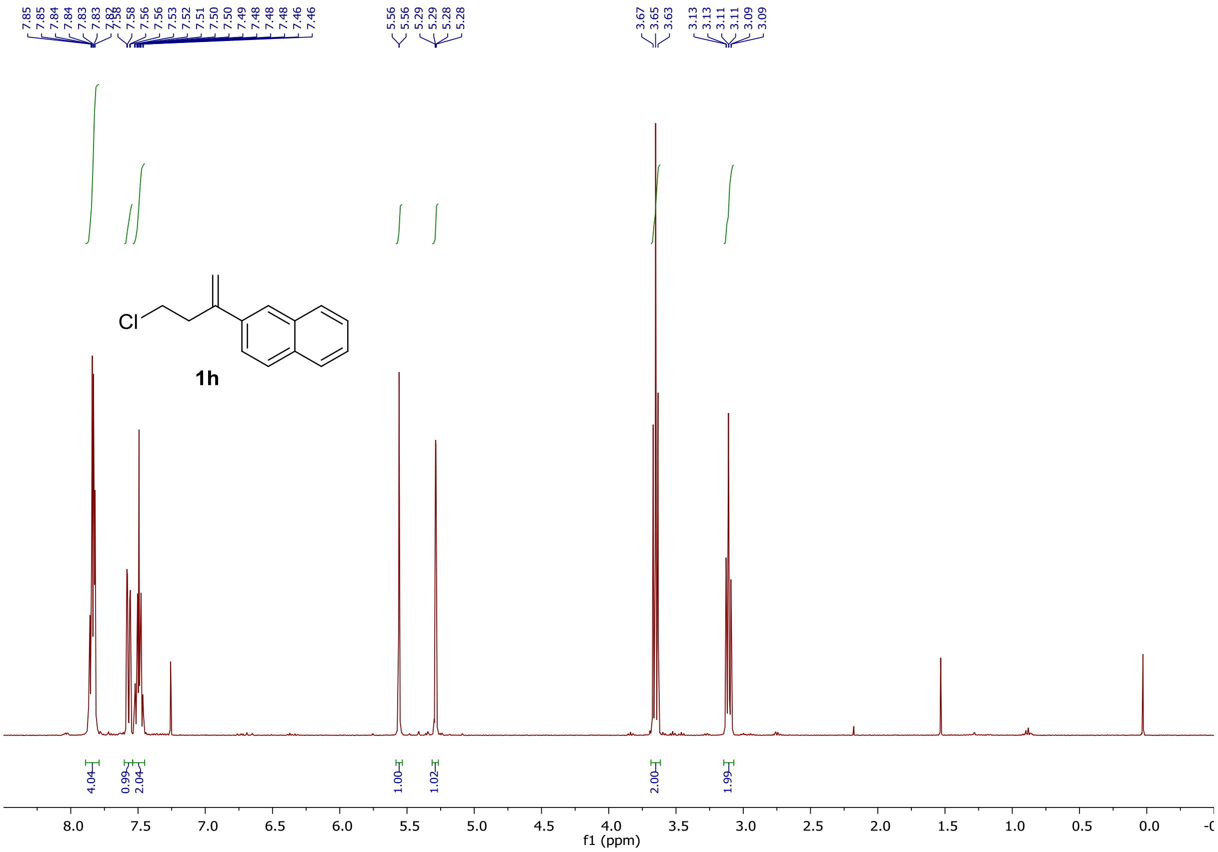

^{13}C NMR (101 MHz, $CDCl_3$)

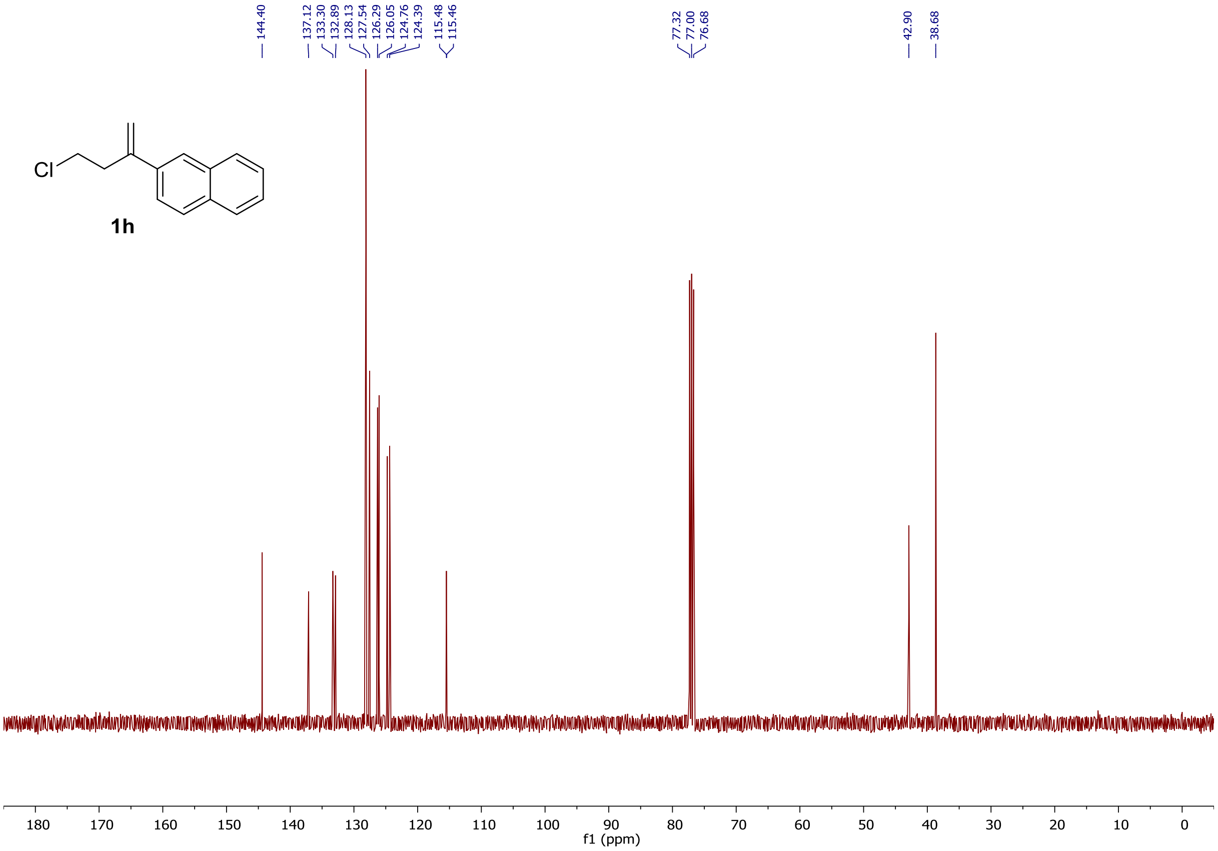

^{1}H NMR (400 MHz, $CDCl_3$)

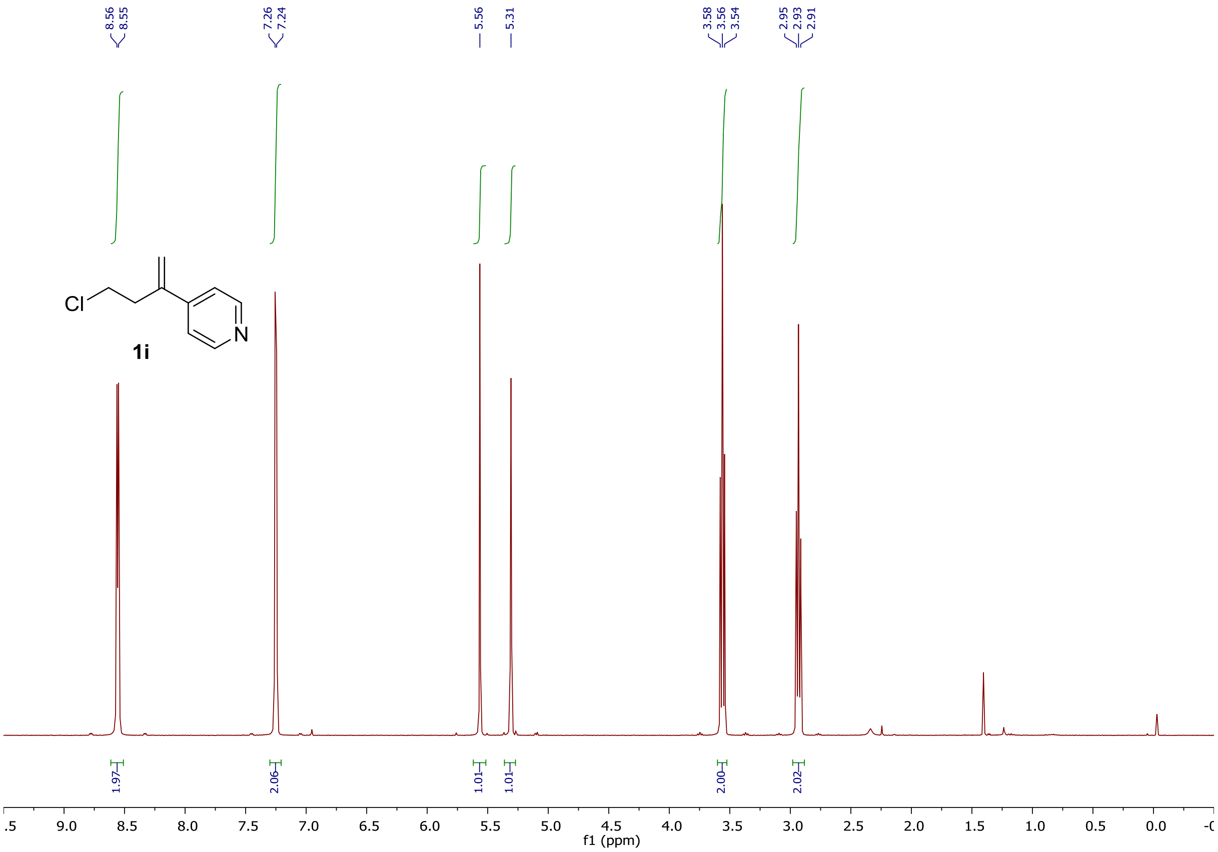

^{13}C NMR (101 MHz, $CDCl_3$)

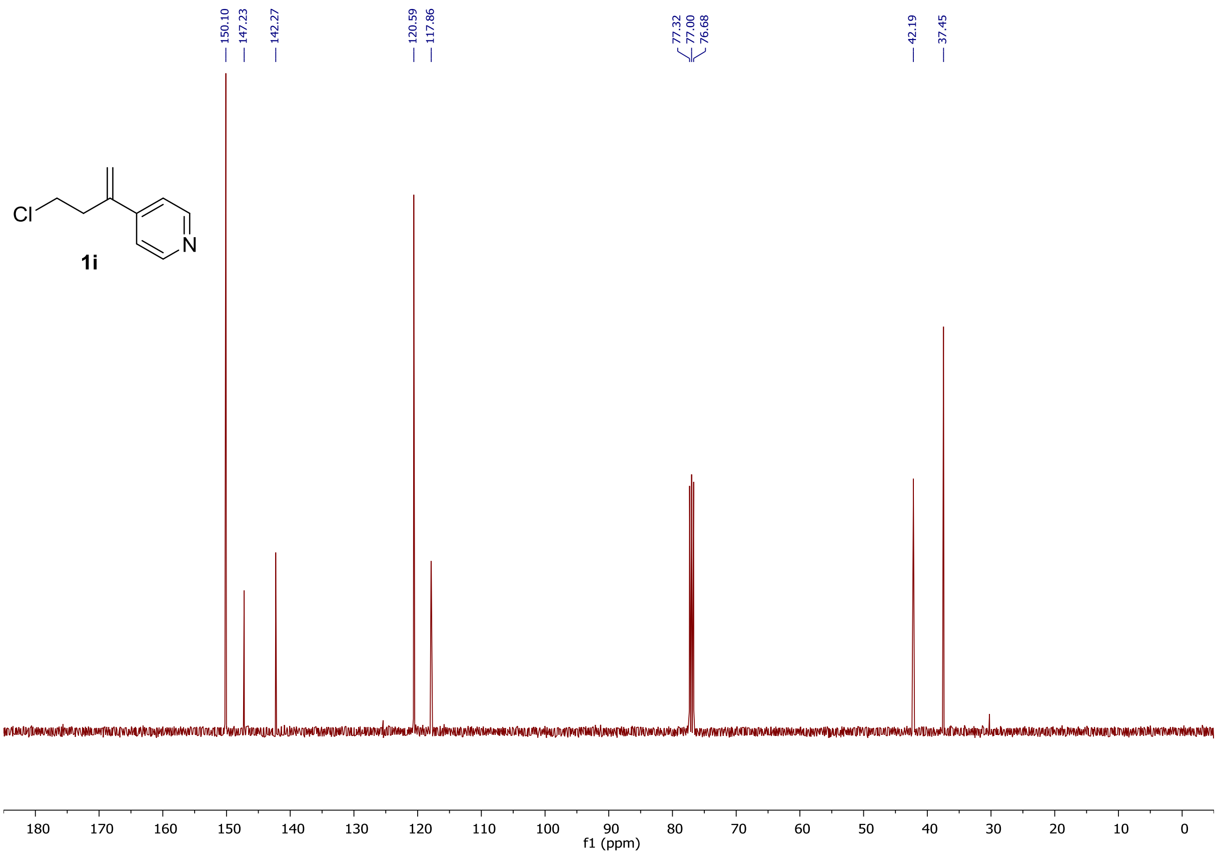

^{1}H NMR (400 MHz, $CDCl_3$)

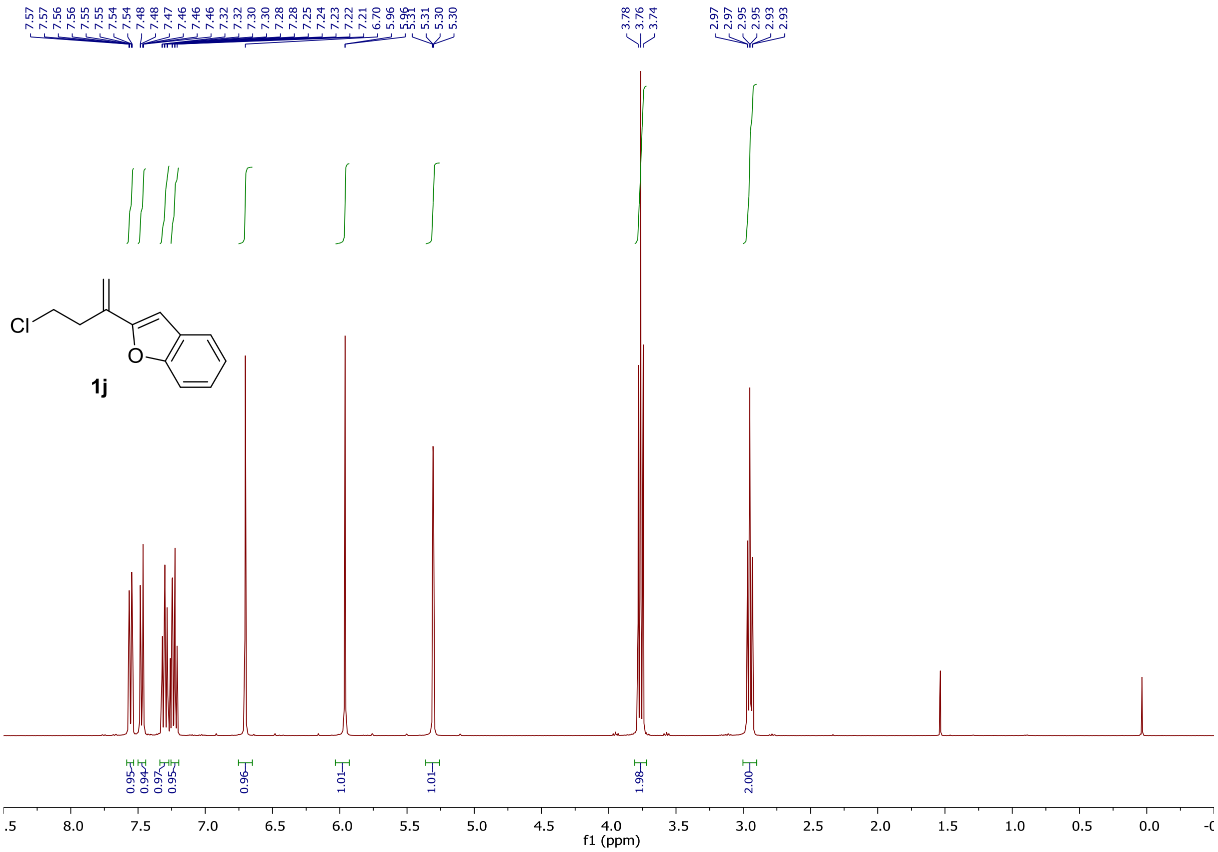

^{13}C NMR (101 MHz, $CDCl_3$)

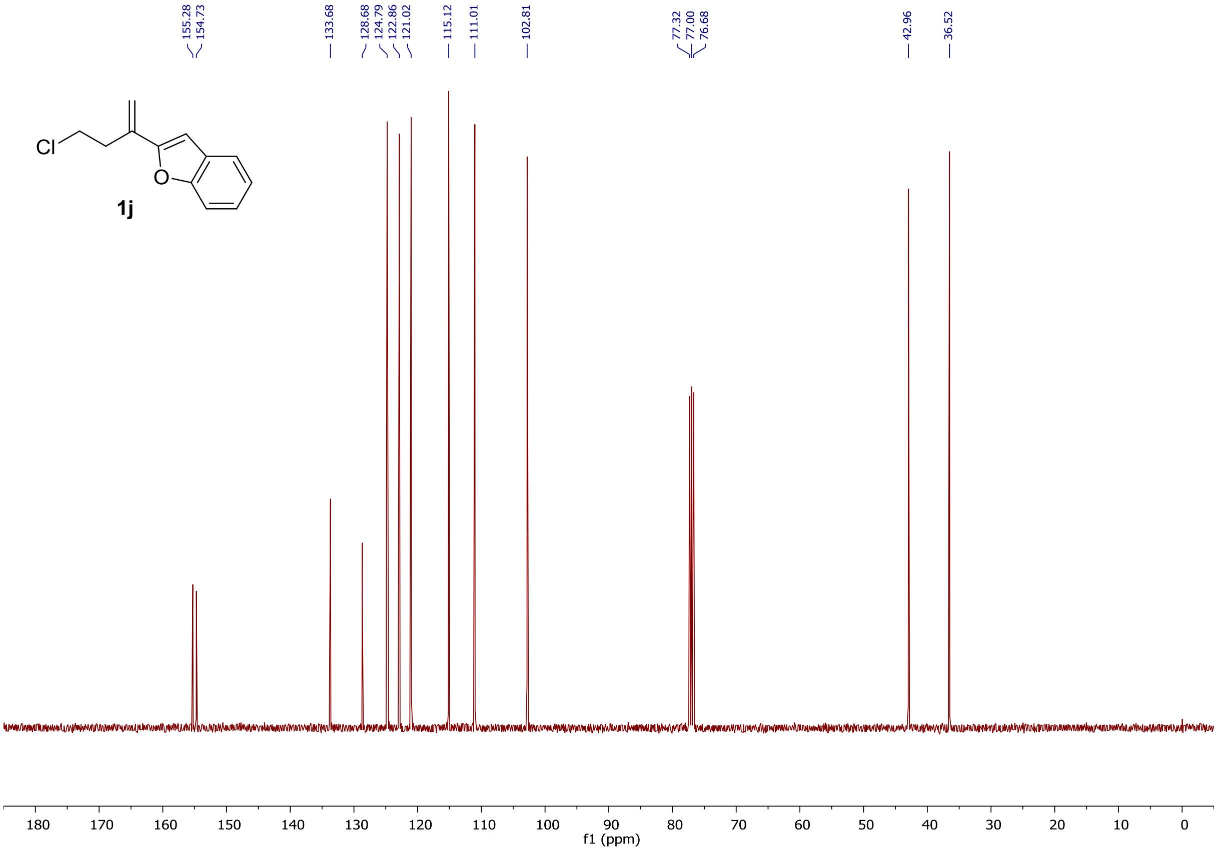

^{1}H NMR (400 MHz, $CDCl_3$)

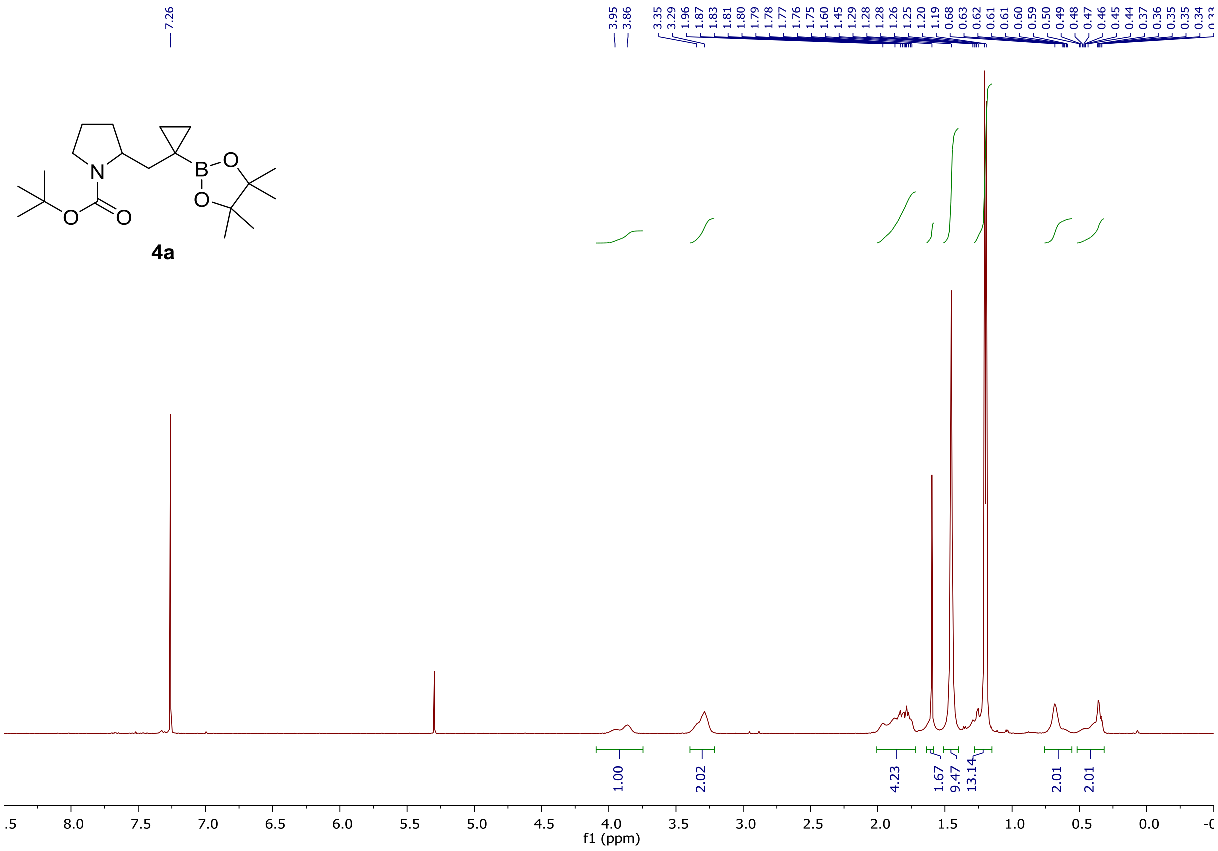

^{13}C NMR (101 MHz, $CDCl_3$)

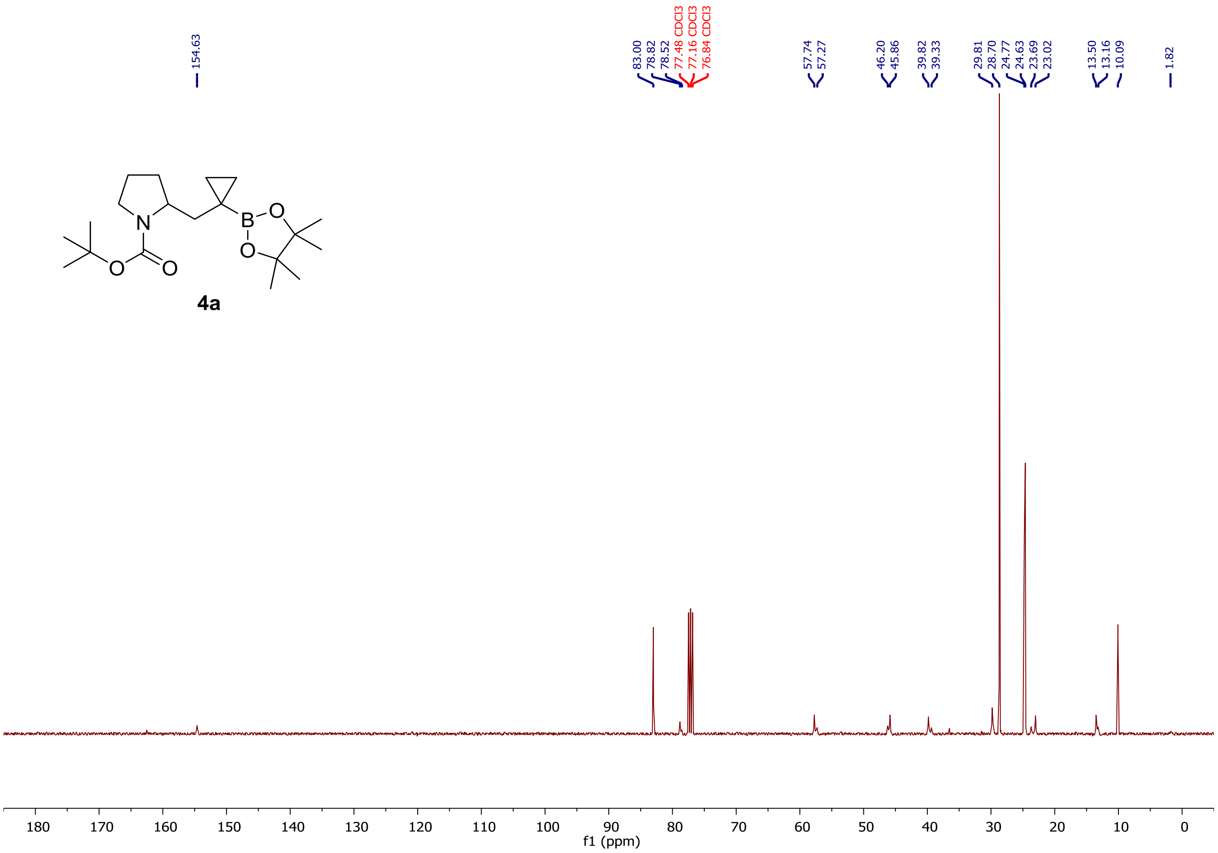

^{1}H NMR (500 MHz, DMSO-d_6, 100 ºC)

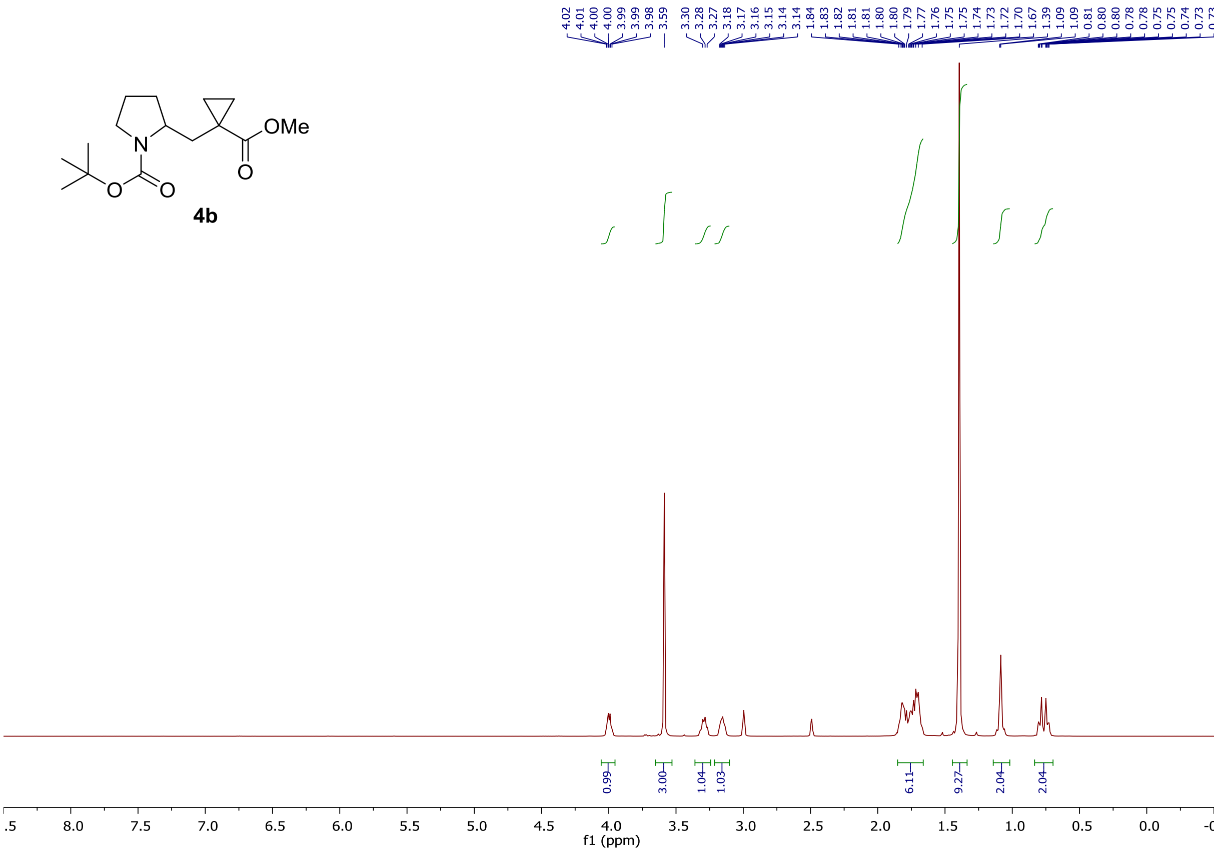

^{13}C NMR (126 MHz, DMSO-d_6, 100 ºC)

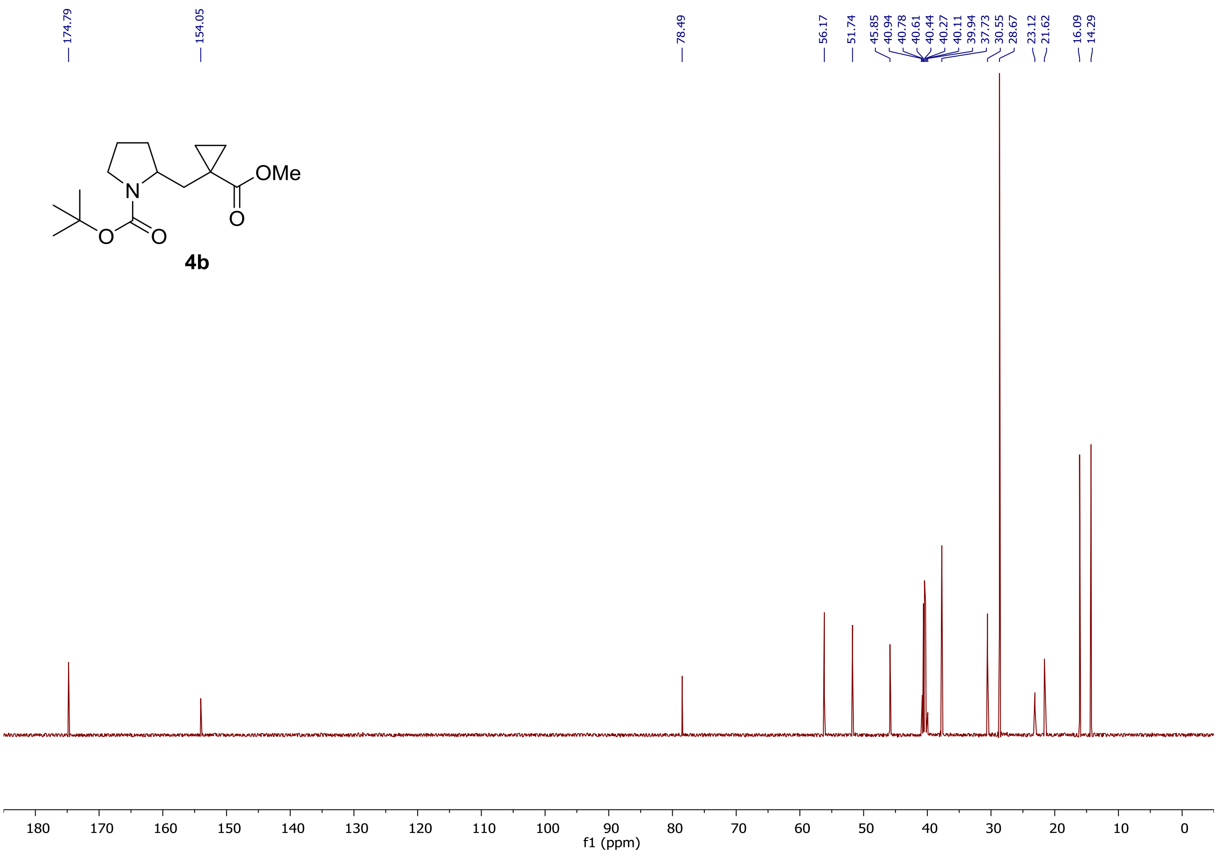

^{1}H NMR (500 MHz, DMSO-d_6, 100 ºC)

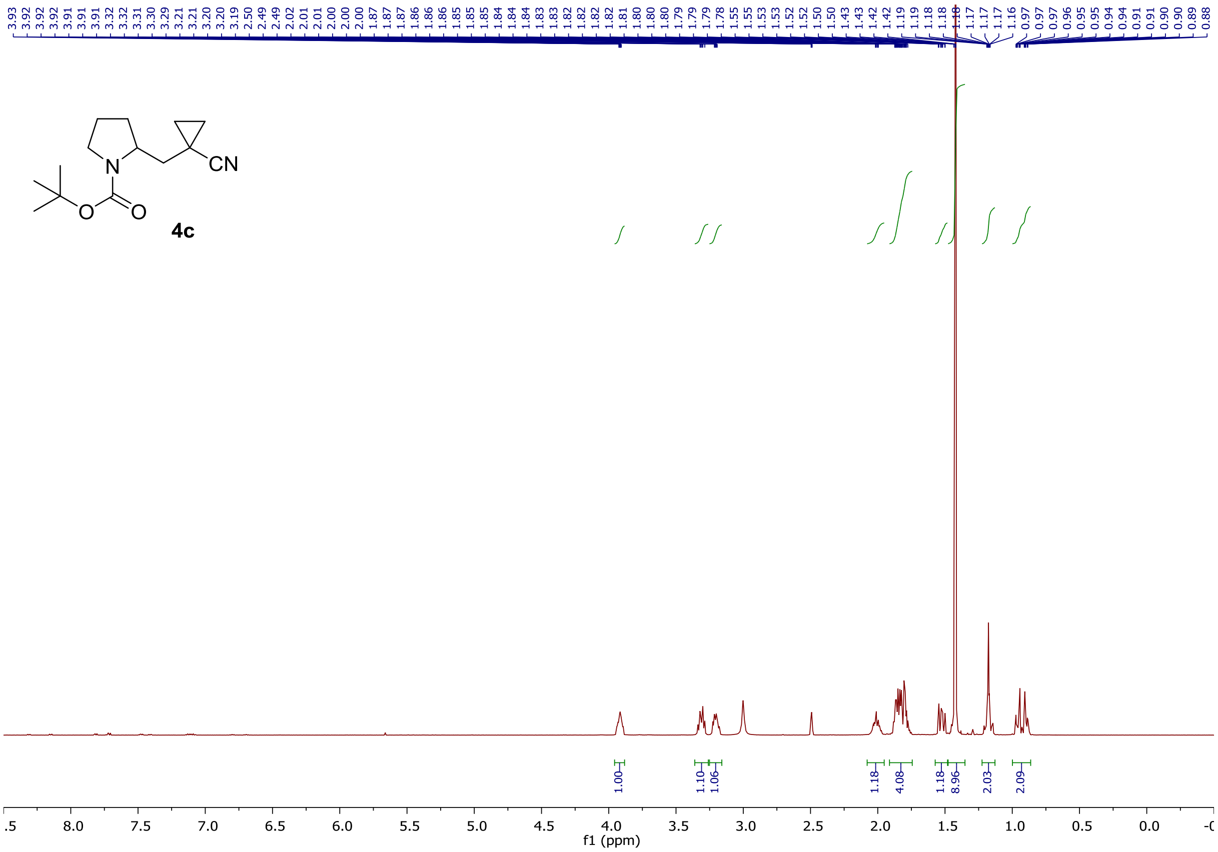

^{13}C NMR (126 MHz, DMSO-d_6, 100 ºC)

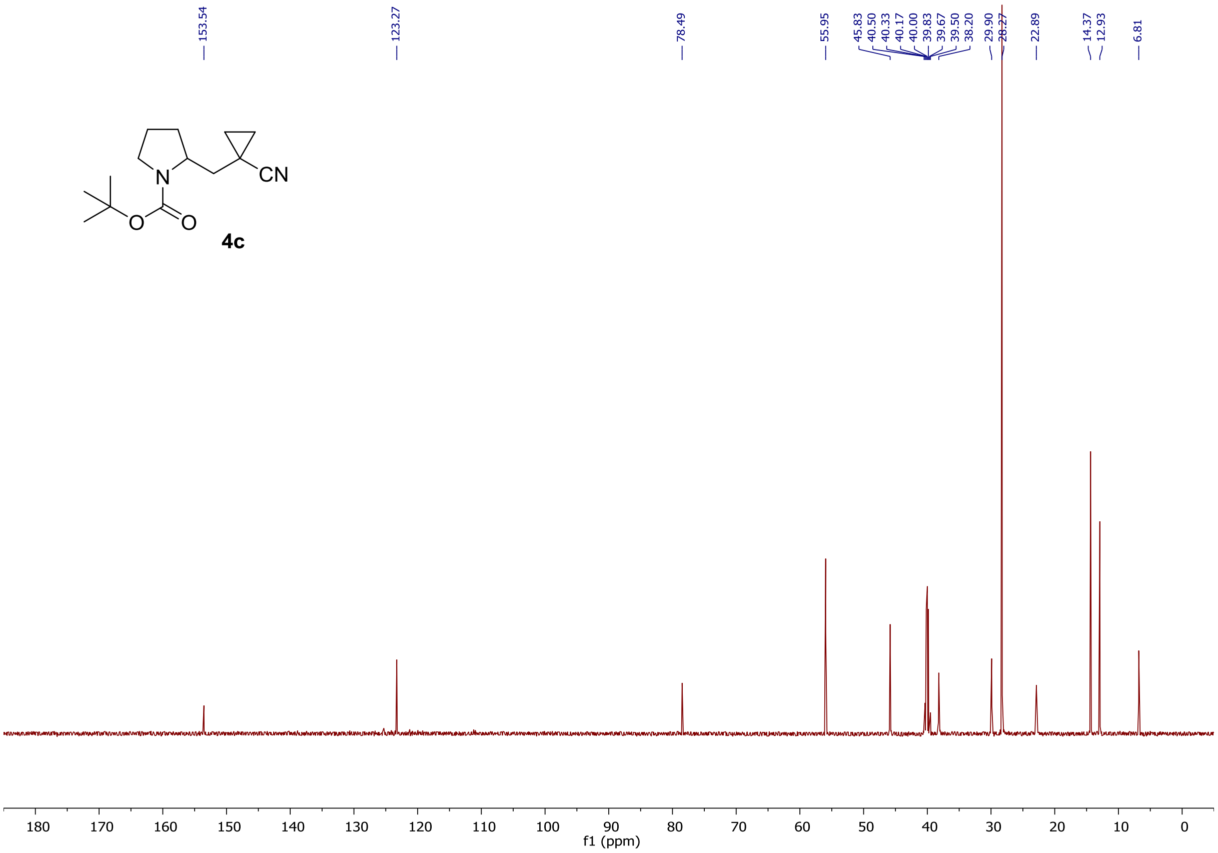

^{1}H NMR (500 MHz, DMSO-d_6, 100 °C)

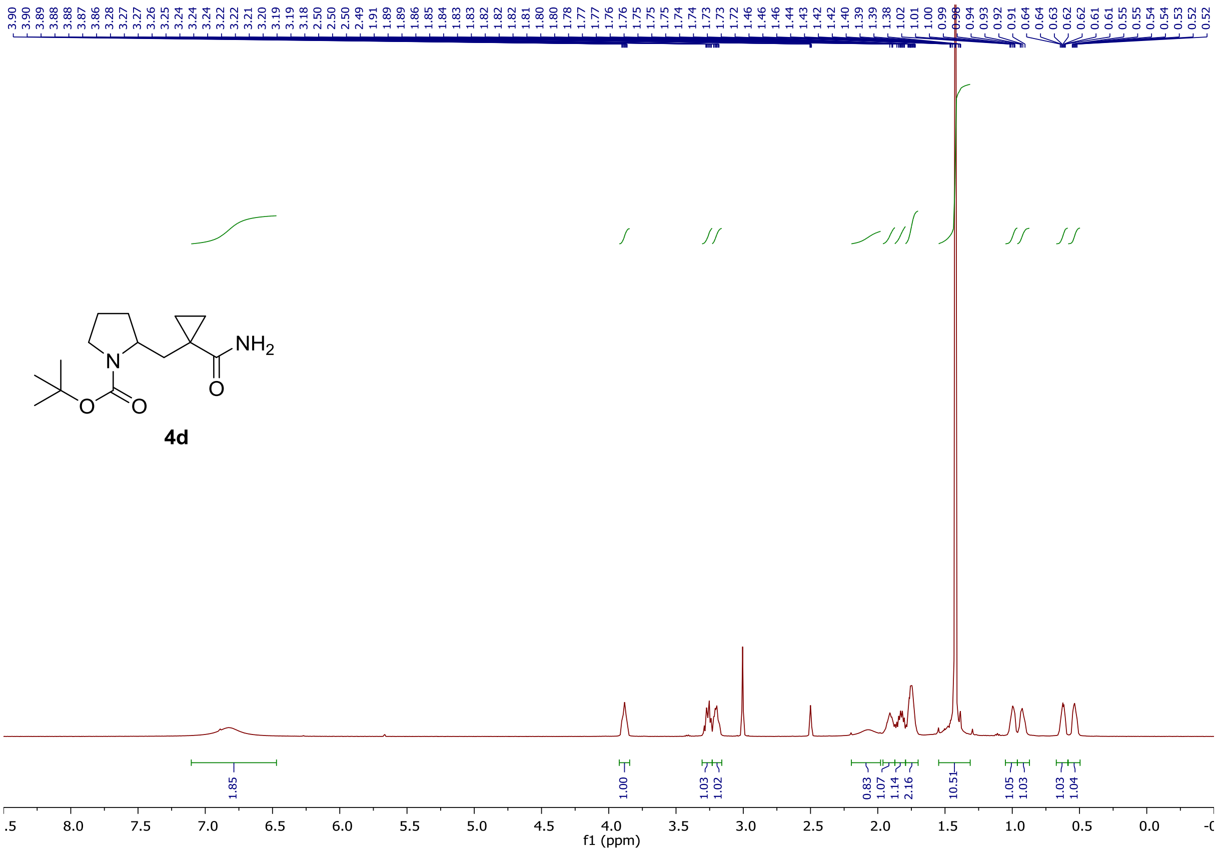

^{13}C NMR (126 MHz, DMSO-d_6, 100 °C)

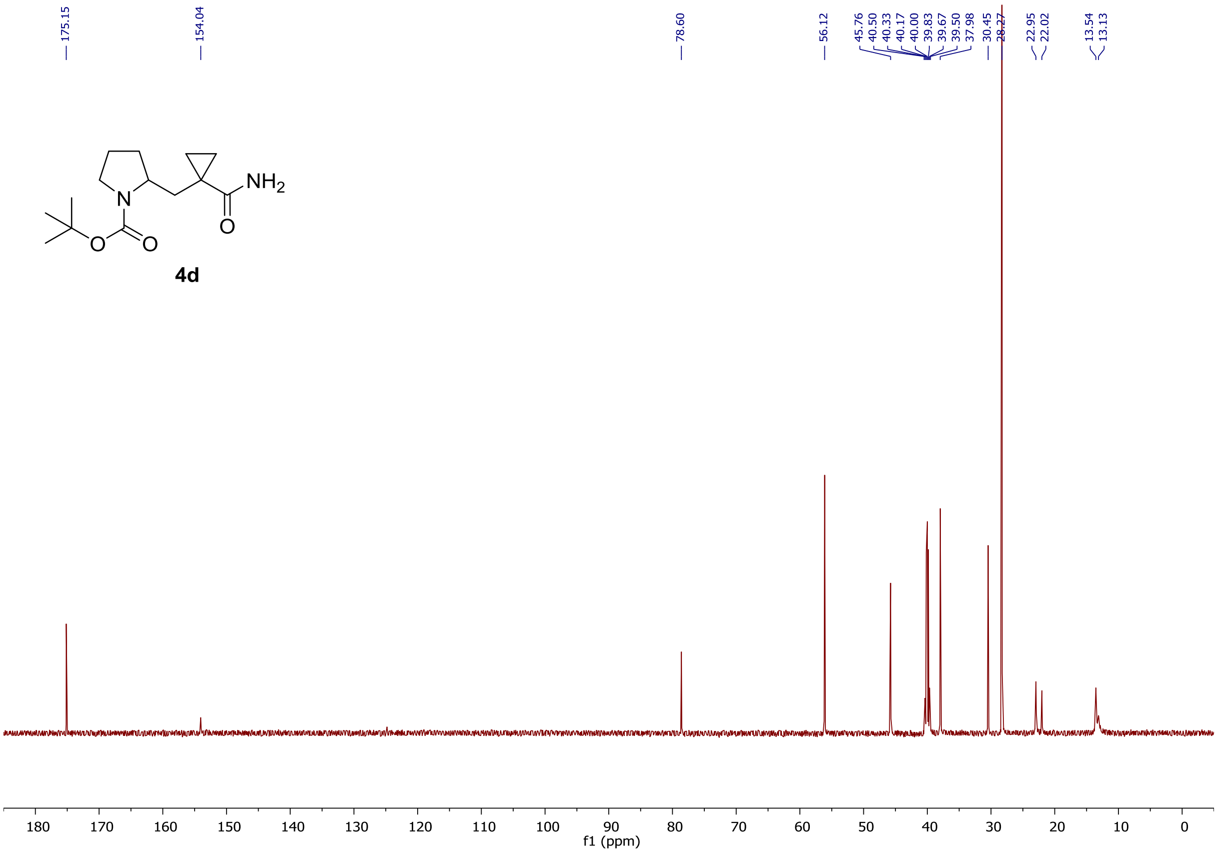

^{1}H NMR (500 MHz, DMSO-d_6, 100 ºC)

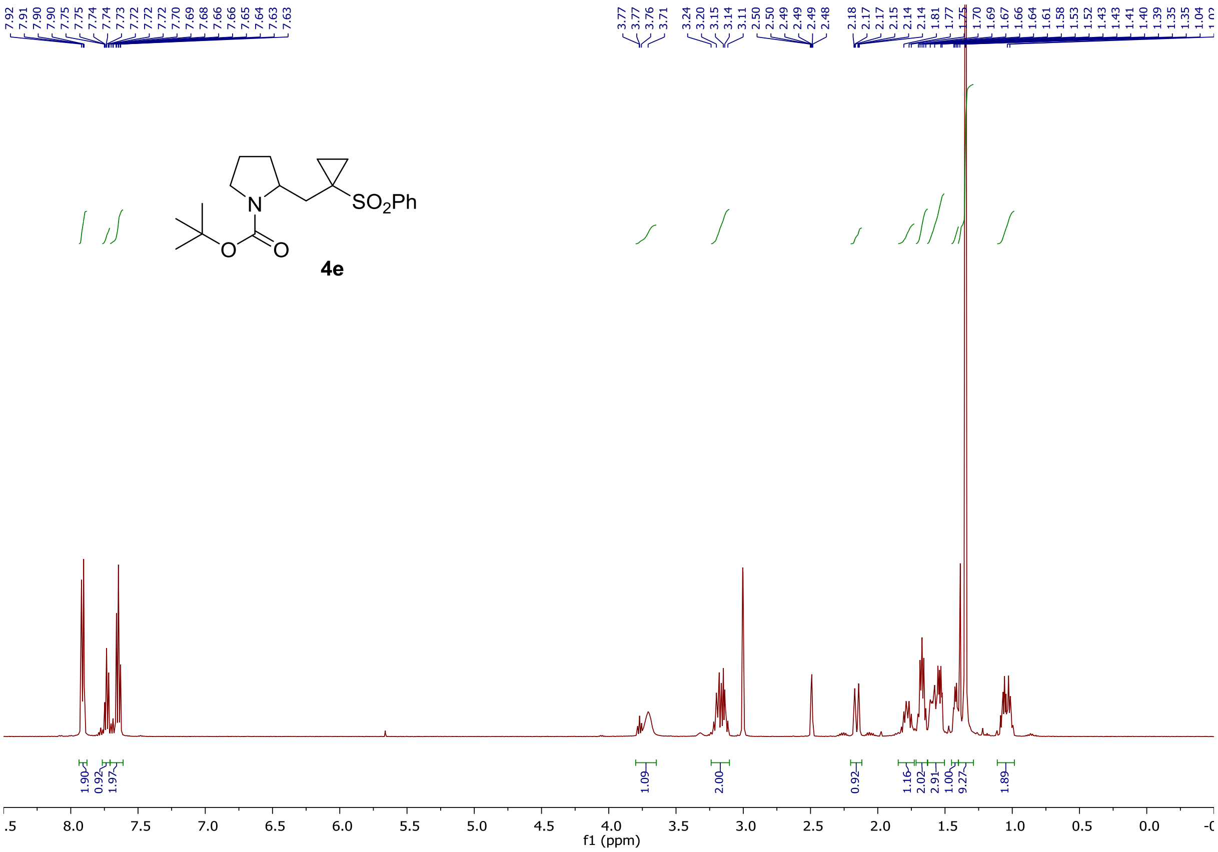

^{13}C NMR (126 MHz, DMSO-d_6, 100 ºC)

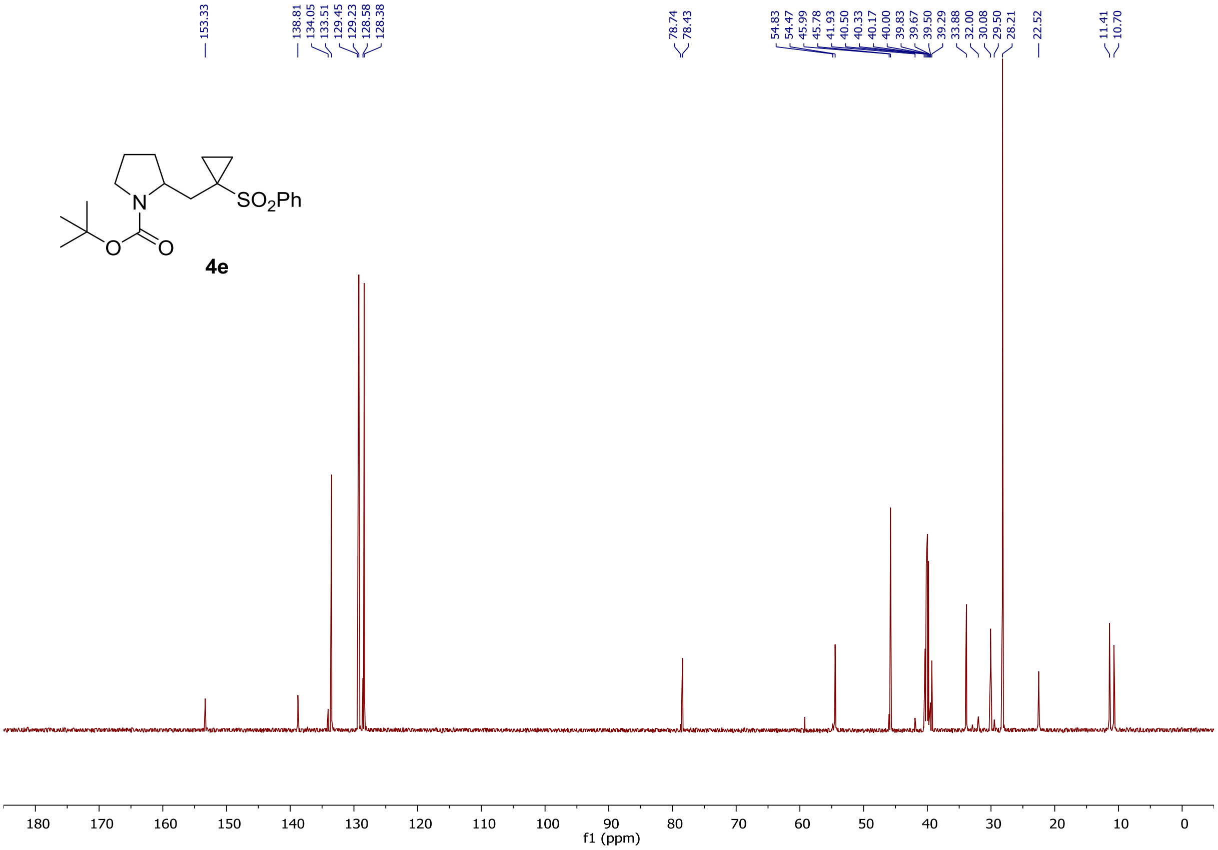

^{1}H NMR (500 MHz, DMSO-d_6, 100 ºC)

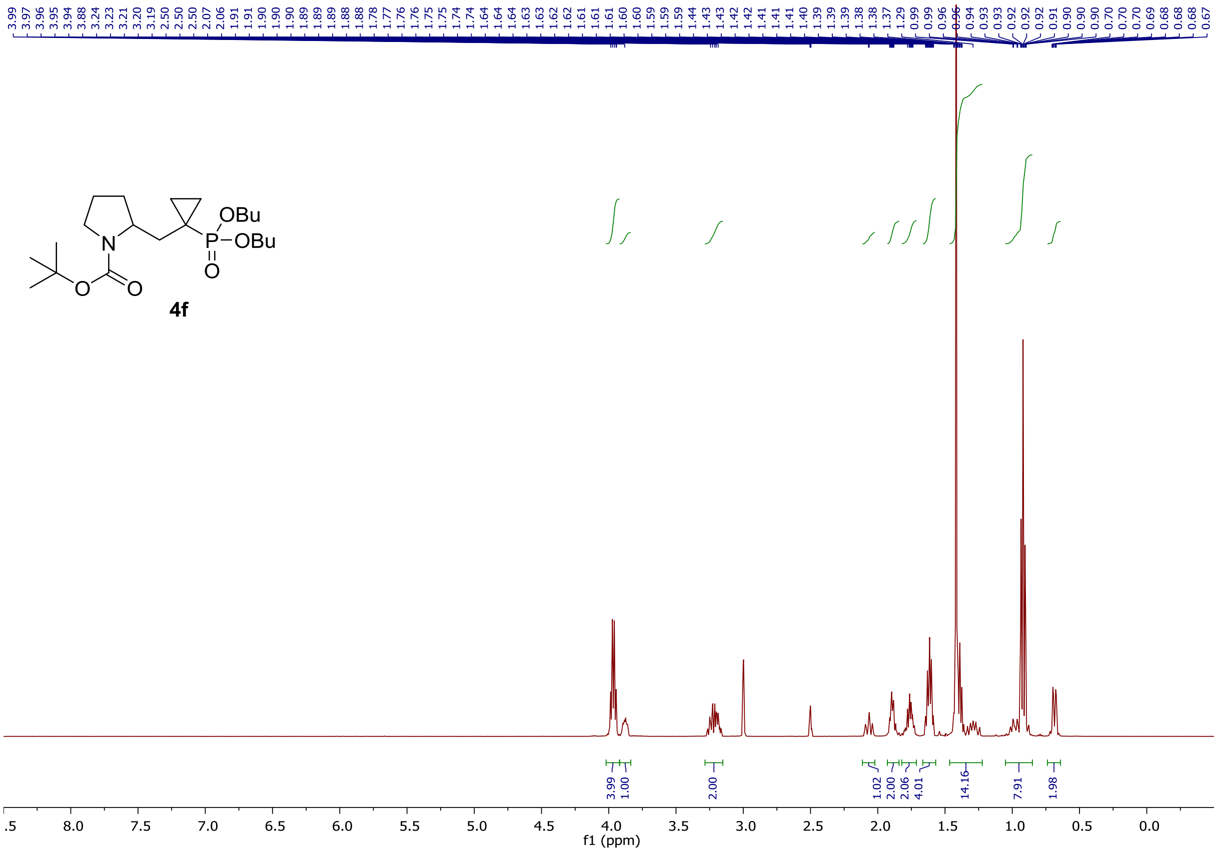

^{13}C NMR (126 MHz, DMSO-d_6, 100 ºC)

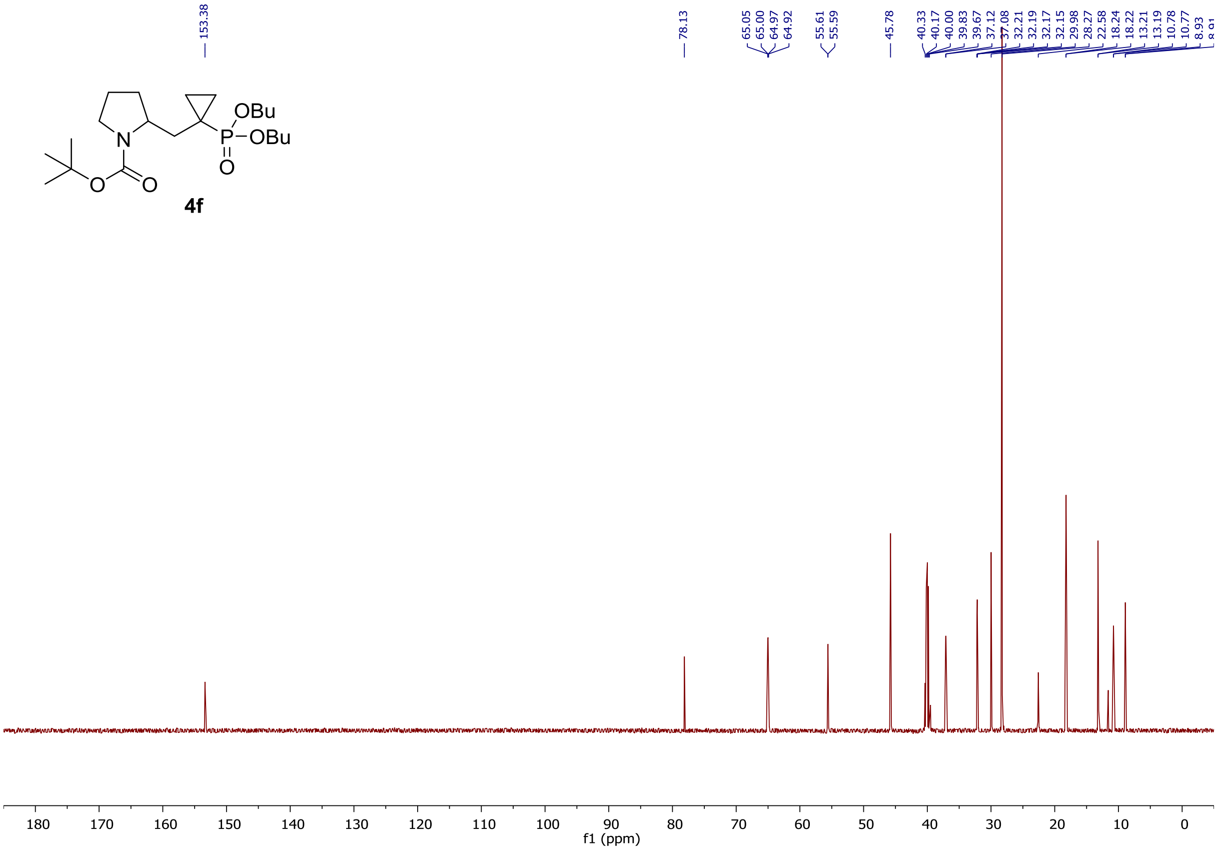

^{1}H NMR (400 MHz, $CDCl_3$)

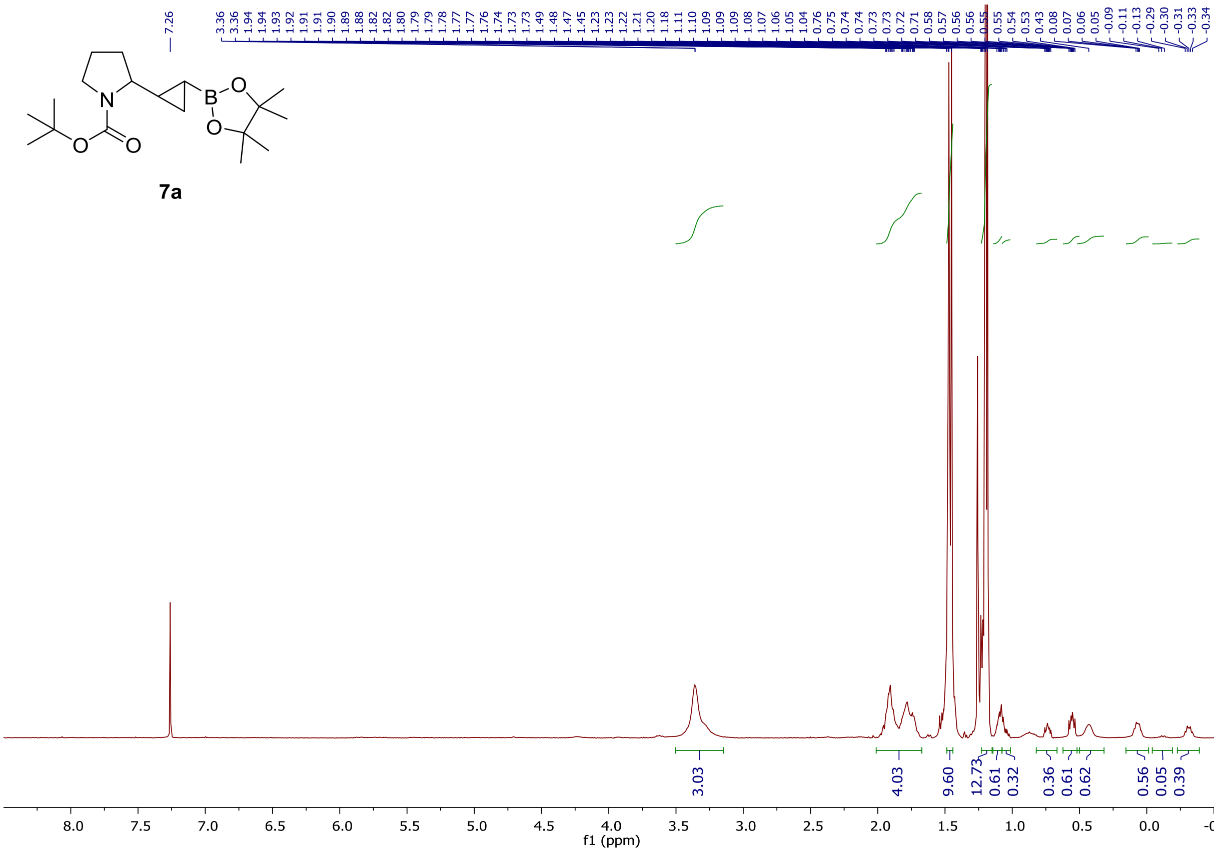

^{13}C NMR (101 MHz, $CDCl_3$)

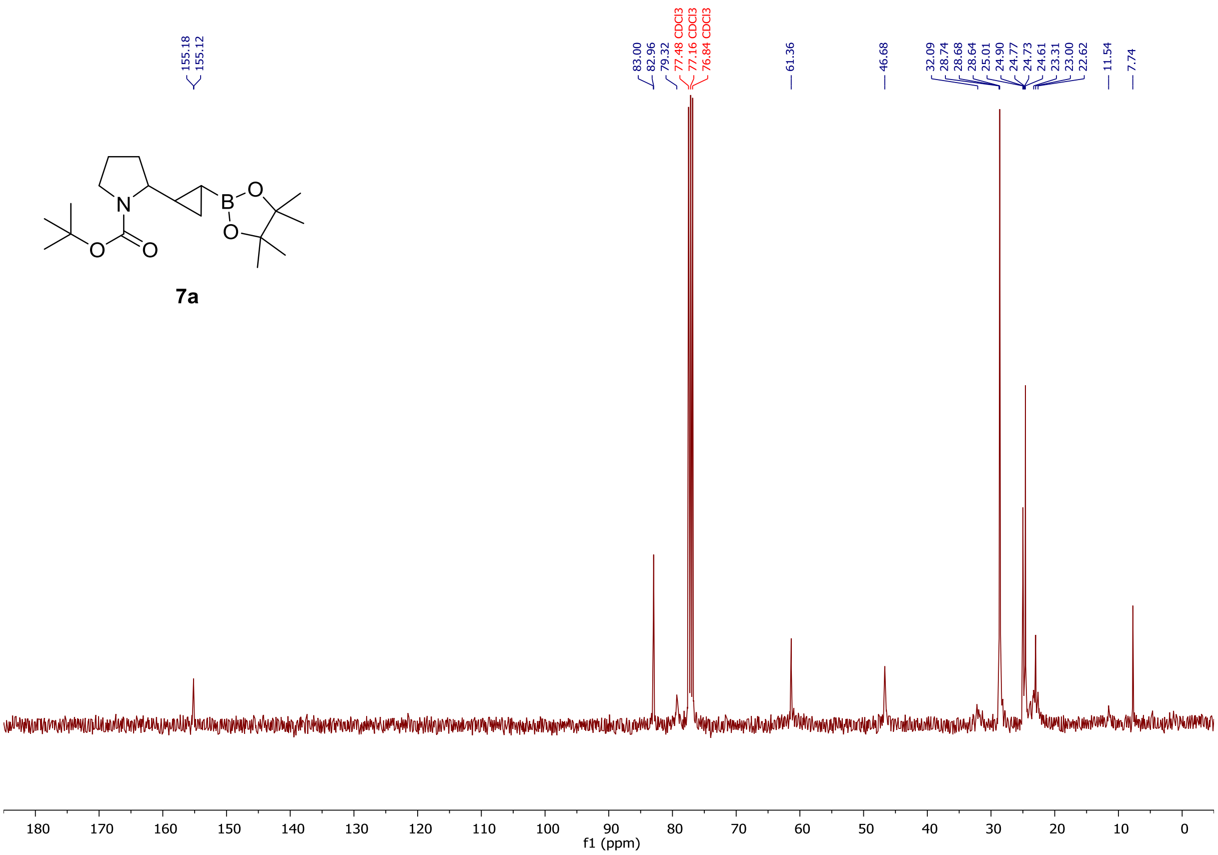

^{1}H NMR (500 MHz, DMSO-d_6, 100 ºC)

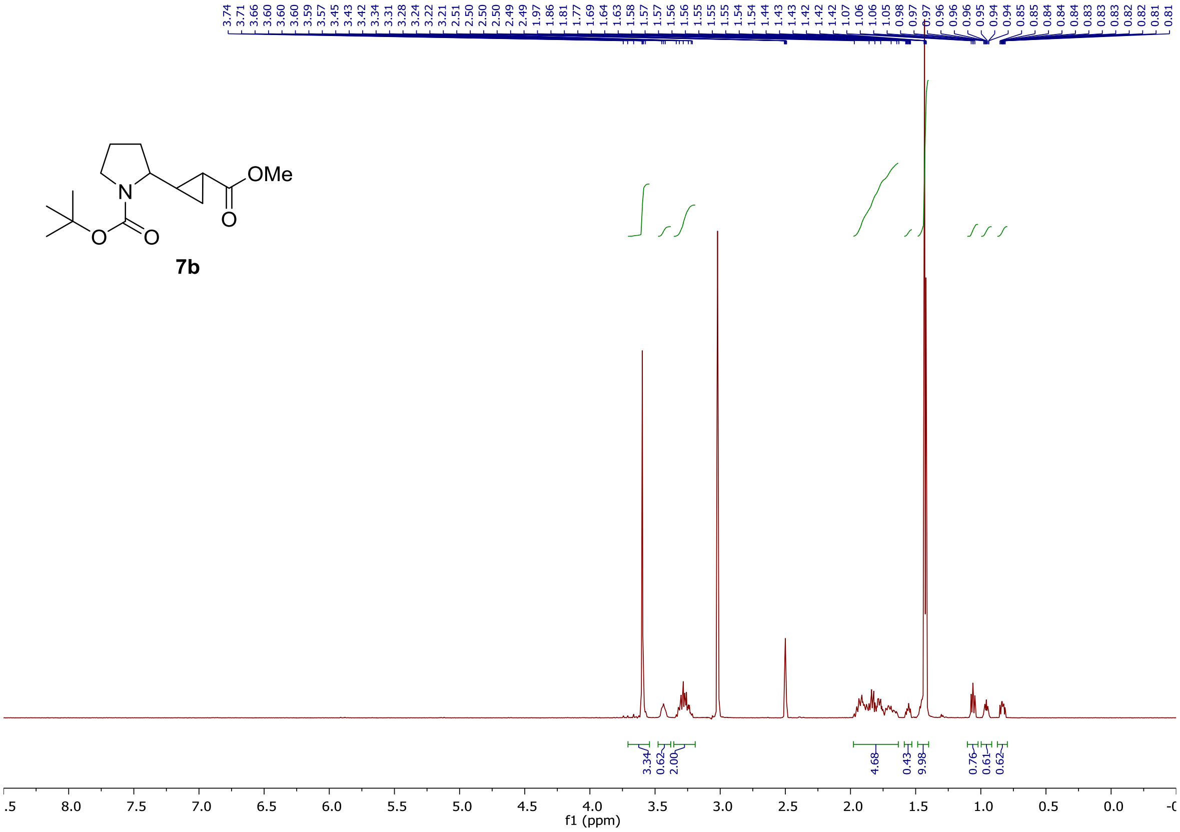

^{13}C NMR (126 MHz, DMSO-d_6, 100 ºC)

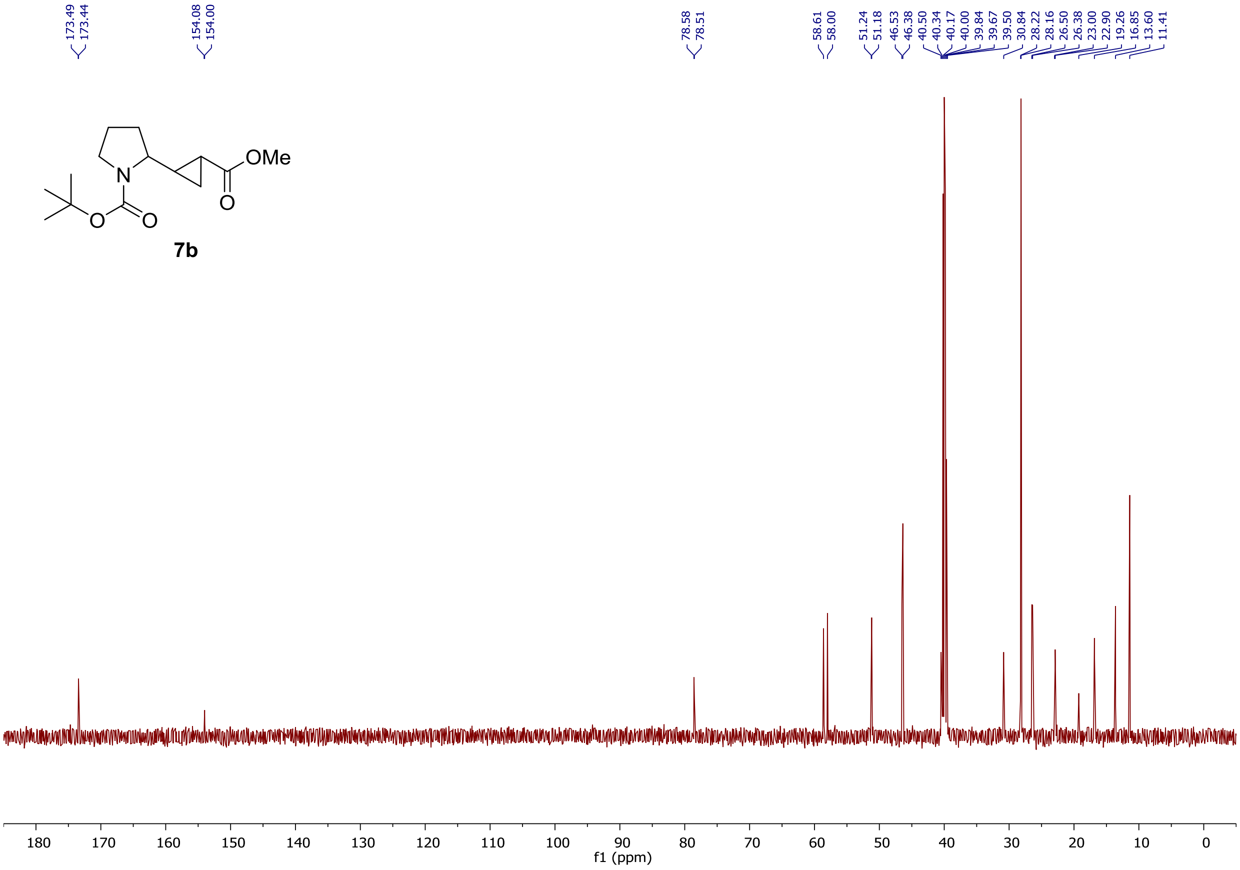

^{1}H NMR (500 MHz, DMSO-d_6, 100 ºC)

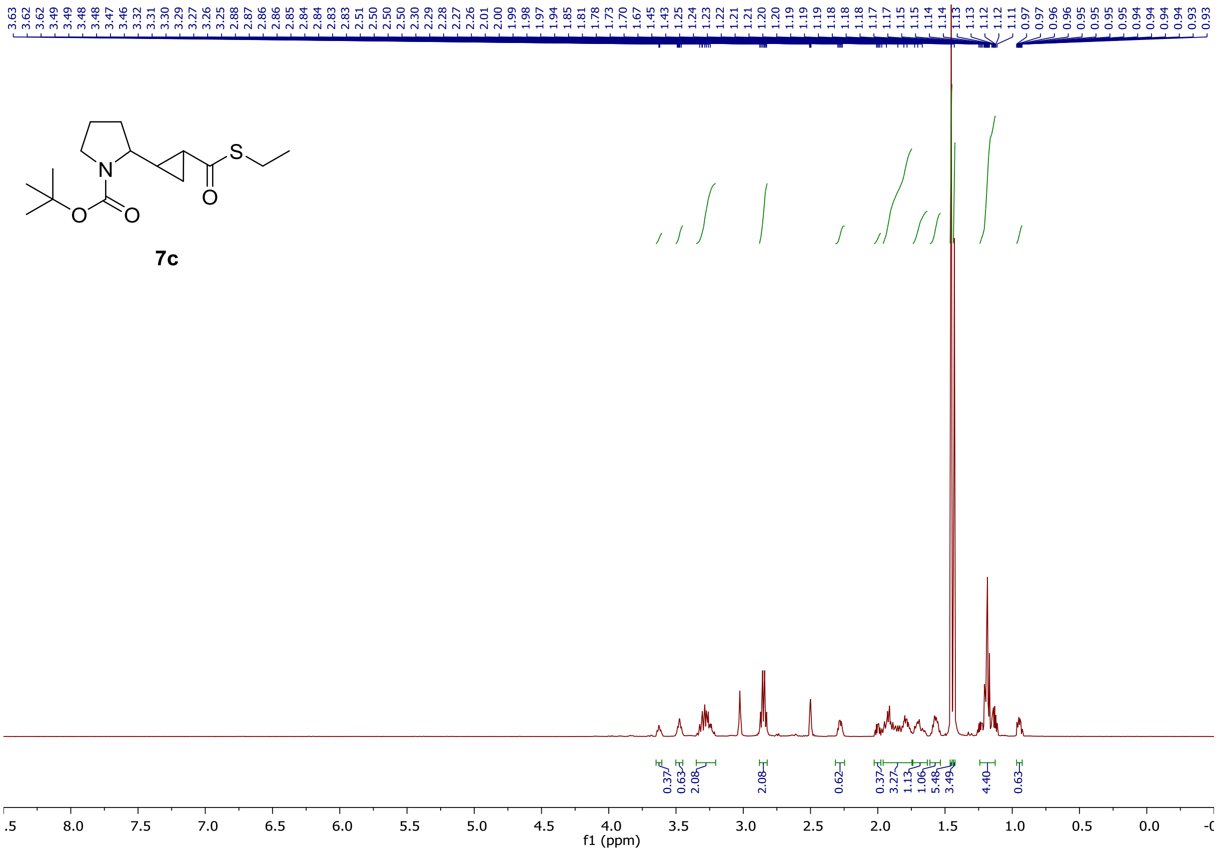

^{13}C NMR (126 MHz, DMSO-d_6, 100 ºC)

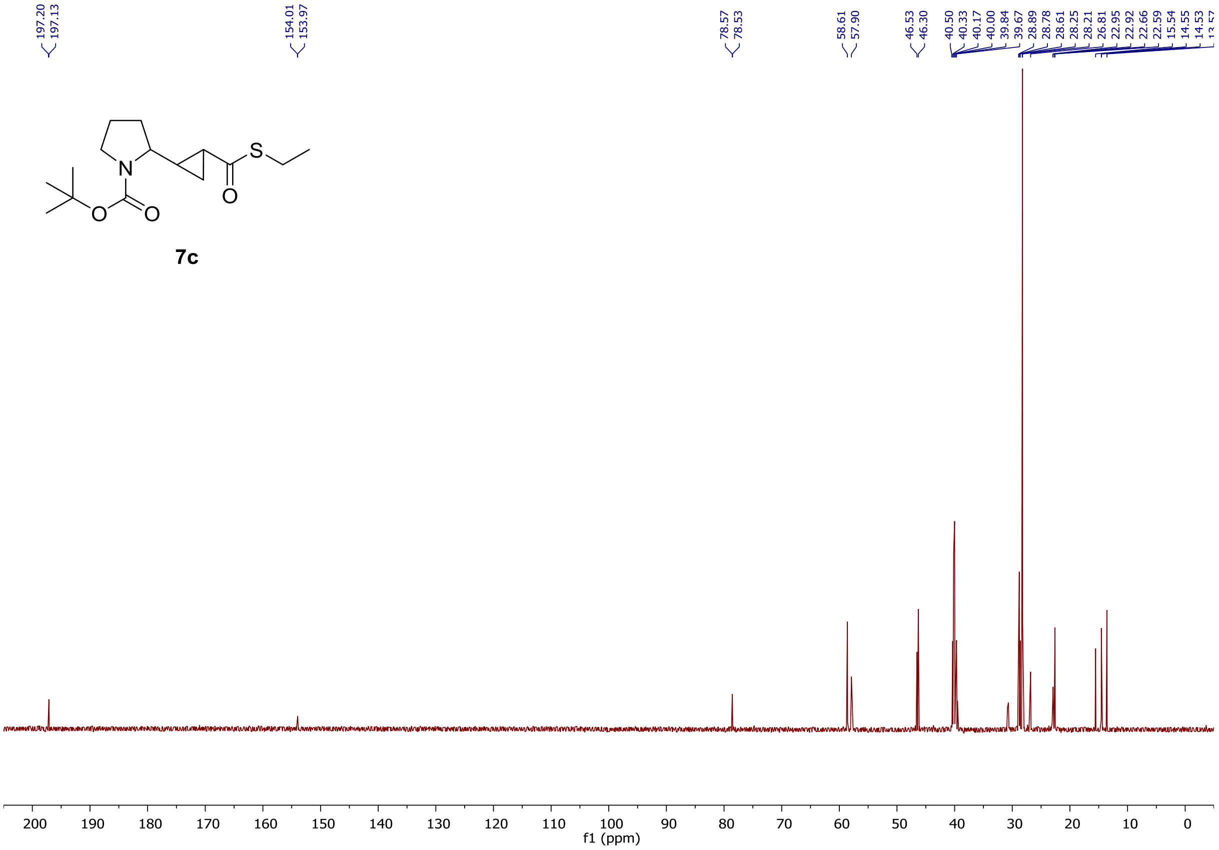

^{1}H NMR (500 MHz, DMSO-d_6, 100 ºC)

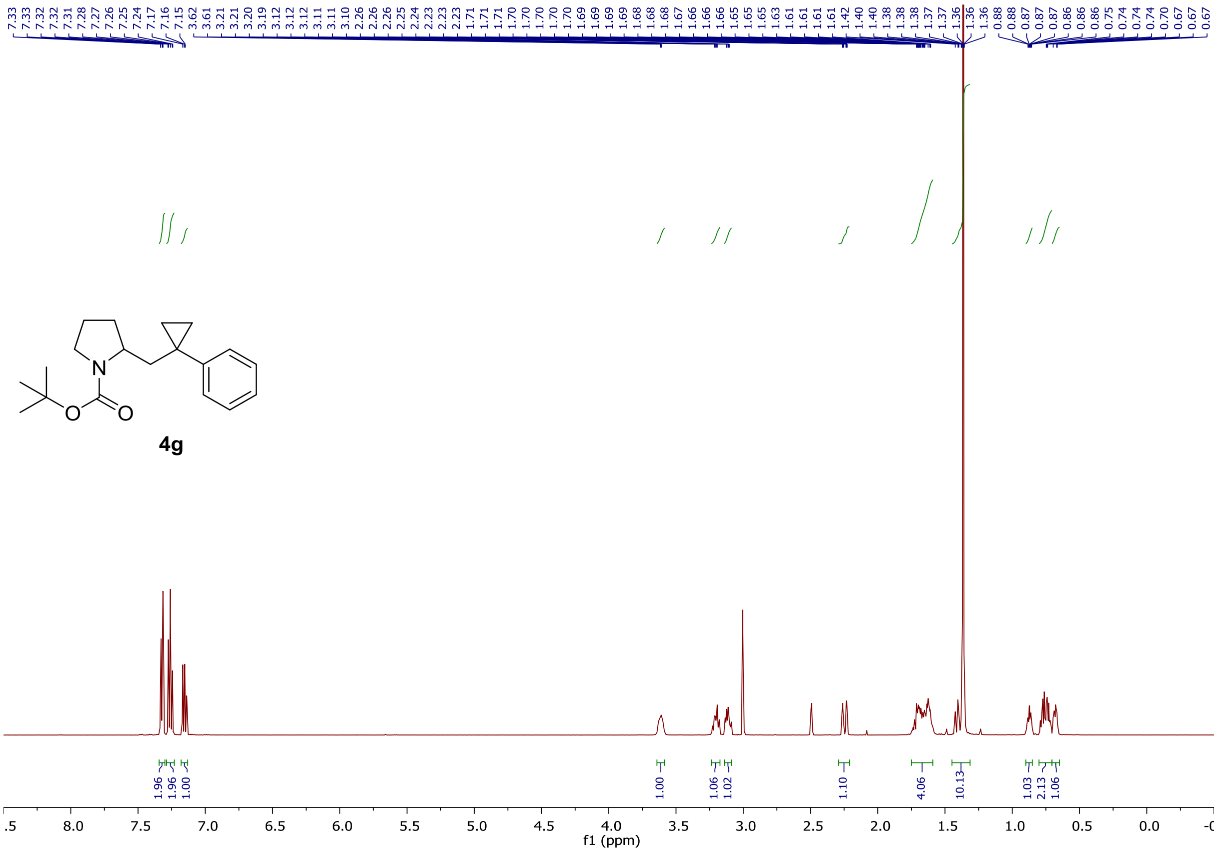

^{13}C NMR (126 MHz, DMSO-d_6, 100 ºC)

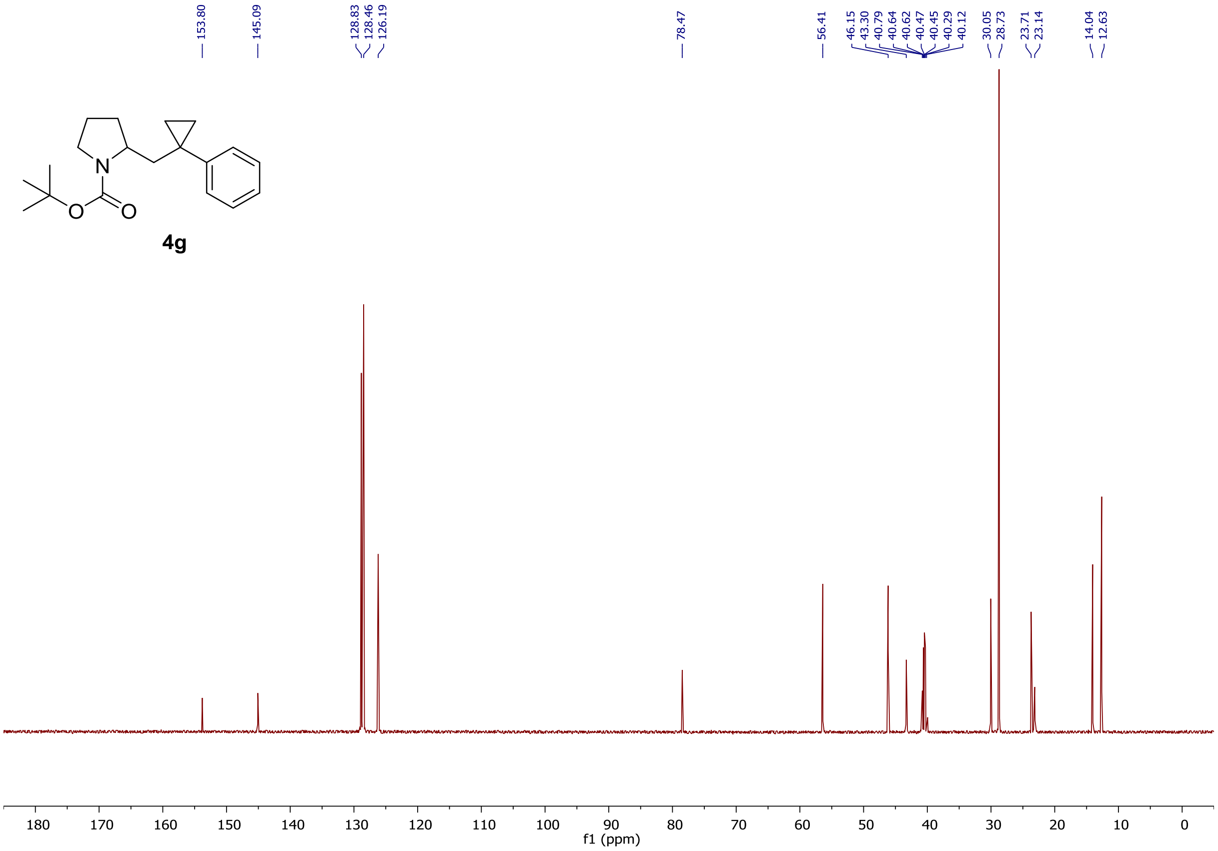

^{1}H NMR (500 MHz, DMSO-d_6, 100 ºC)

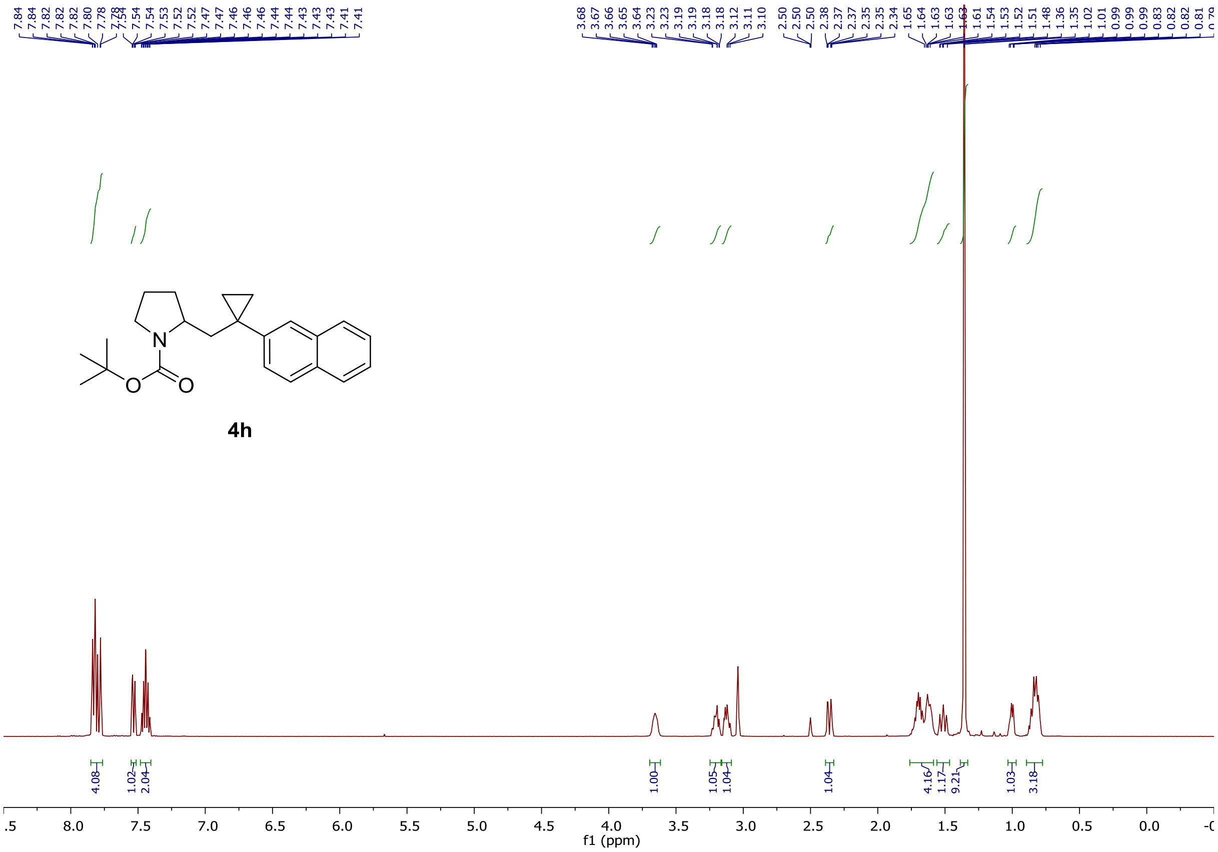

^{13}C NMR (126 MHz, DMSO-d_6, 100 ºC)

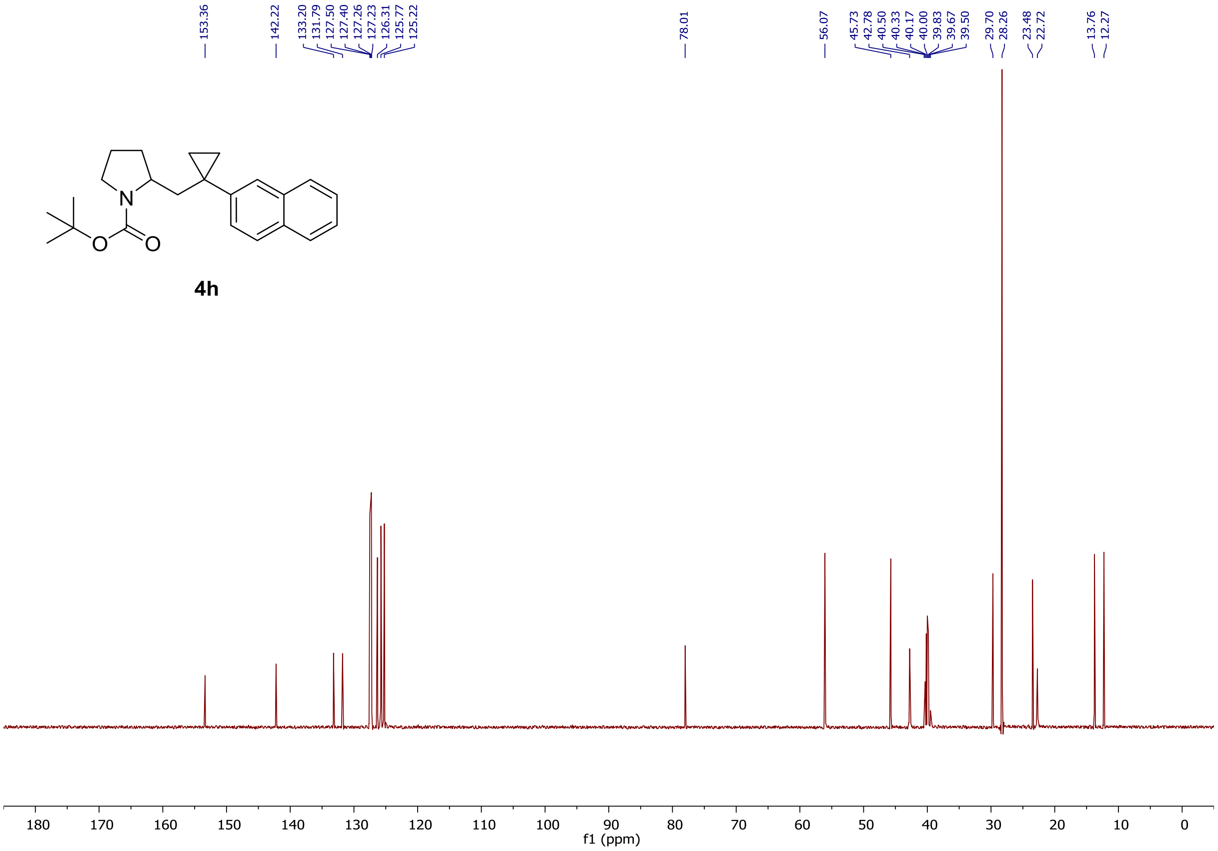

^{1}H NMR (500 MHz, DMSO-d_6, 100 °C)

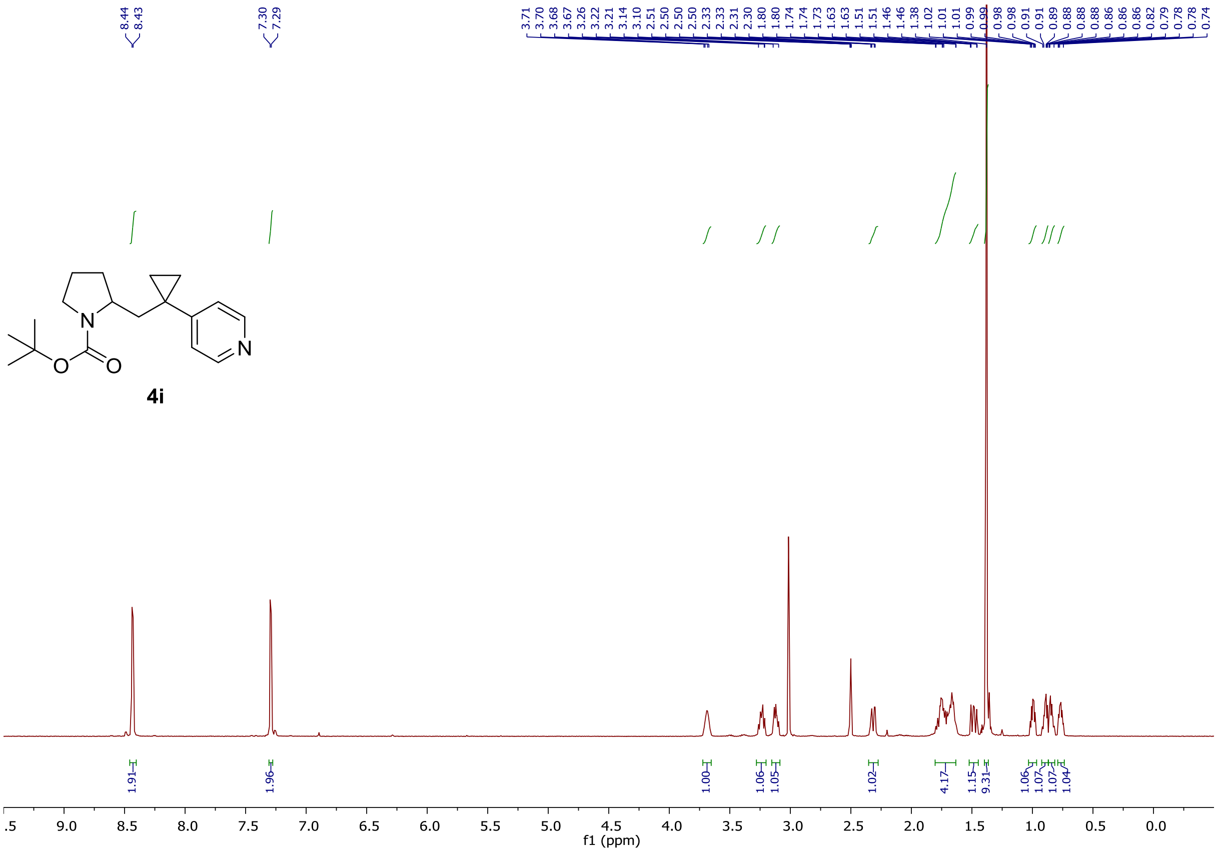

^{13}C NMR (126 MHz, DMSO-d_6, 100 °C)

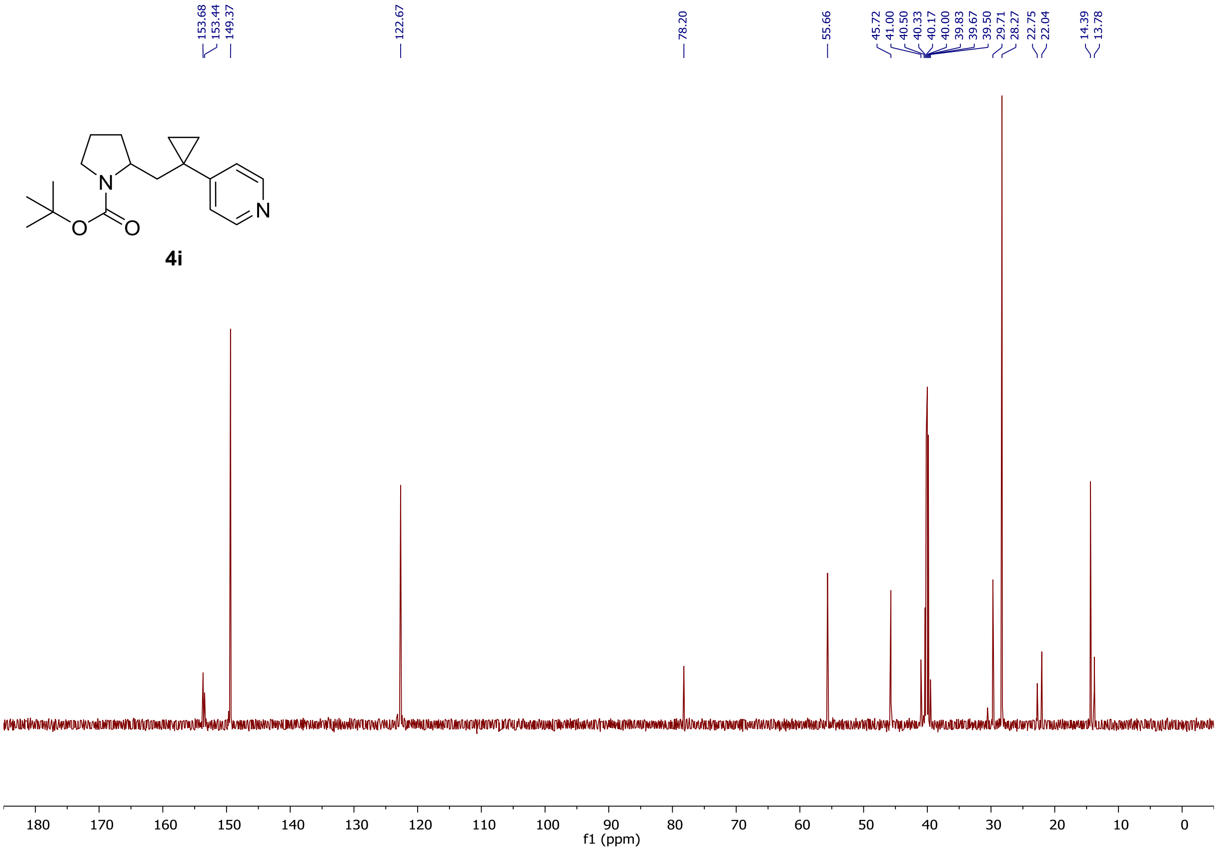

^{1}H NMR (500 MHz, DMSO-d_6, 100 ºC)

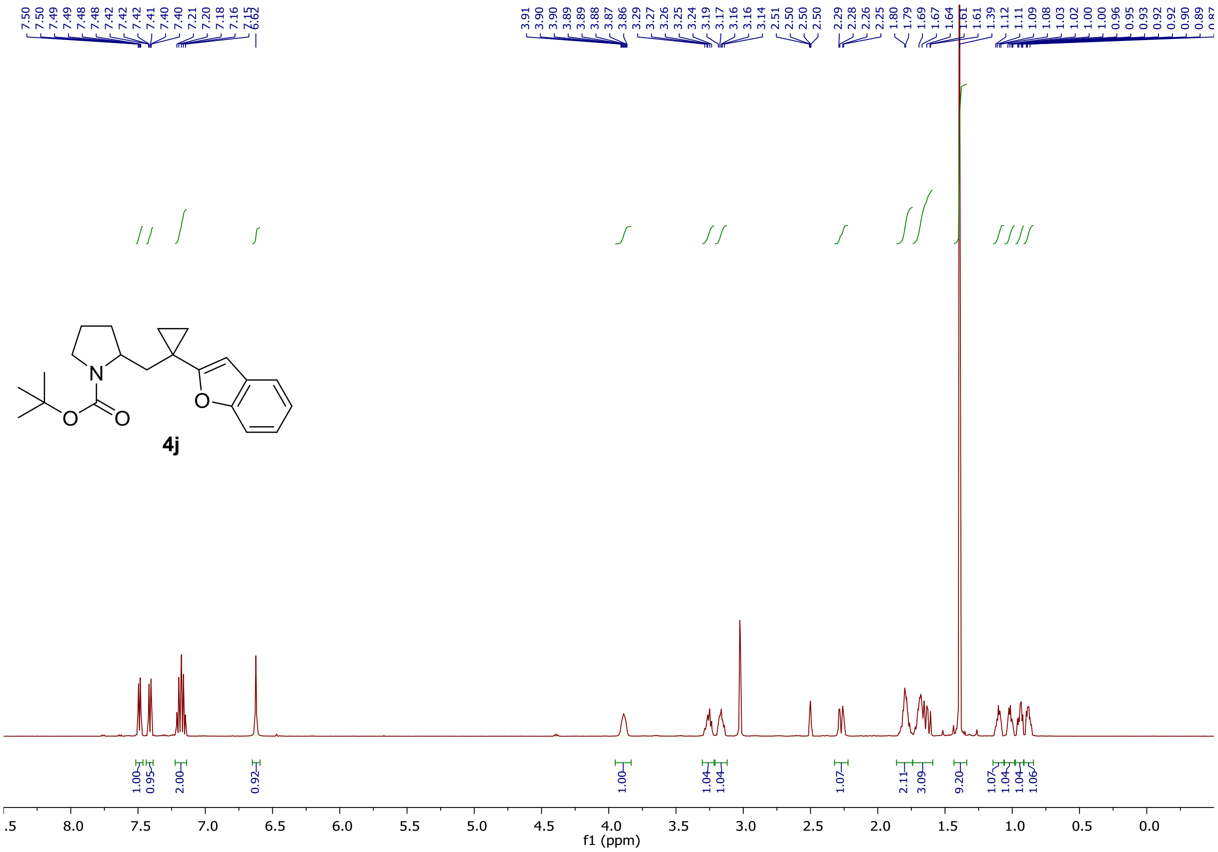

4j

^{13}C NMR (126 MHz, DMSO-d_6, 100 ºC)

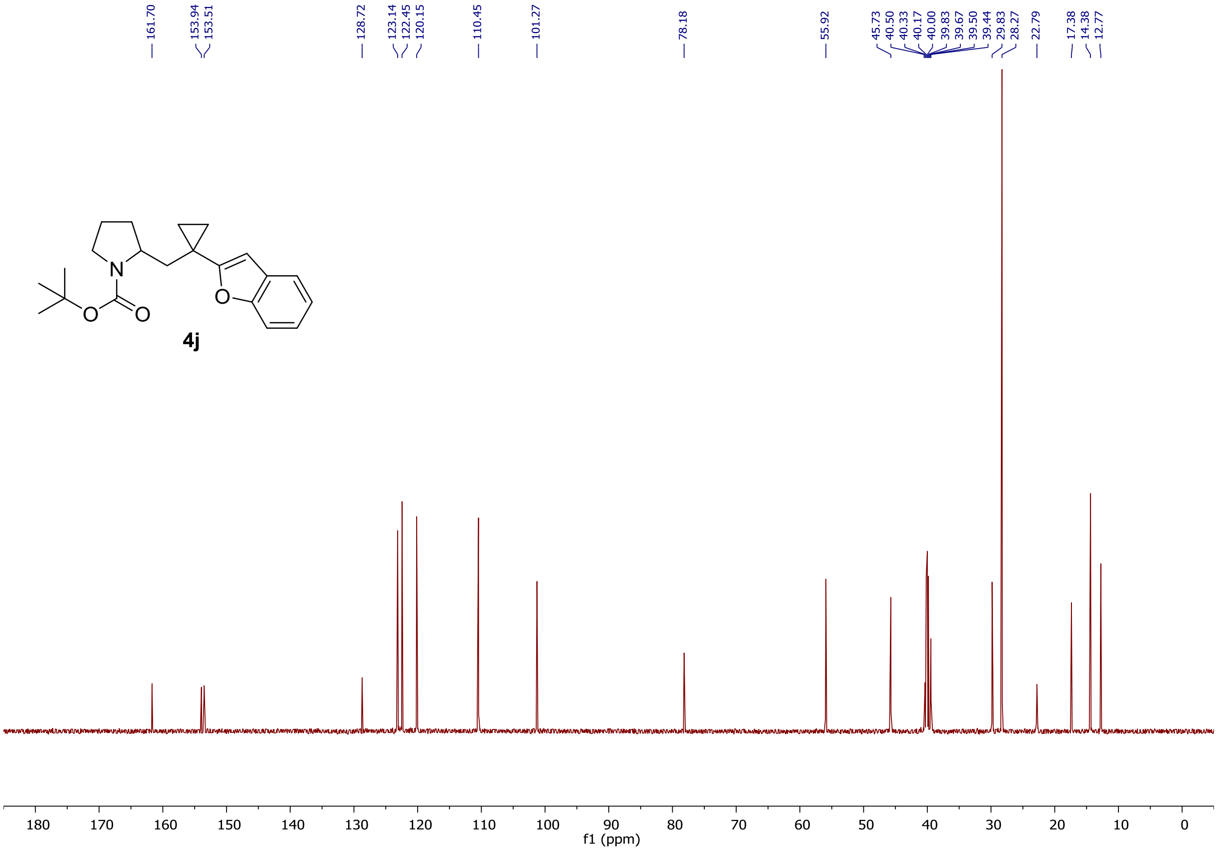

4j

^{1}H NMR (400 MHz, $CDCl_3$)

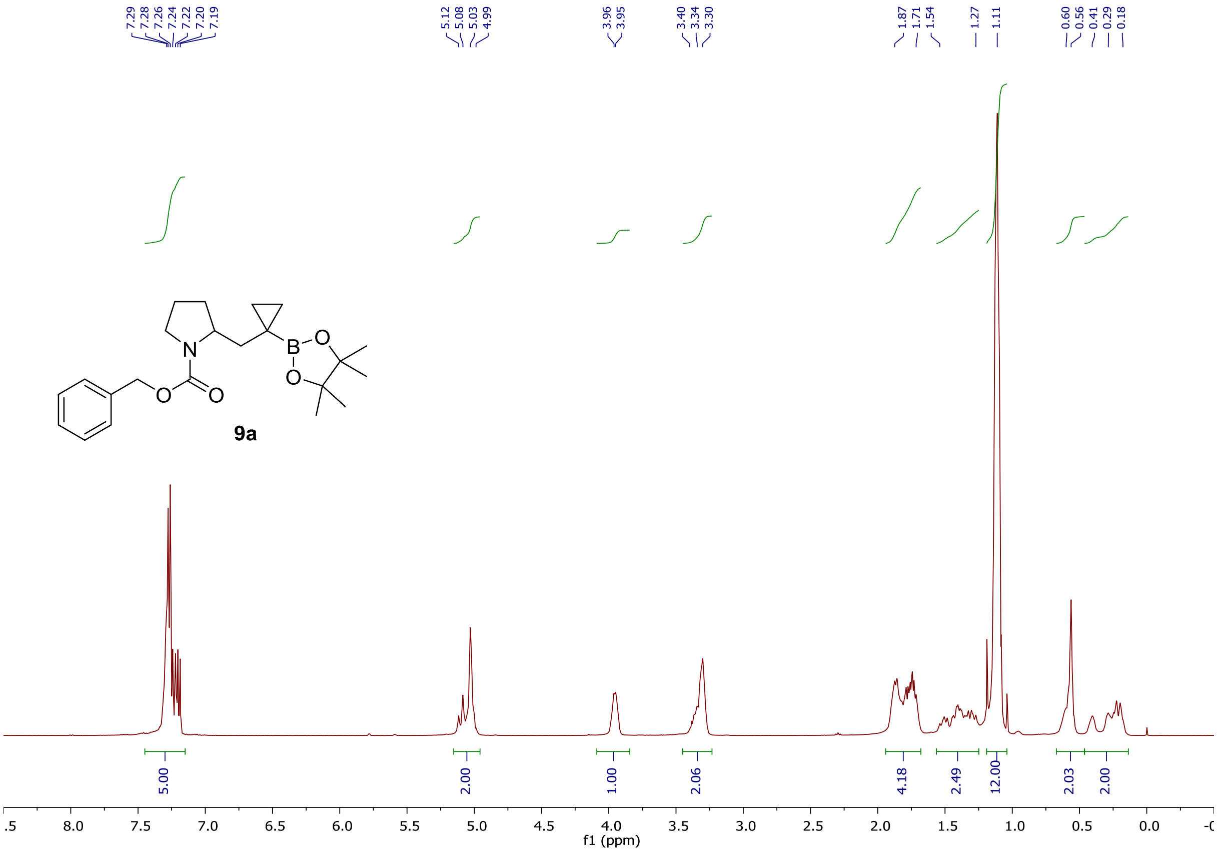

^{13}C NMR (101 MHz, $CDCl_3$)

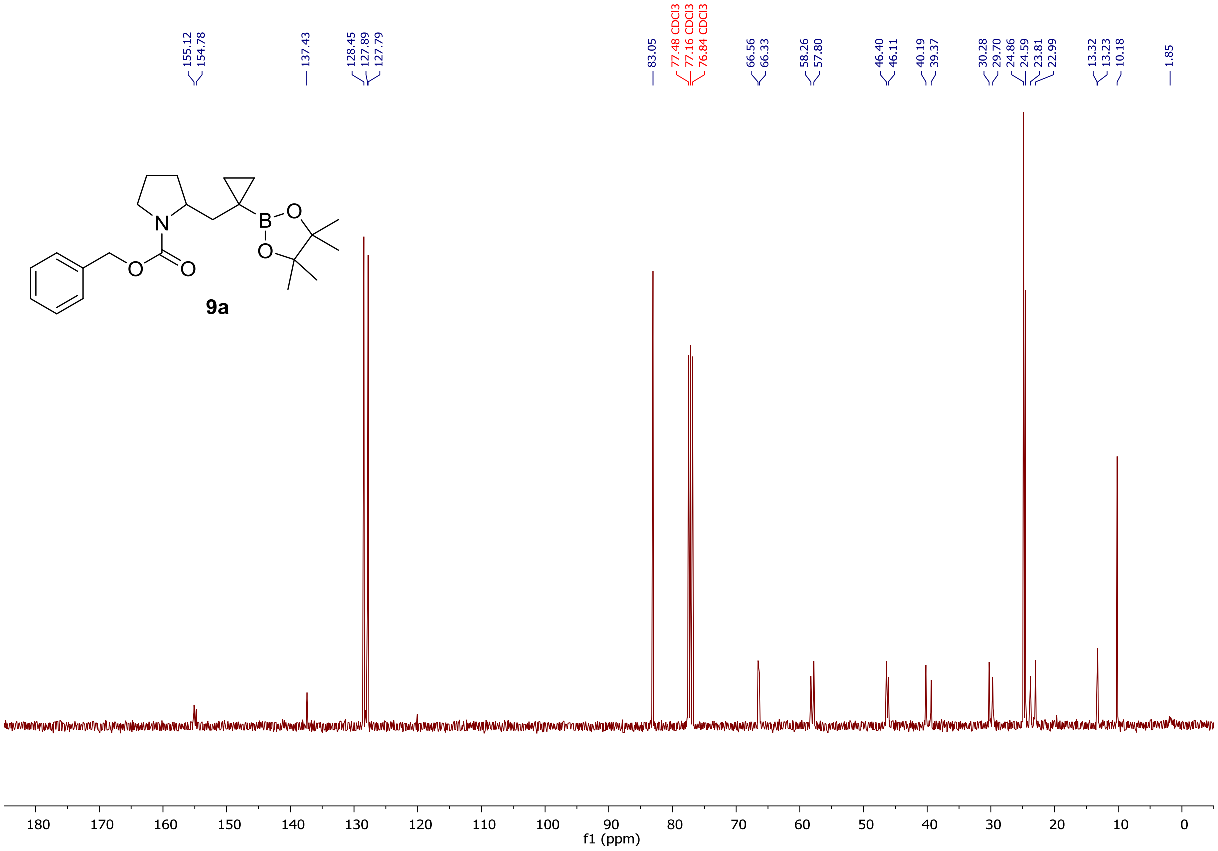

^{1}H NMR (400 MHz, $CDCl_3$)

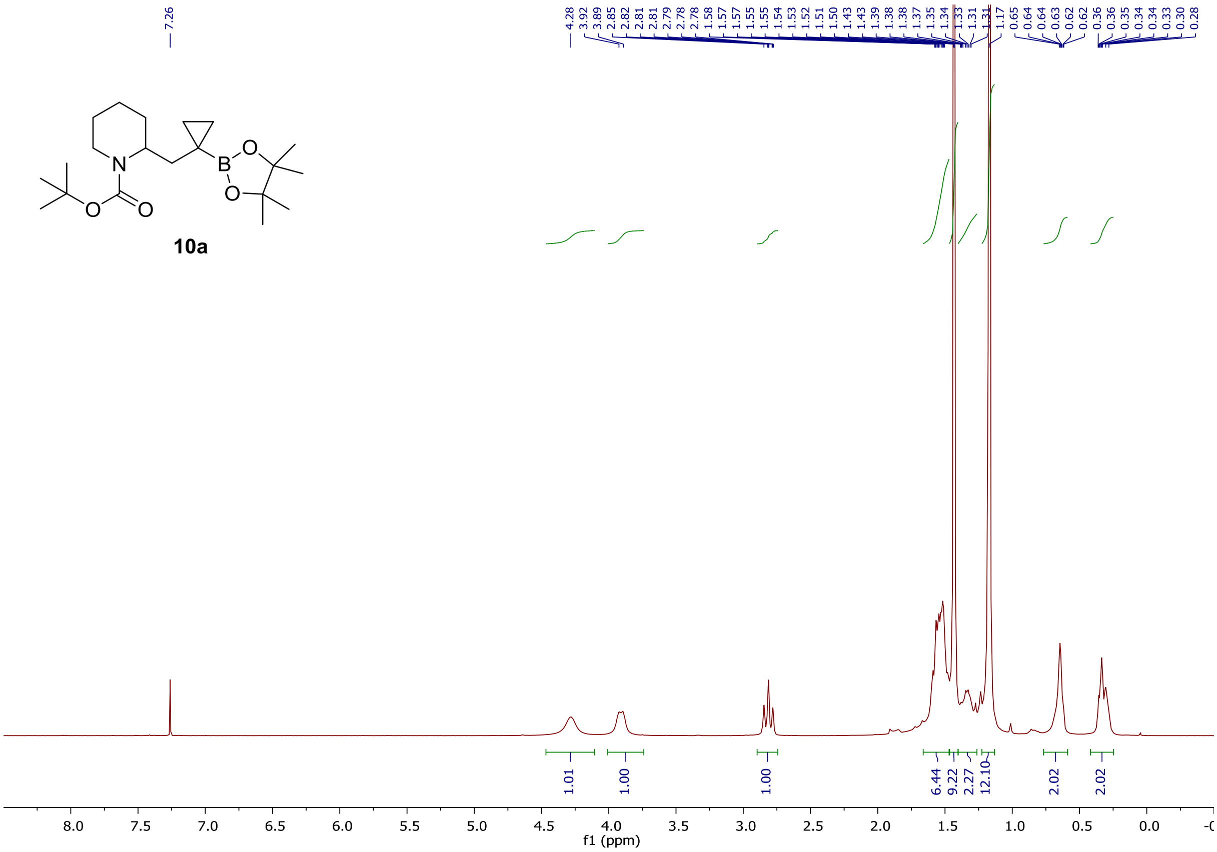

^{13}C NMR (101 MHz, $CDCl_3$)

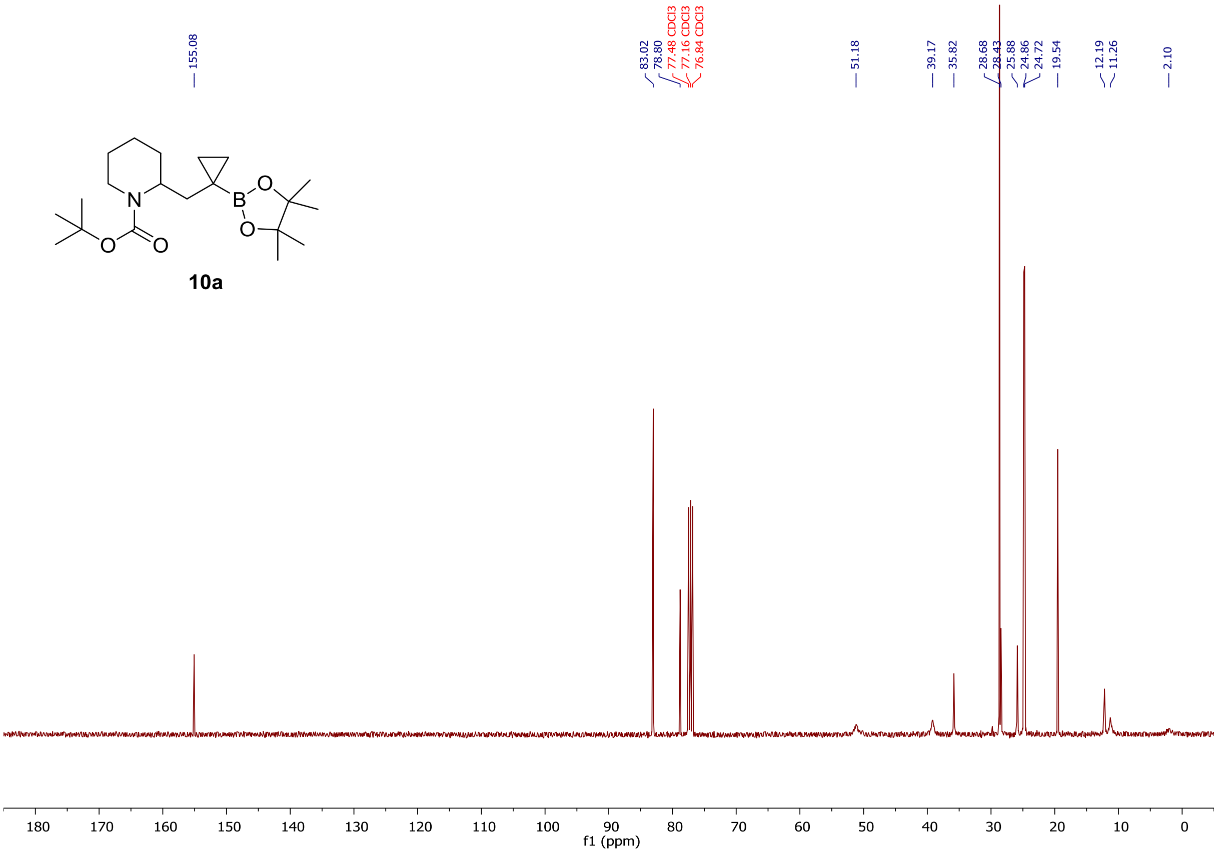

^{1}H NMR (400 MHz, $CDCl_3$)

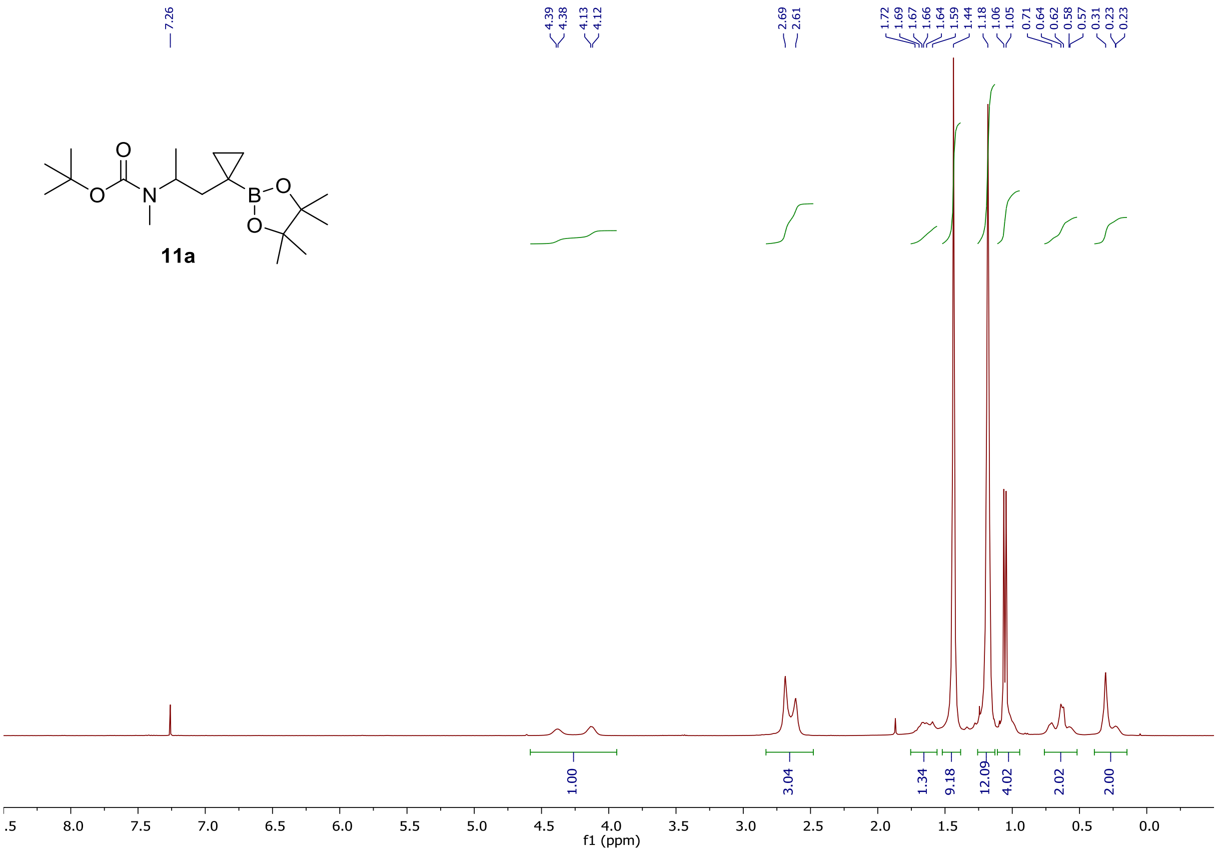

^{13}C NMR (101 MHz, $CDCl_3$)

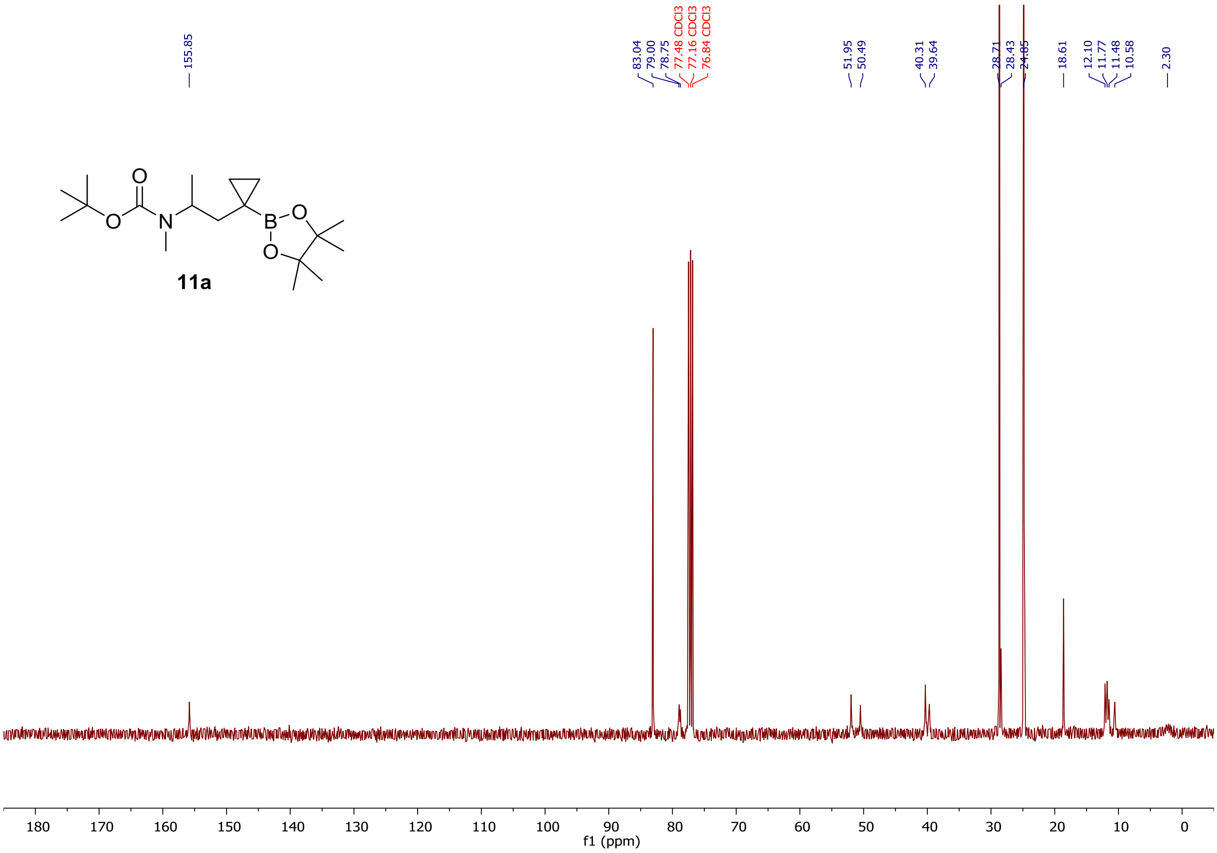

^{1}H NMR (400 MHz, $CDCl_3$)

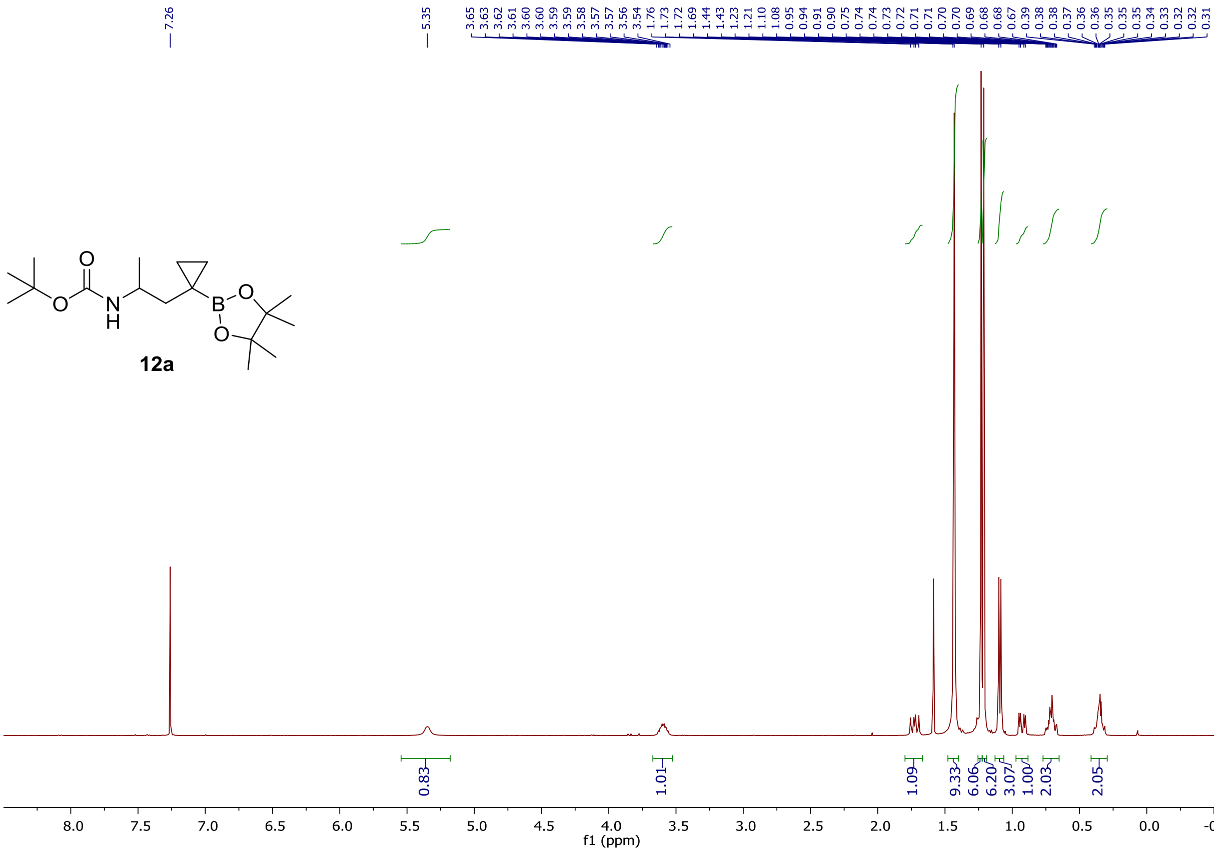

^{13}C NMR (101 MHz, $CDCl_3$)

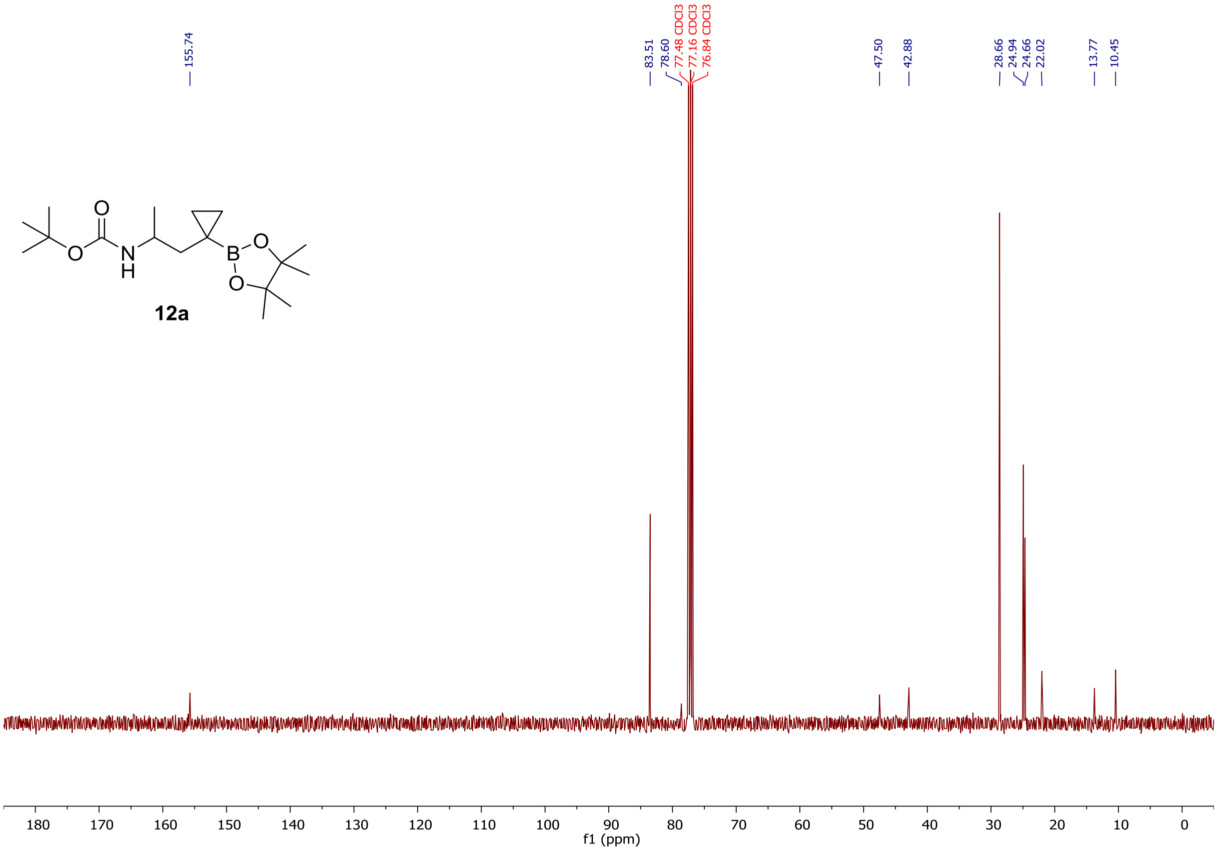

^{1}H NMR (400 MHz, $CDCl_3$)

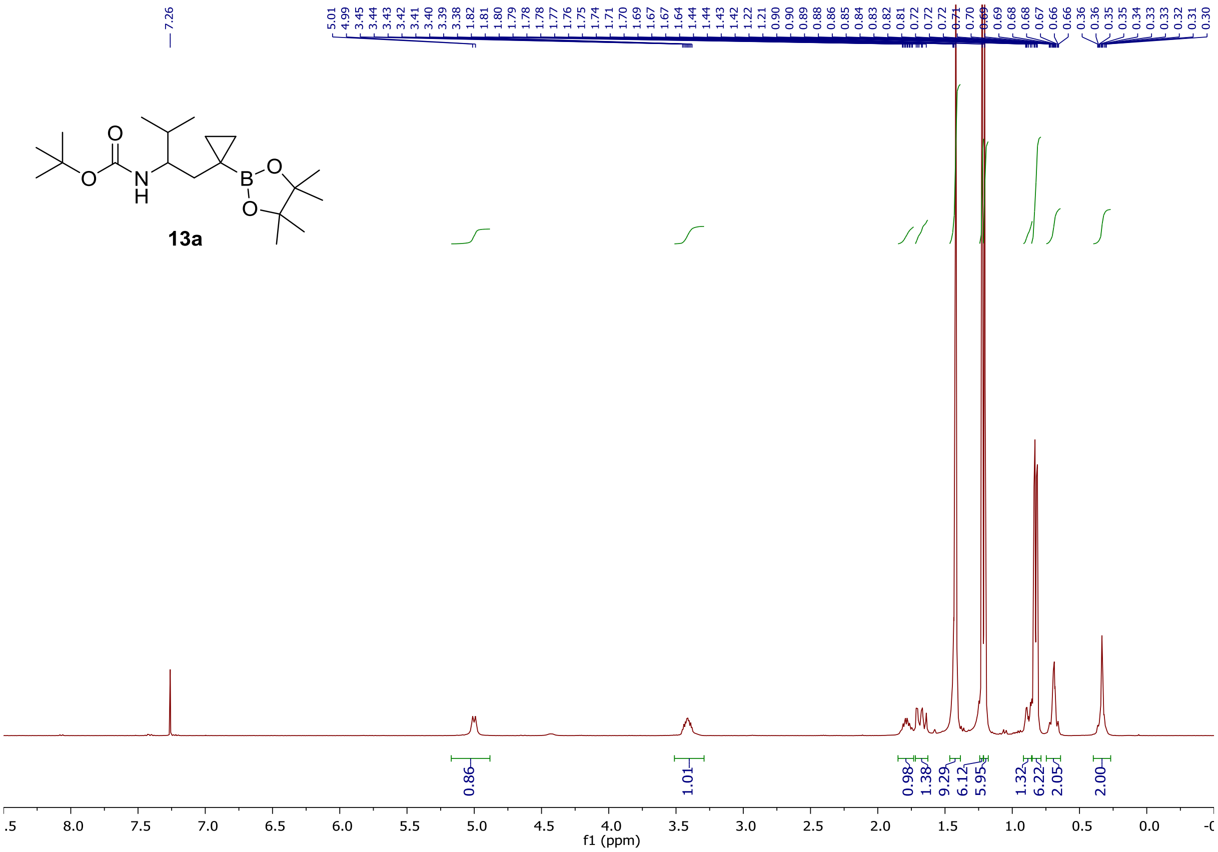

^{13}C NMR (101 MHz, $CDCl_3$)

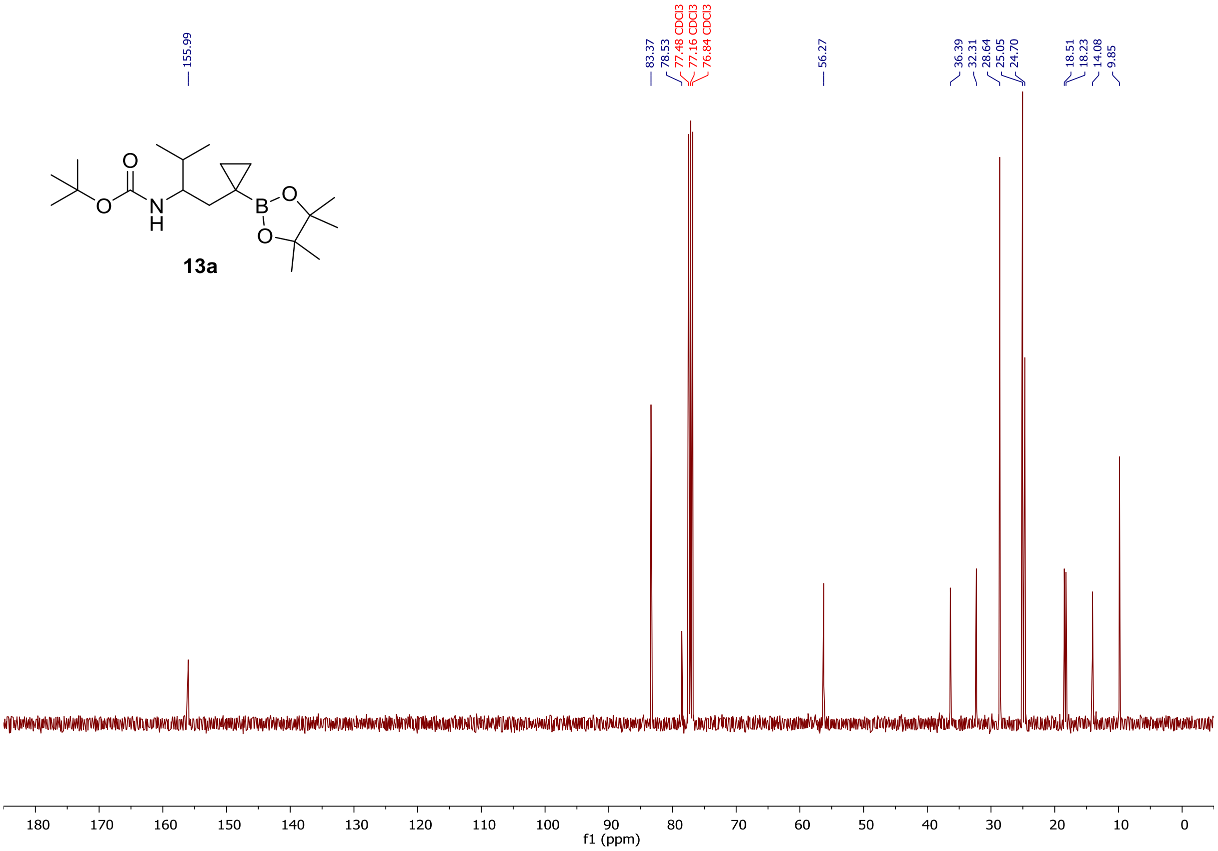

^{1}H NMR (400 MHz, $CDCl_3$)

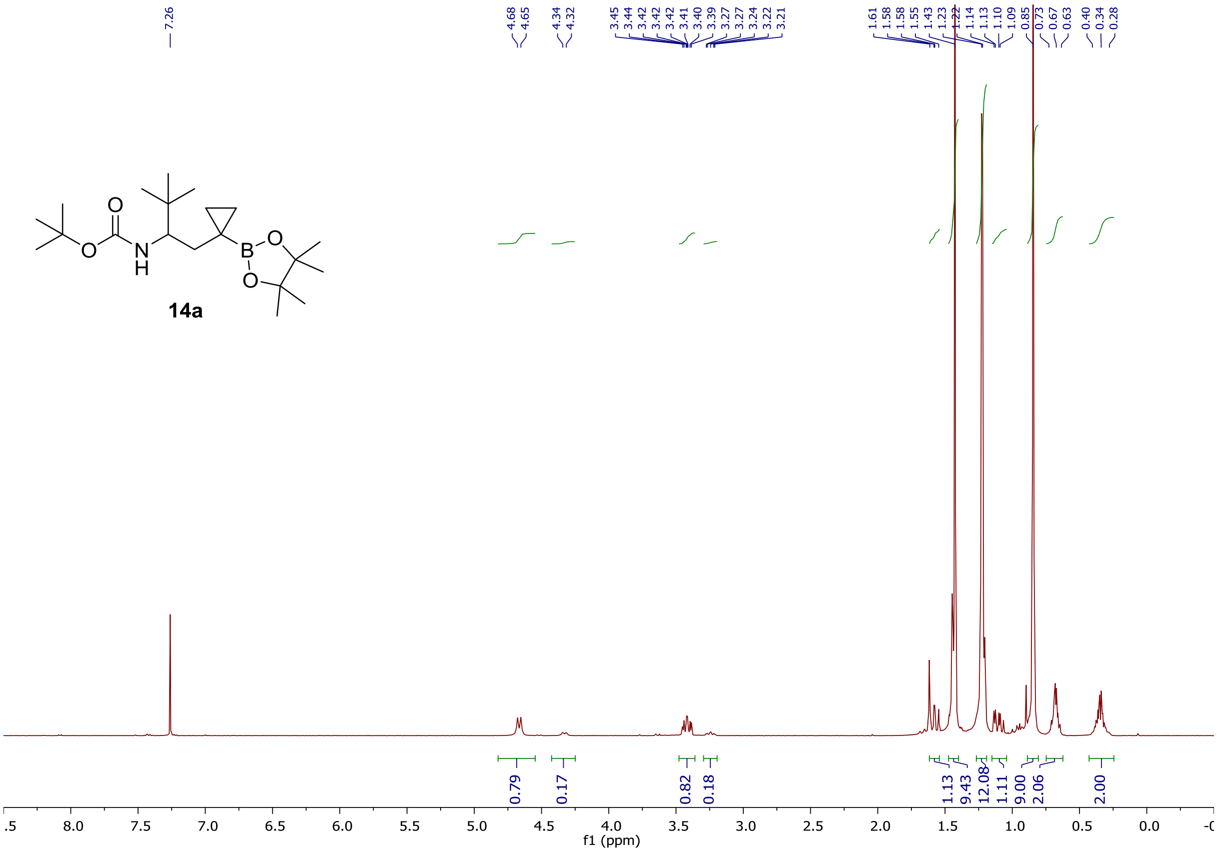

^{13}C NMR (101 MHz, $CDCl_3$)

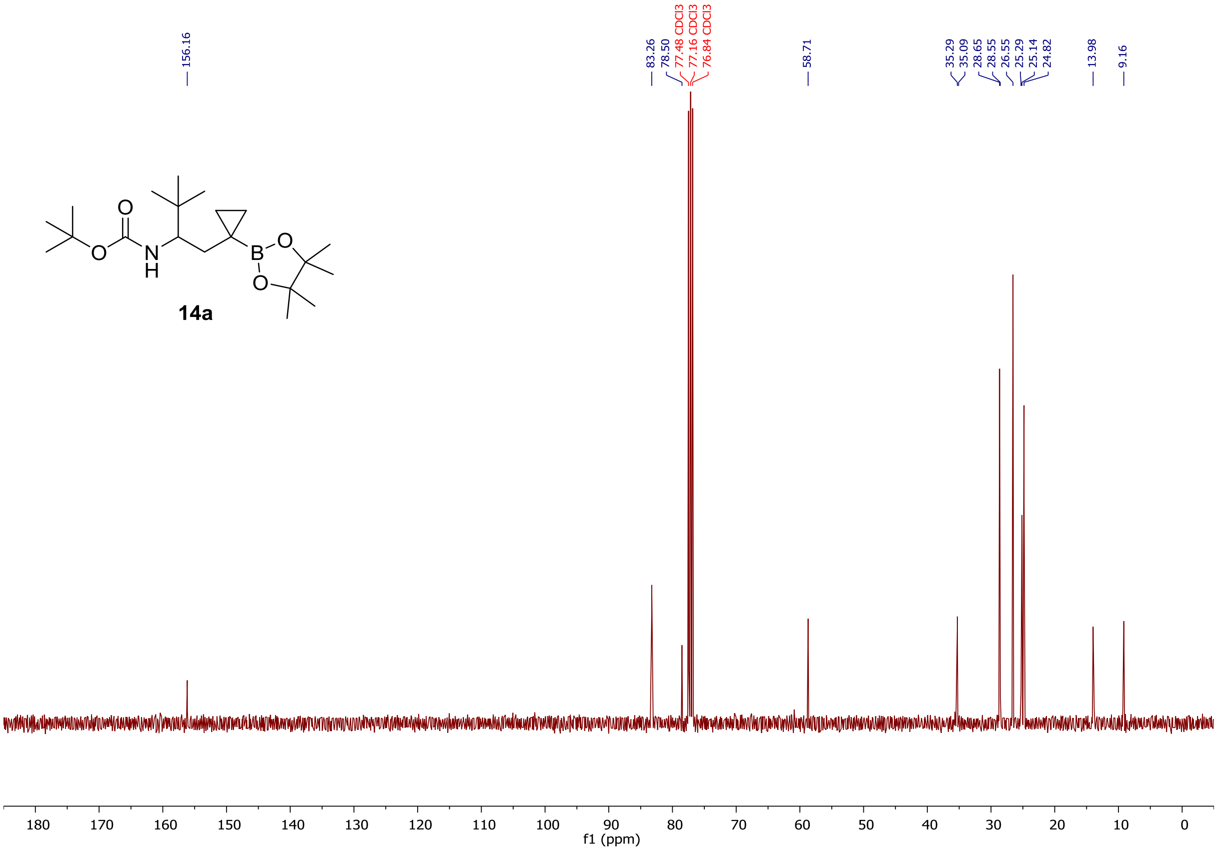

^{1}H NMR (400 MHz, $CDCl_3$)

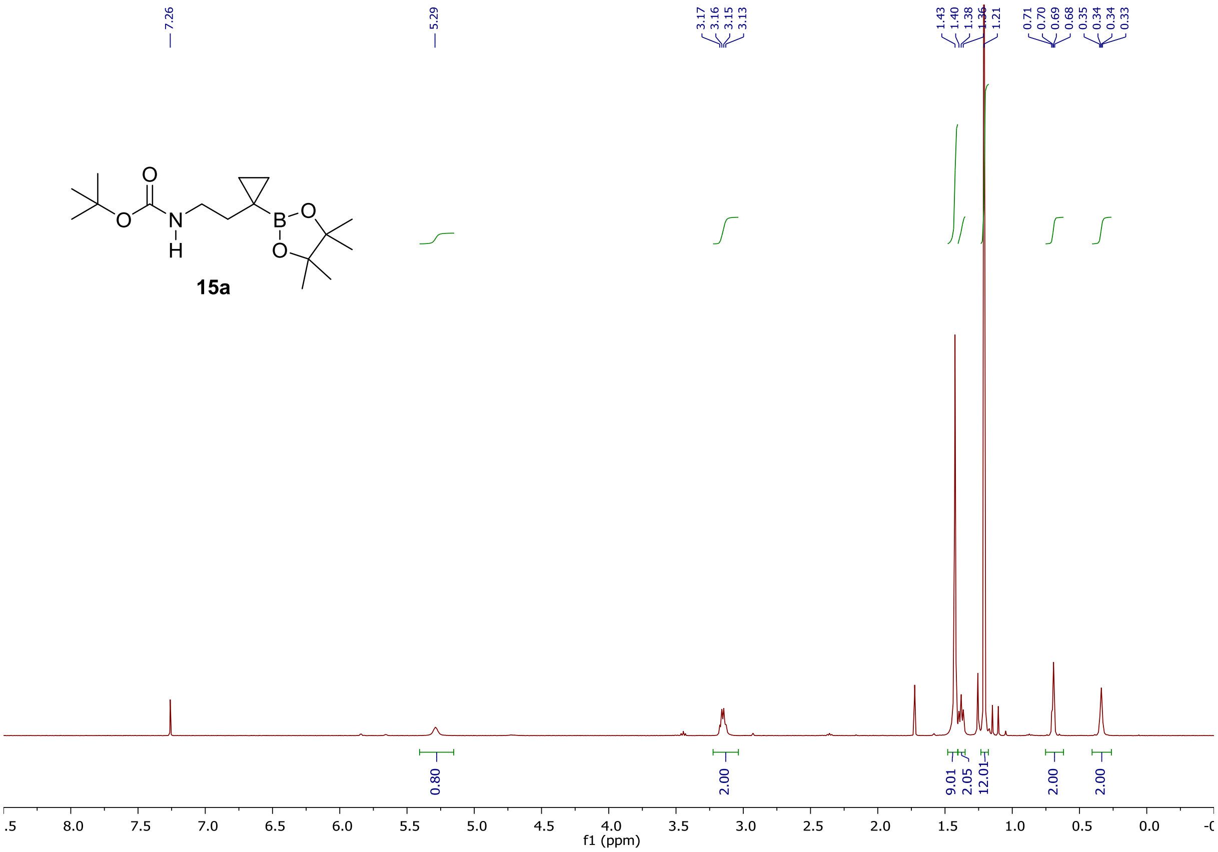

^{13}C NMR (101 MHz, $CDCl_3$)

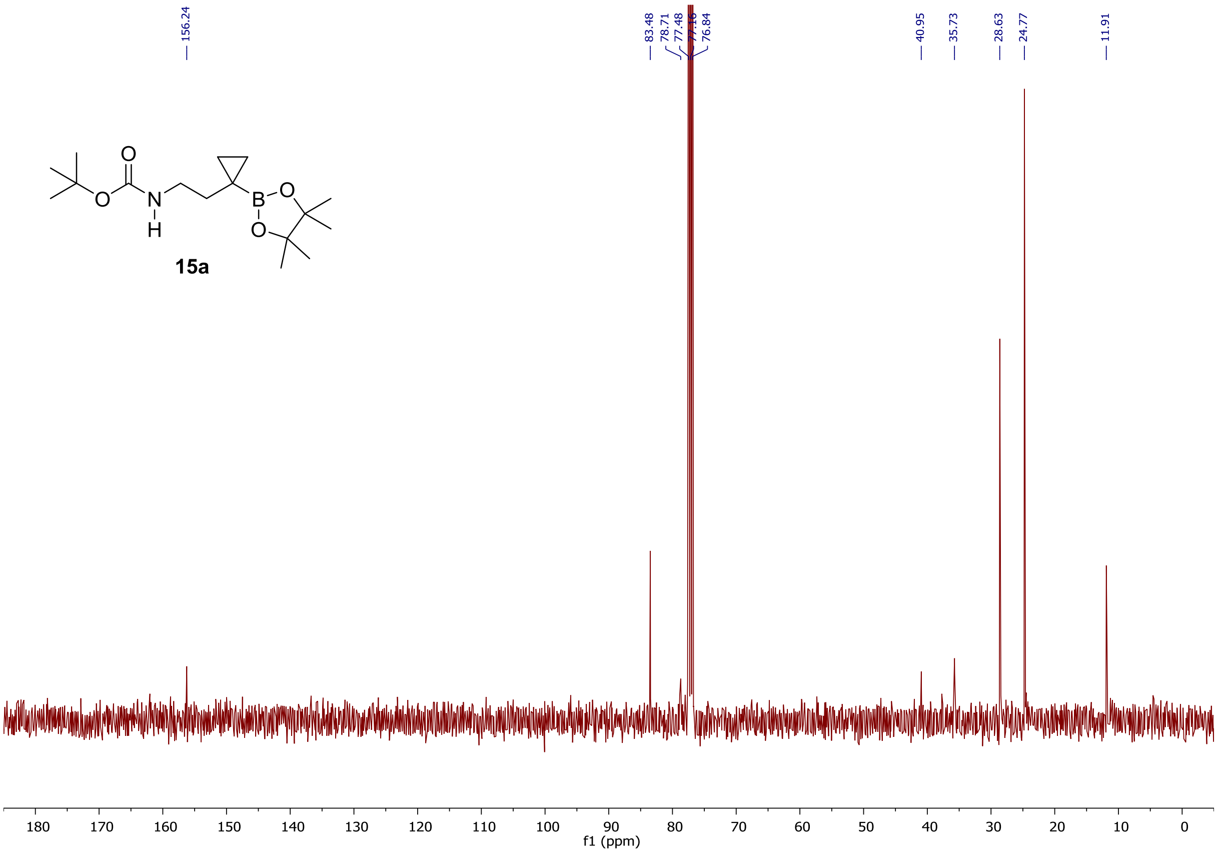

^{1}H NMR (400 MHz, DMSO-d_6)

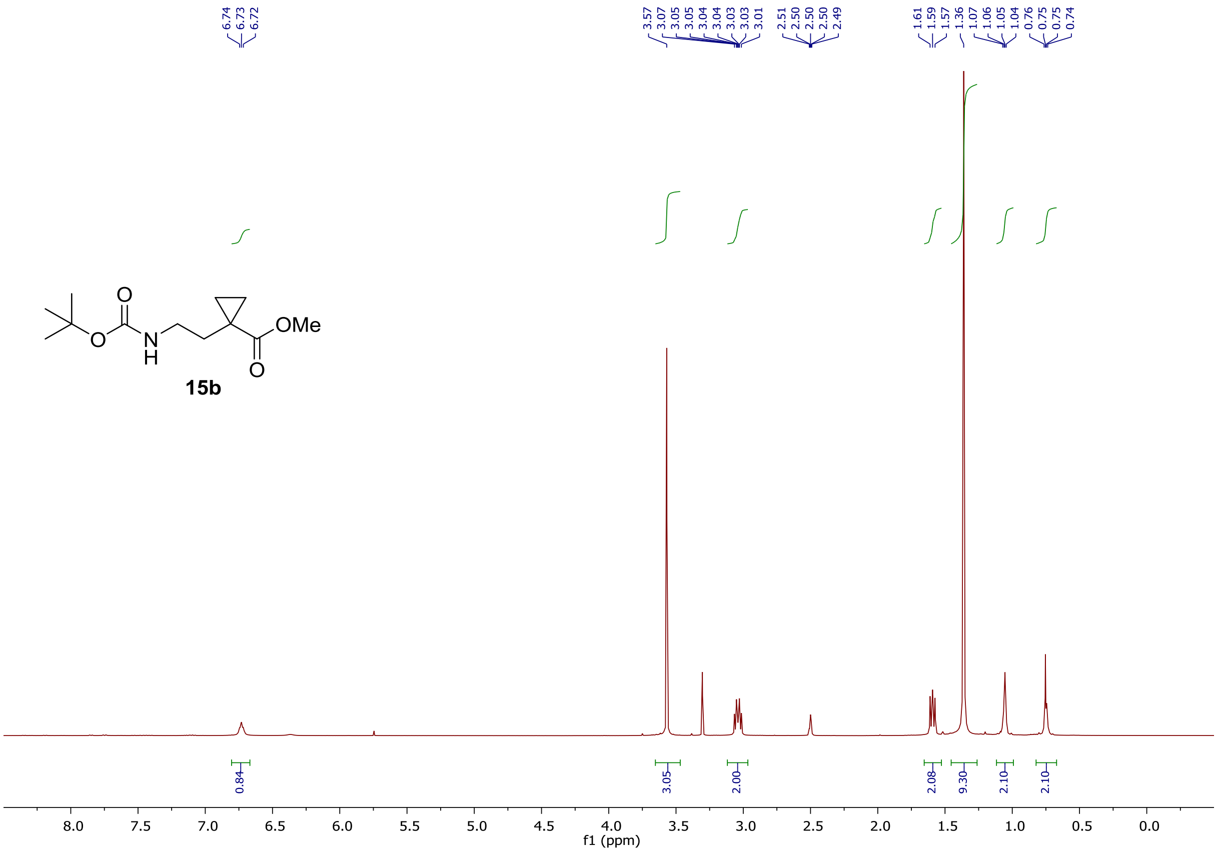

^{13}C NMR (101 MHz, DMSO-d_6)

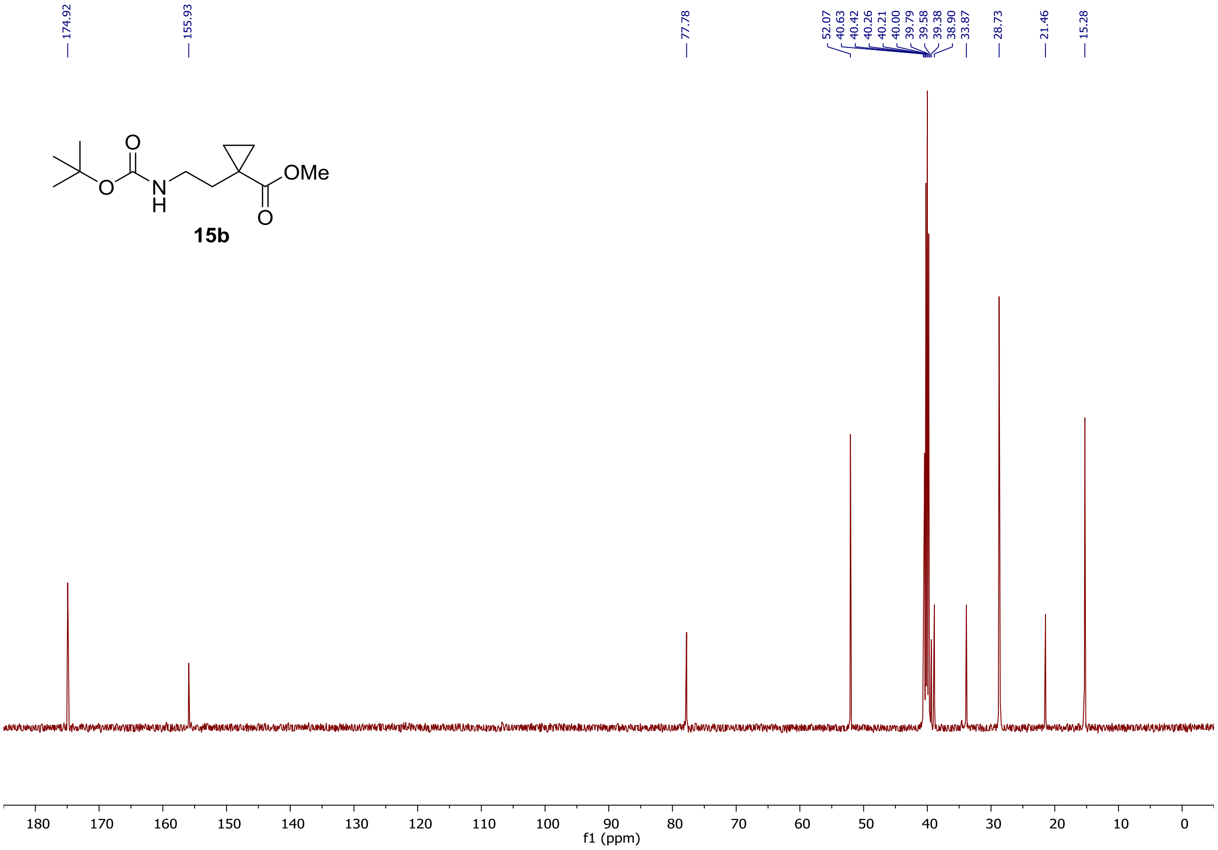

^{1}H NMR (400 MHz, $CDCl_3$)

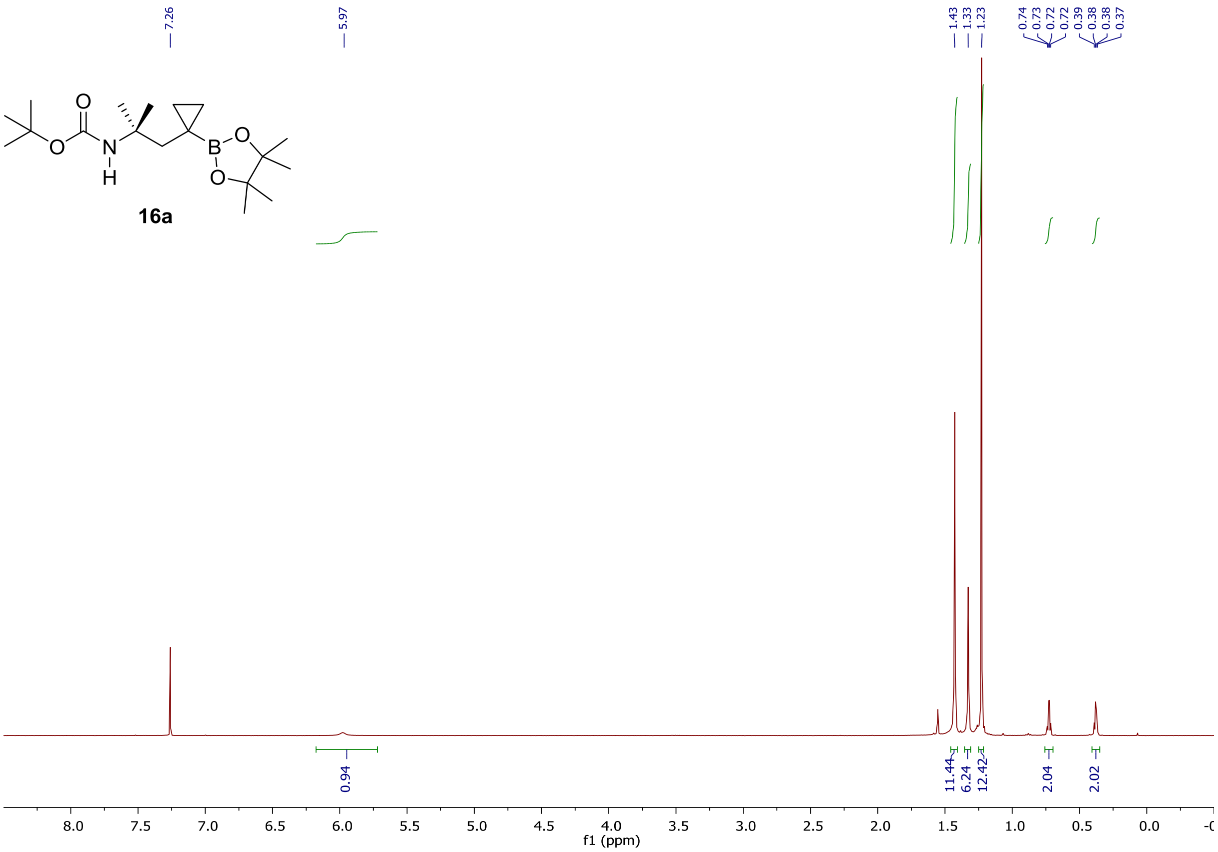

^{13}C NMR (101 MHz, $CDCl_3$)

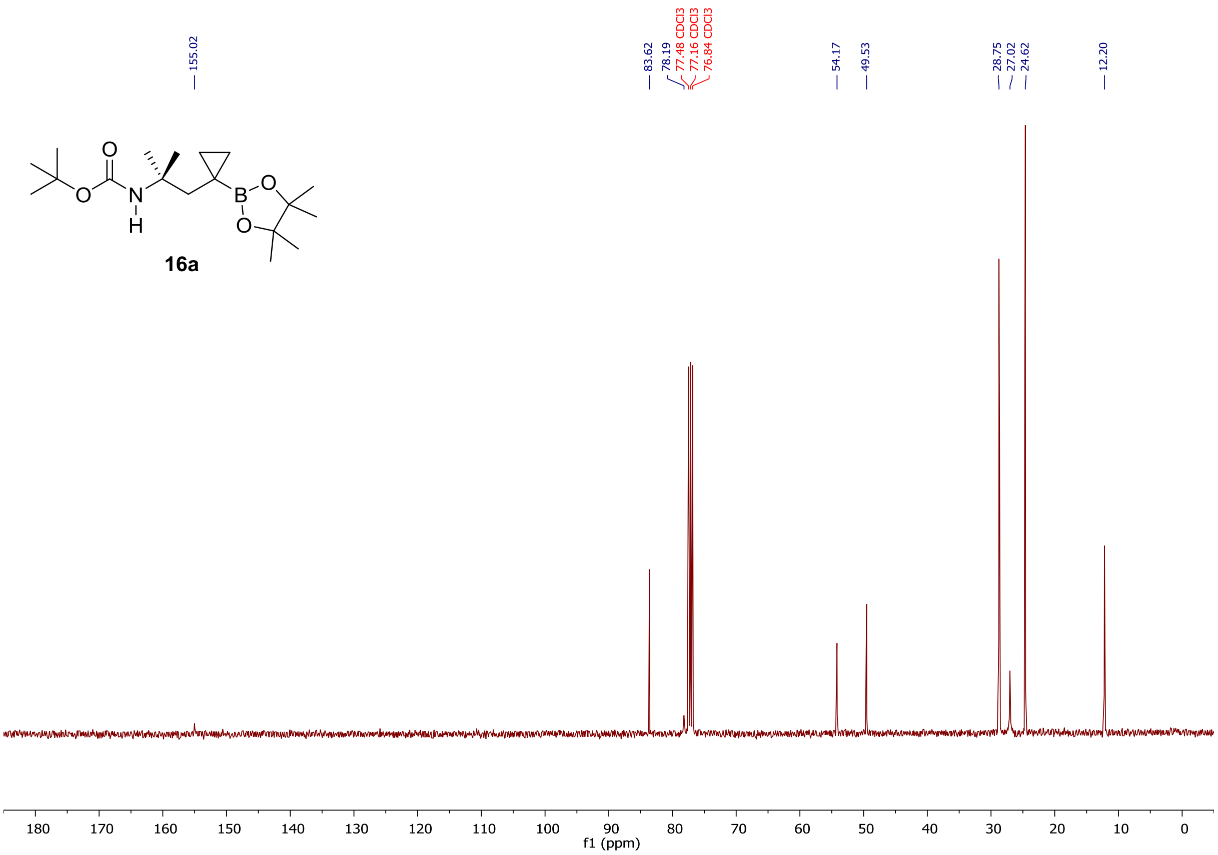

^{1}H NMR (400 MHz, $CDCl_3$)

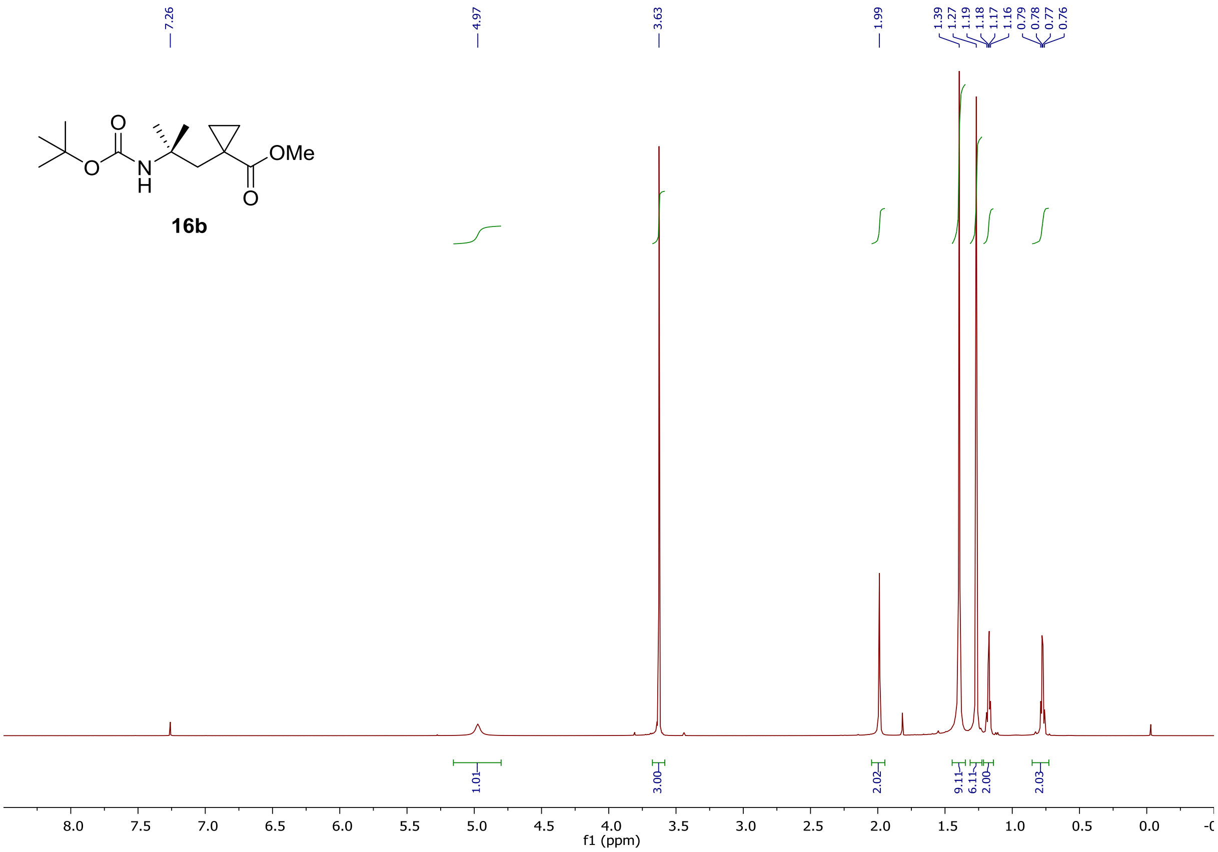

^{13}C NMR (101 MHz, $CDCl_3$)

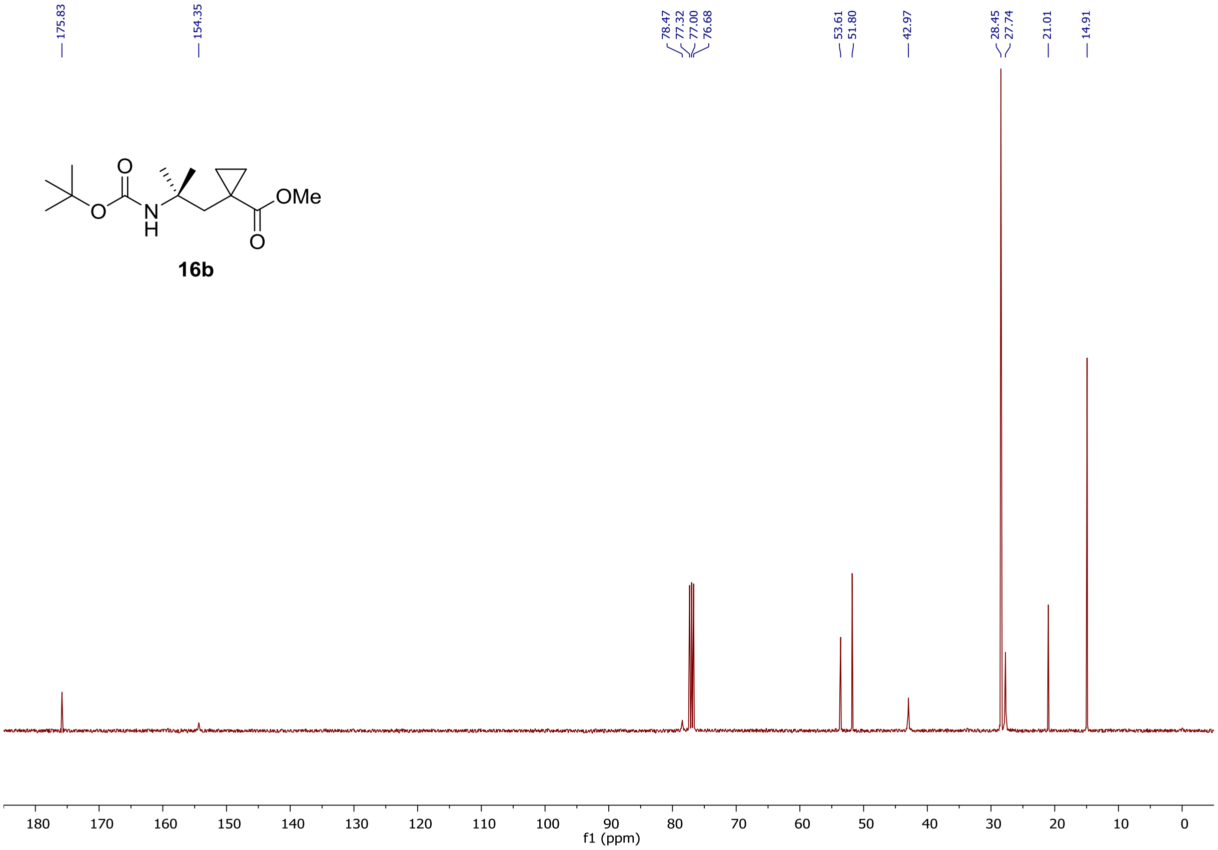

^{1}H NMR (400 MHz, $CDCl_3$)

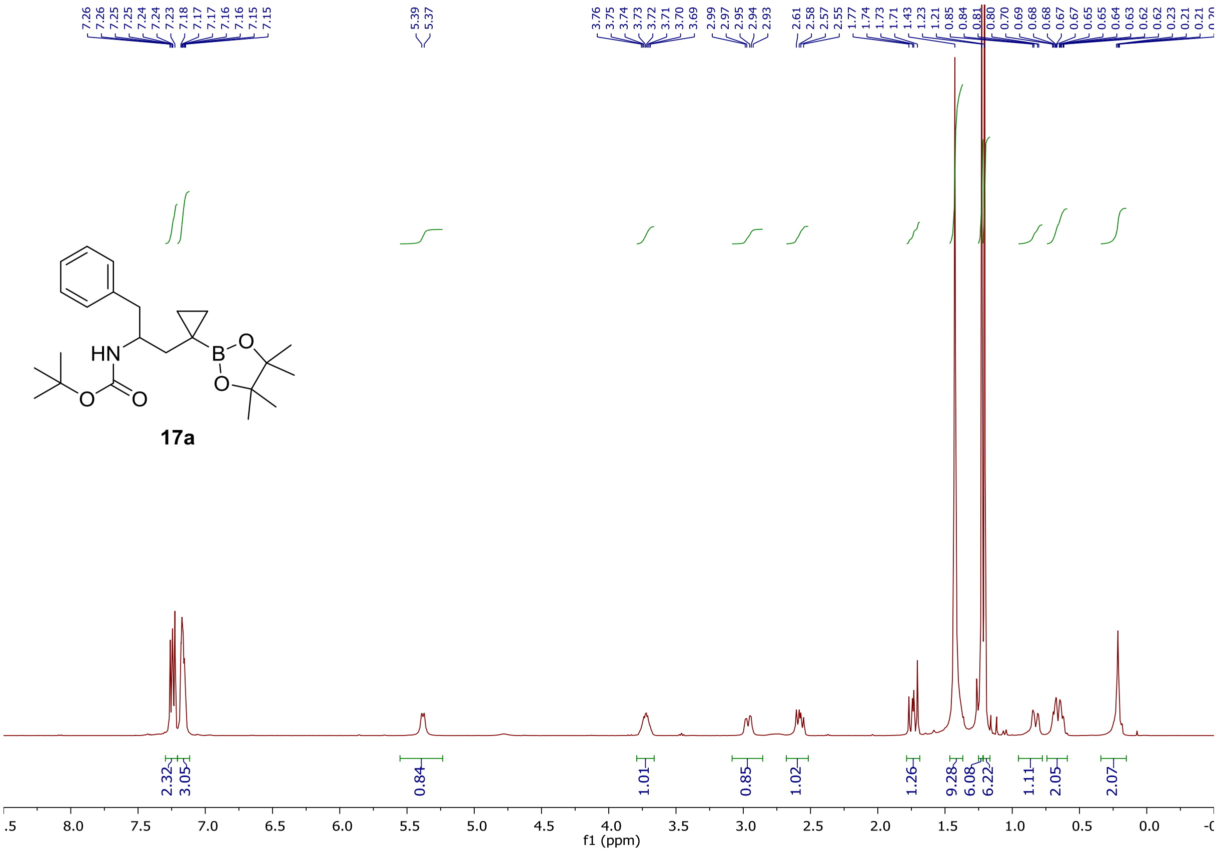

^{13}C NMR (101 MHz, $CDCl_3$)

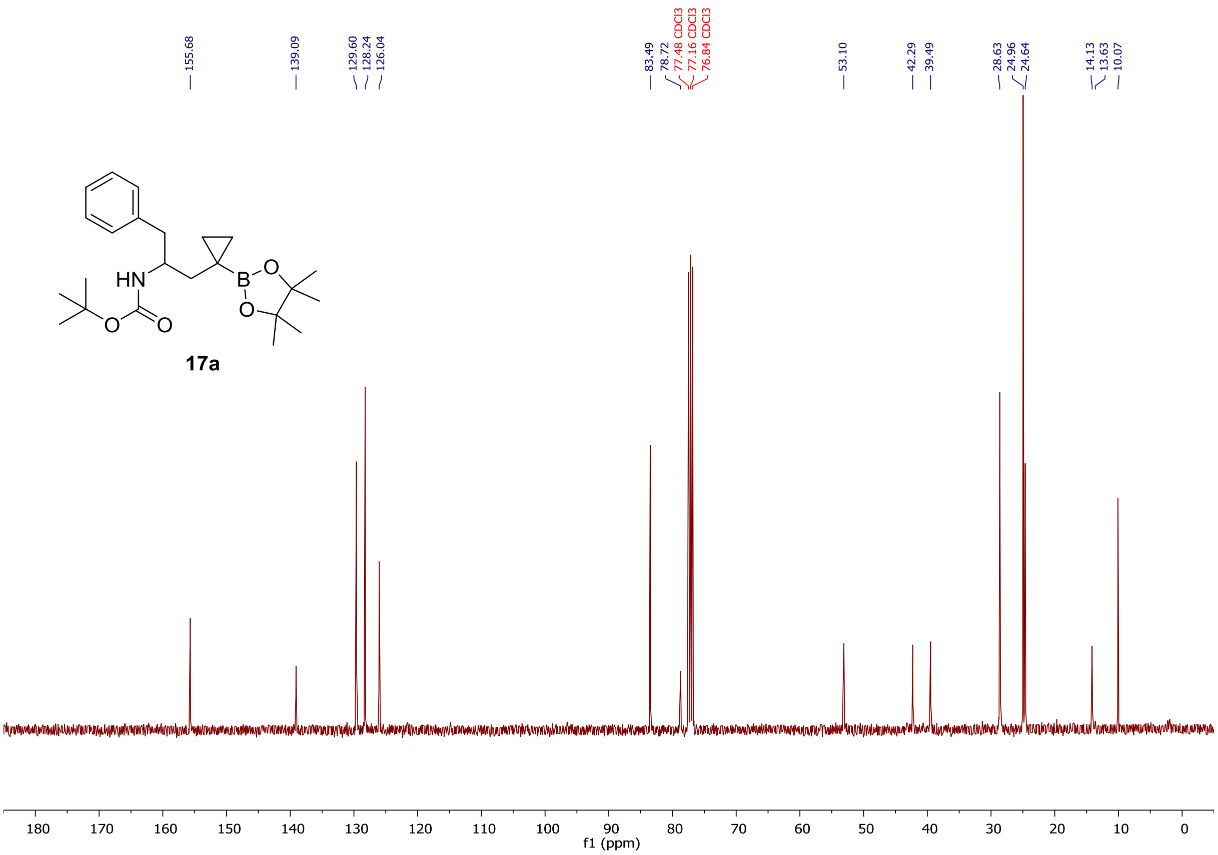

^{1}H NMR (400 MHz, $CDCl_3$)

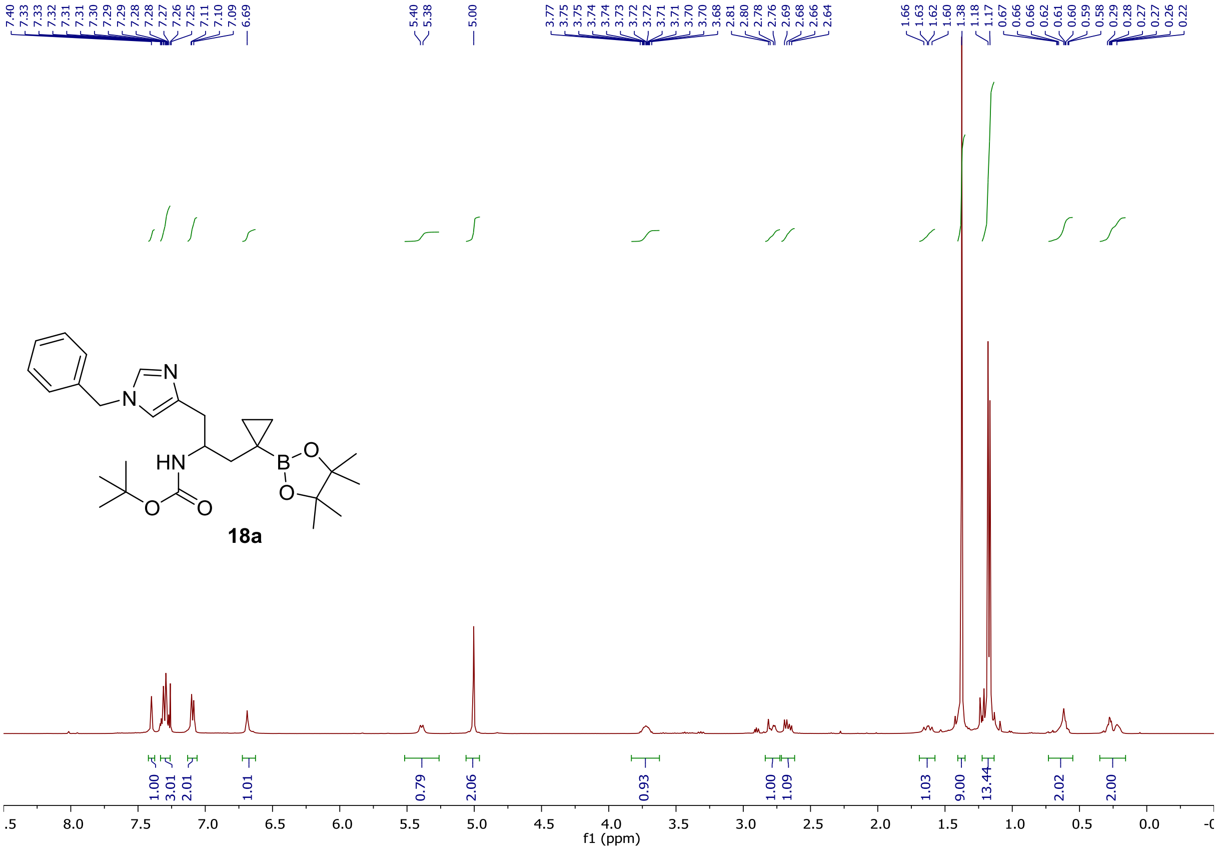

^{13}C NMR (101 MHz, $CDCl_3$)

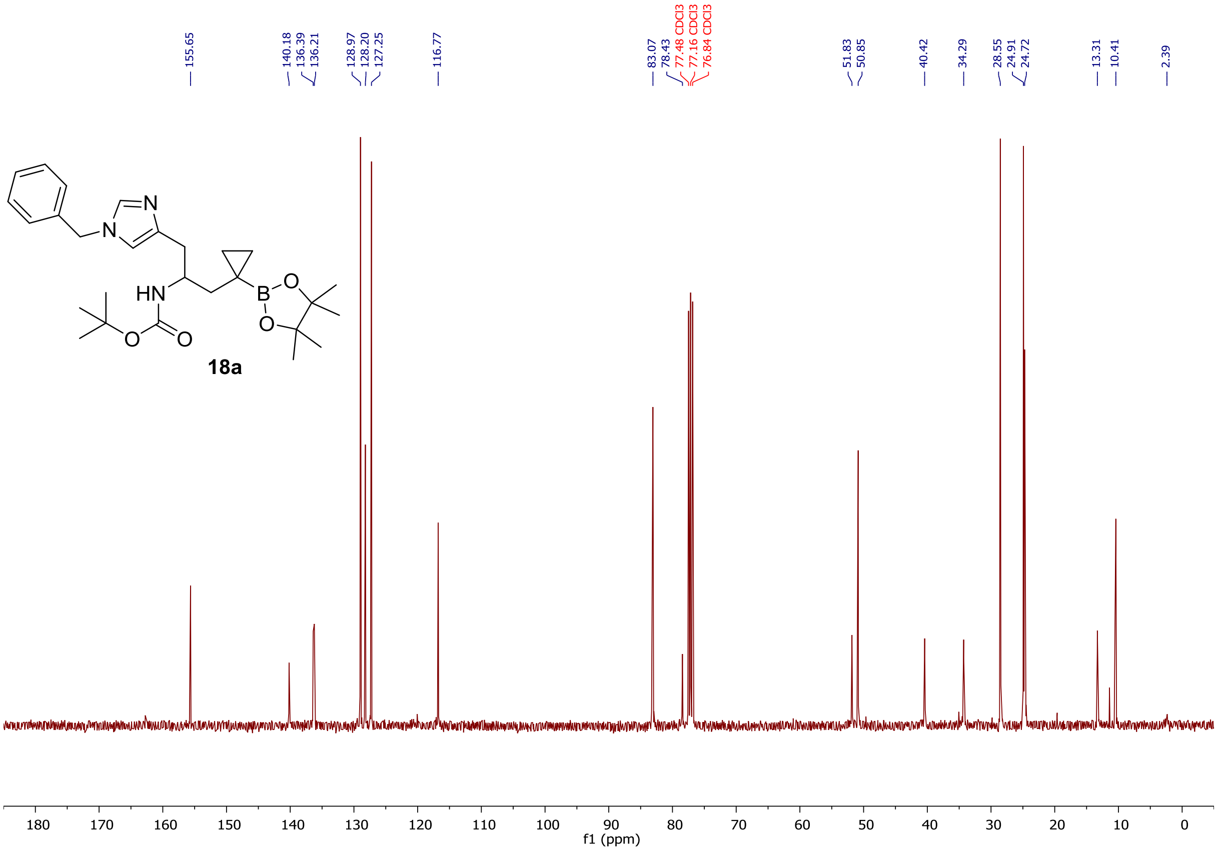

^{1}H NMR (400 MHz, $CDCl_3$)

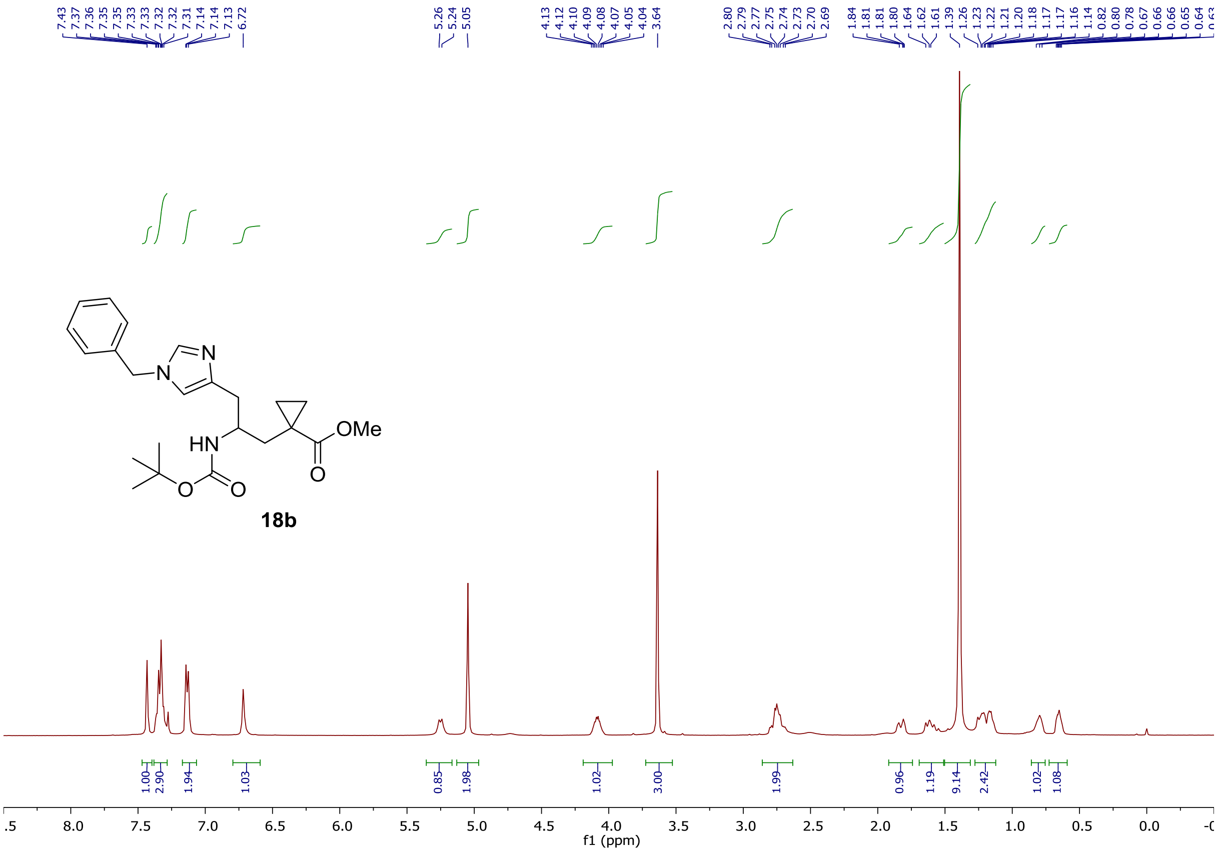

^{13}C NMR (101 MHz, $CDCl_3$)

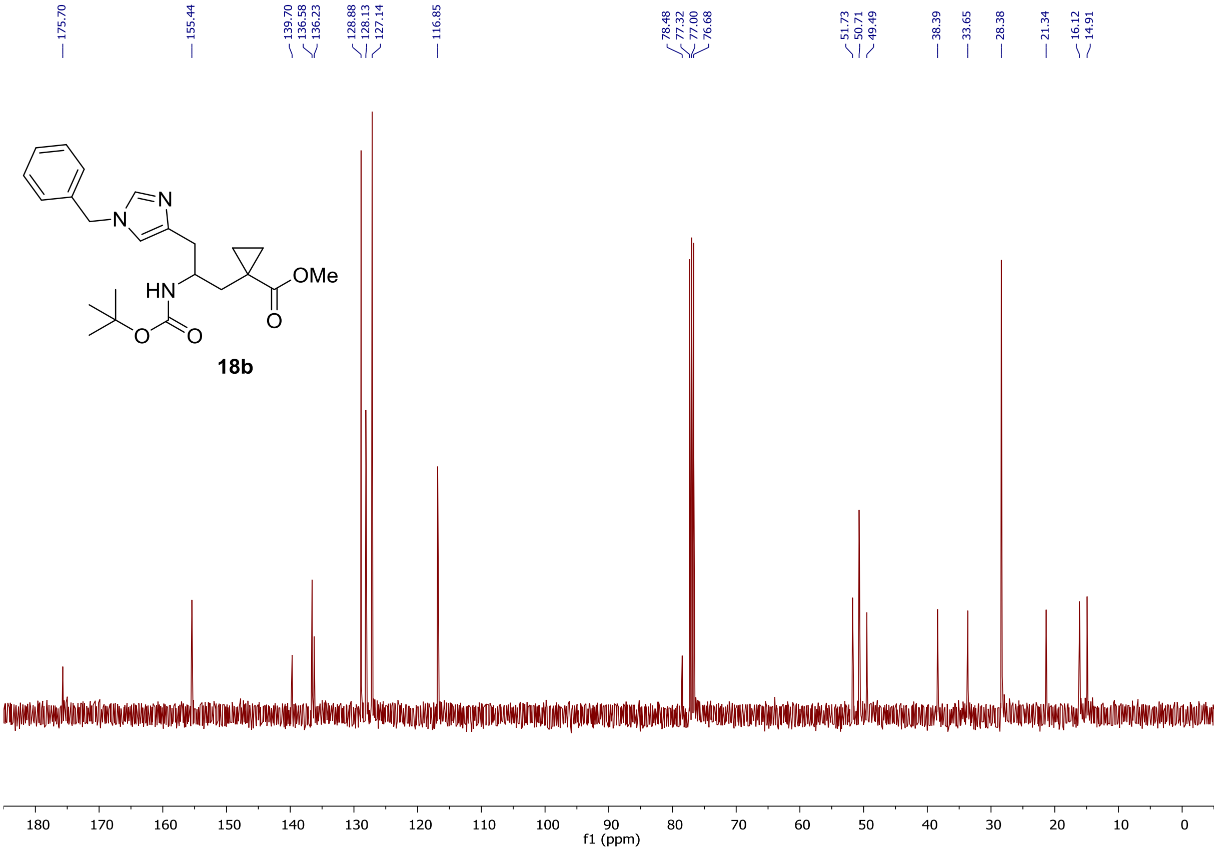

^1H NMR (400 MHz, $CDCl_3$)

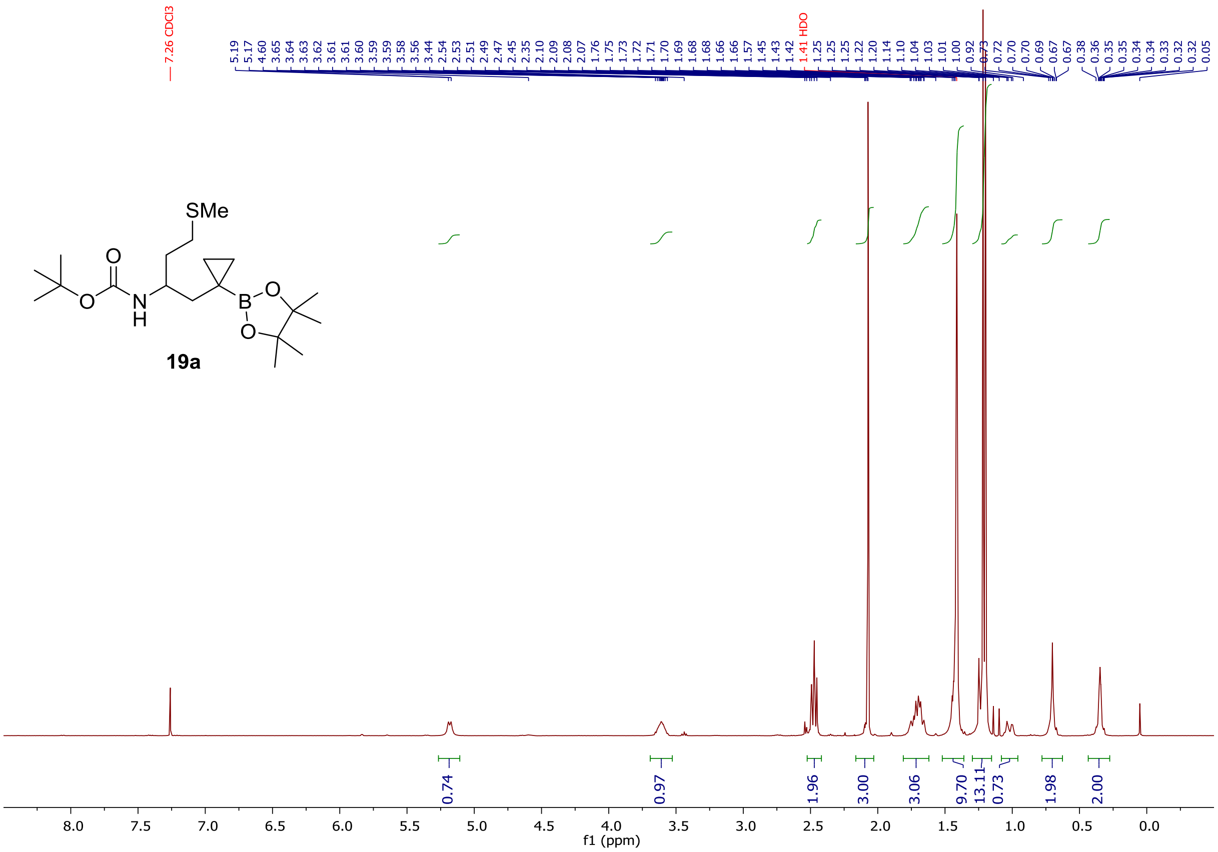

^{13}C NMR (101 MHz, $CDCl_3$)

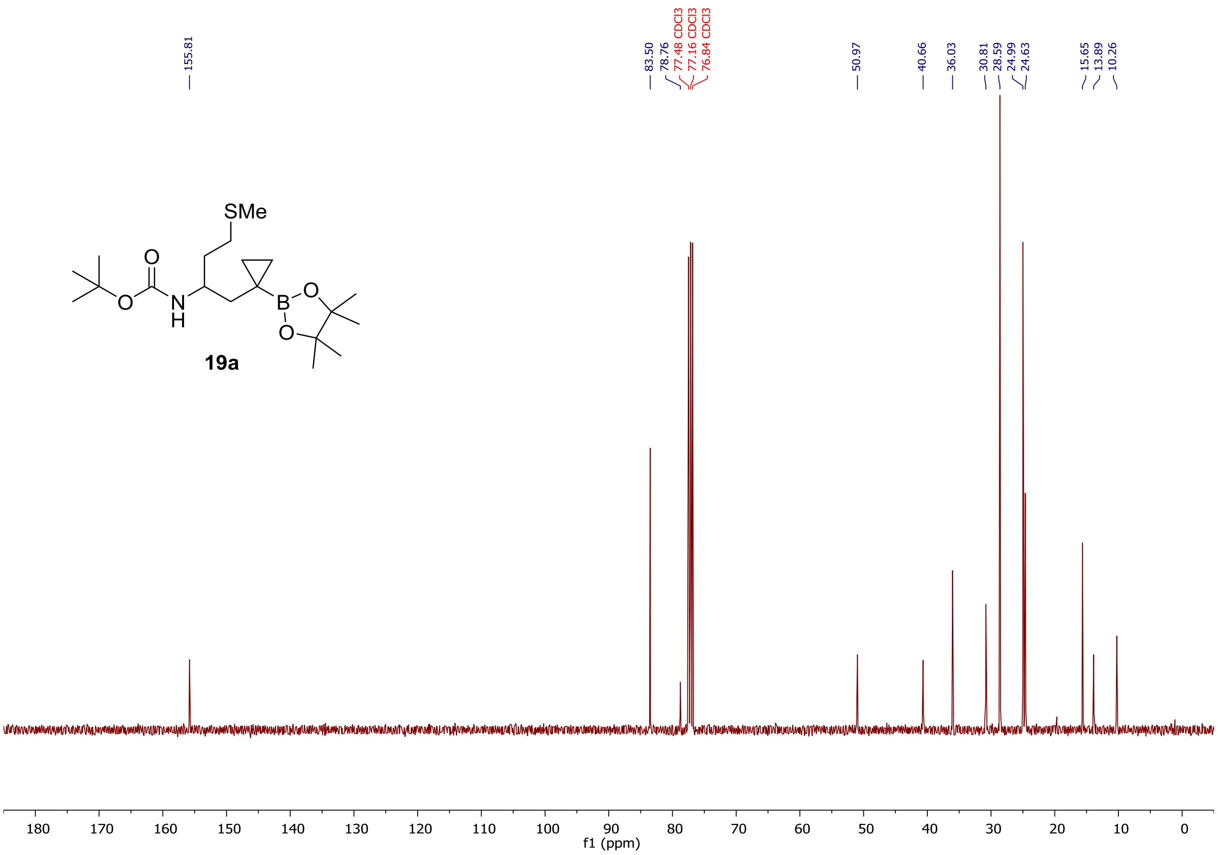

^{1}H NMR (400 MHz, $CDCl_3$)

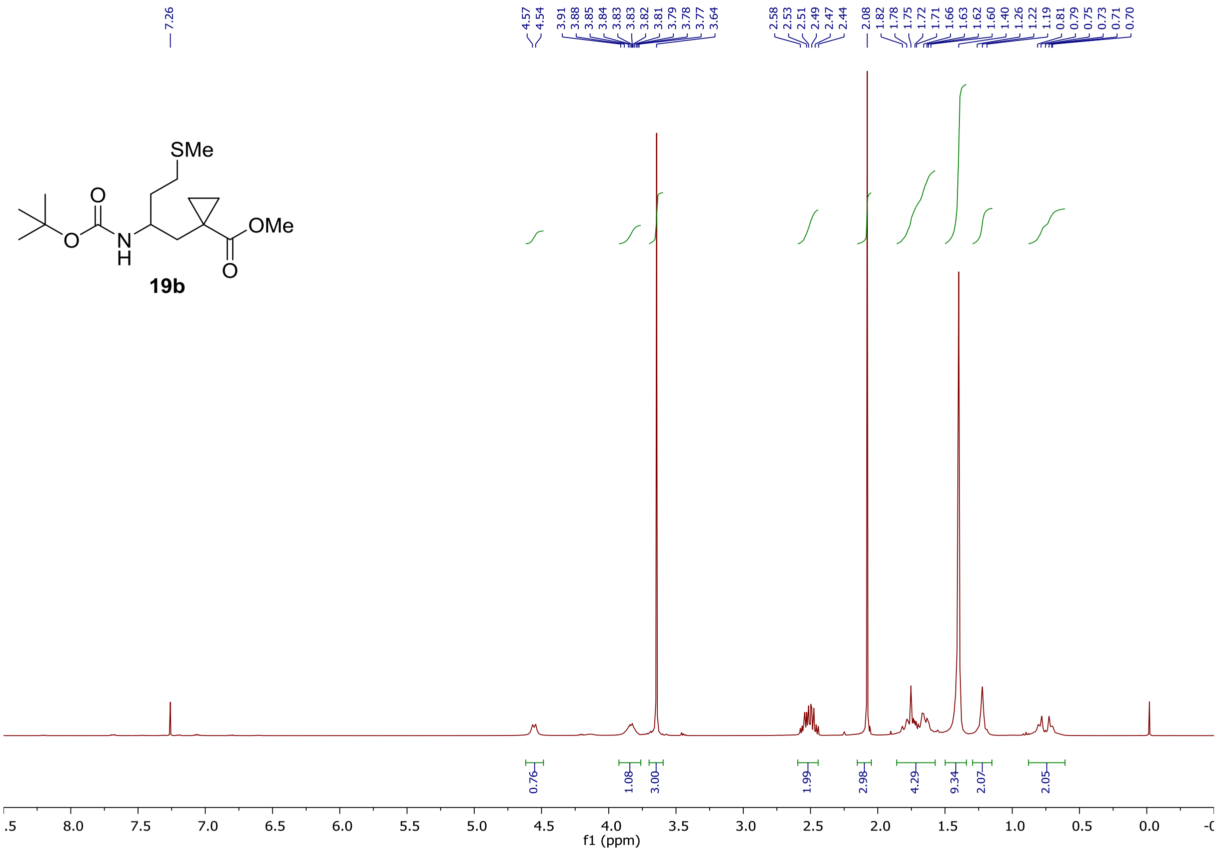

^{13}C NMR (101 MHz, $CDCl_3$)

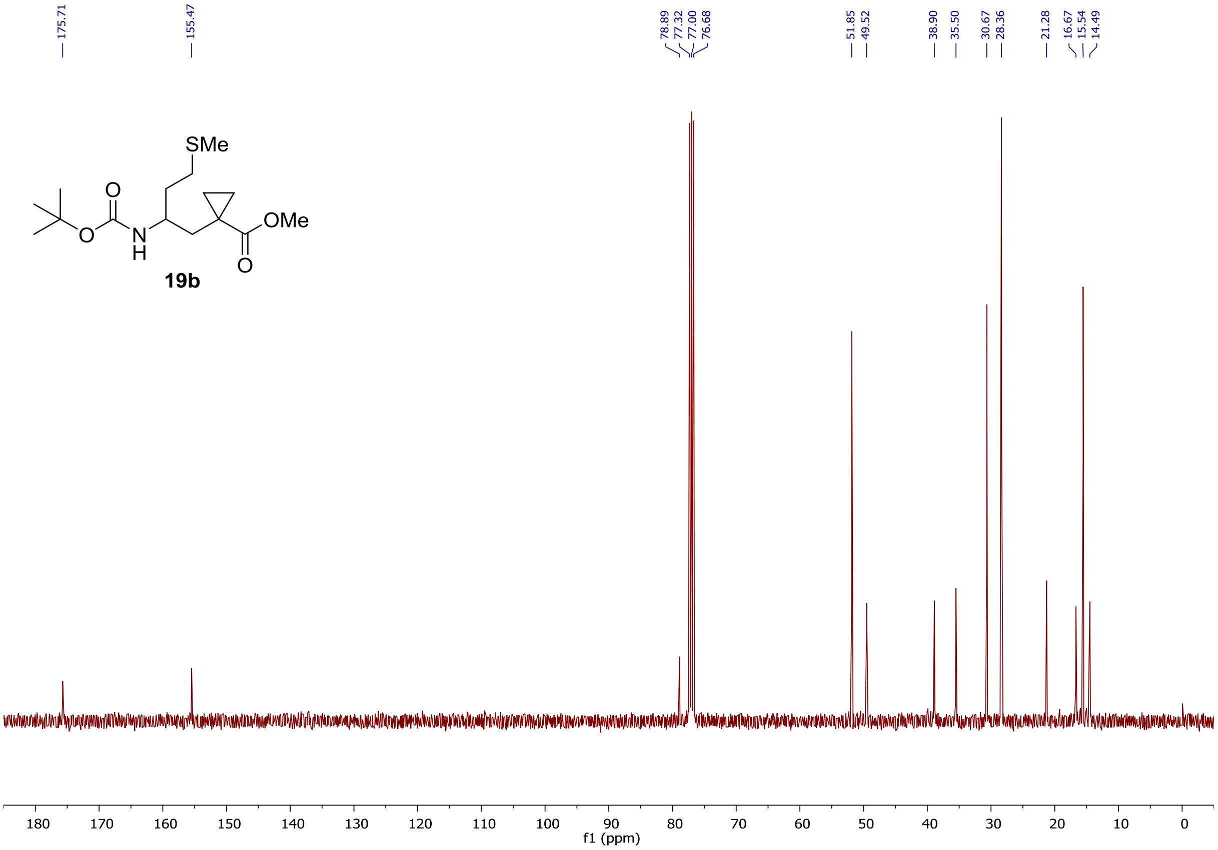

^{1}H NMR (400 MHz, $CDCl_3$)

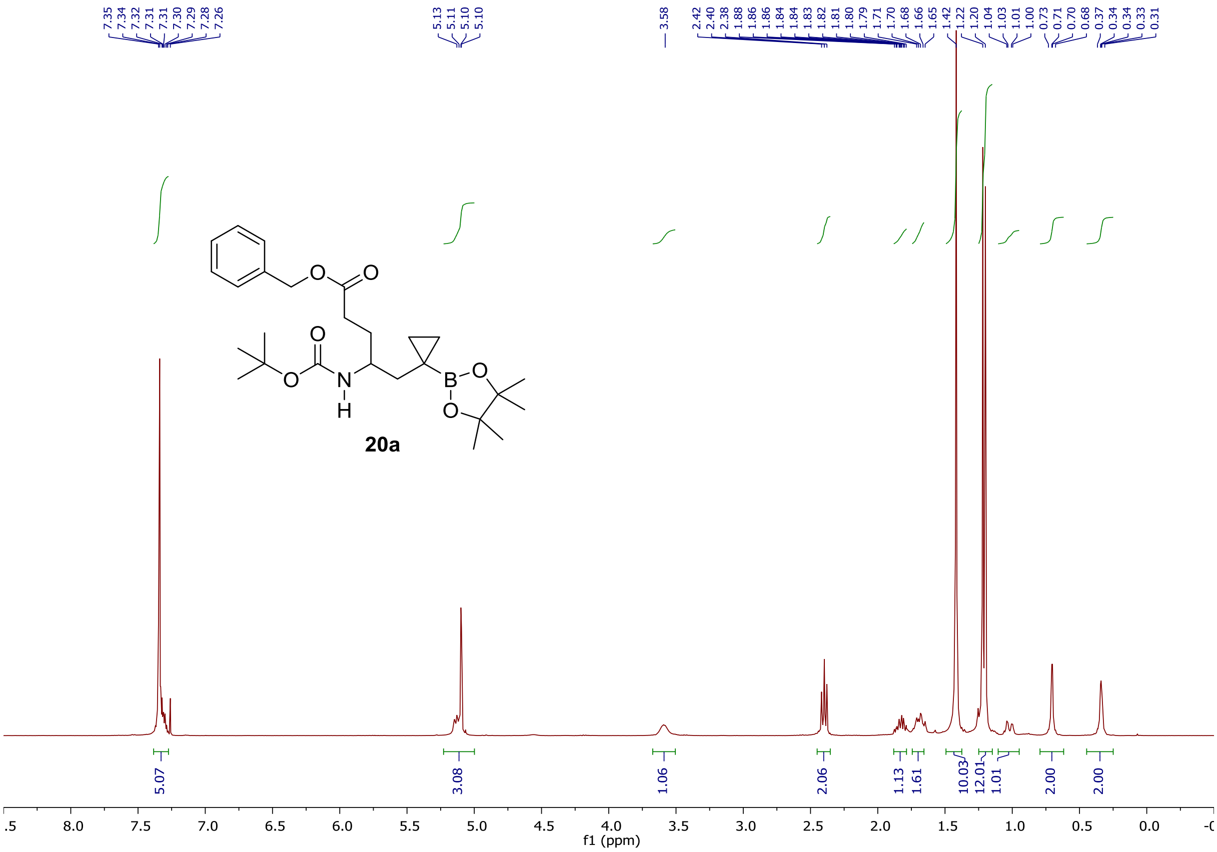

^{13}C NMR (101 MHz, $CDCl_3$)

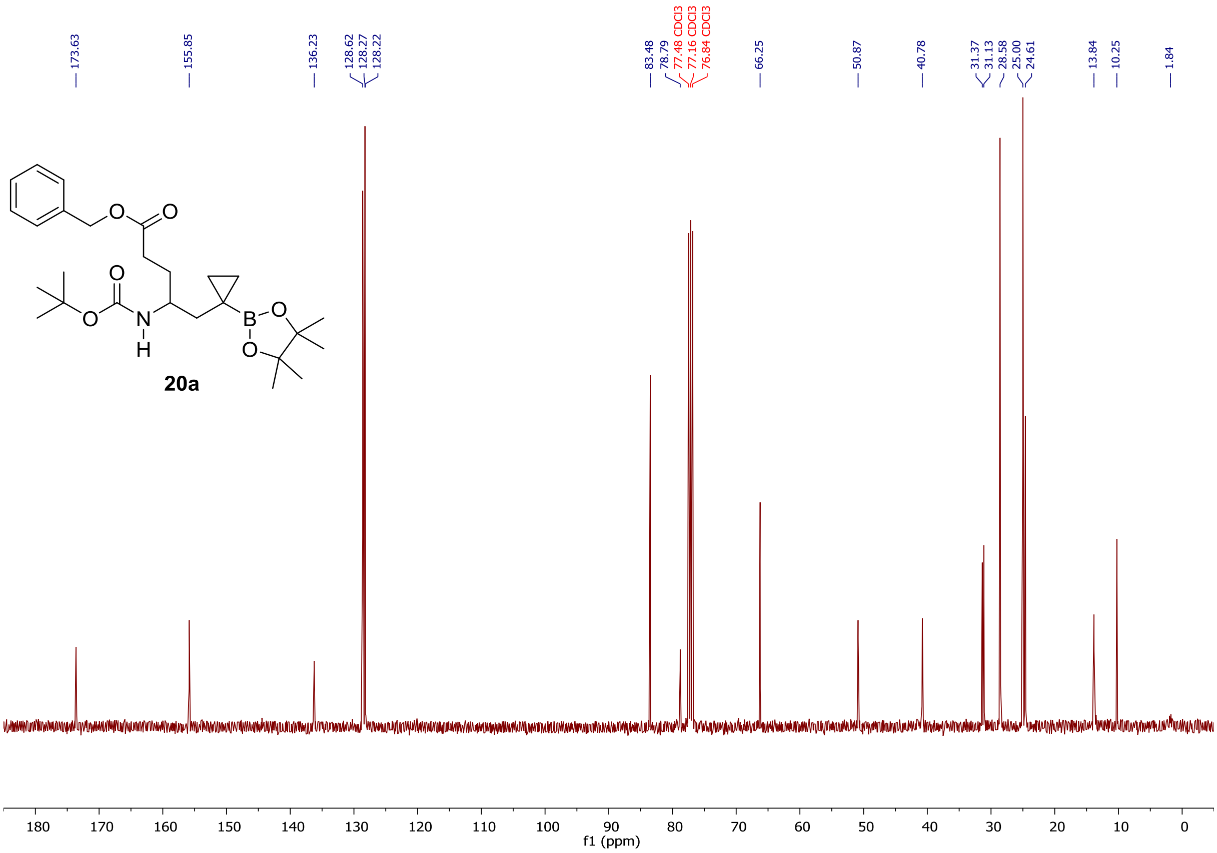

^{1}H NMR (400 MHz, $CDCl_3$)

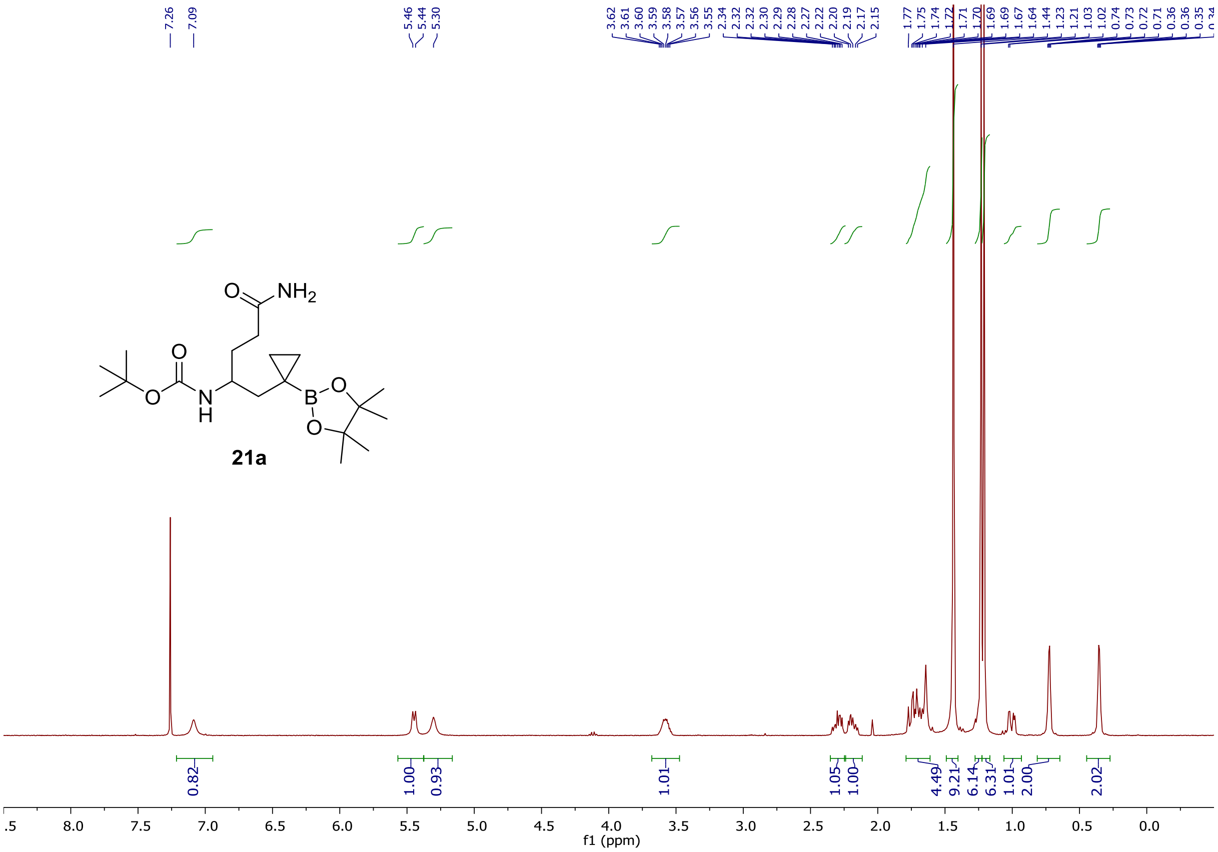

^{13}C NMR (101 MHz, $CDCl_3$)

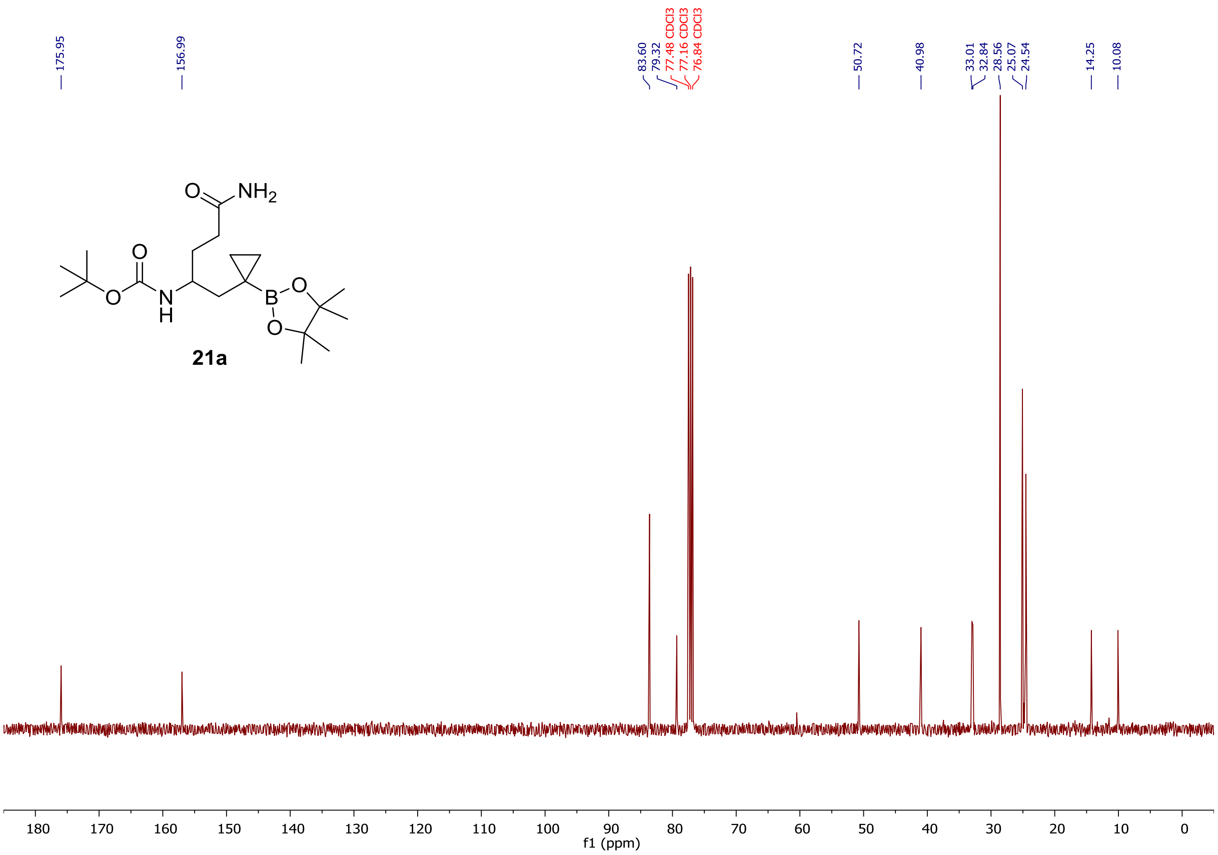

^{1}H NMR (400 MHz, $CDCl_3$)

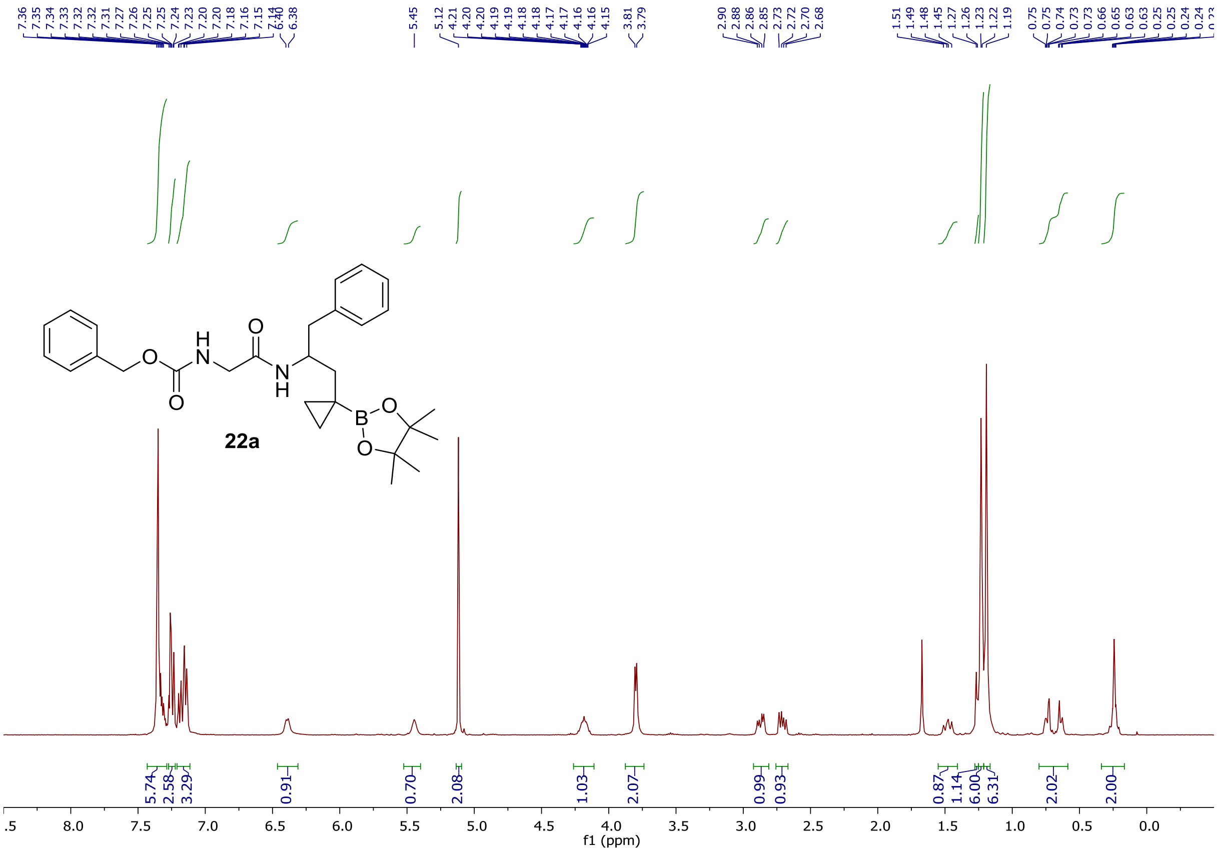

^{13}C NMR (101 MHz, $CDCl_3$)

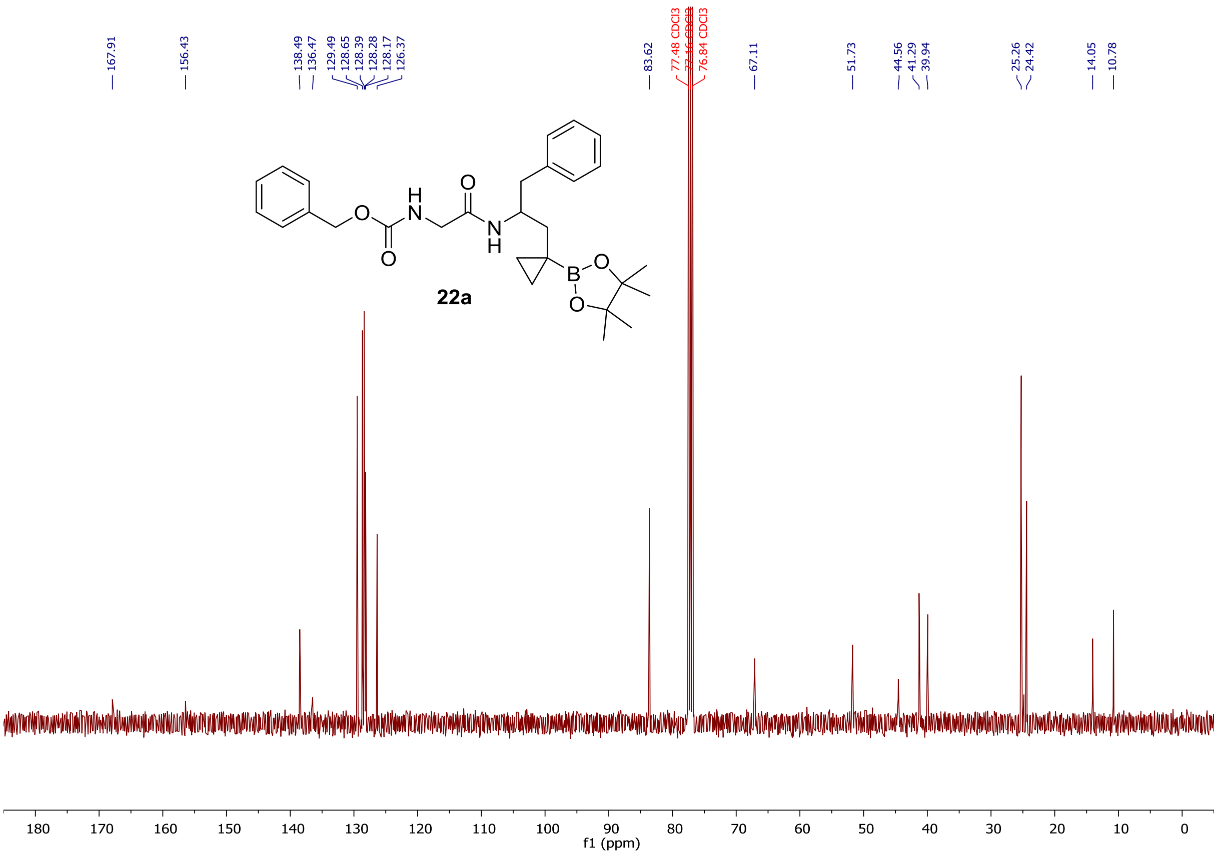

^{1}H NMR (400 MHz, $CDCl_3$)

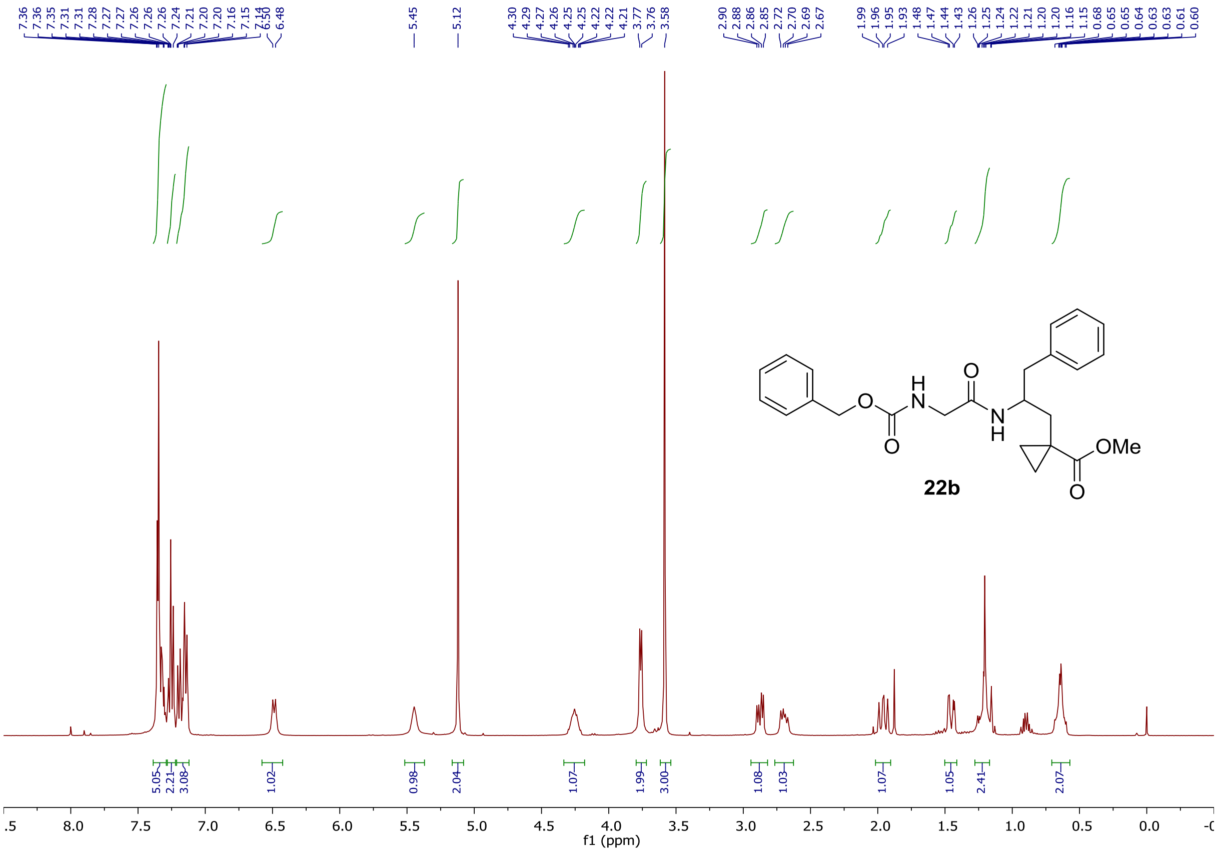

^{13}C NMR (101 MHz, $CDCl_3$)

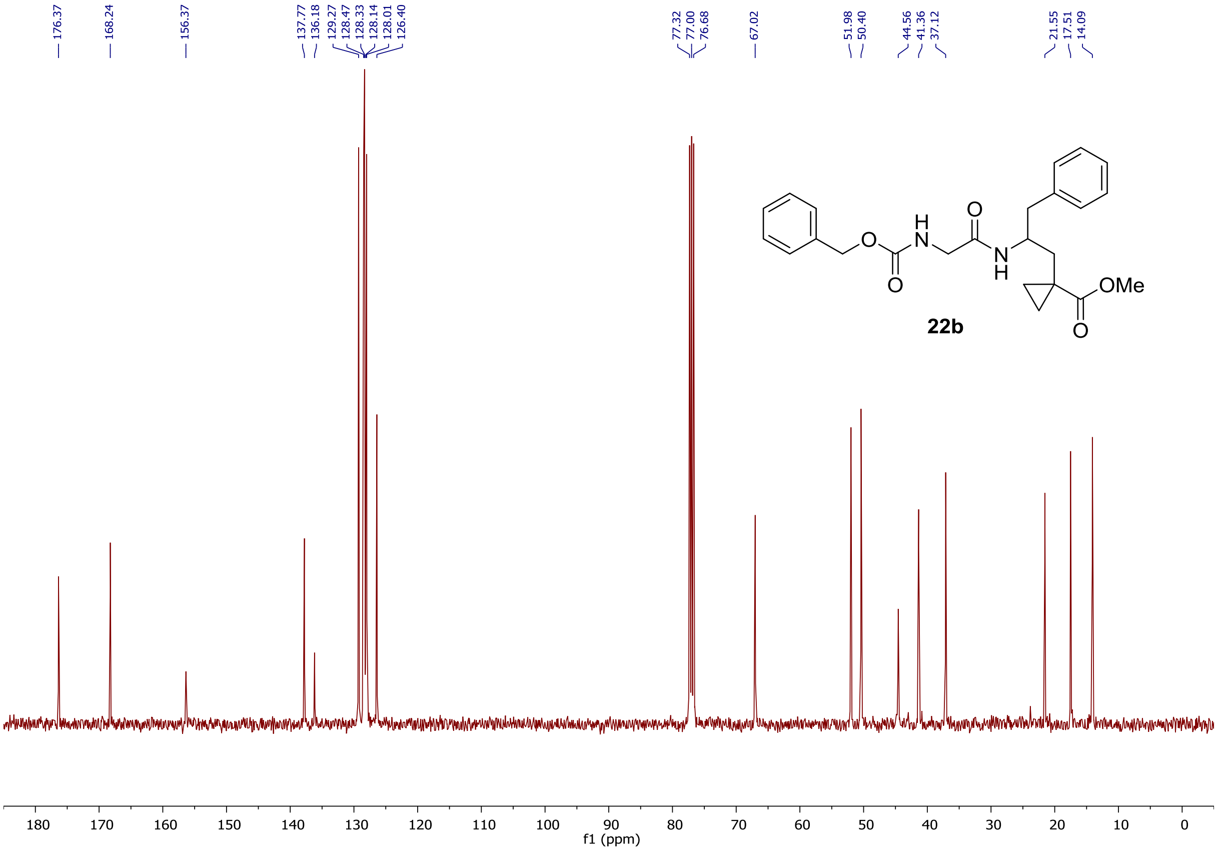

^{1}H NMR (500 MHz, DMSO-d_6, 100°C)

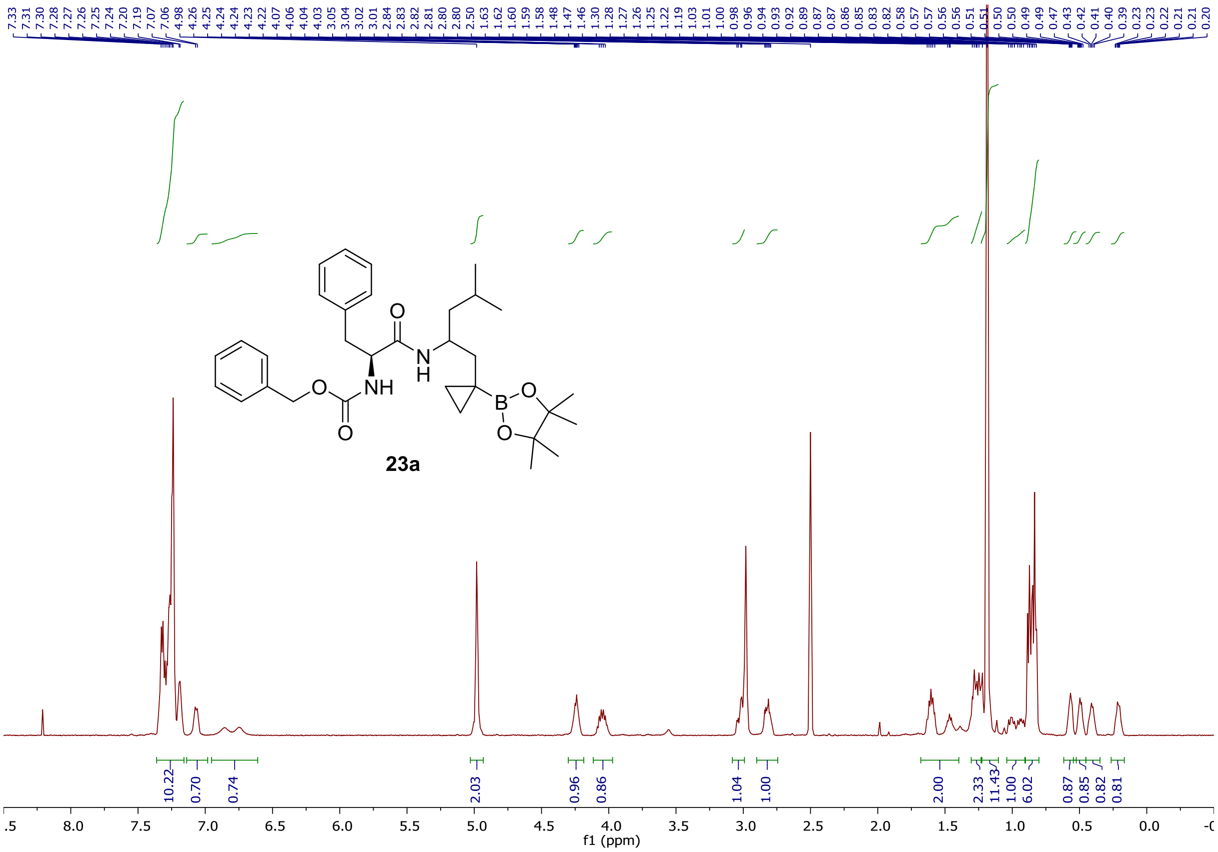

^{13}C NMR (126 MHz, DMSO-d_6, 100°C)

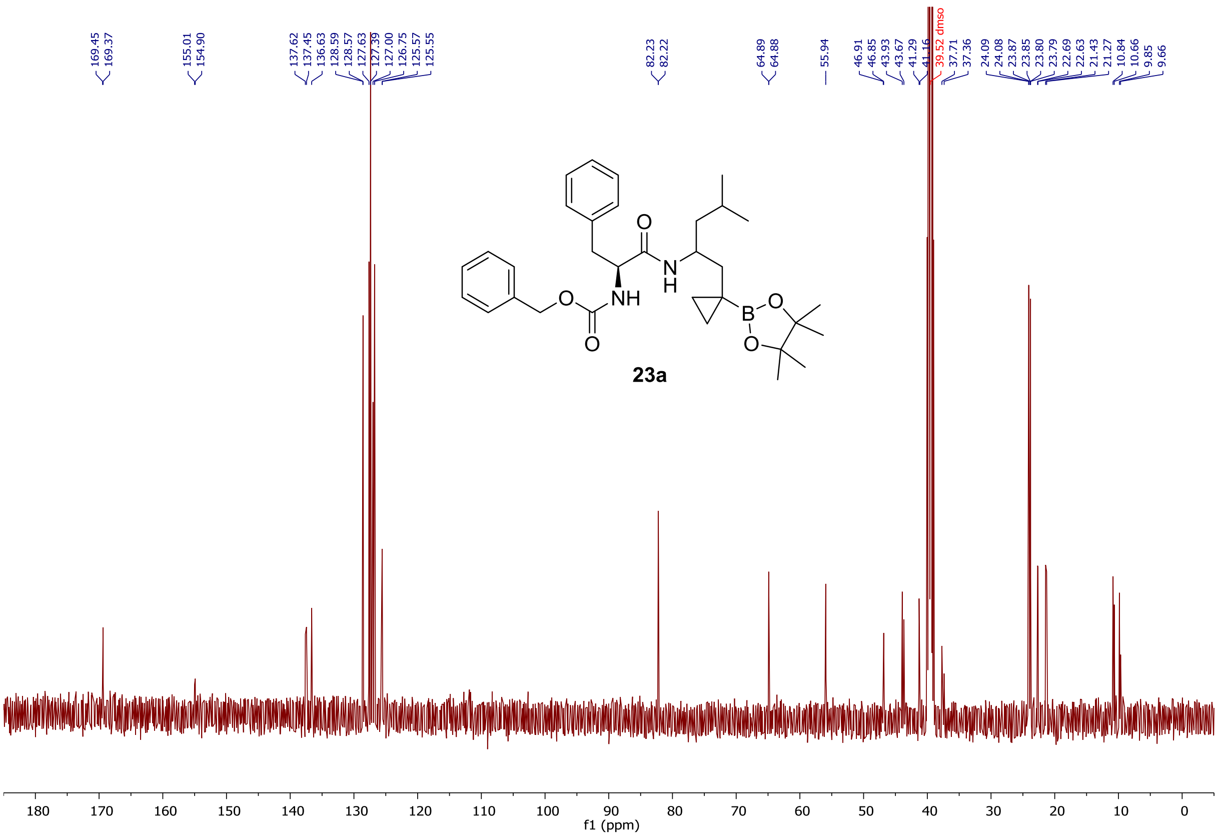

^{1}H NMR (400 MHz, $CDCl_3$)

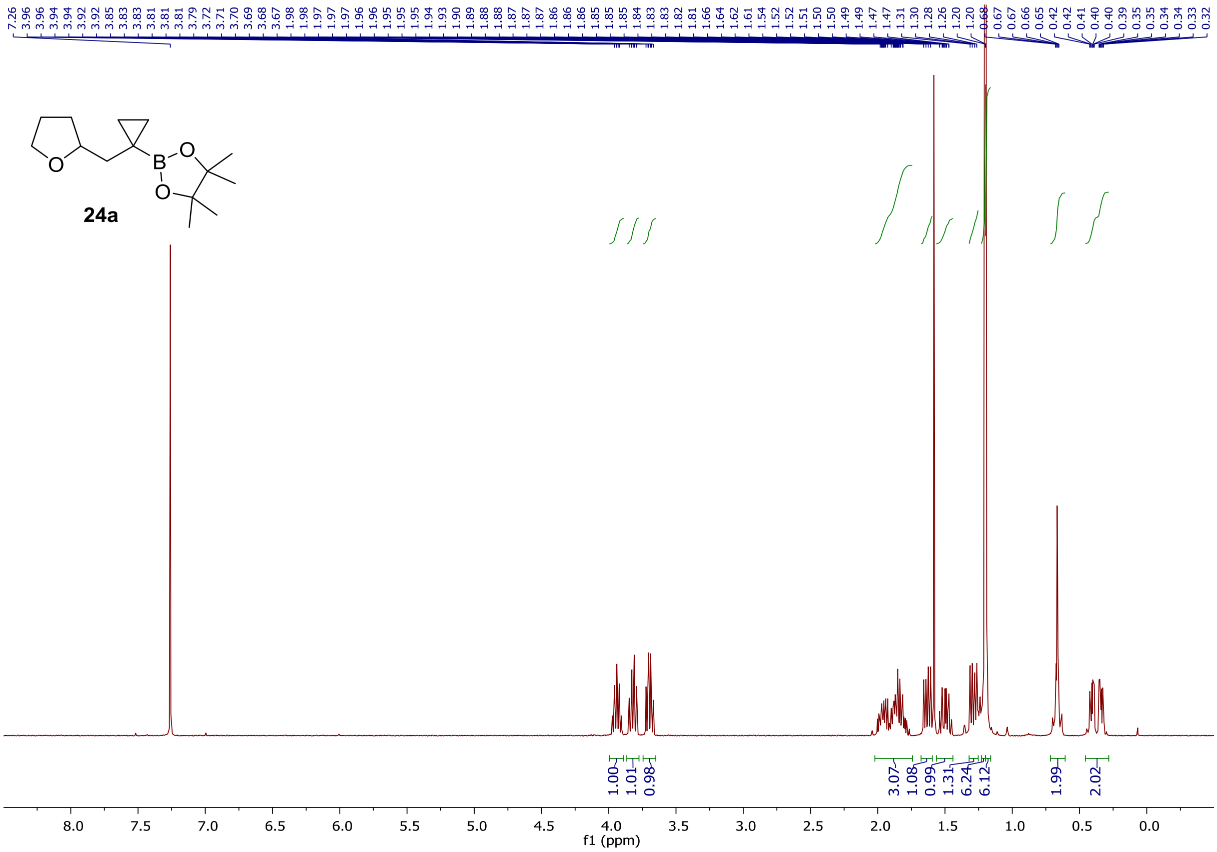

^{13}C NMR (101 MHz, $CDCl_3$)

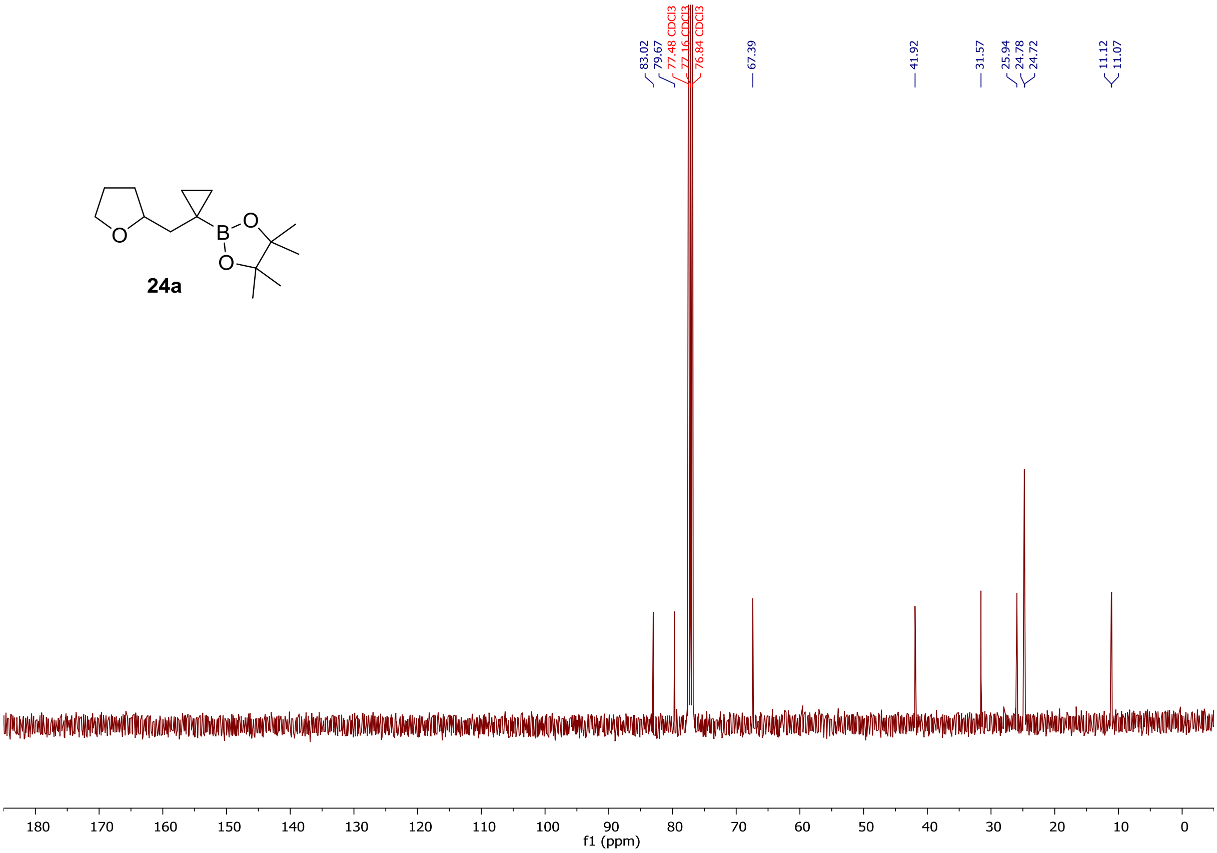

^{1}H NMR (400 MHz, $CDCl_3$)

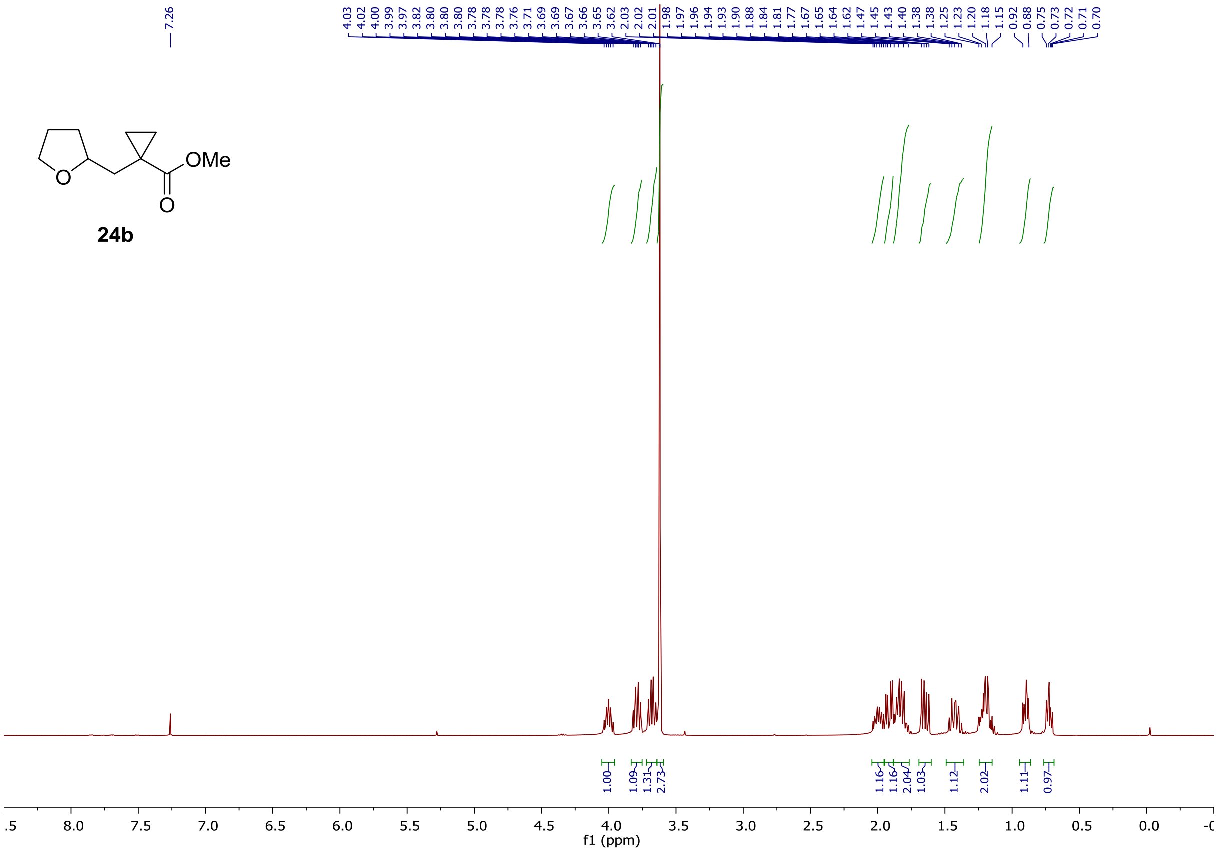

24b

^{13}C NMR (101 MHz, $CDCl_3$)

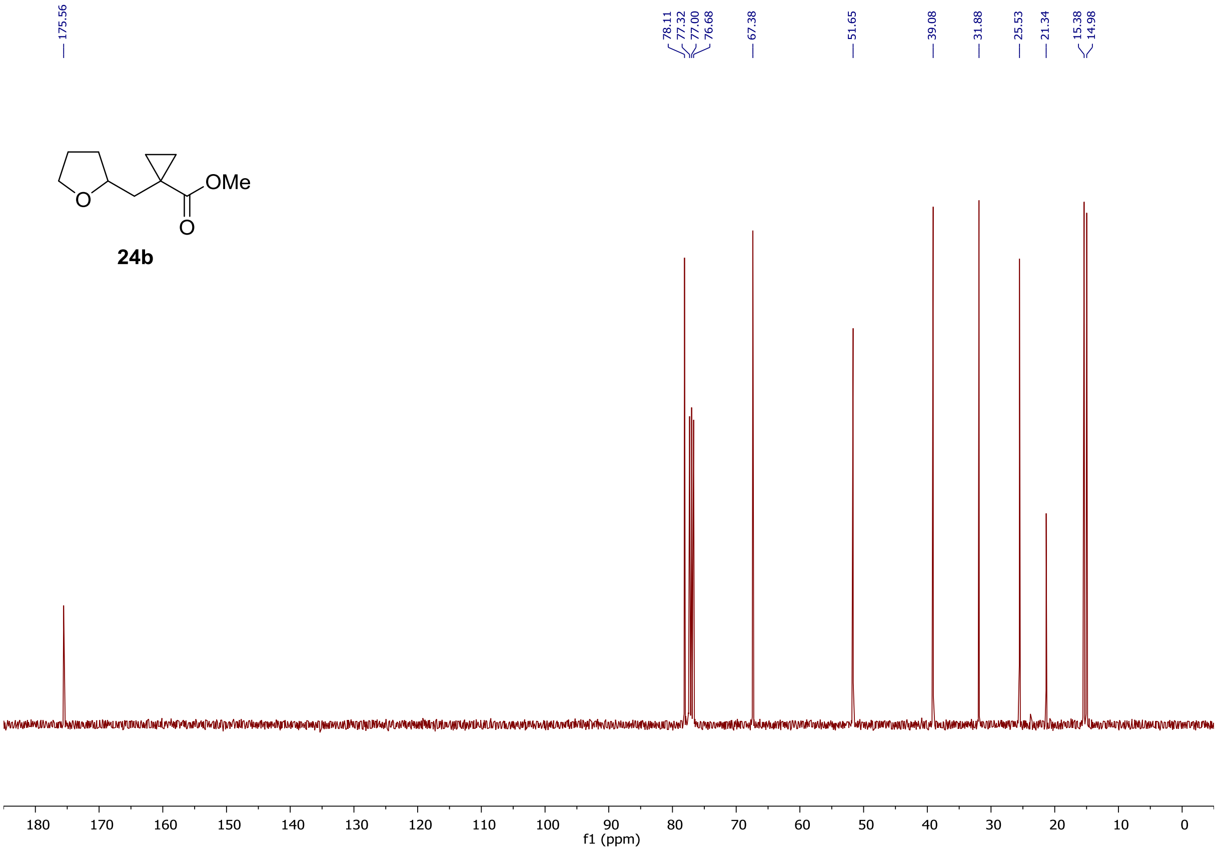

24b

^{1}H NMR (400 MHz, $CDCl_3$)

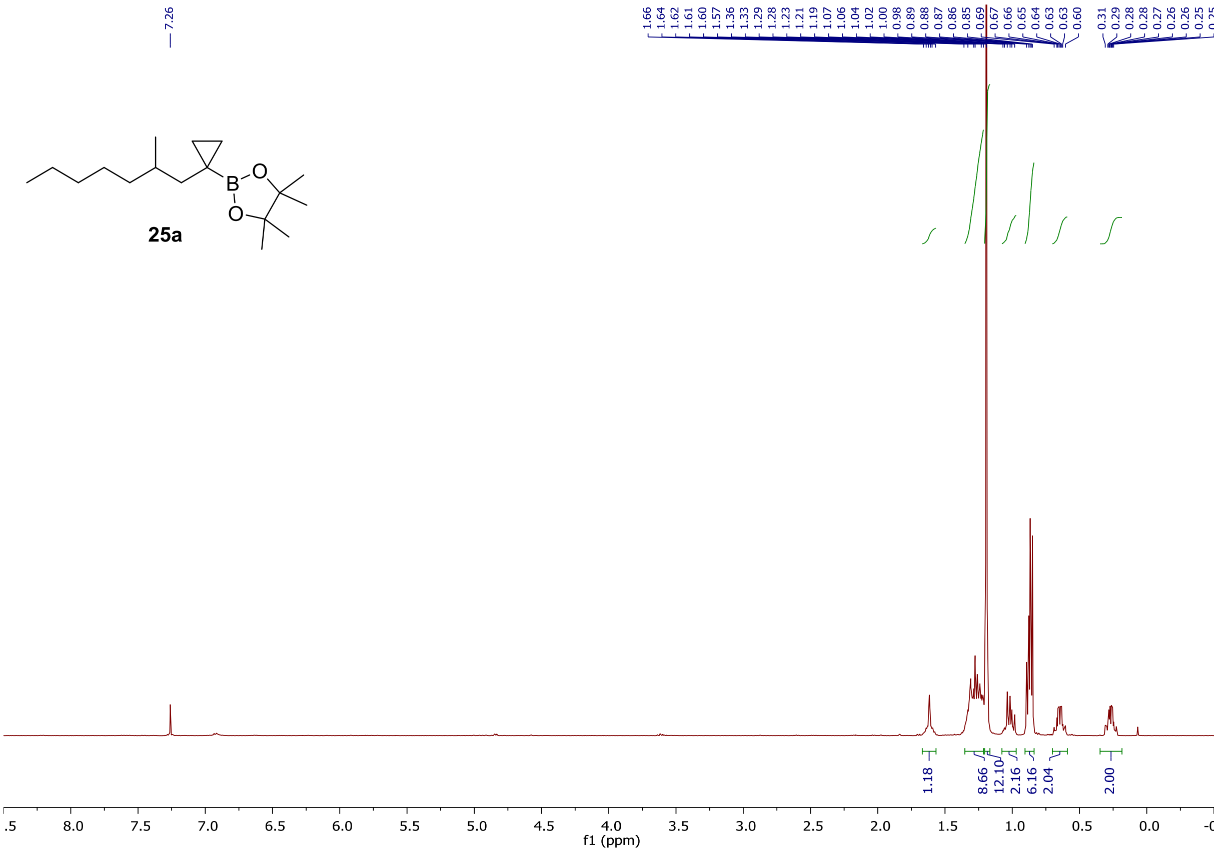

^{13}C NMR (101 MHz, $CDCl_3$)

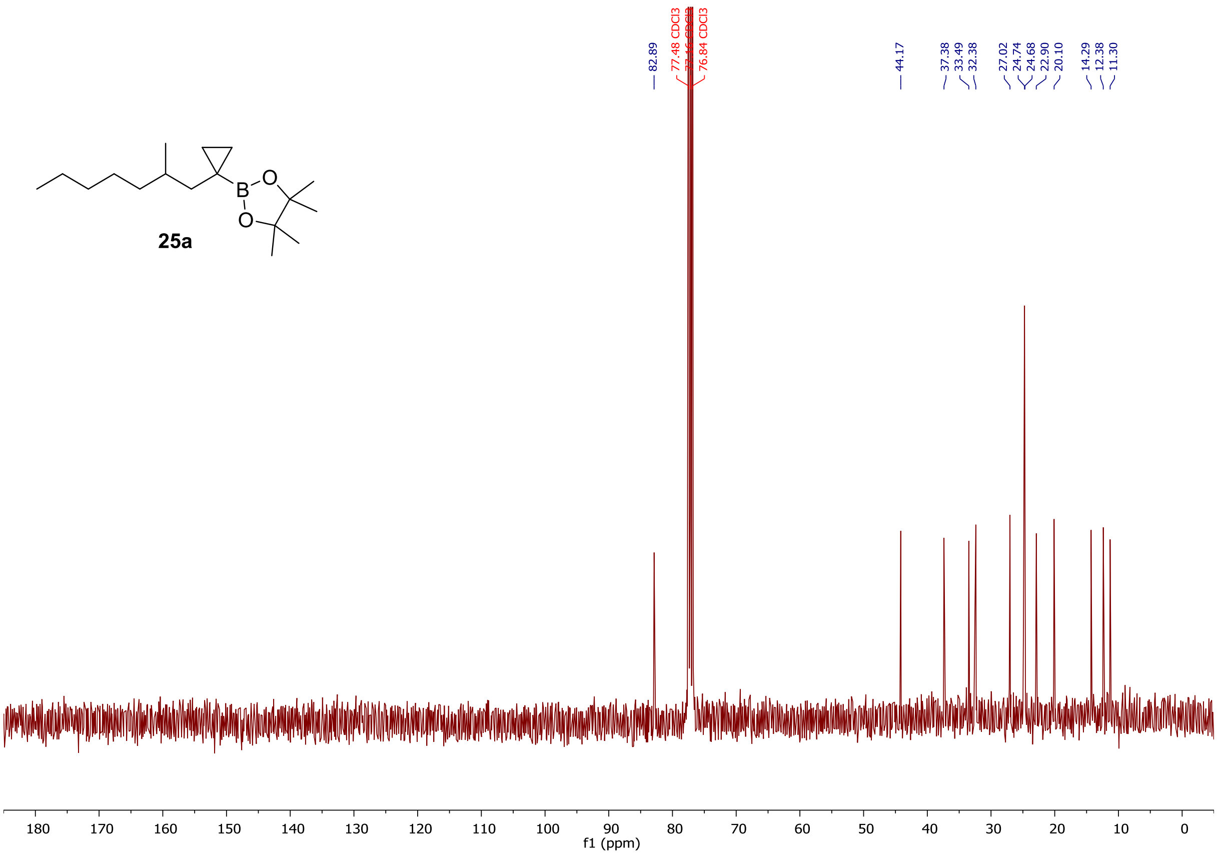

^{1}H NMR (400 MHz, $CDCl_3$)

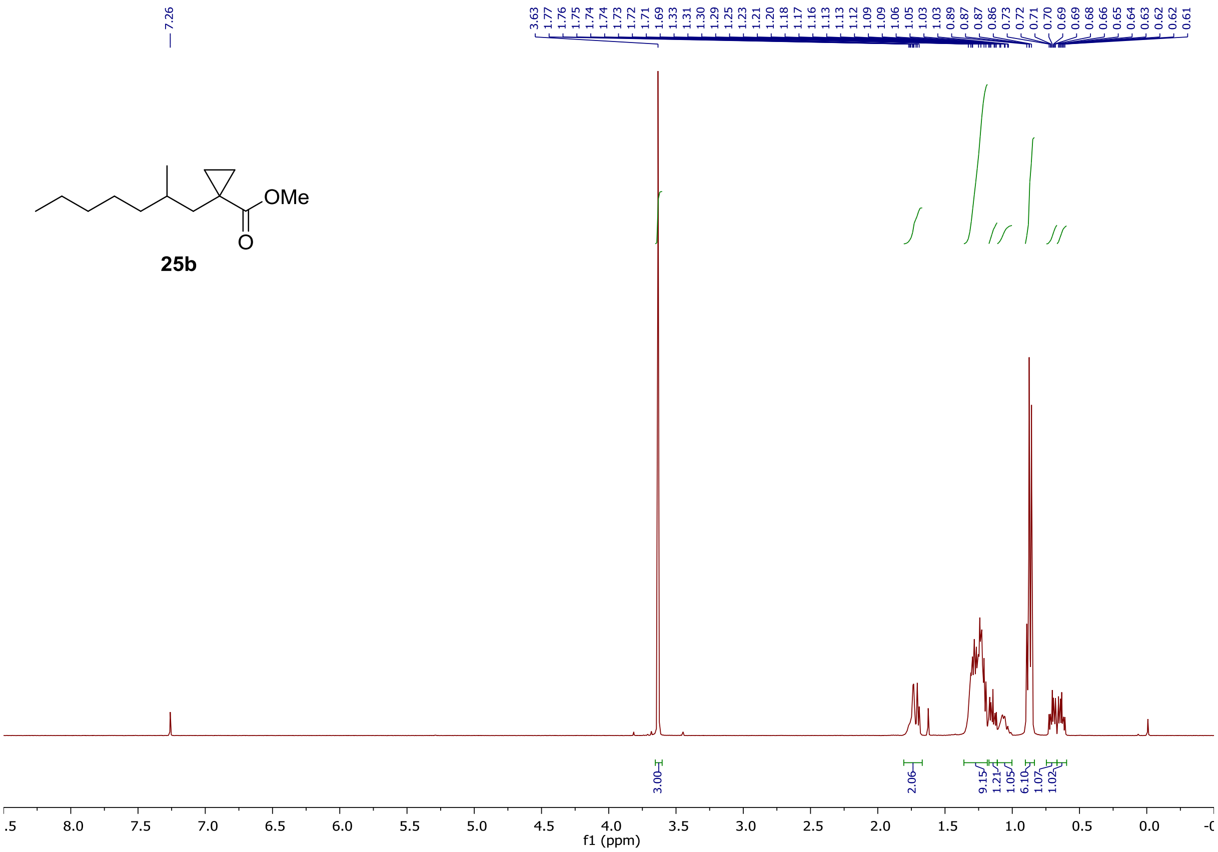

^{13}C NMR (101 MHz, $CDCl_3$)

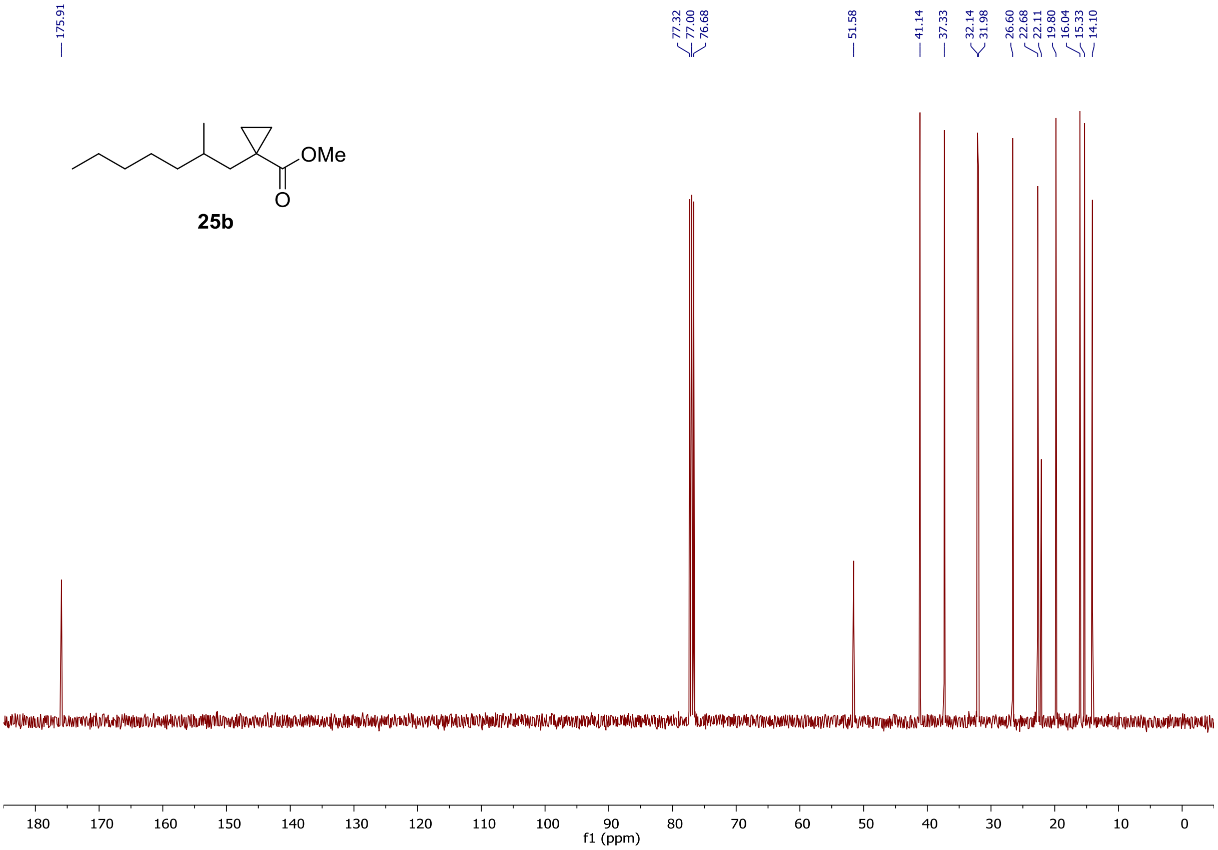

^{1}H NMR (400 MHz, $CDCl_3$)

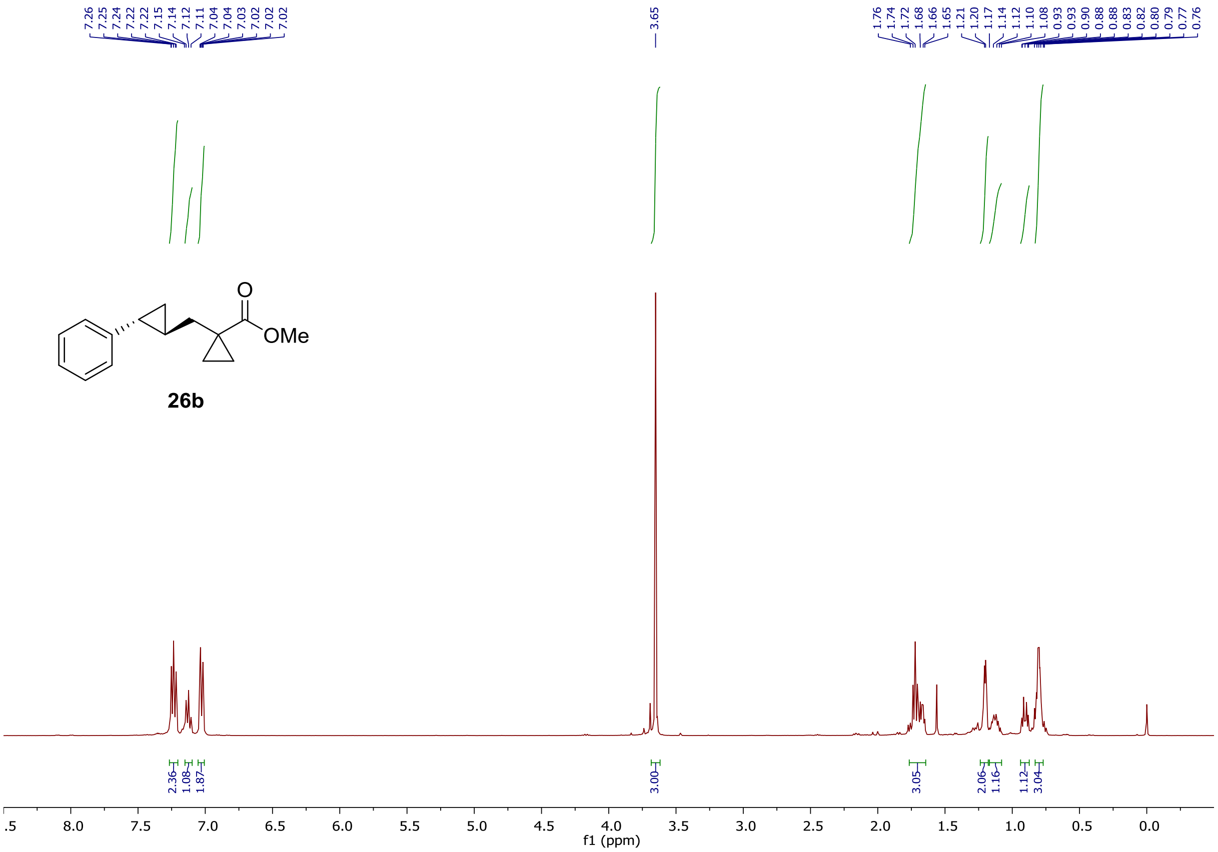

^{13}C NMR (101 MHz, $CDCl_3$)

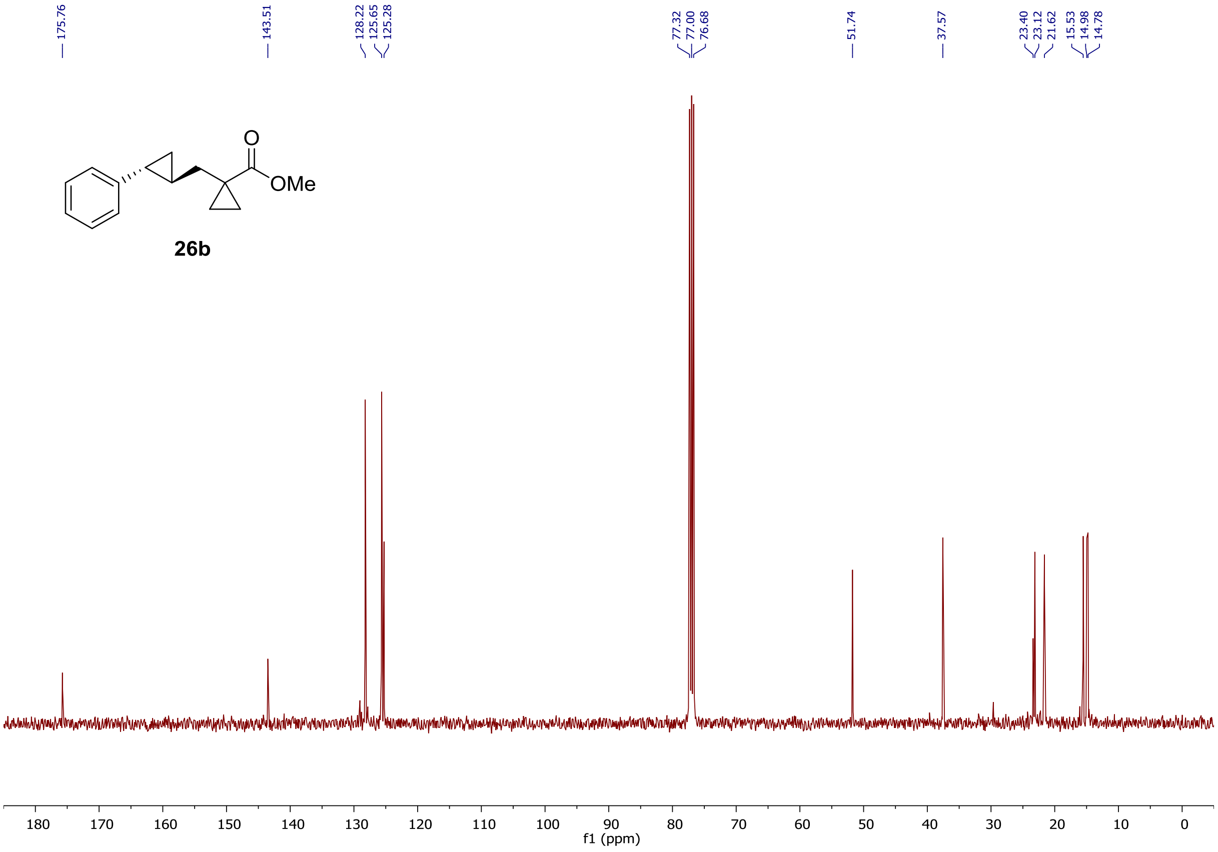

^{1}H NMR (400 MHz, $CDCl_3$)

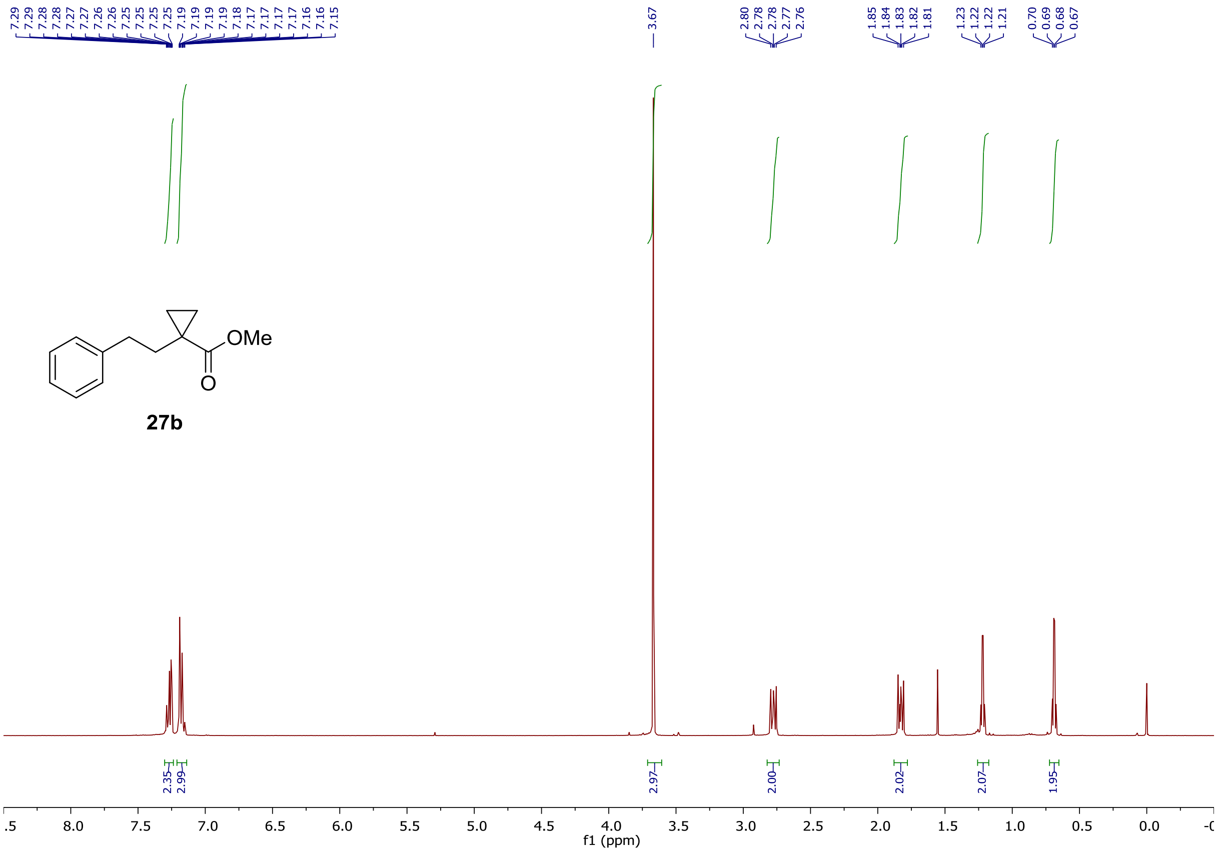

^{13}C NMR (101 MHz, $CDCl_3$)

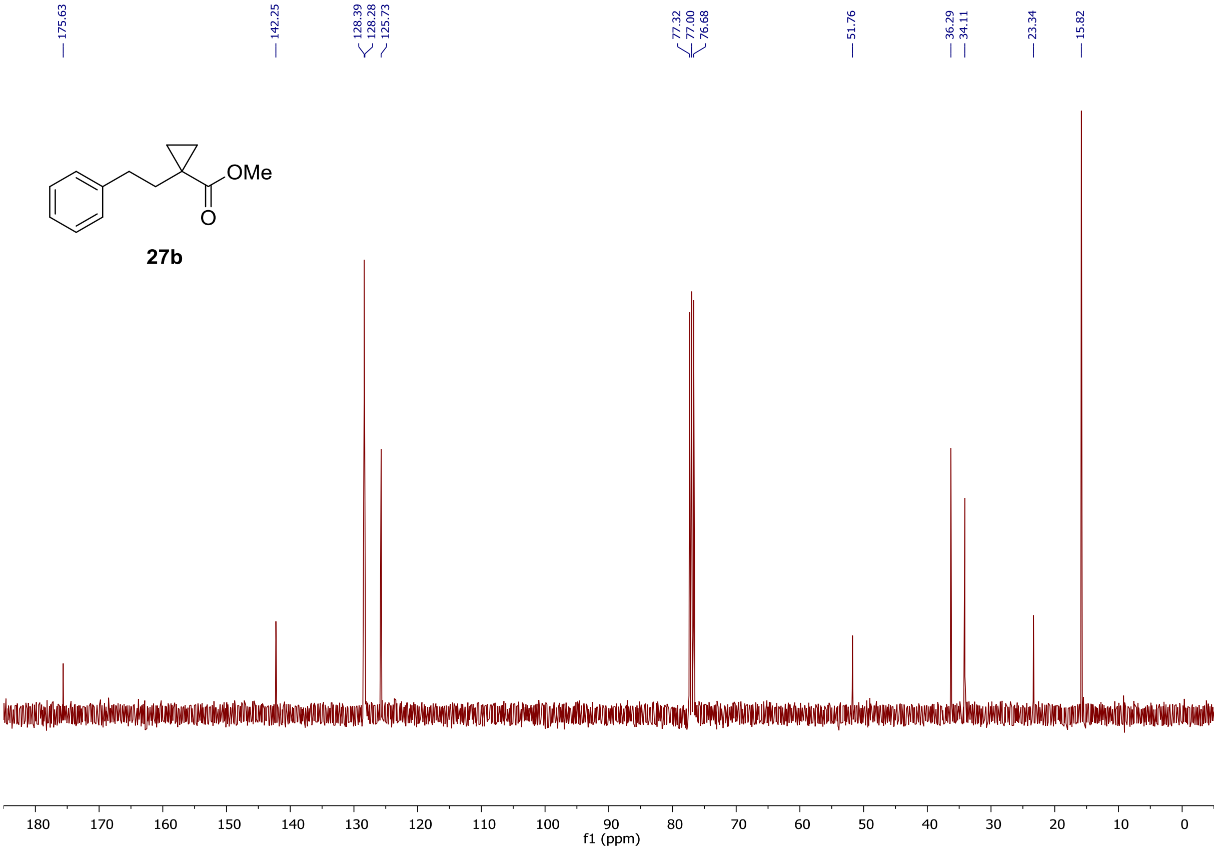

^{1}H NMR (400 MHz, $CDCl_3$)

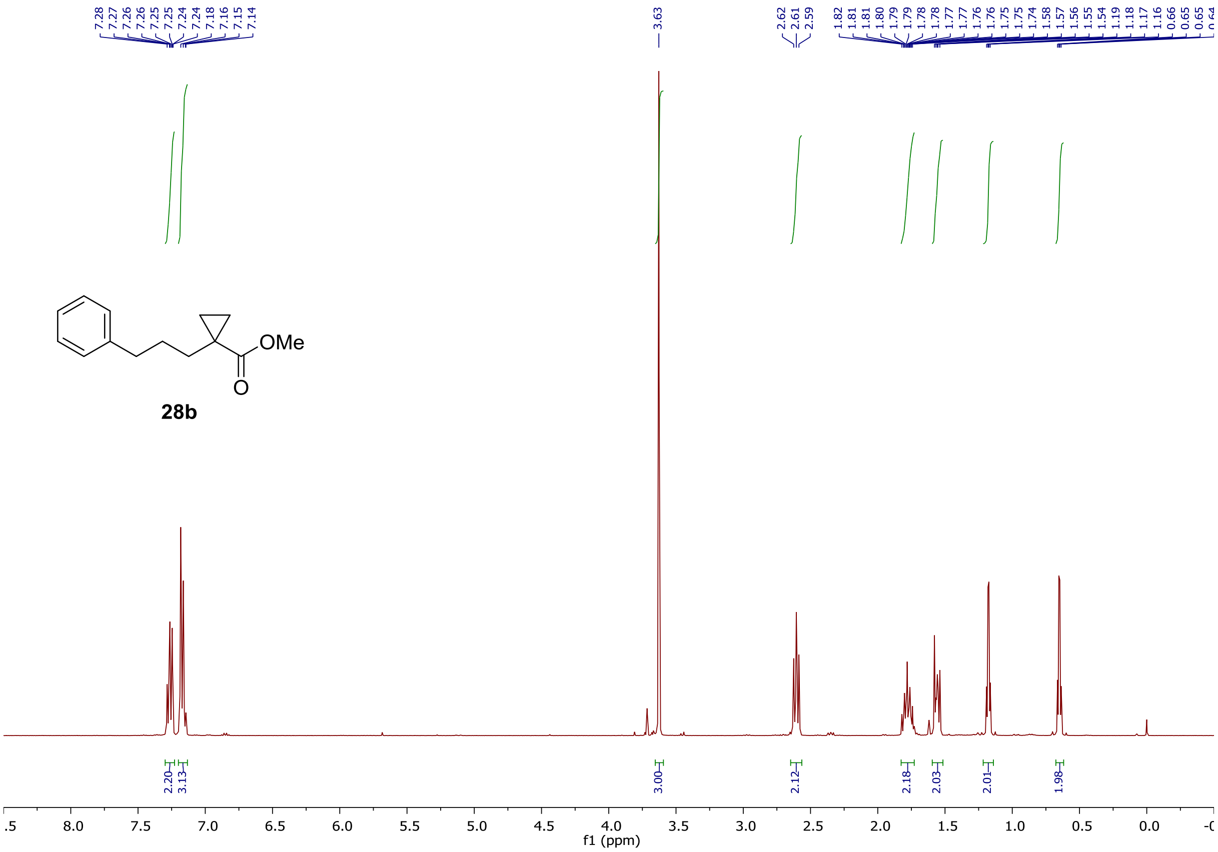

^{13}C NMR (101 MHz, $CDCl_3$)

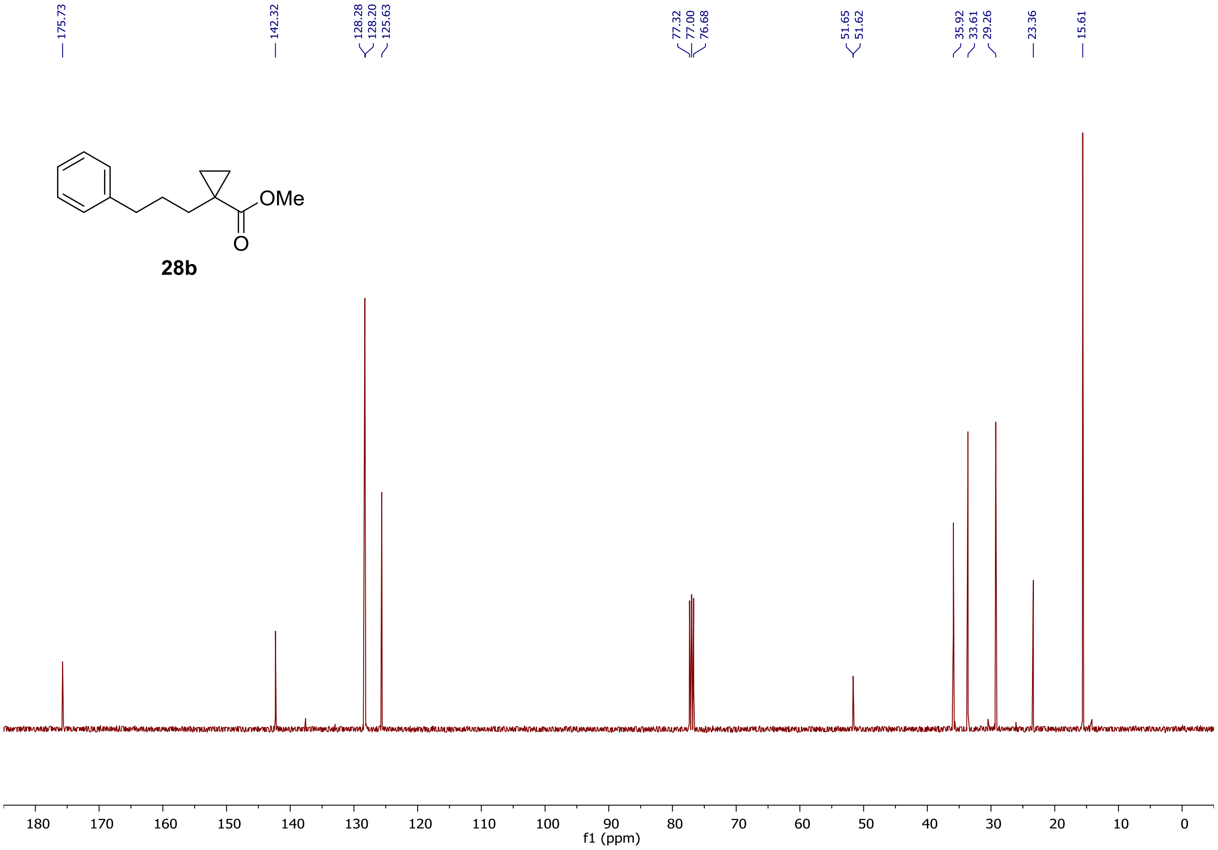

^{1}H NMR (400 MHz, $CDCl_3$)

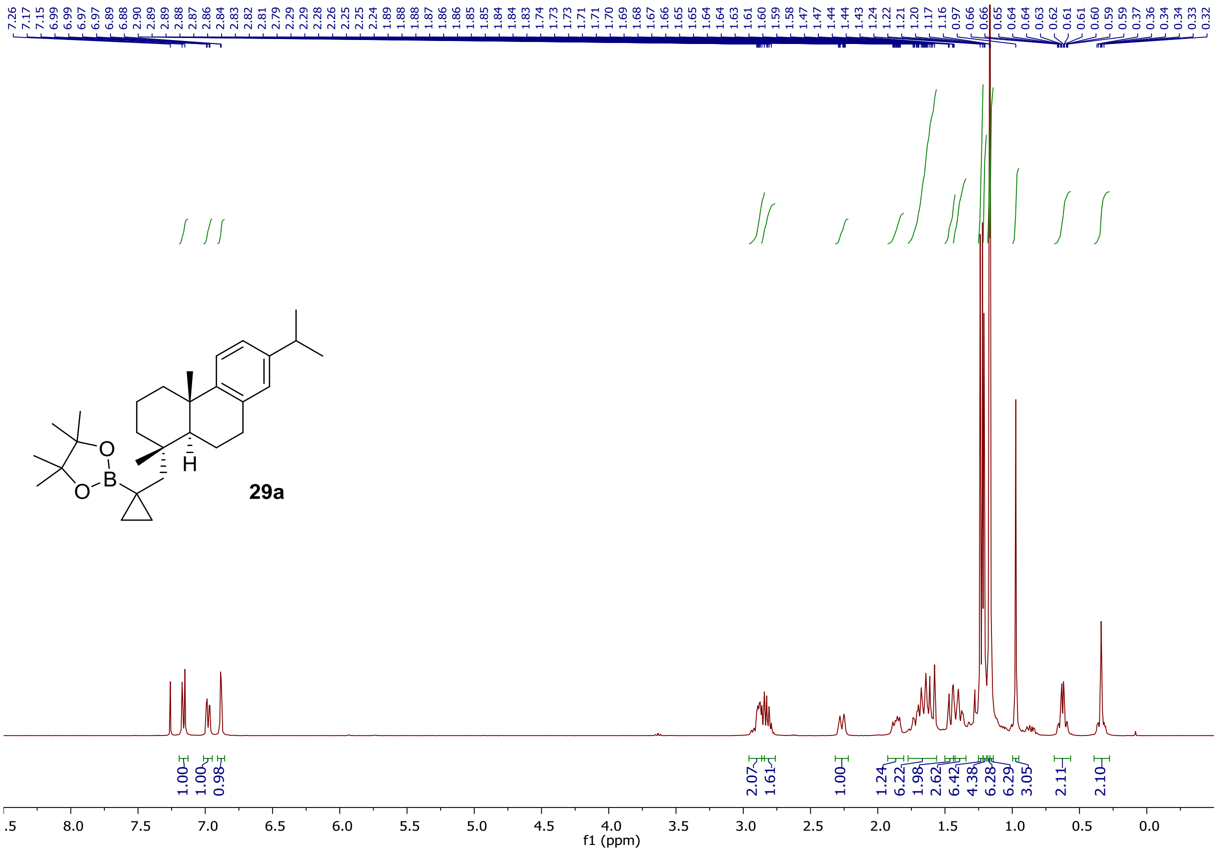

^{13}C NMR (101 MHz, $CDCl_3$)

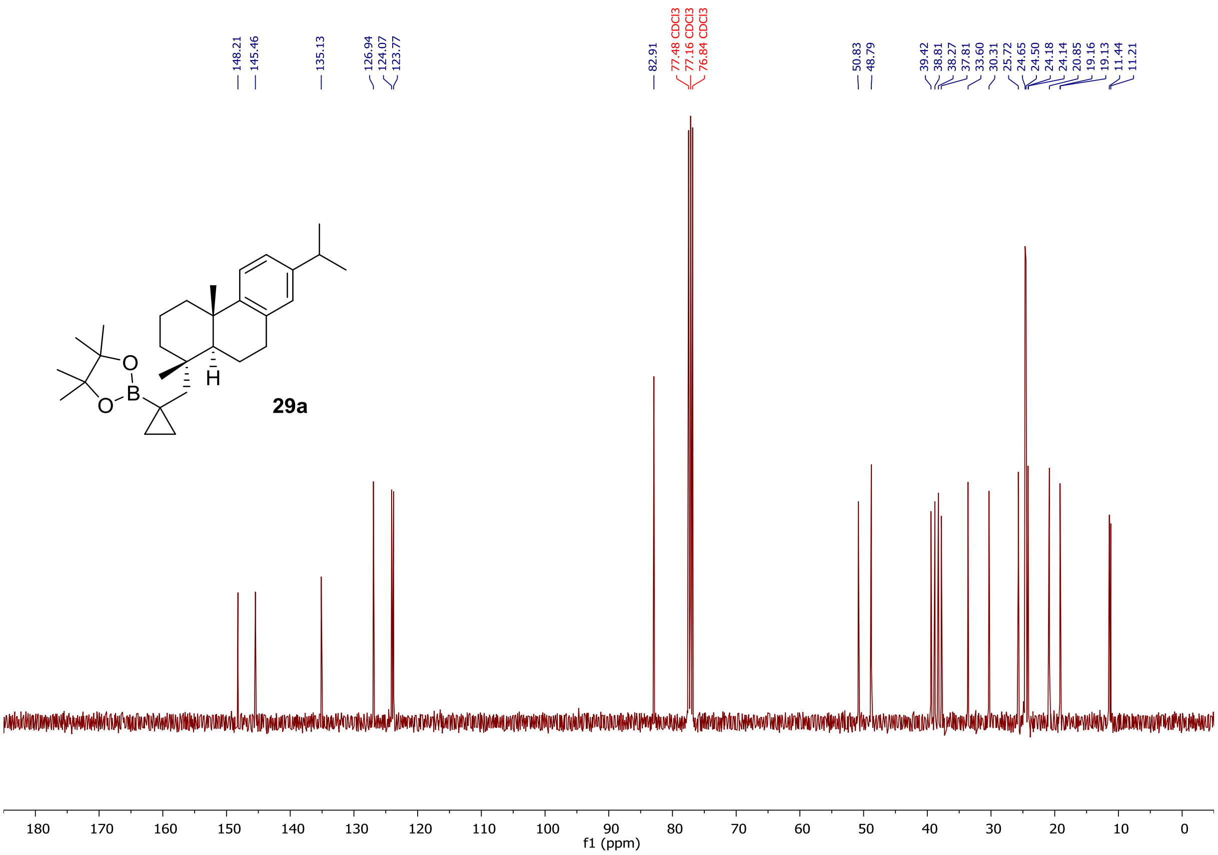

^{1}H NMR (400 MHz, $CDCl_3$)

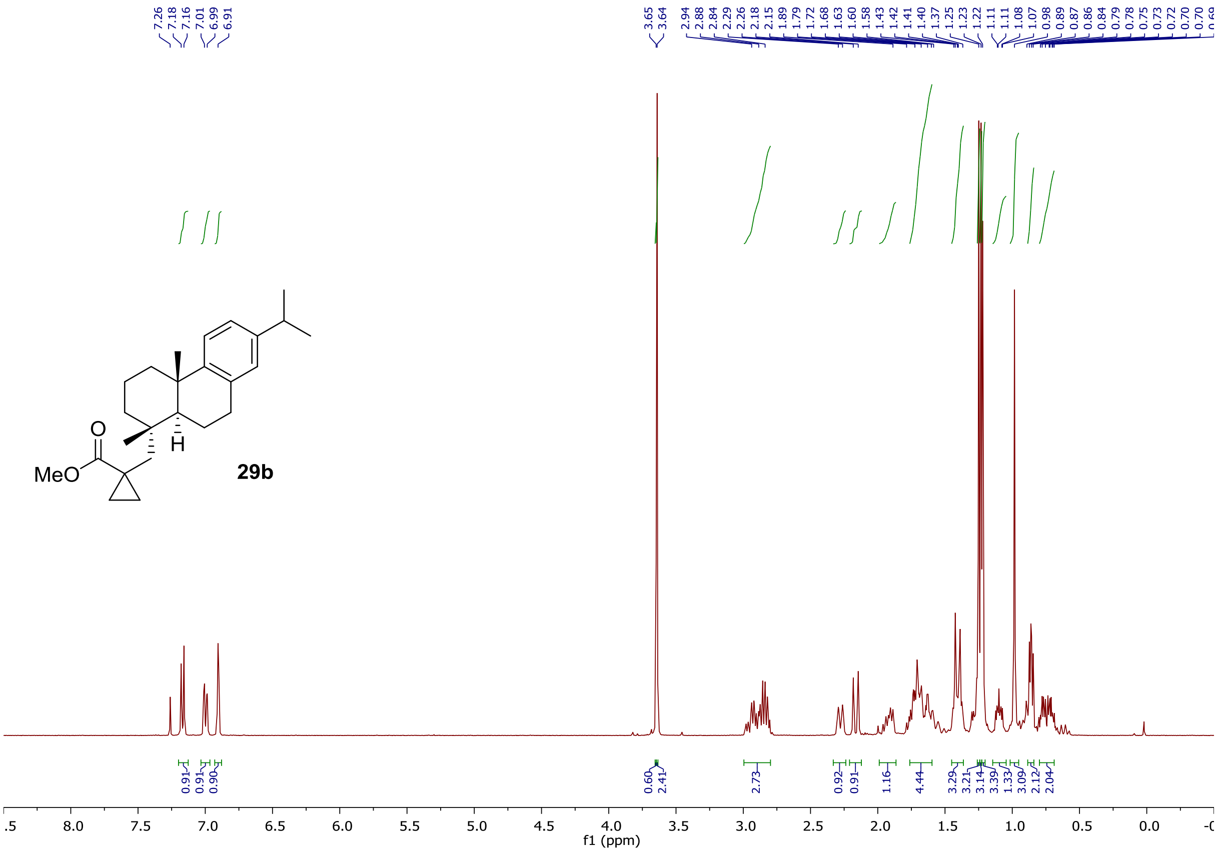

^{13}C NMR (121 MHz, DMSO-d_6, 100 °C)

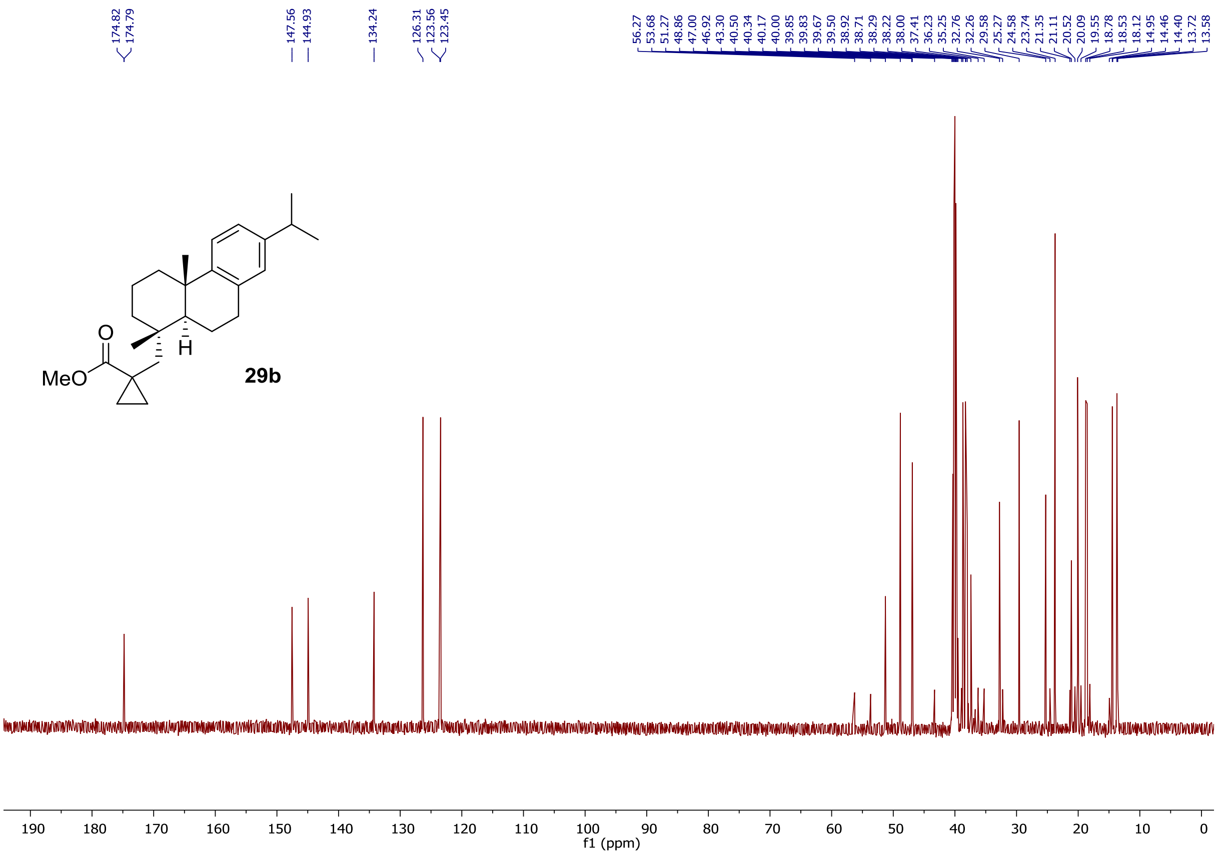

^{1}H NMR (400 MHz, $CDCl_3$)

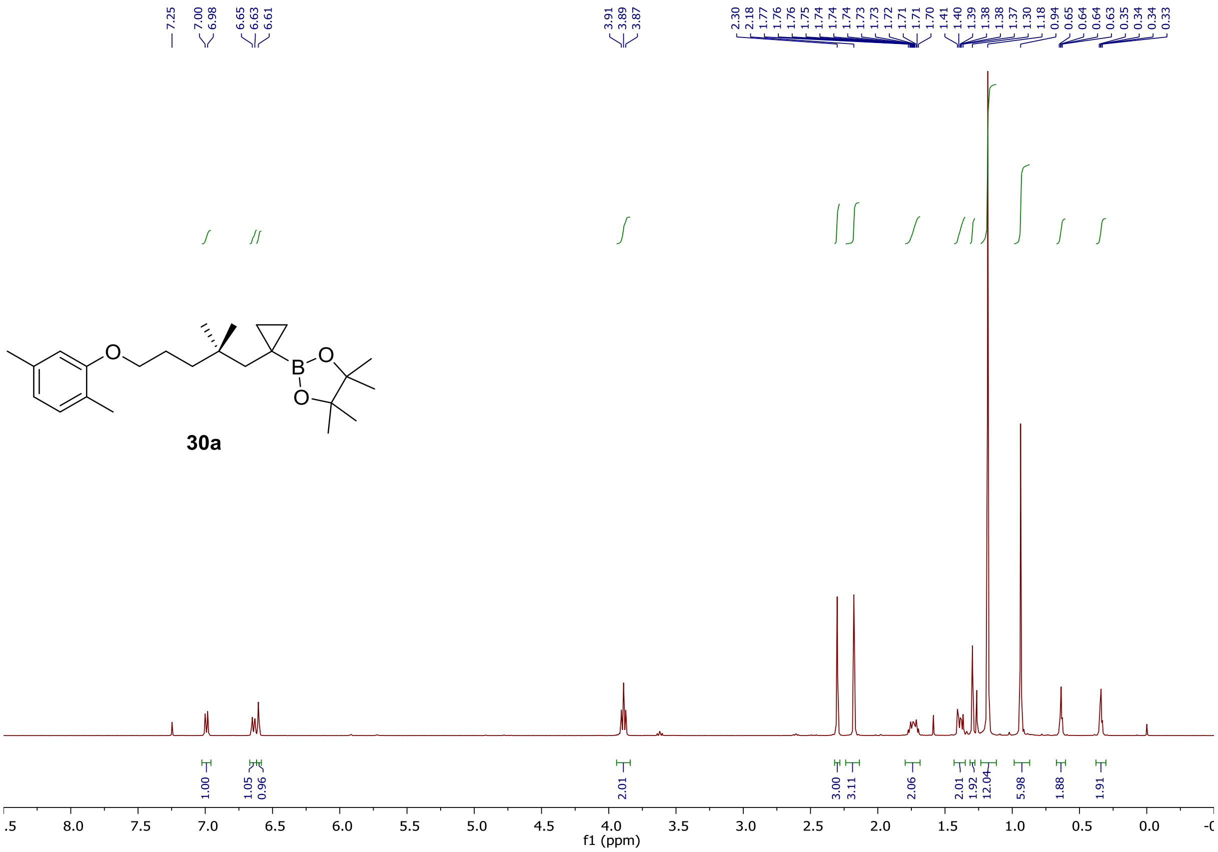

^{13}C NMR (101 MHz, $CDCl_3$)

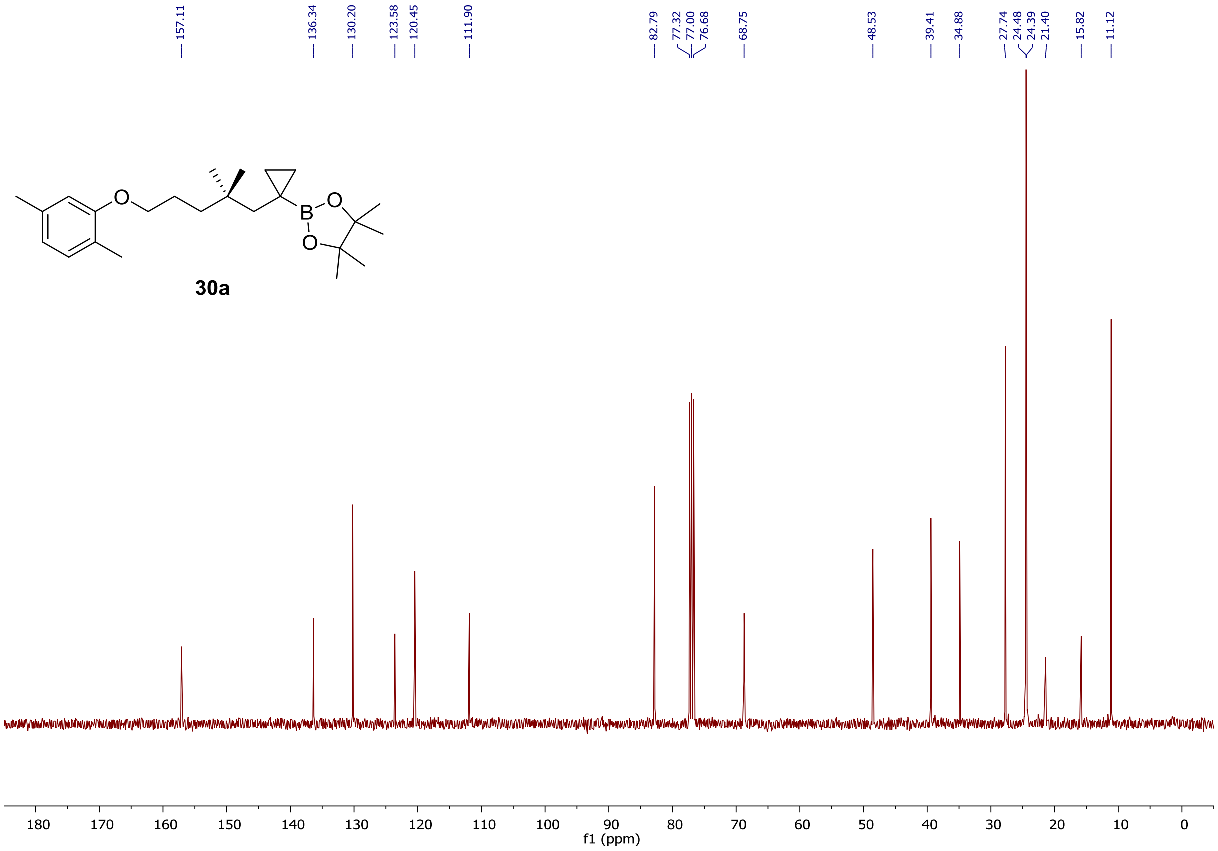

^{1}H NMR (400 MHz, $CDCl_3$)

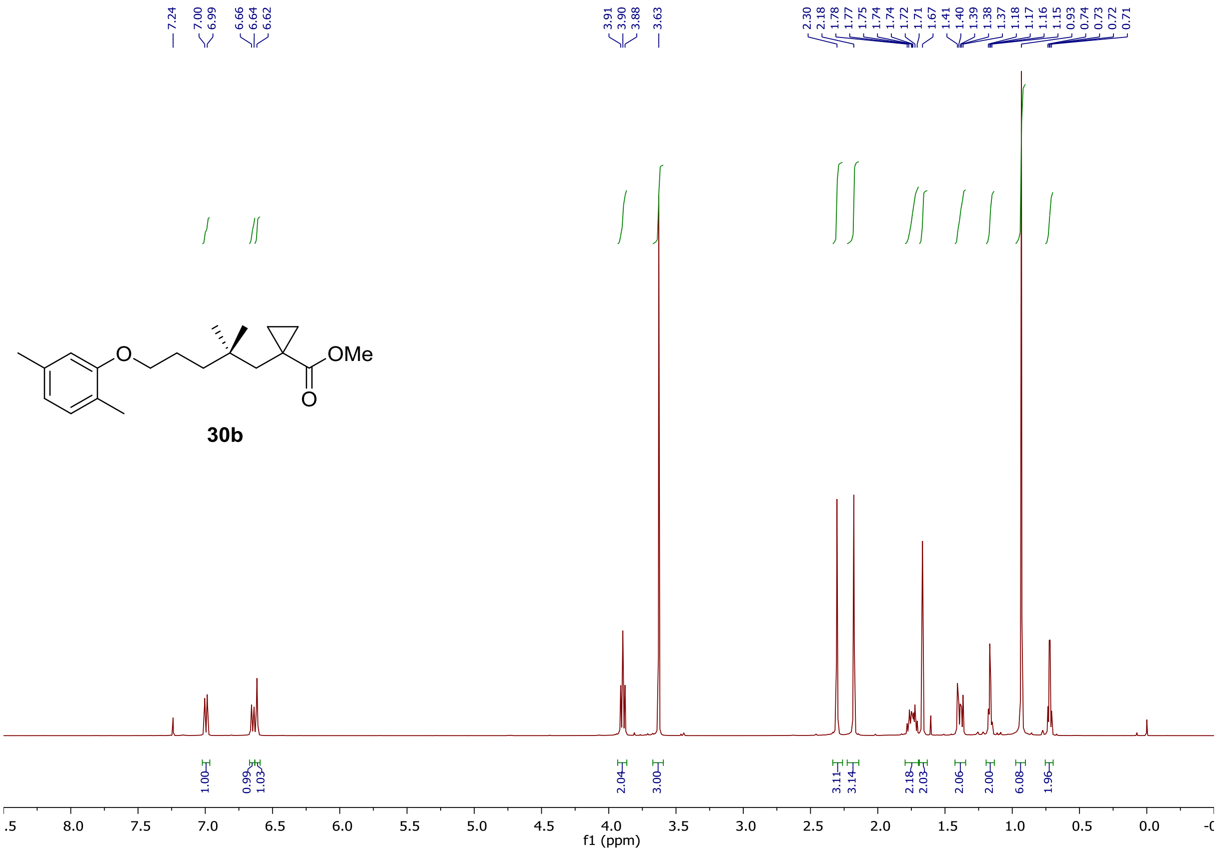

^{13}C NMR (101 MHz, $CDCl_3$)

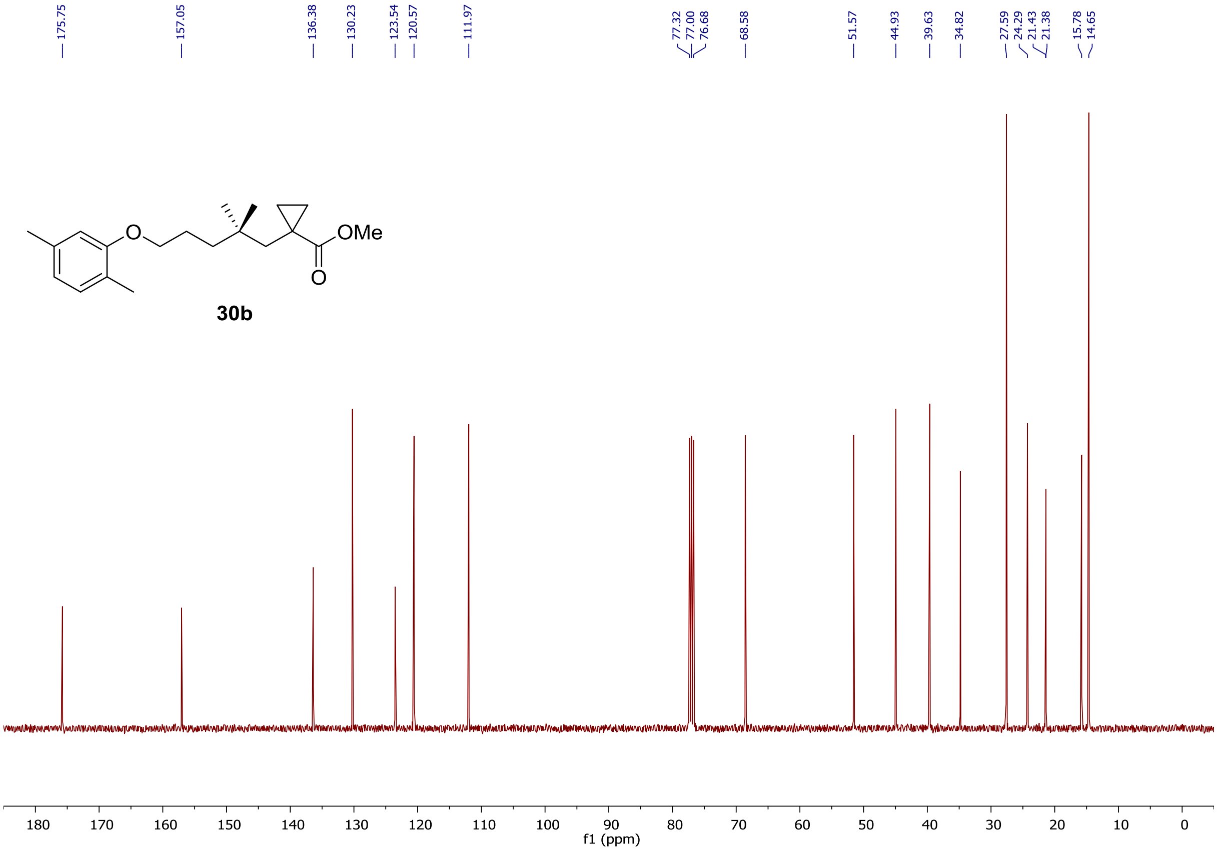

^{1}H NMR (400 MHz, $CDCl_3$)

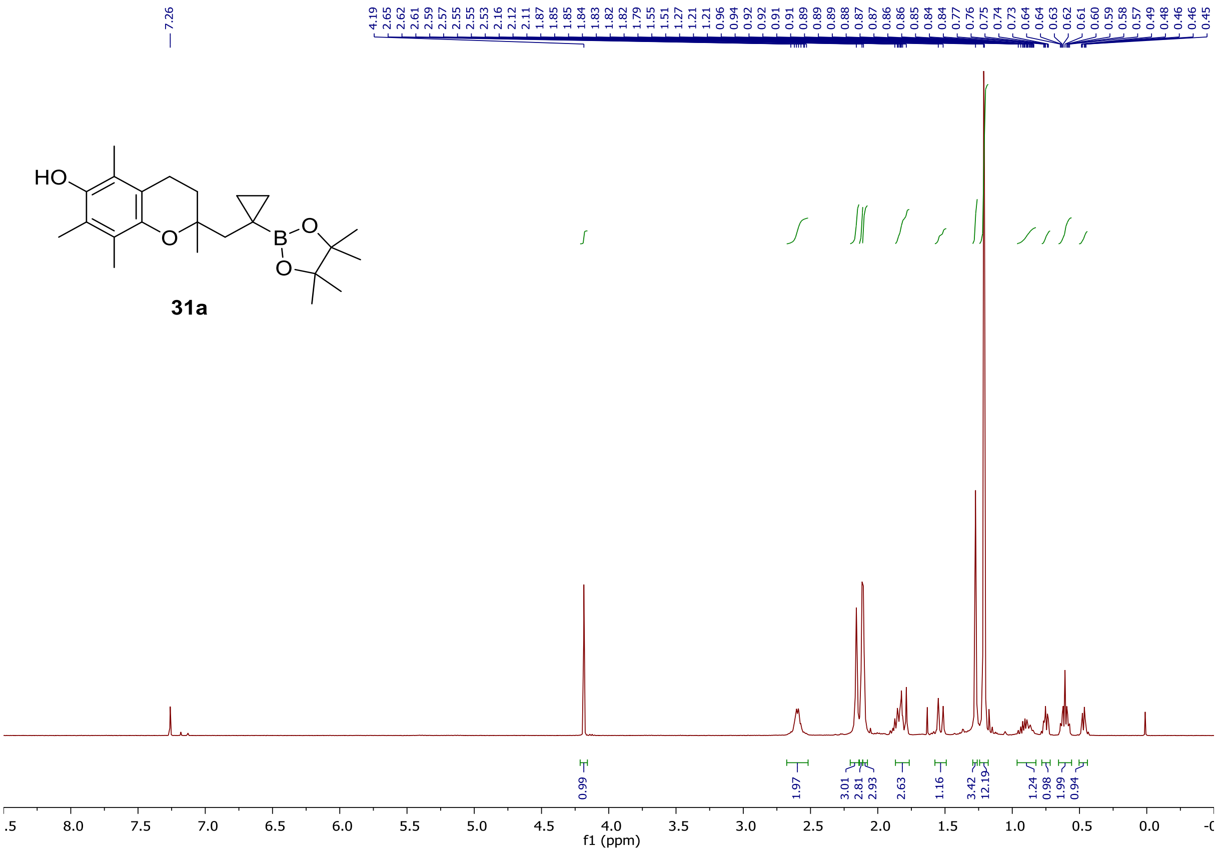

31a

^{13}C NMR (101 MHz, $CDCl_3$)

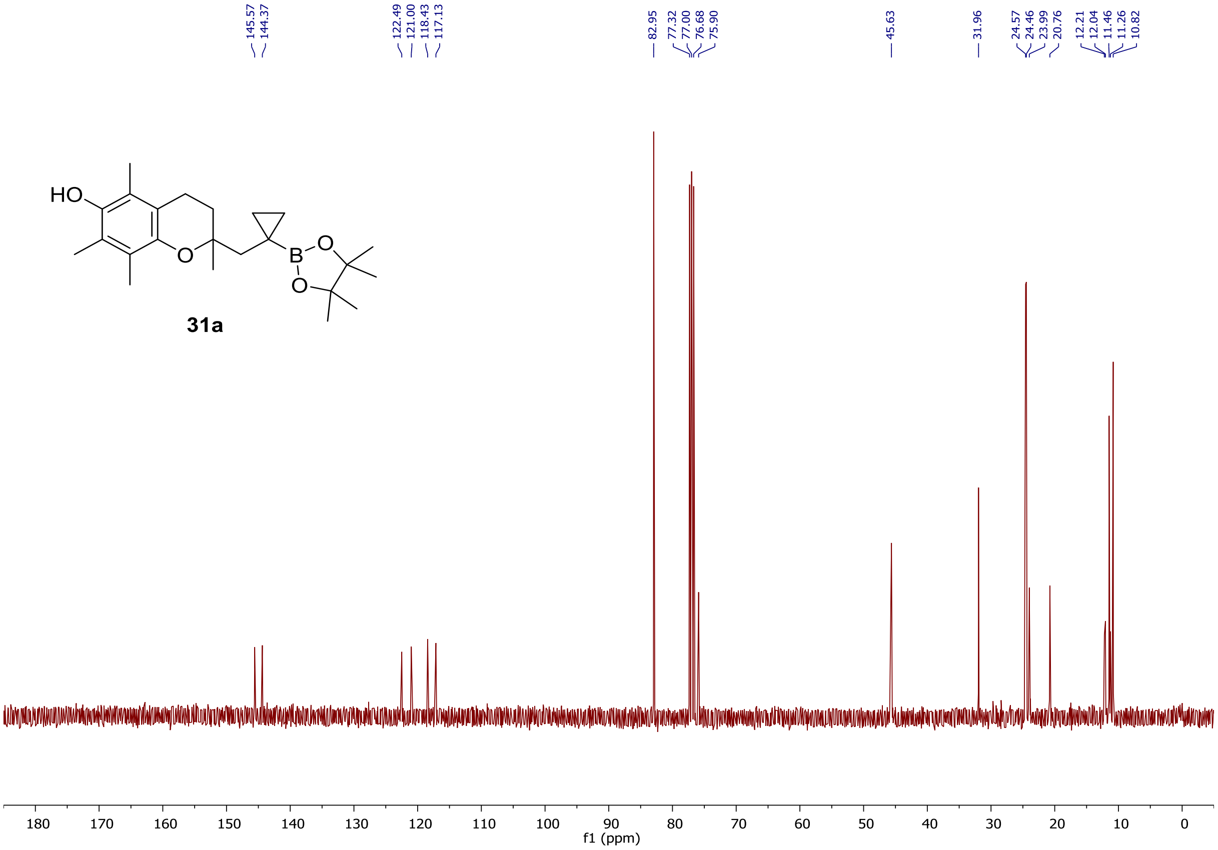

31a

^{1}H NMR (400 MHz, $CDCl_3$)

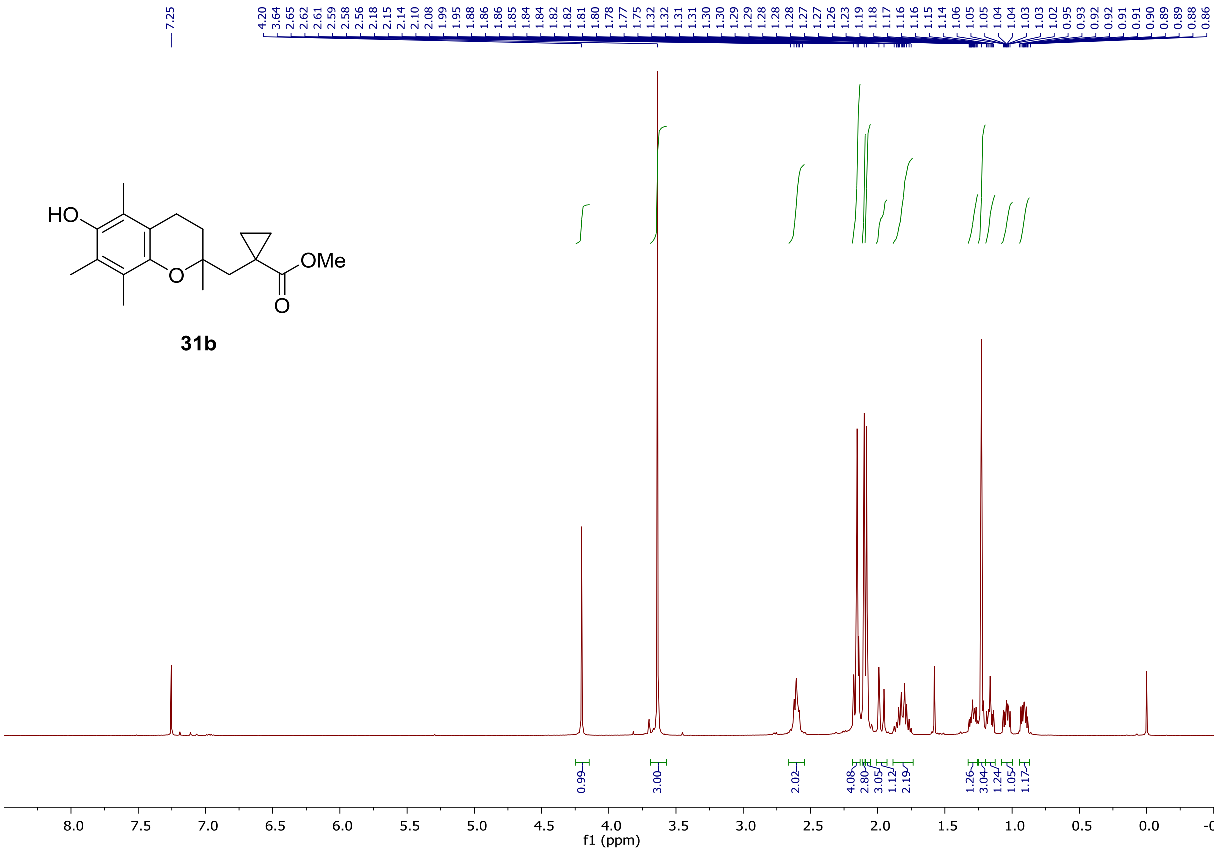

^{13}C NMR (101 MHz, $CDCl_3$)

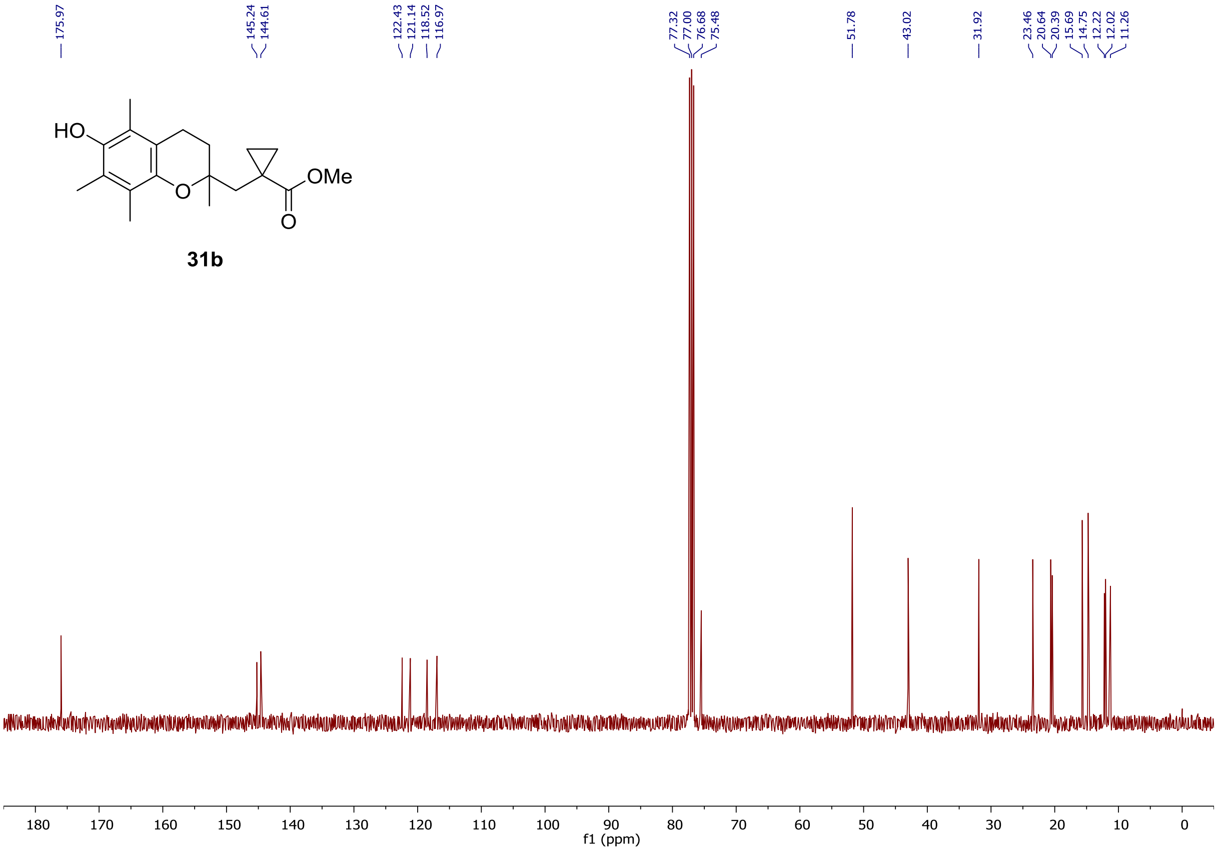

^{1}H NMR (400 MHz, $CDCl_3$)

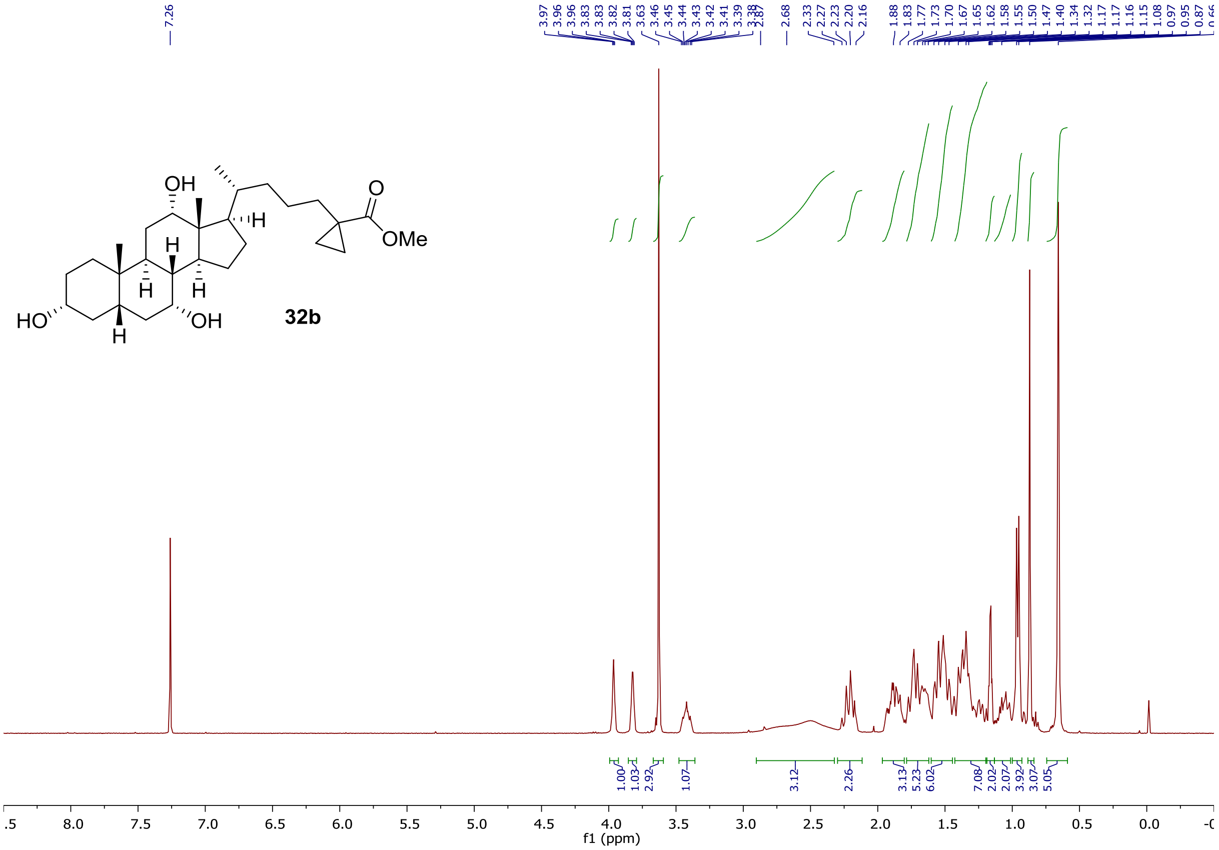

^{13}C NMR (101 MHz, $CDCl_3$)

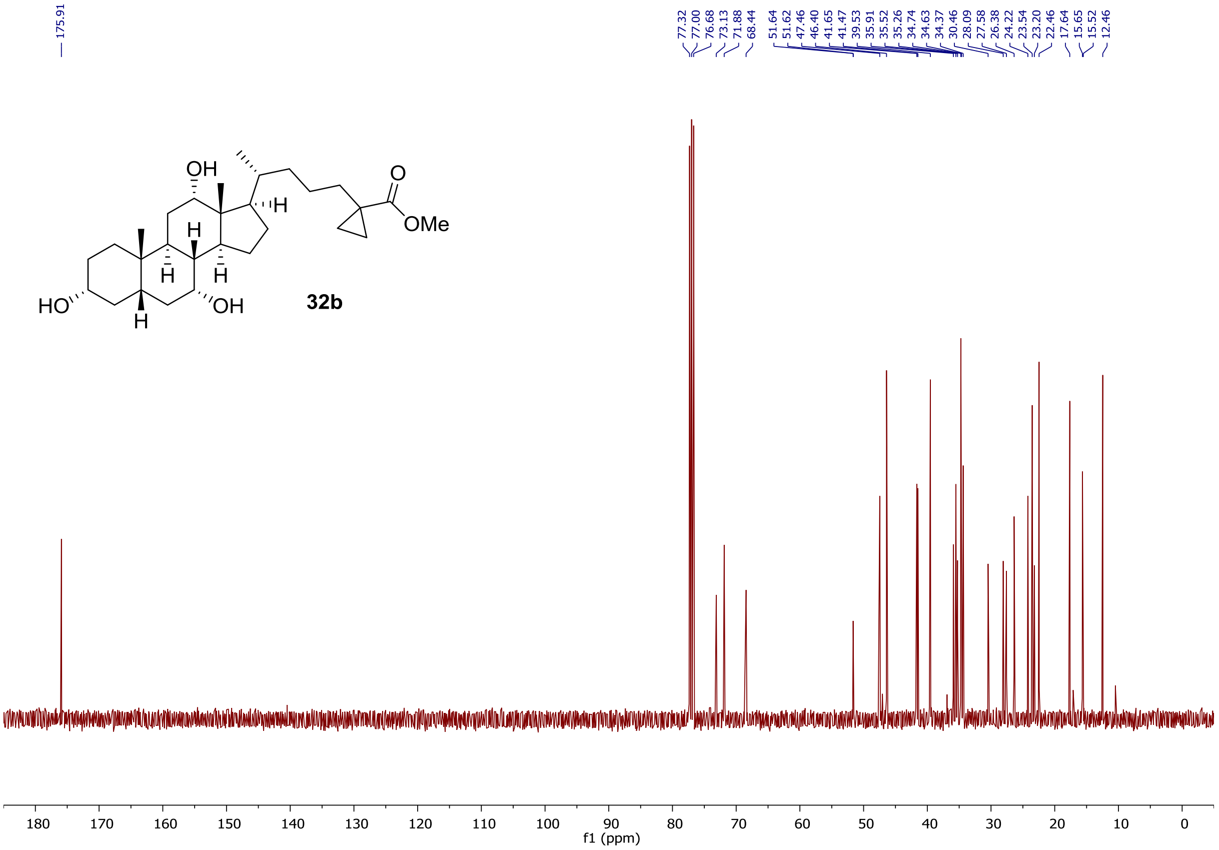

^{1}H NMR (400 MHz, $CDCl_3$)

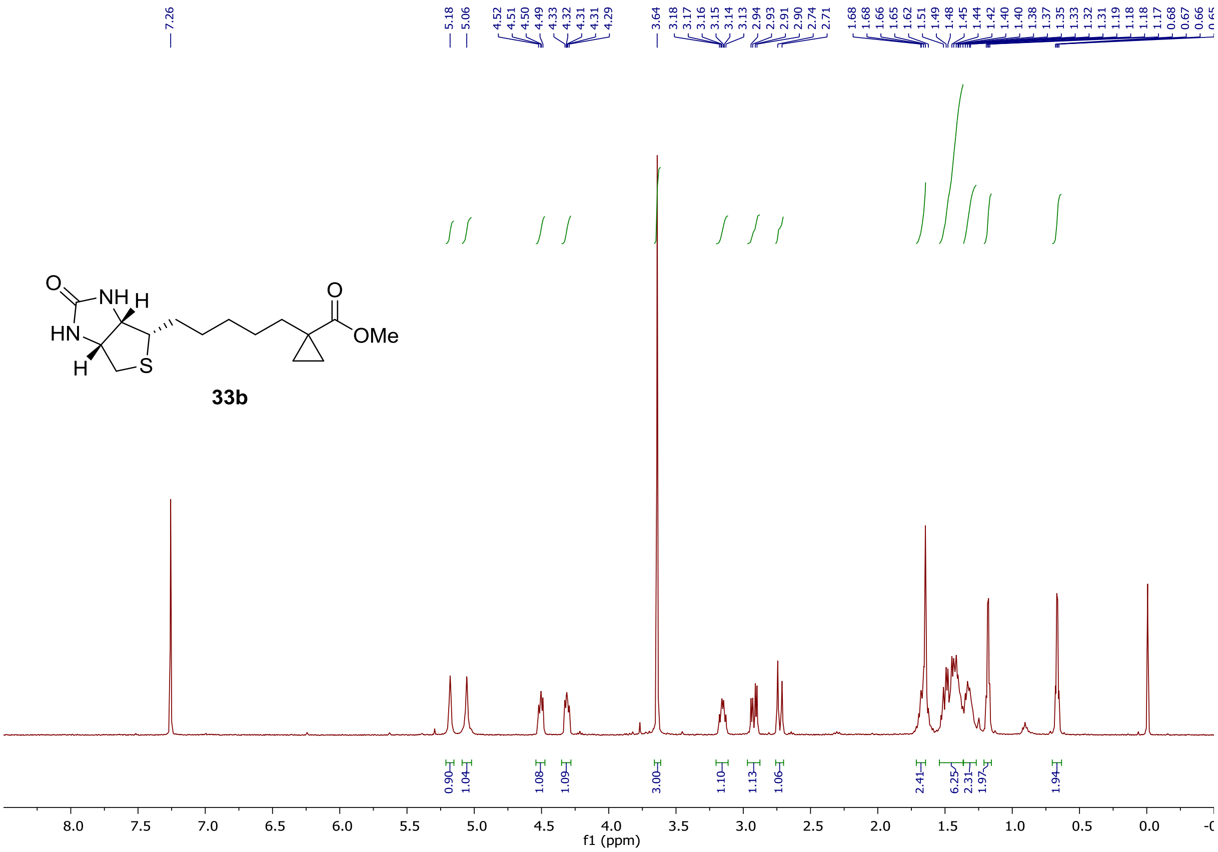

^{13}C NMR (101 MHz, $CDCl_3$)

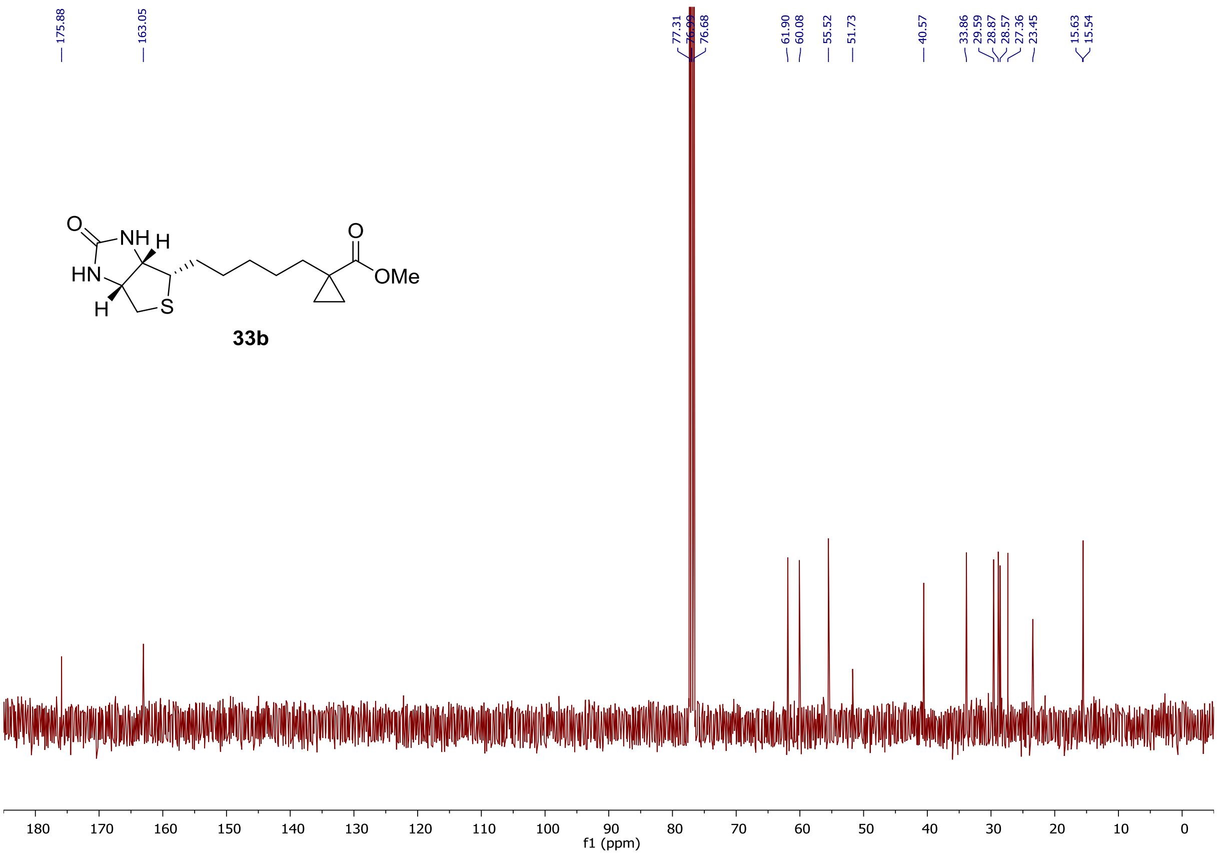

^{1}H NMR (400 MHz, $CDCl_3$)

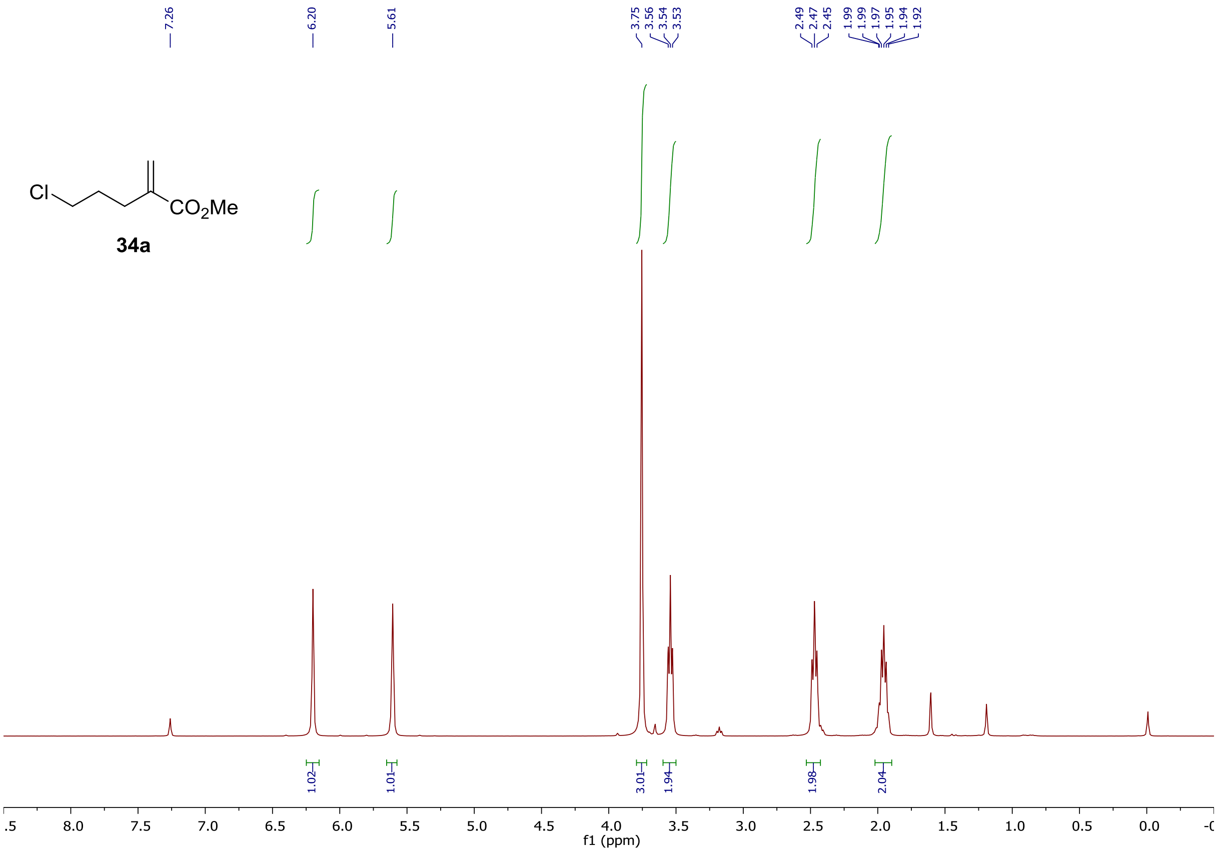

^{13}C NMR (101 MHz, $CDCl_3$)

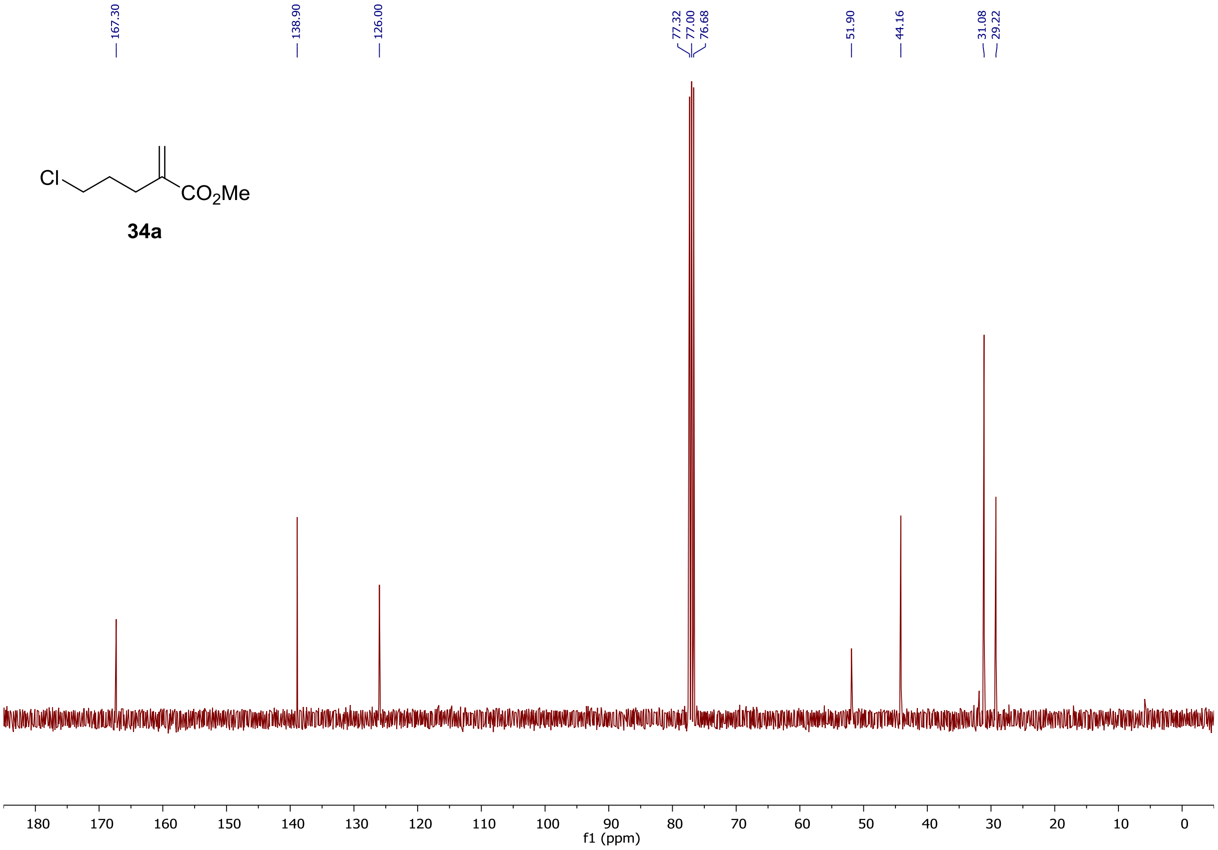

^{1}H NMR (400 MHz, $CDCl_3$)

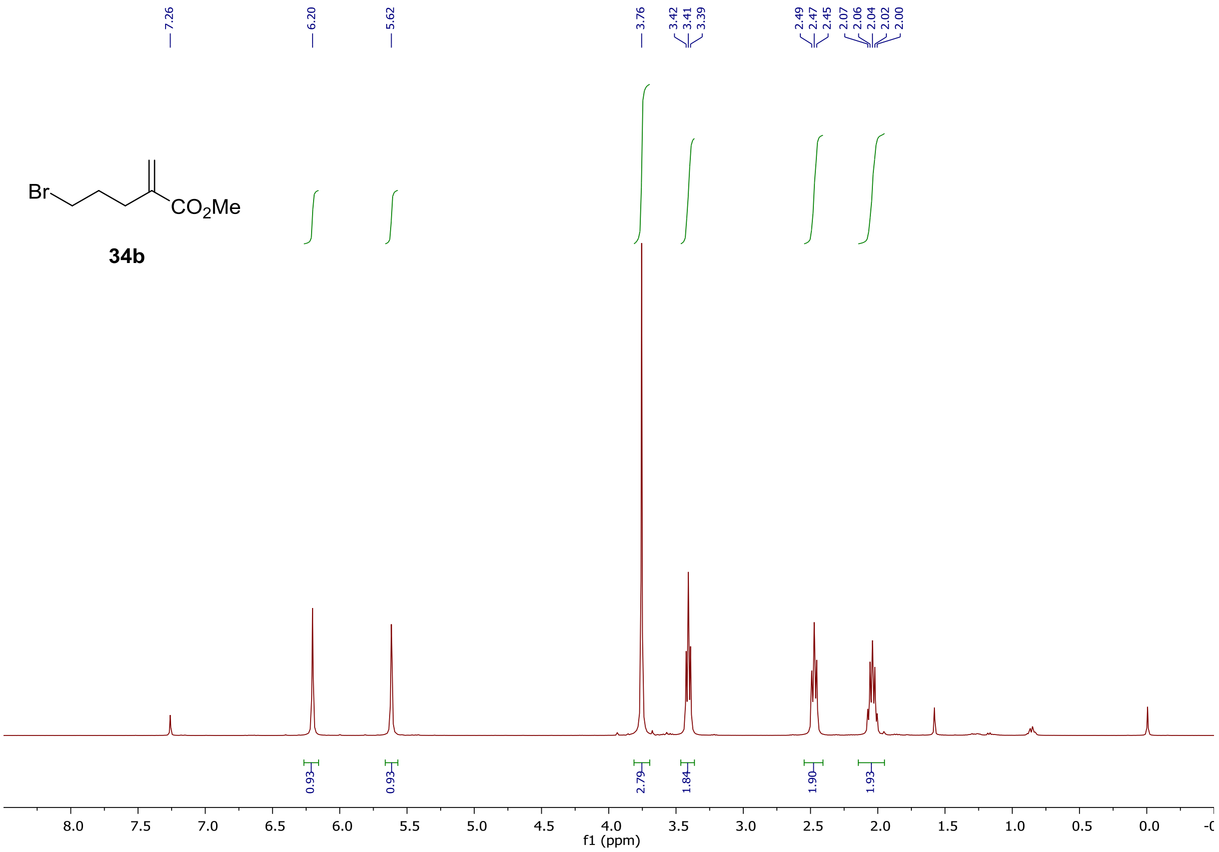

^{13}C NMR (101 MHz, $CDCl_3$)

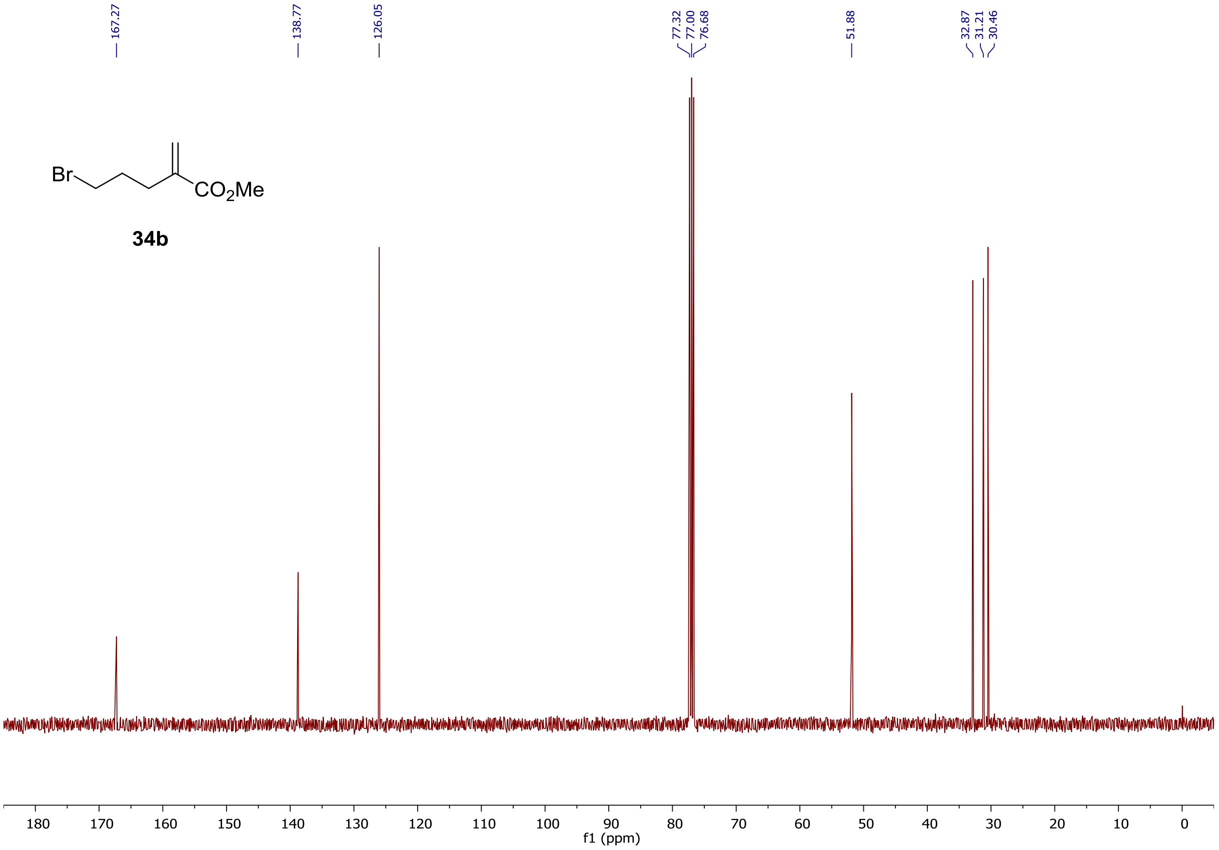

^{1}H NMR (400 MHz, $CDCl_3$)

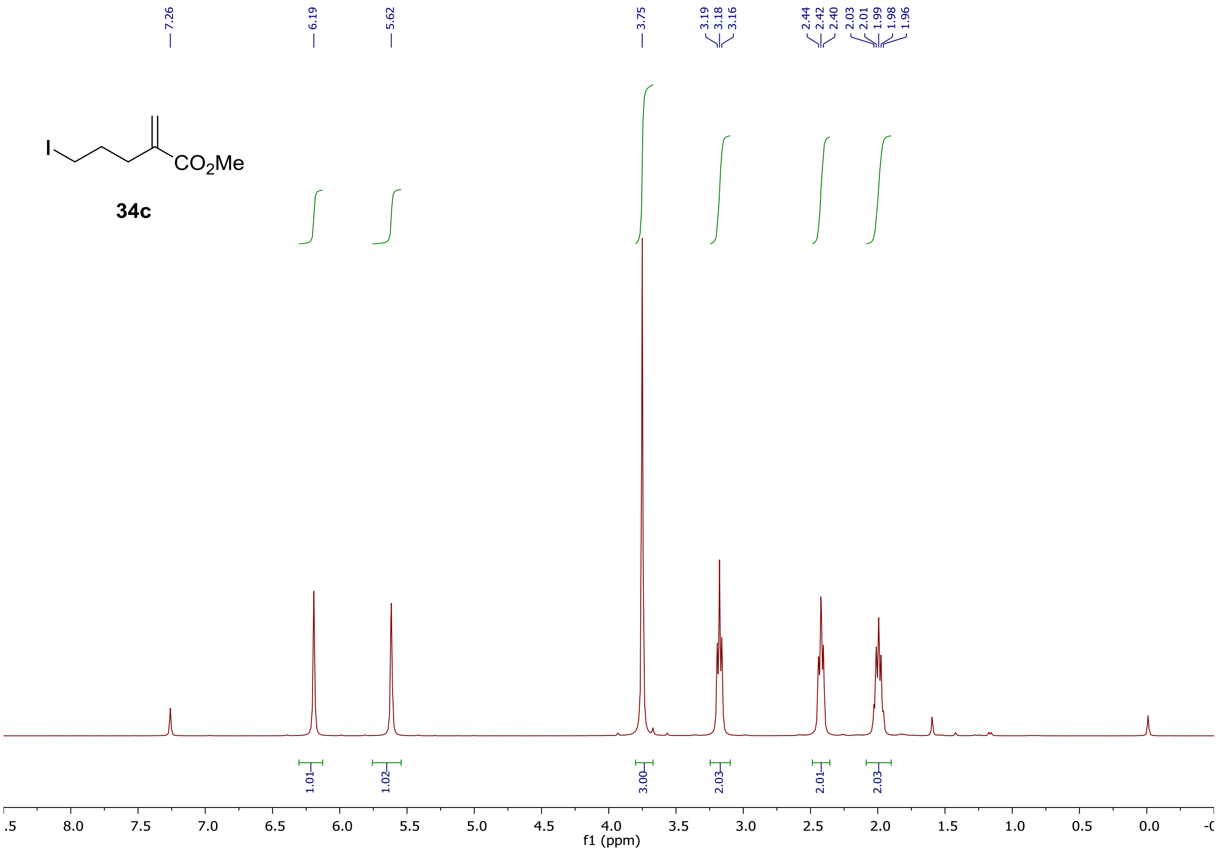

^{13}C NMR (101 MHz, $CDCl_3$)

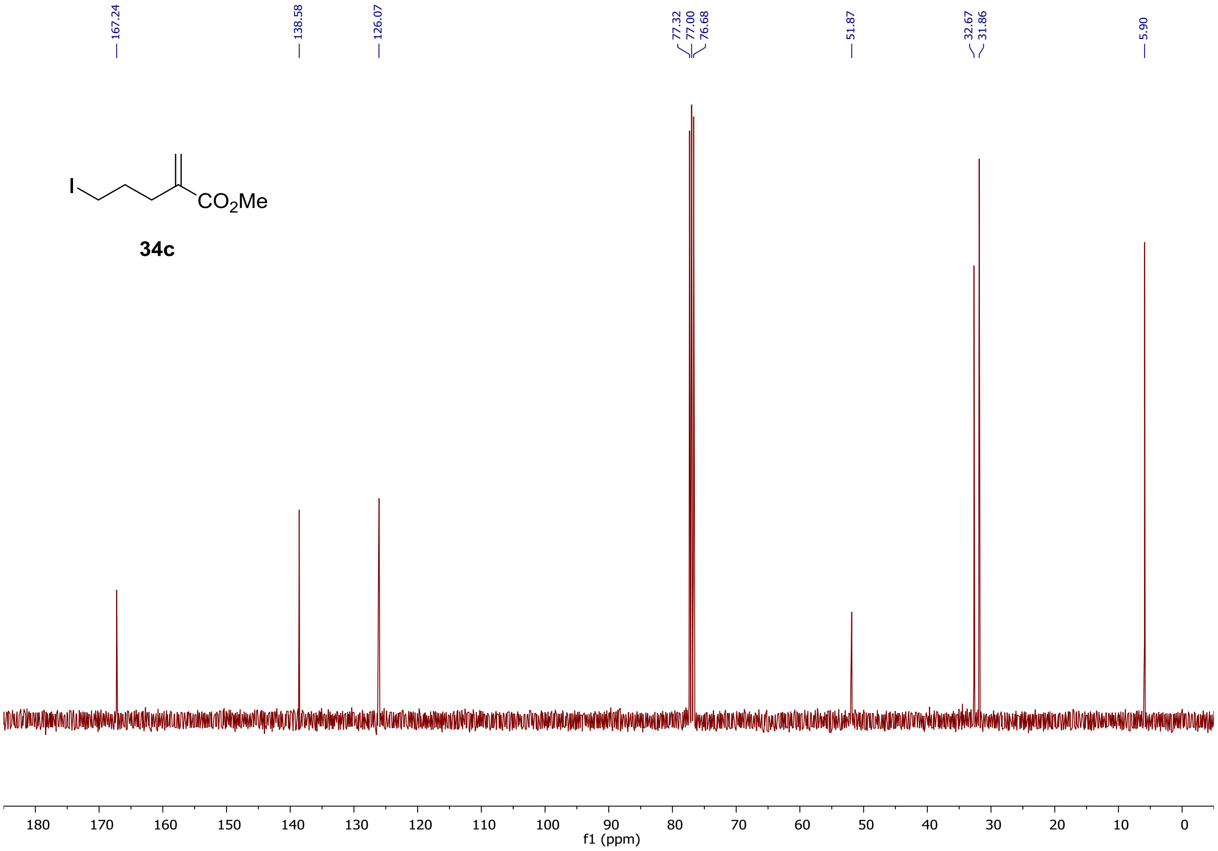

^{1}H NMR (400 MHz, $CDCl_3$)

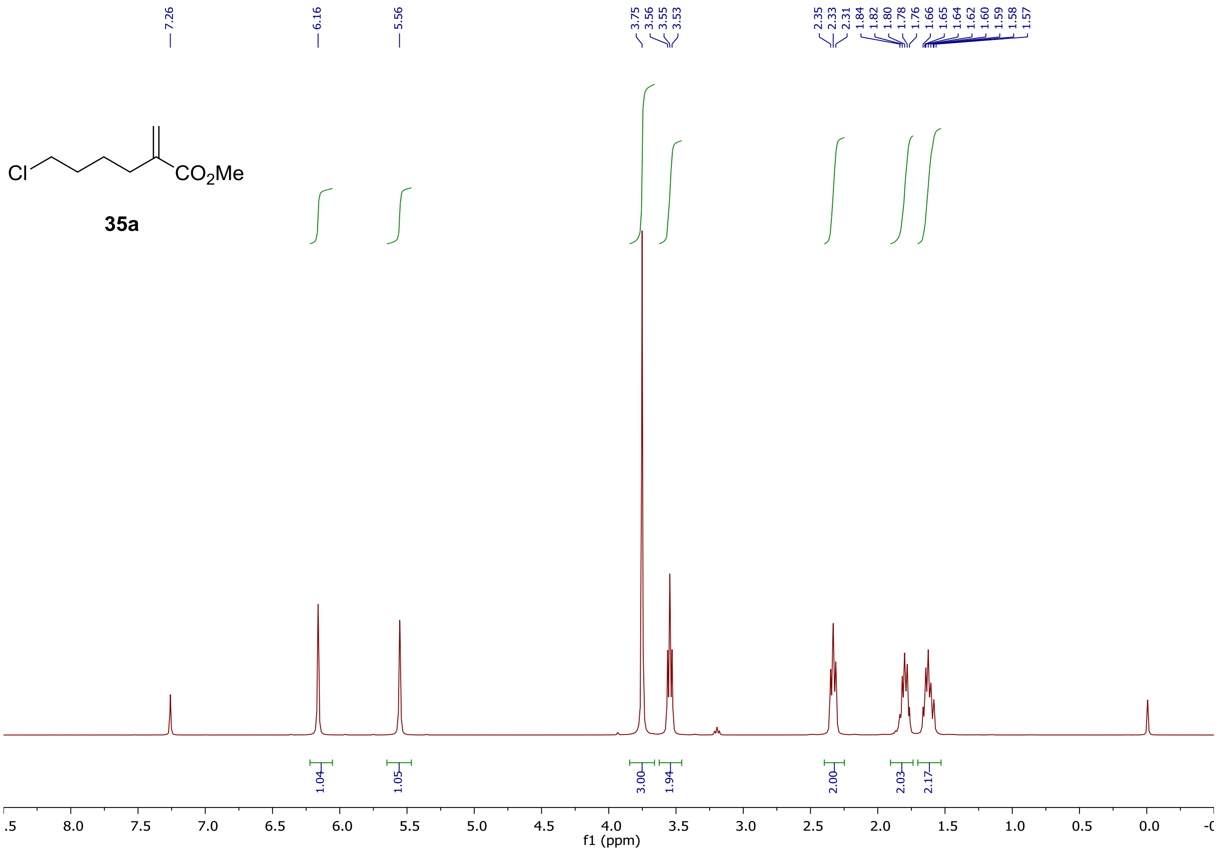

^{13}C NMR (101 MHz, $CDCl_3$)

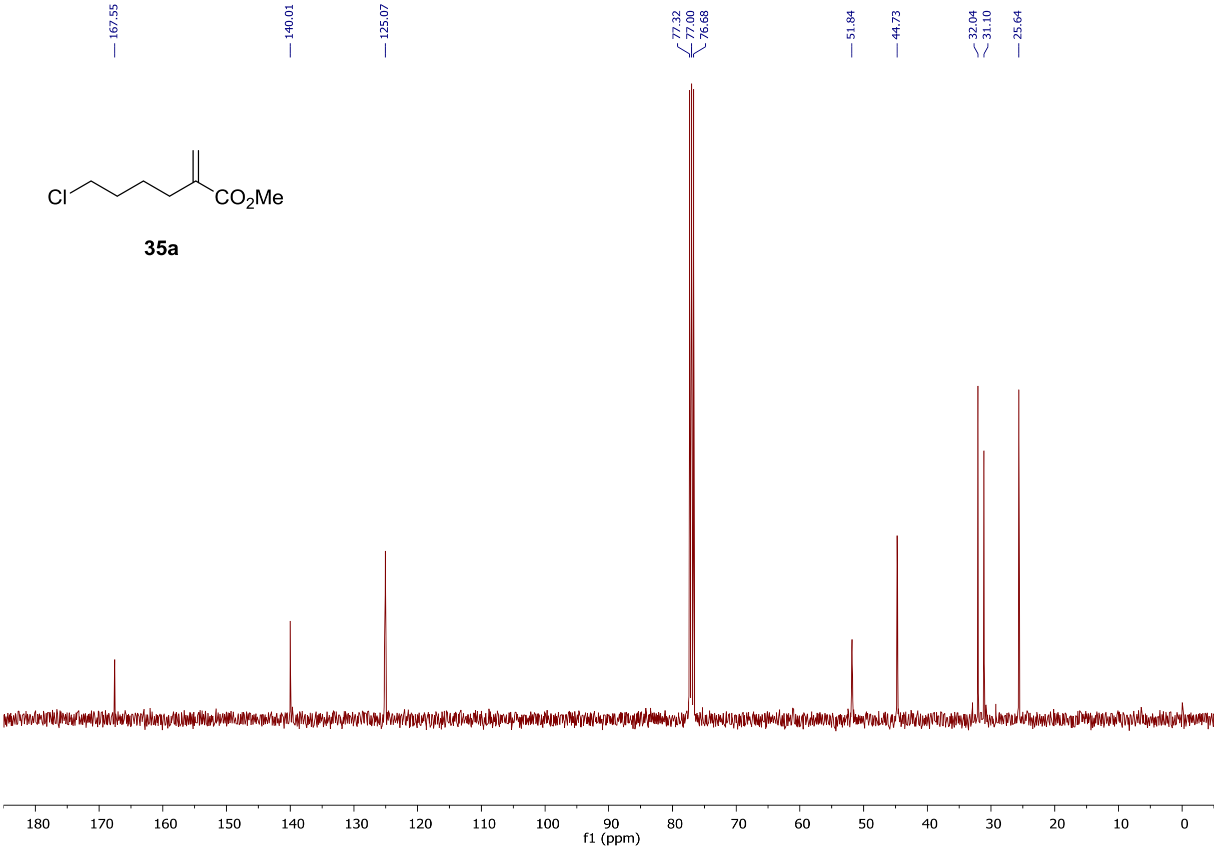

^{1}H NMR (400 MHz, $CDCl_3$)

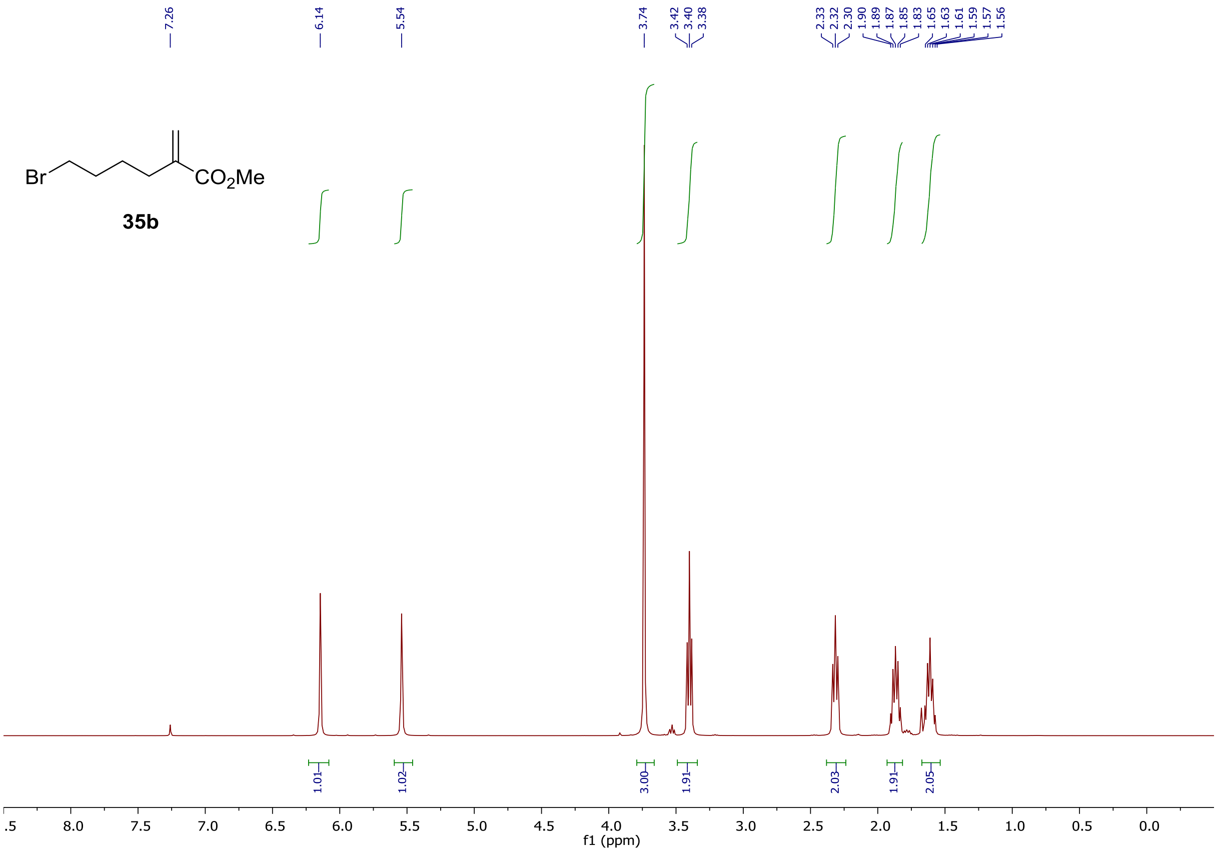

^{13}C NMR (101 MHz, $CDCl_3$)

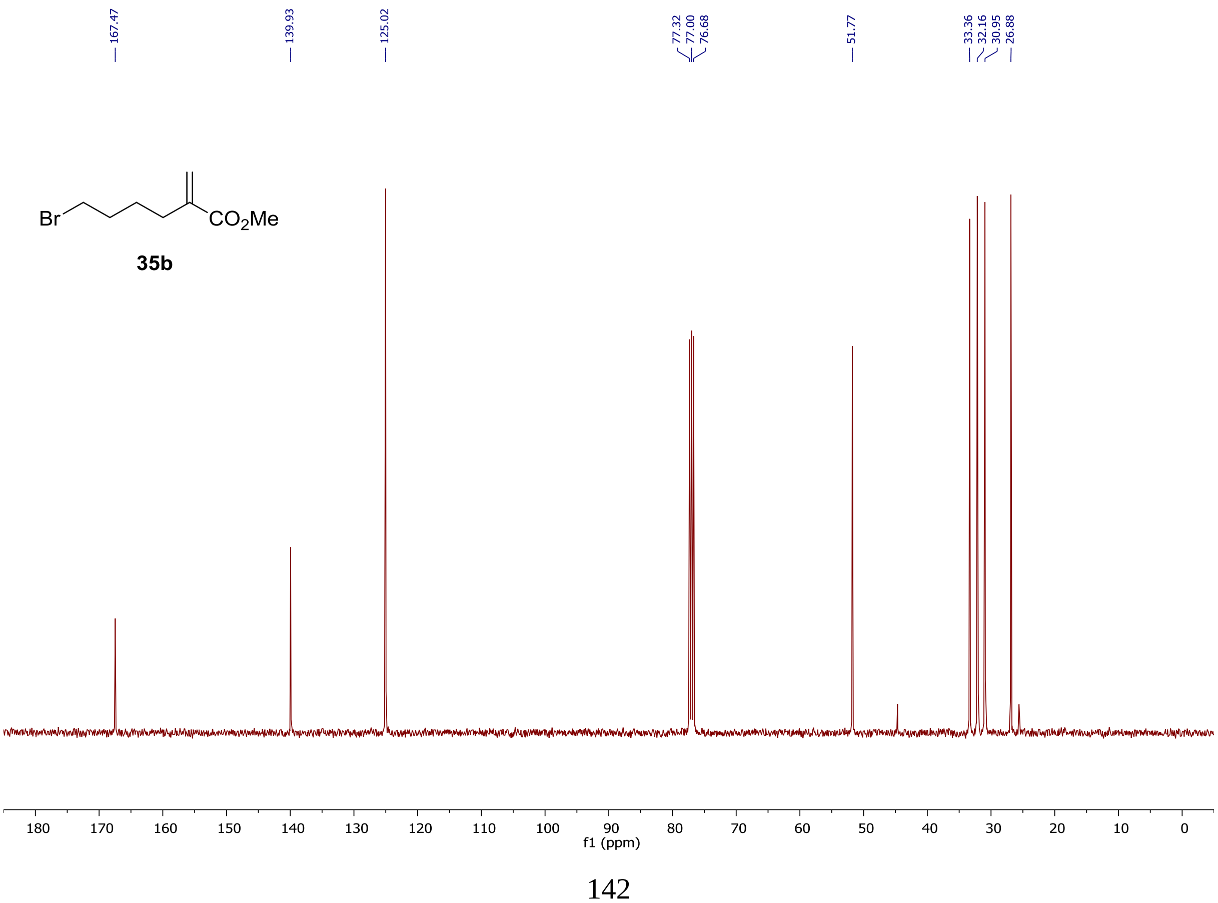

^{1}H NMR (400 MHz, $CDCl_3$)

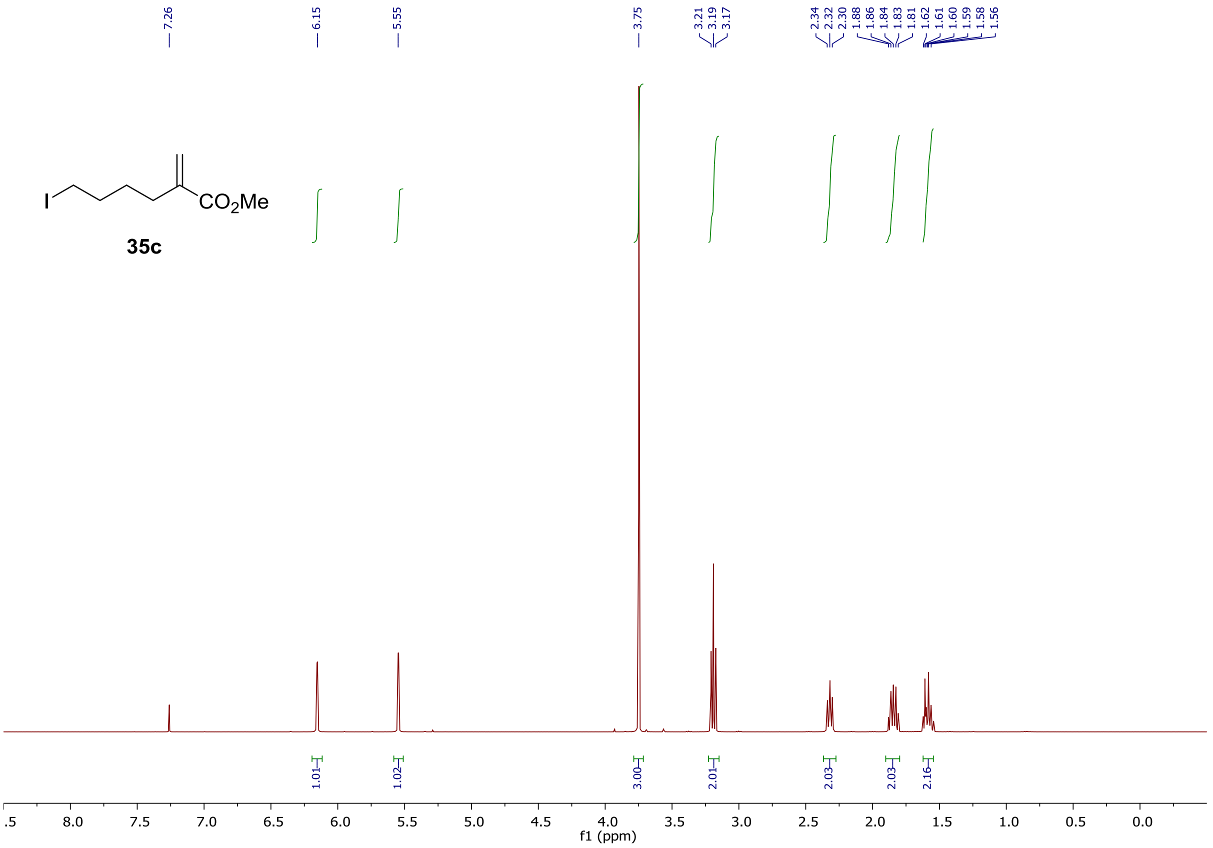

^{13}C NMR (101 MHz, $CDCl_3$)

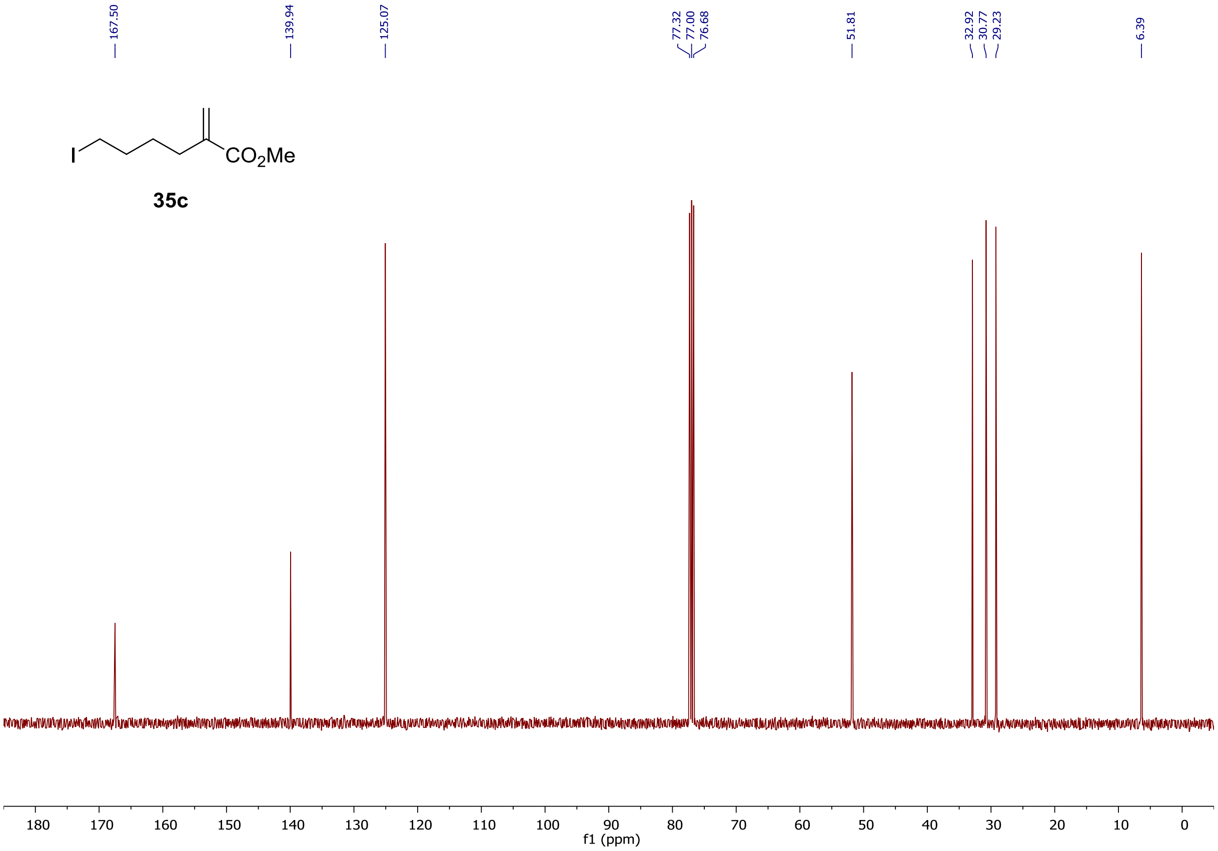

^{1}H NMR (400 MHz, $CDCl_3$)

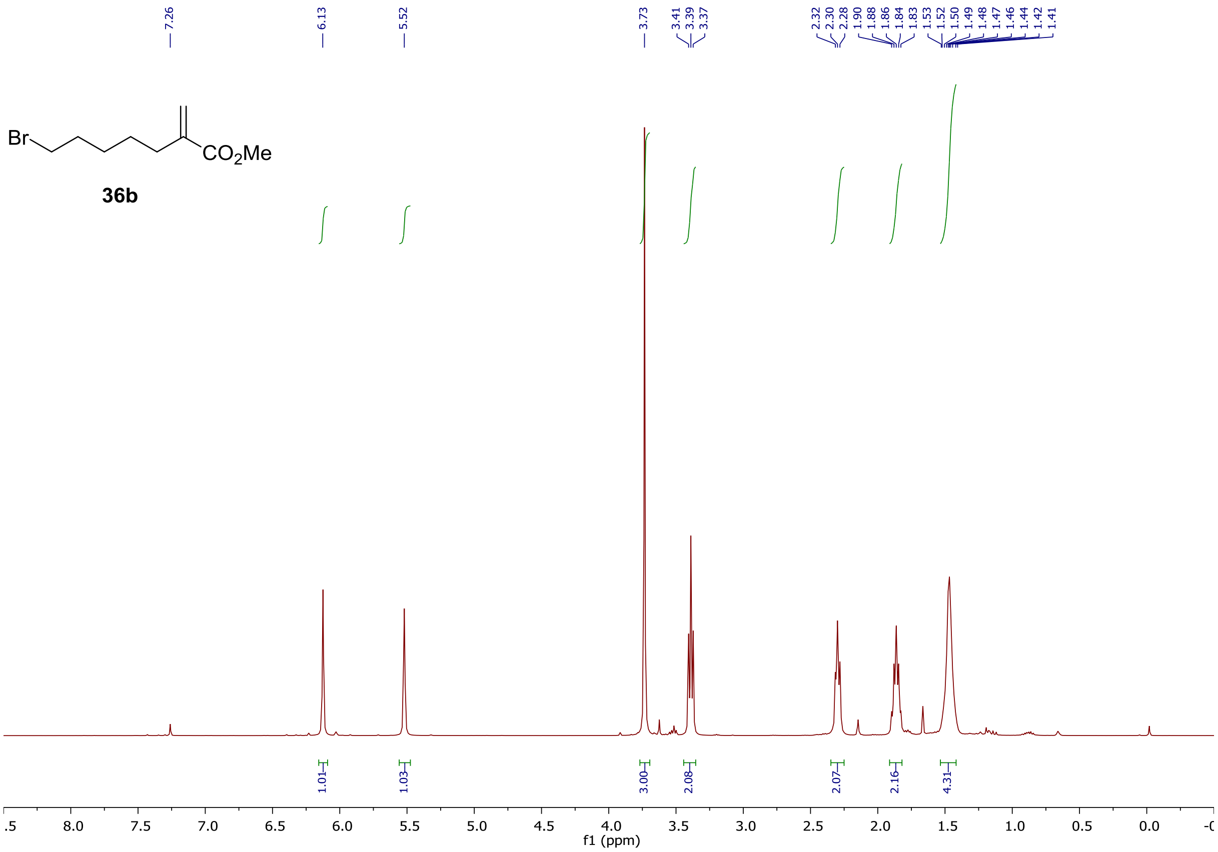

^{13}C NMR (101 MHz, $CDCl_3$)

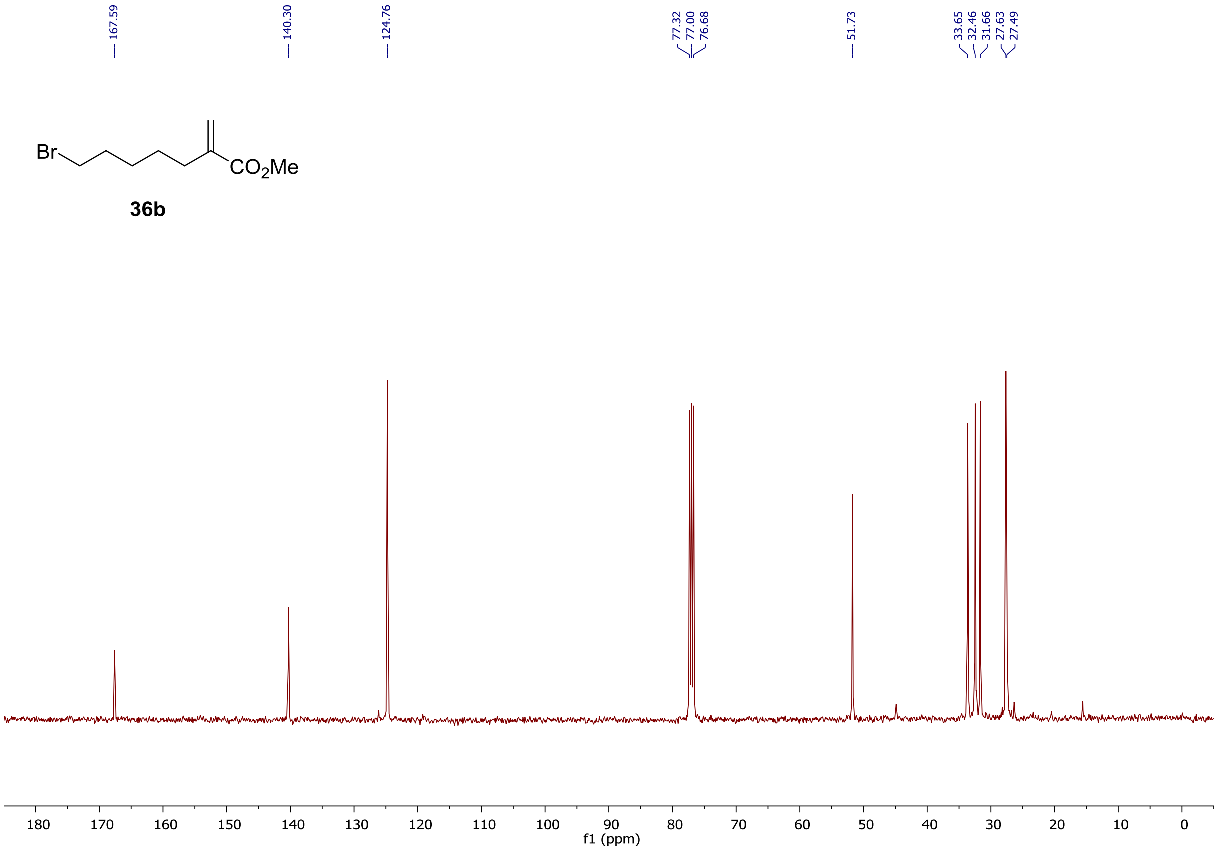

^{1}H NMR (400 MHz, $CDCl_3$)

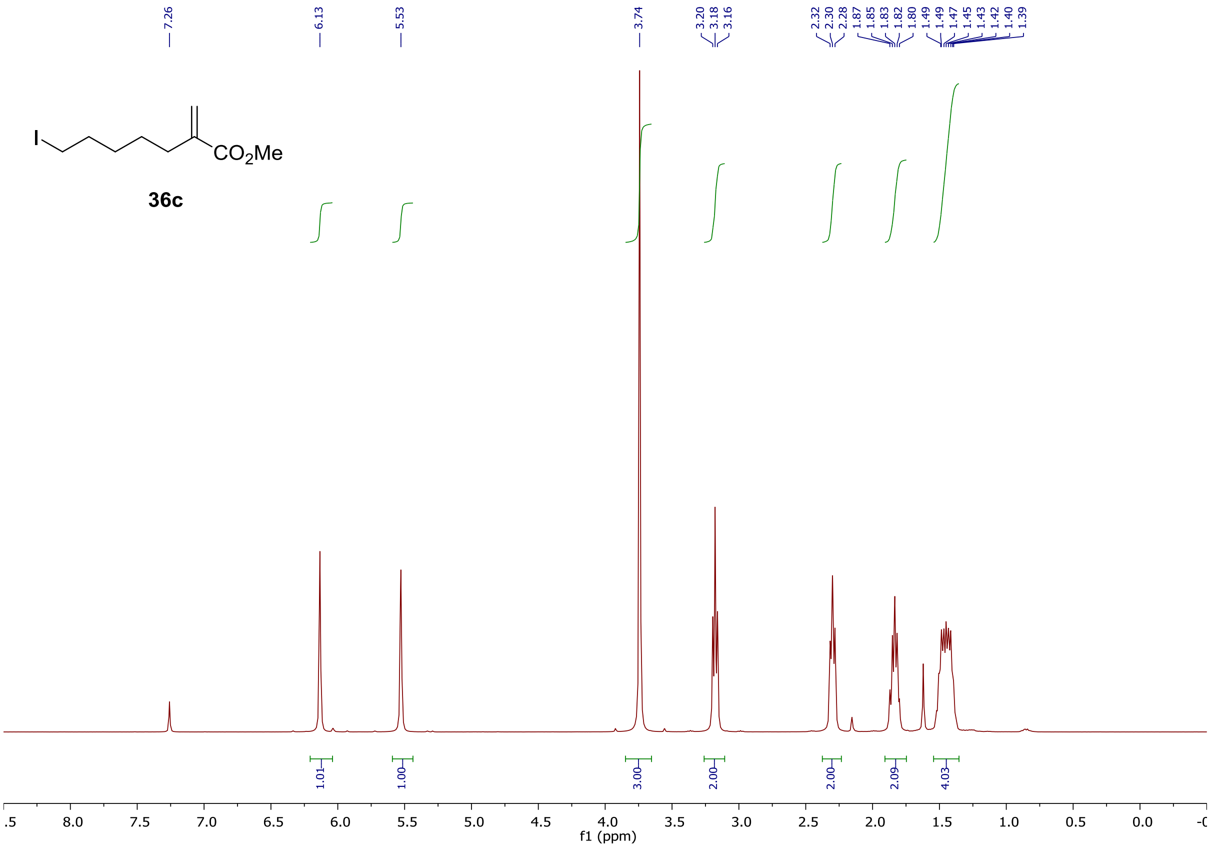

^{13}C NMR (101 MHz, $CDCl_3$)

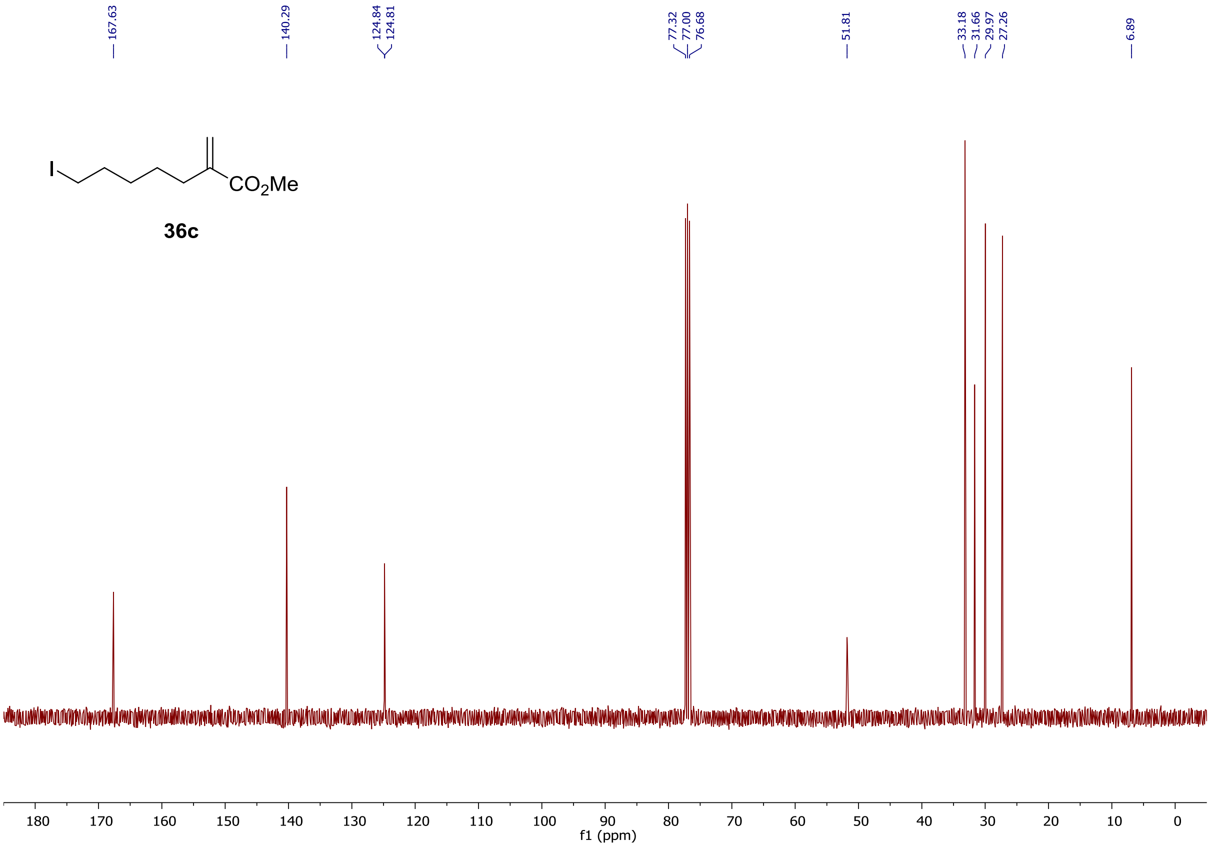

^{1}H NMR (400 MHz, DMSO-d_6)

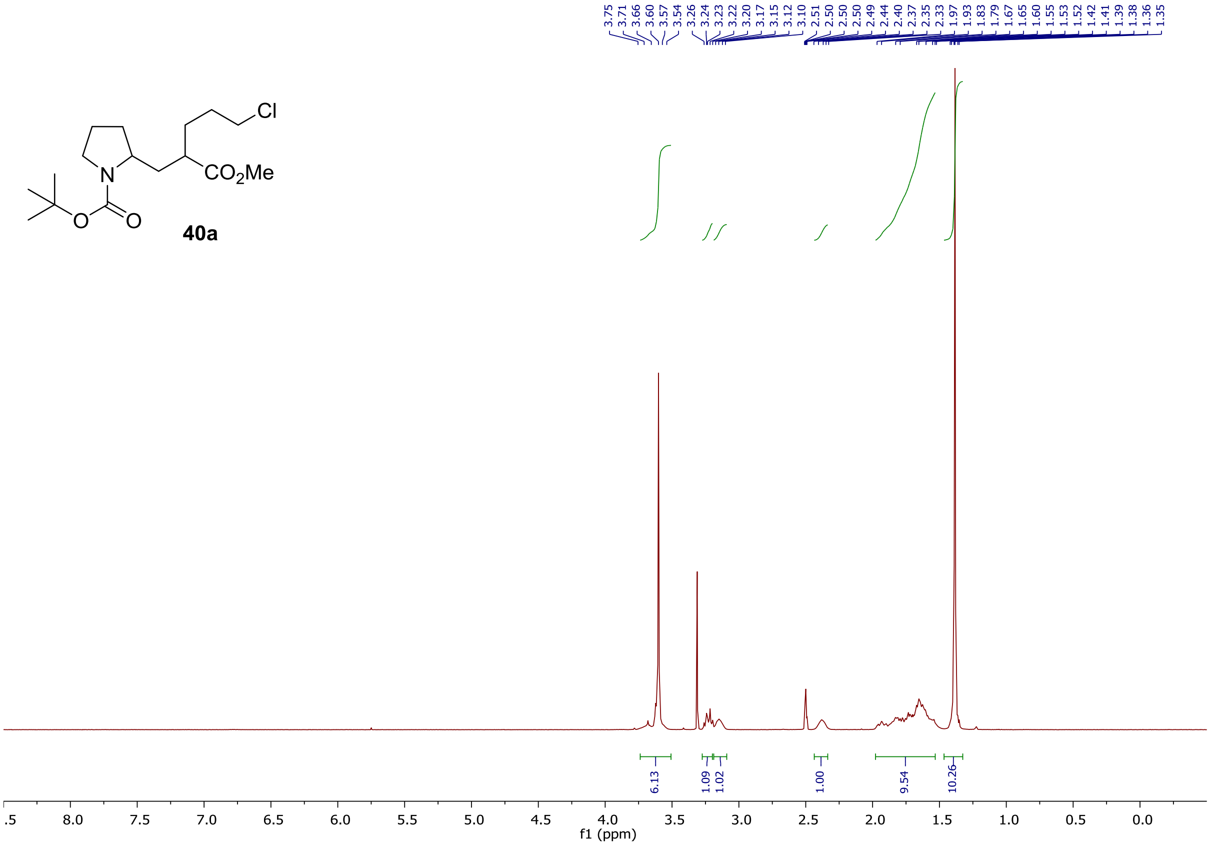

^{13}C NMR (101 MHz, DMSO-d_6)

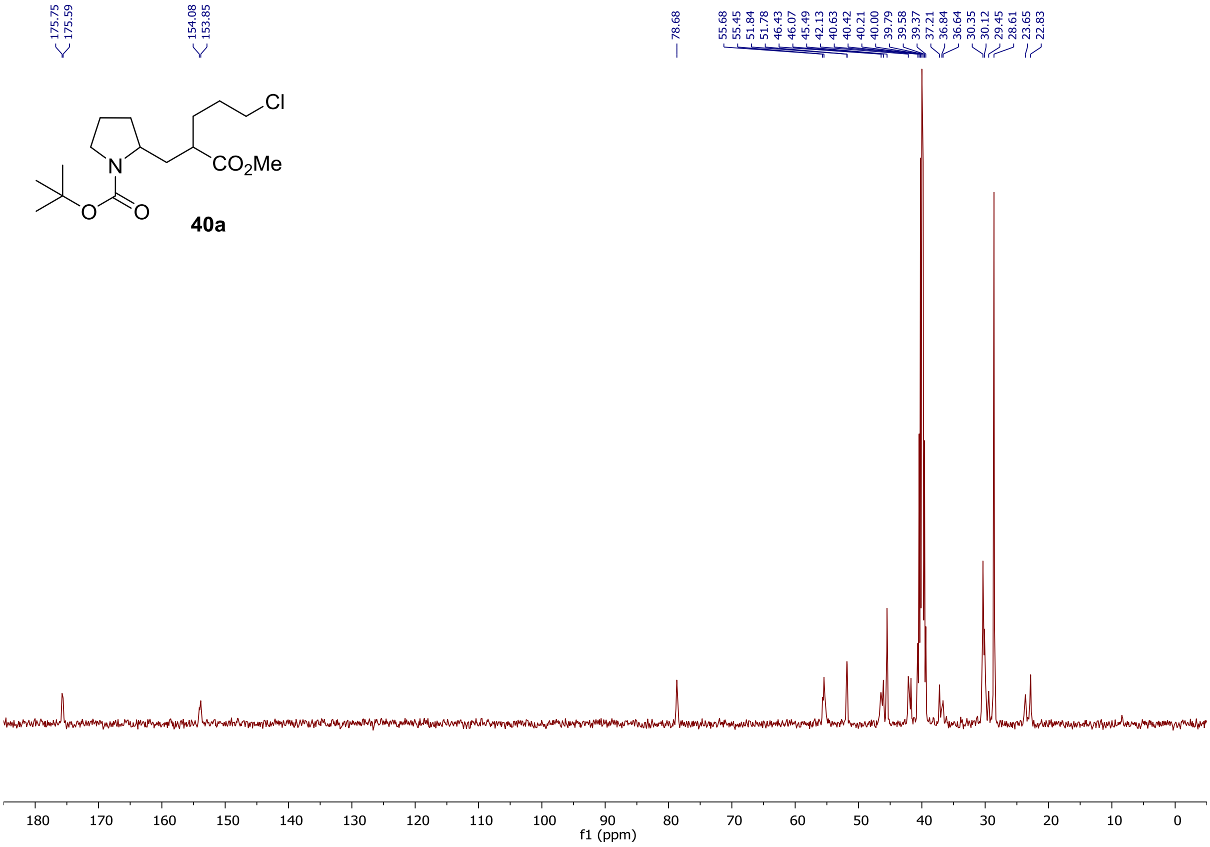

^{1}H NMR (400 MHz, $CDCl_3$)

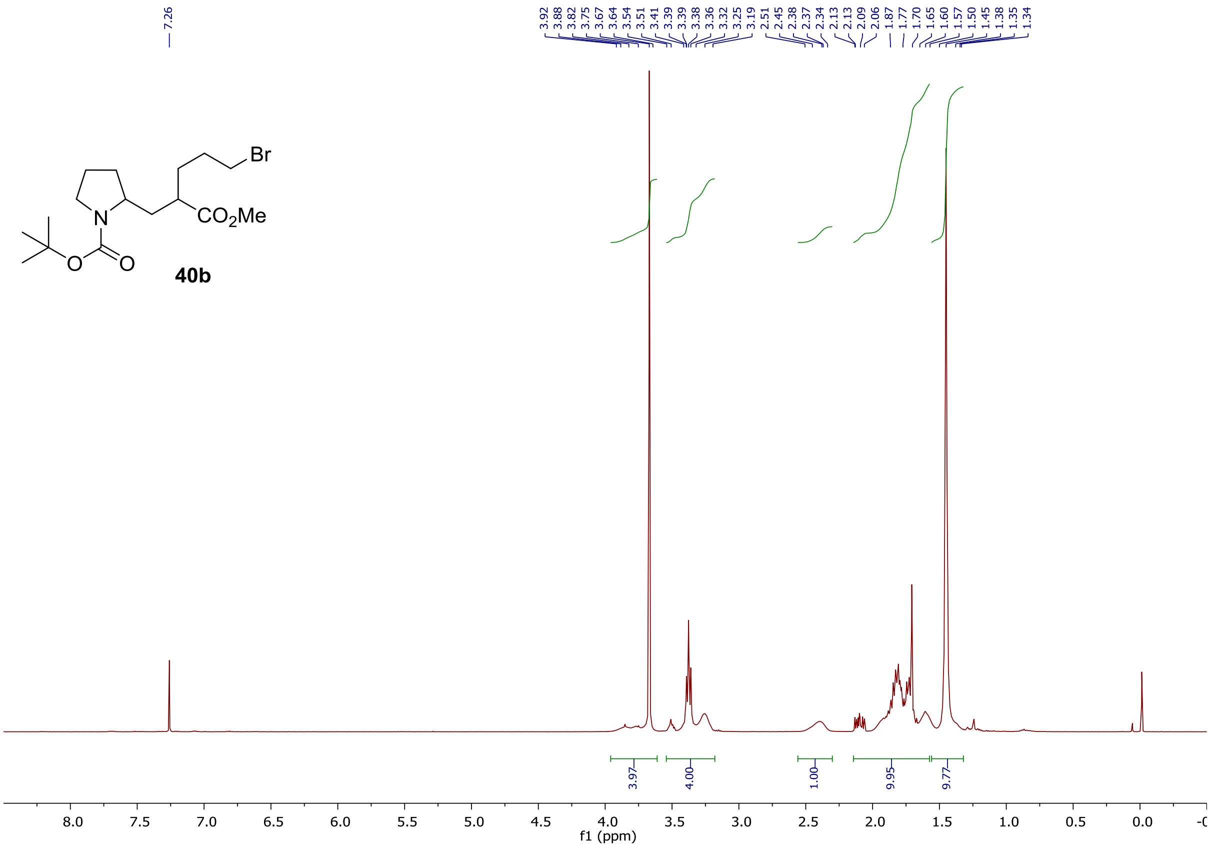

^{13}C NMR (101 MHz, $CDCl_3$)

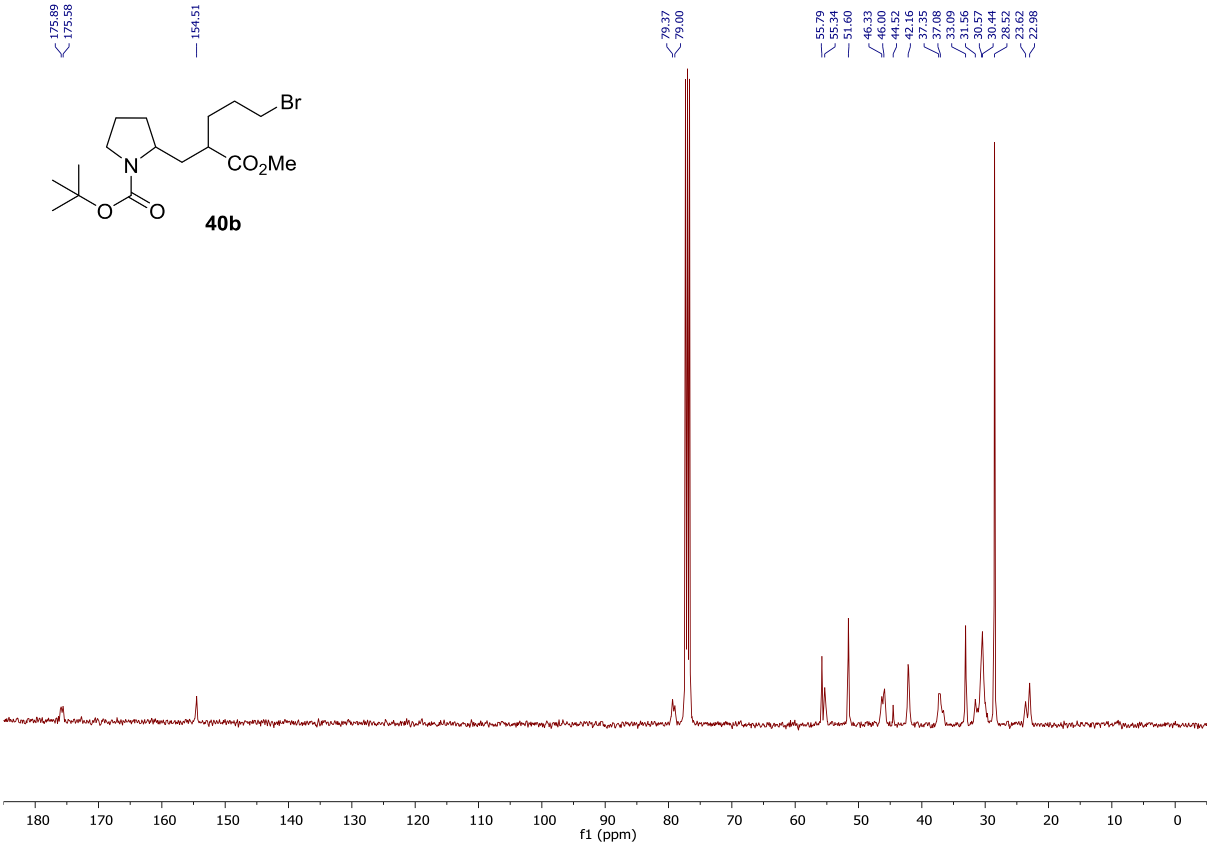

^{1}H NMR (400 MHz, $CDCl_3$)

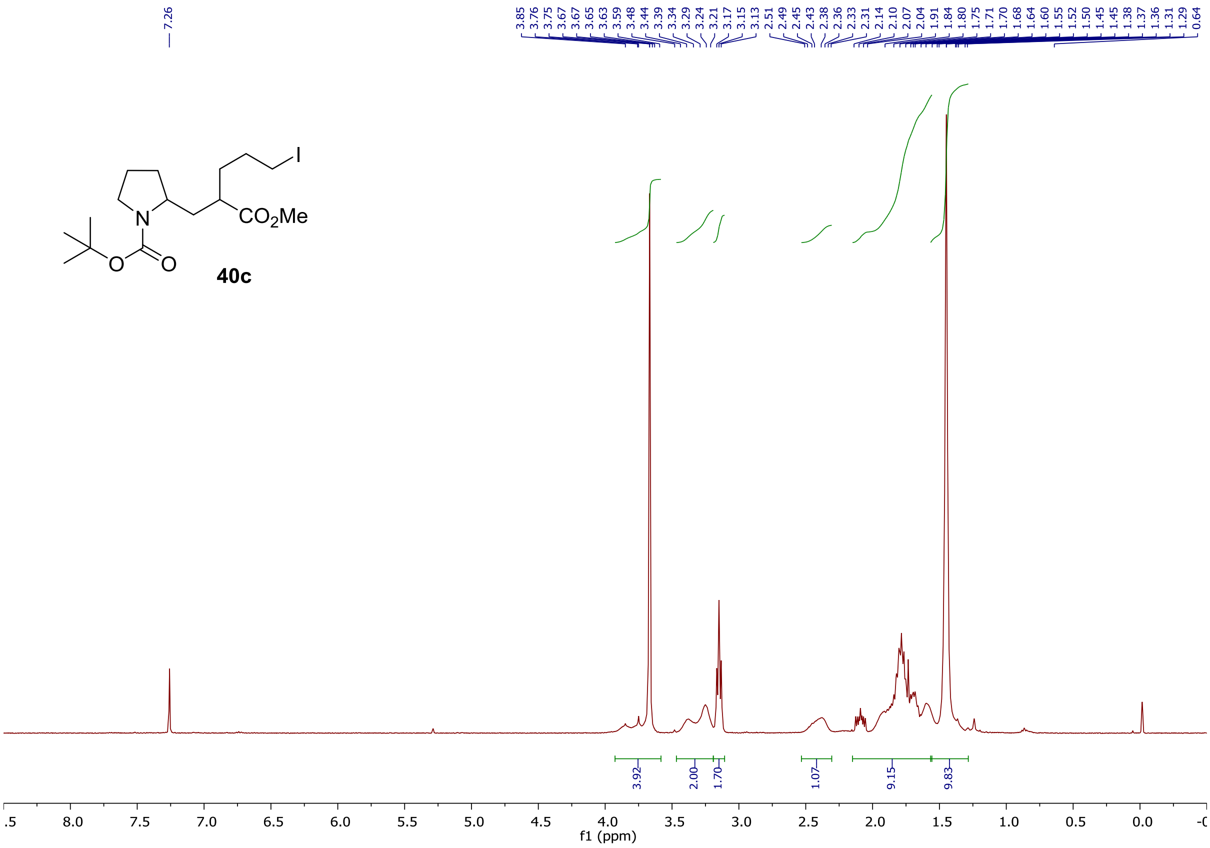

^{13}C NMR (101 MHz, $CDCl_3$)

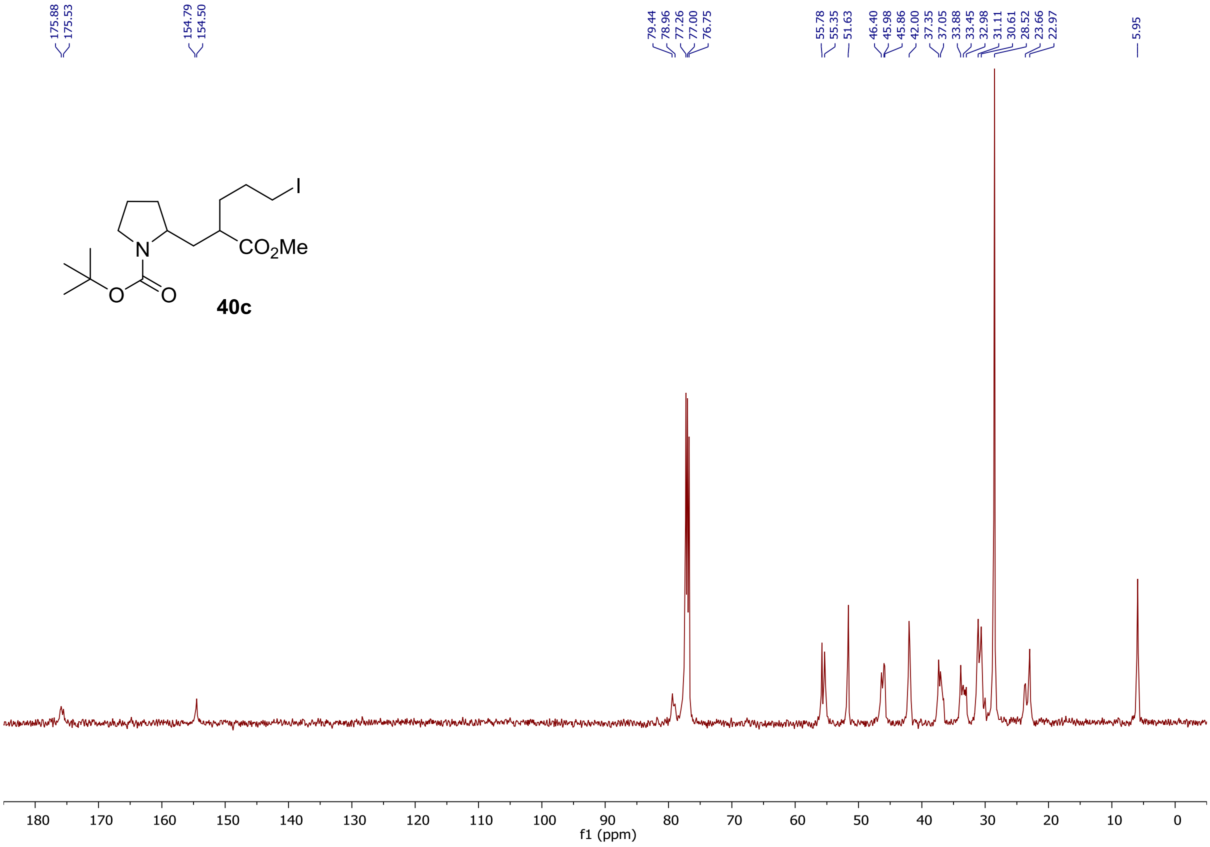

^{1}H NMR (400 MHz, $CDCl_3$)

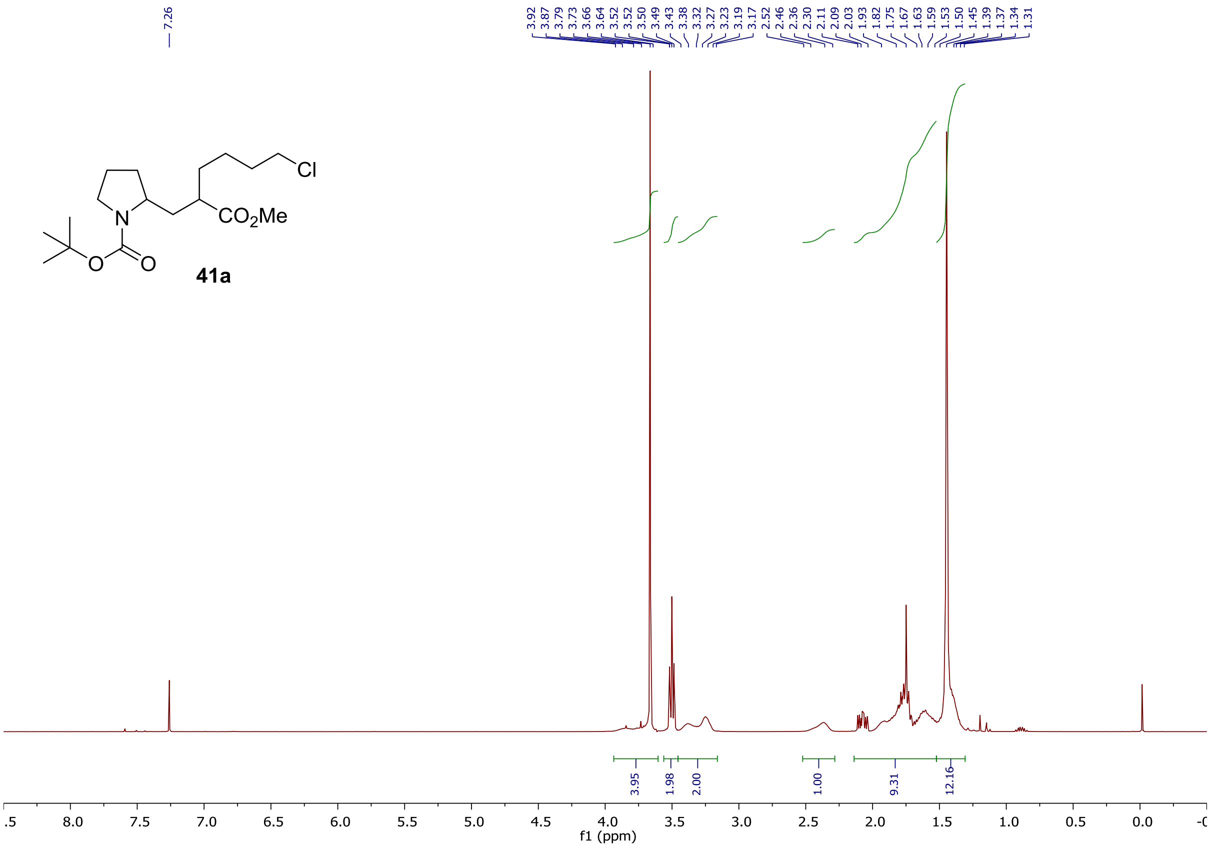

^{13}C NMR (101 MHz, $CDCl_3$)

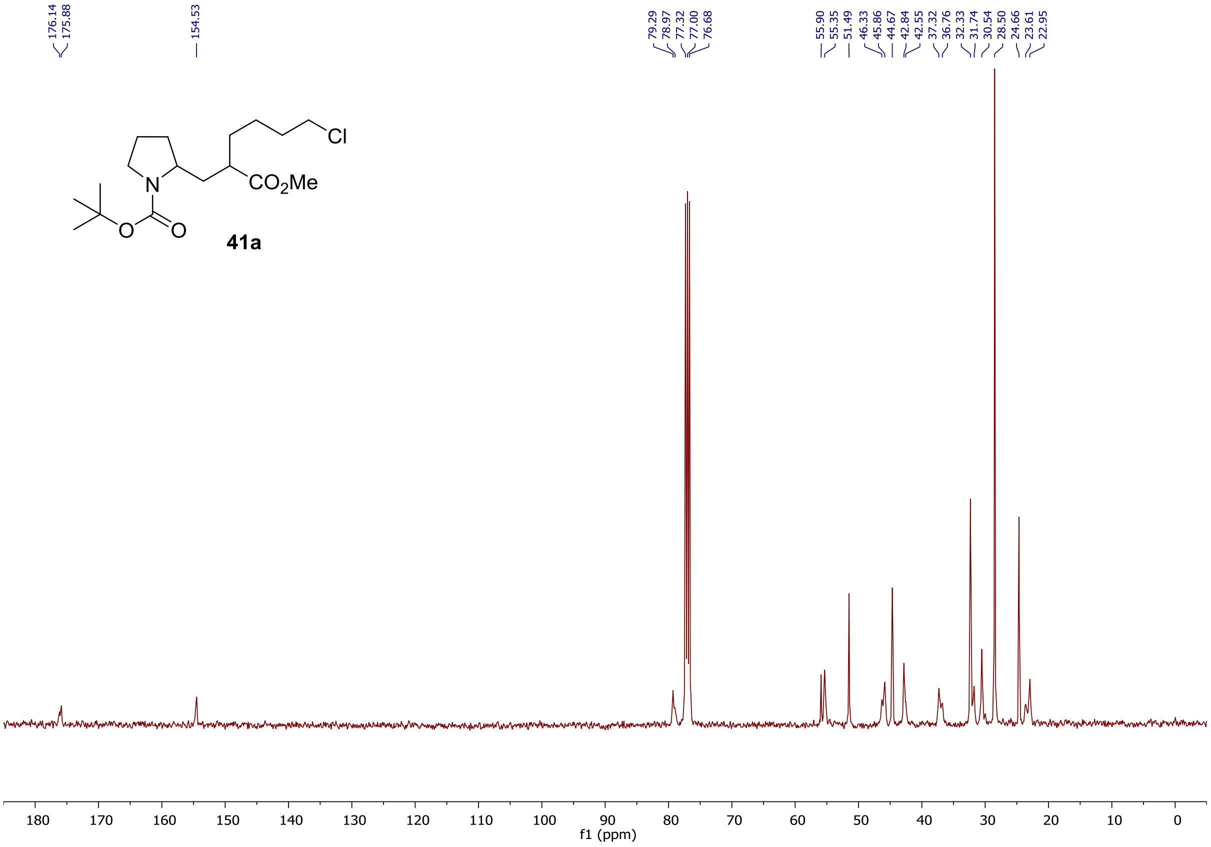

^{1}H NMR (400 MHz, $CDCl_3$)

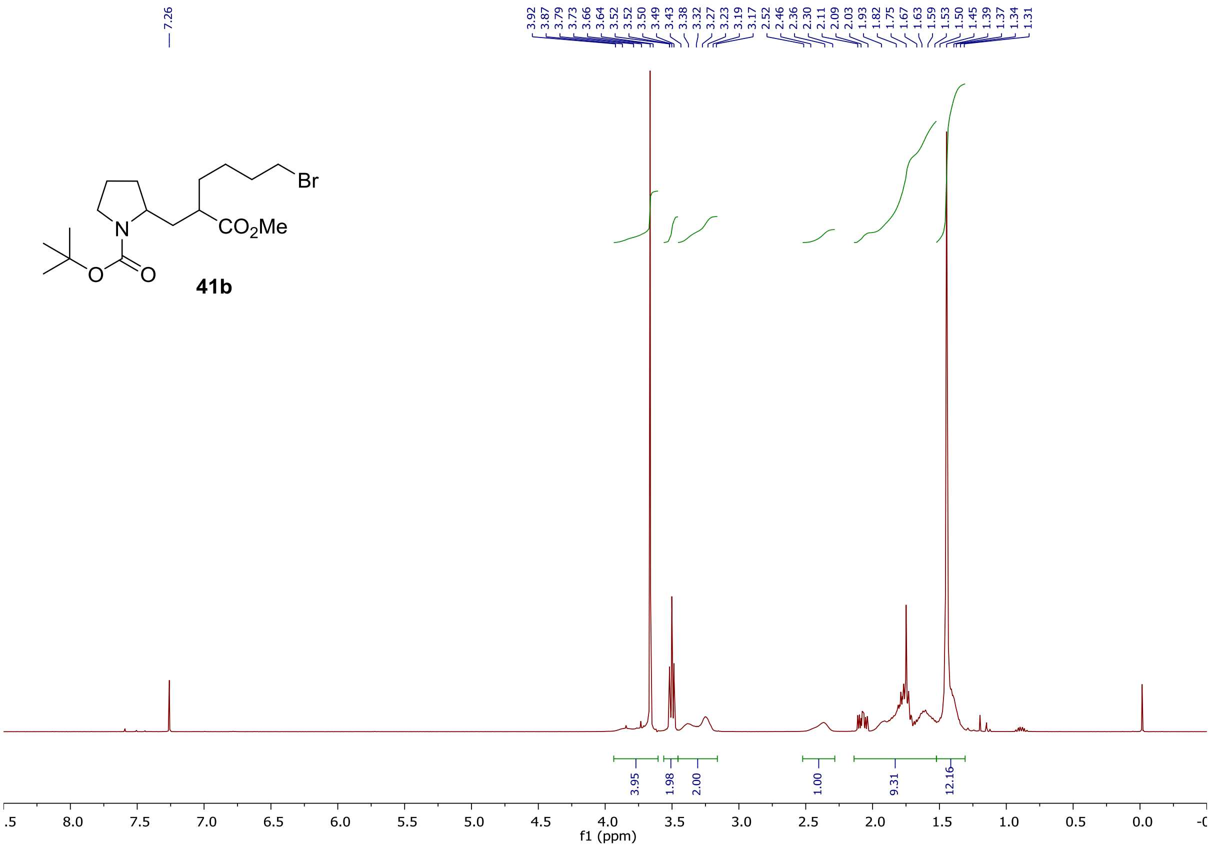

^{13}C NMR (101 MHz, $CDCl_3$)

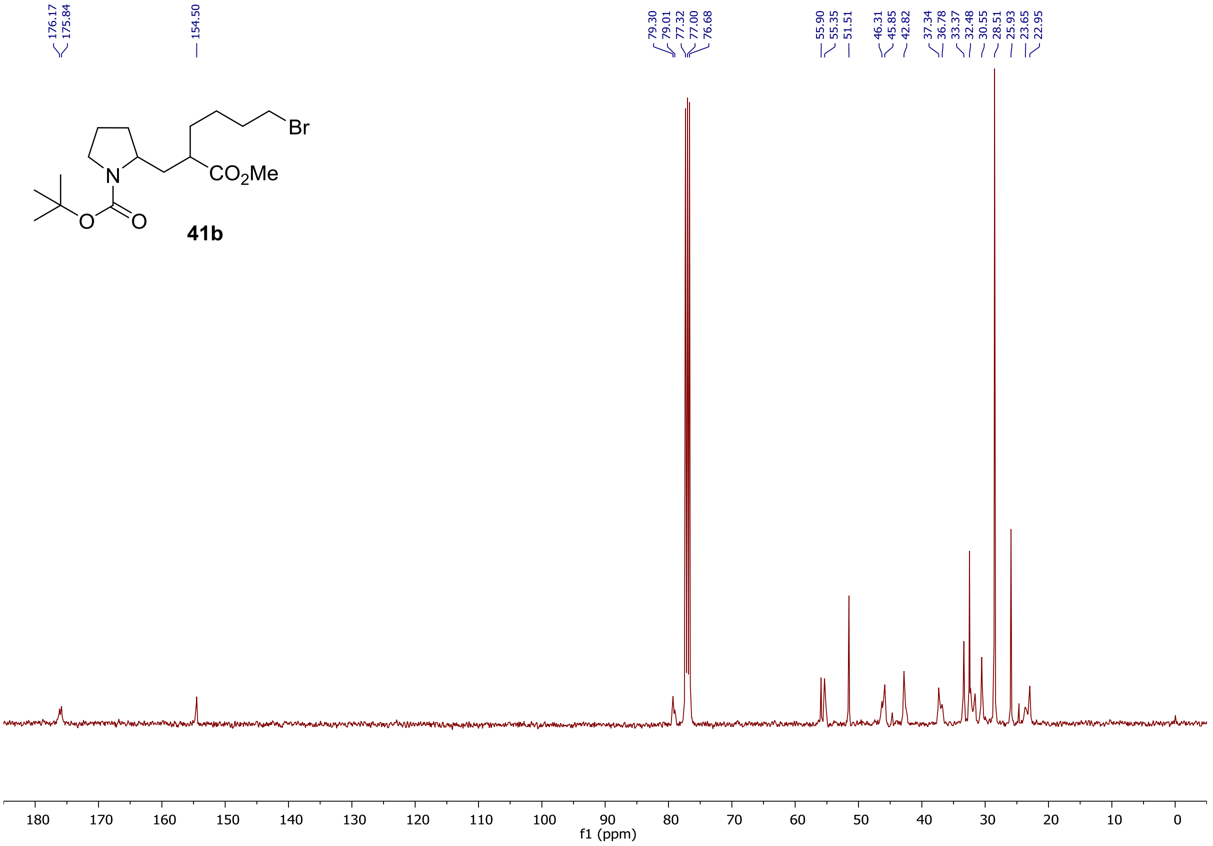

^{1}H NMR (400 MHz, $CDCl_3$)

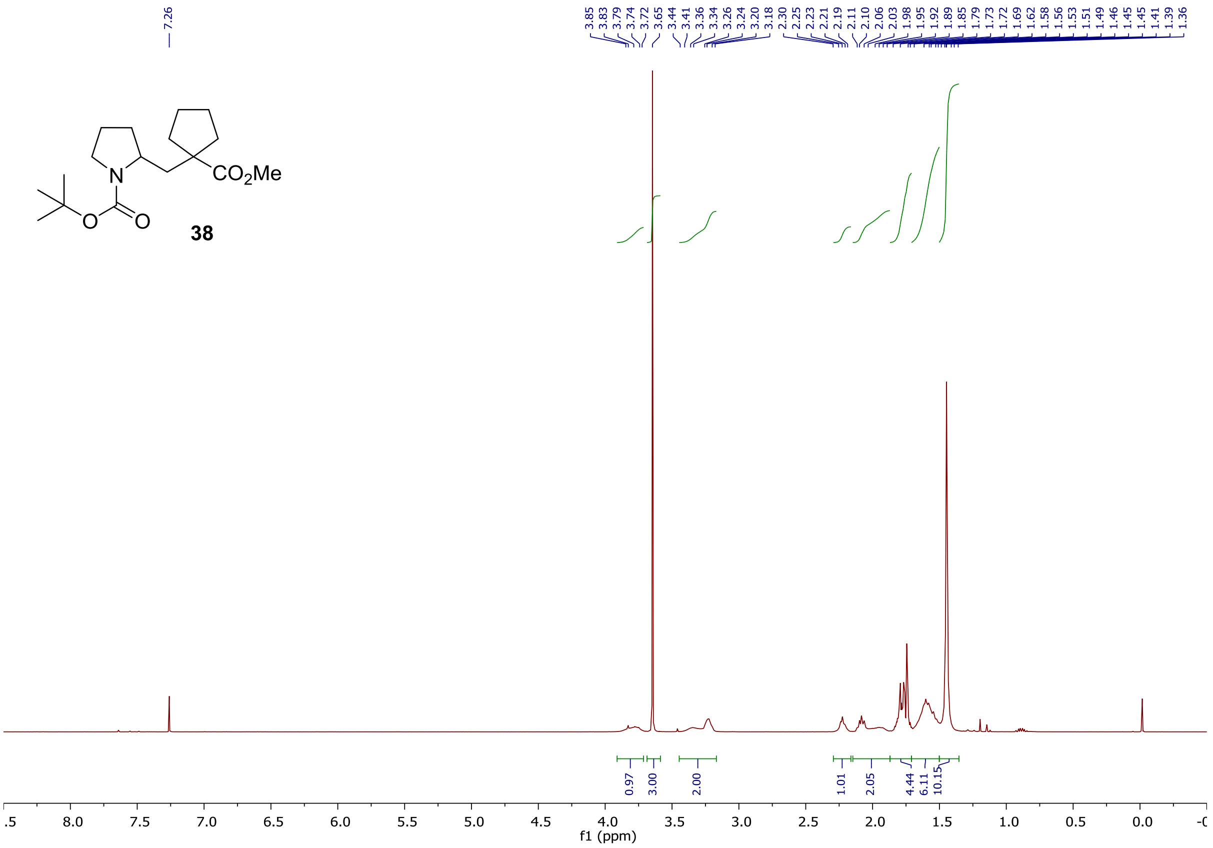

^{13}C NMR (101 MHz, $CDCl_3$)

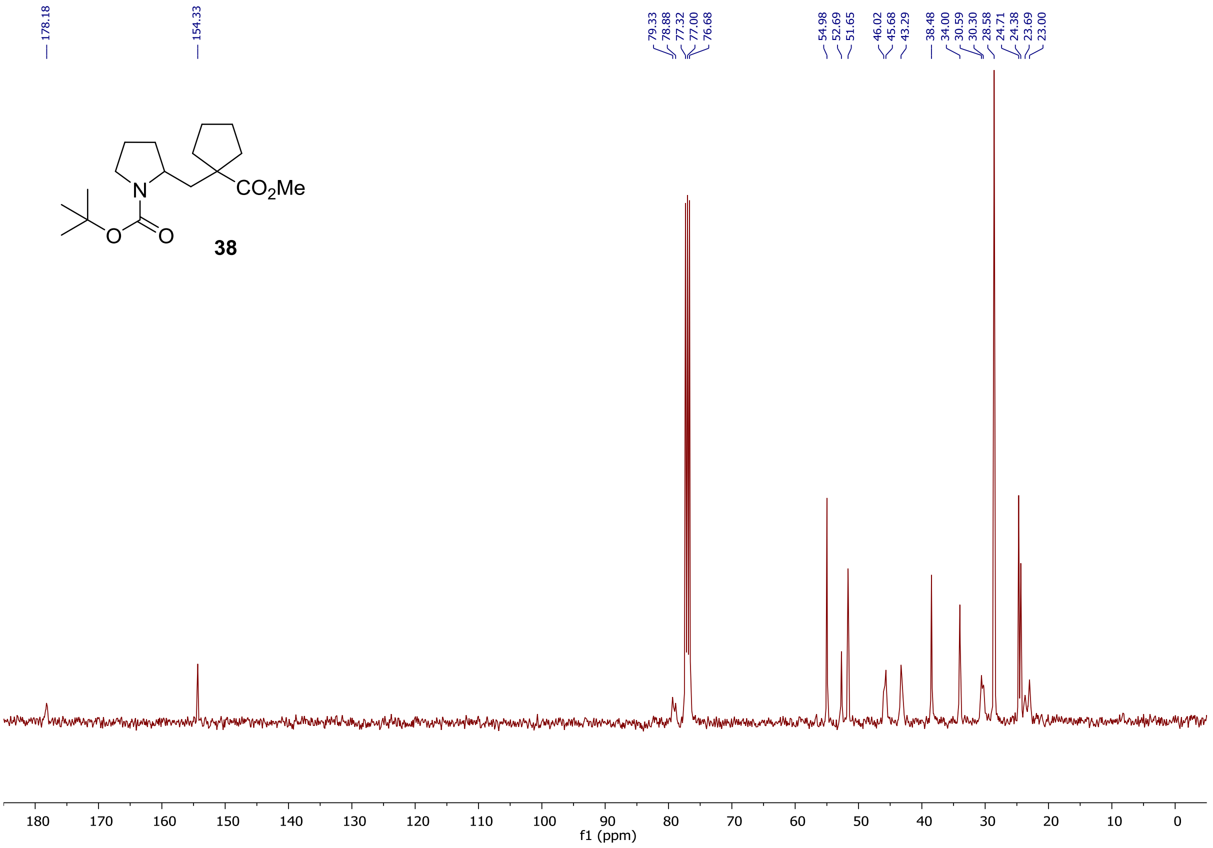

^{1}H NMR (400 MHz, $CDCl_3$)

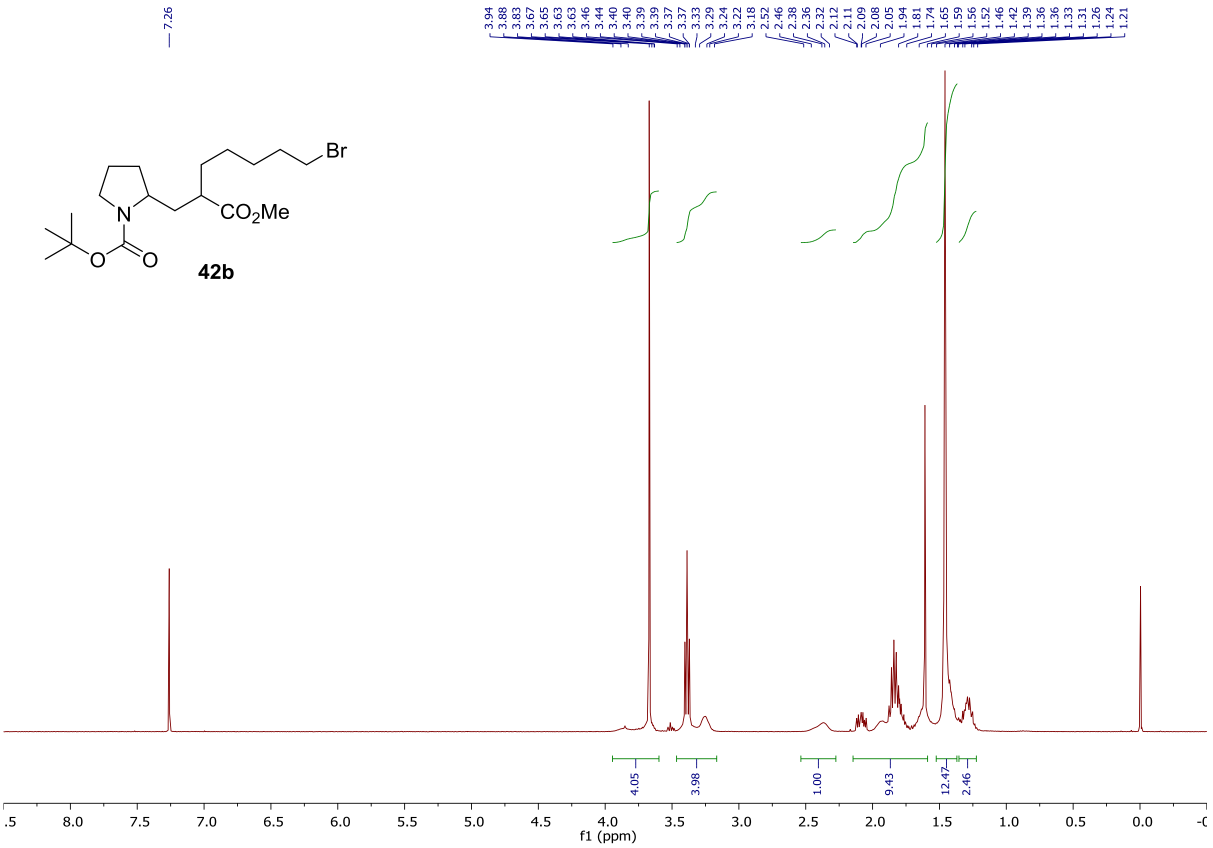

^{13}C NMR (101 MHz, $CDCl_3$)

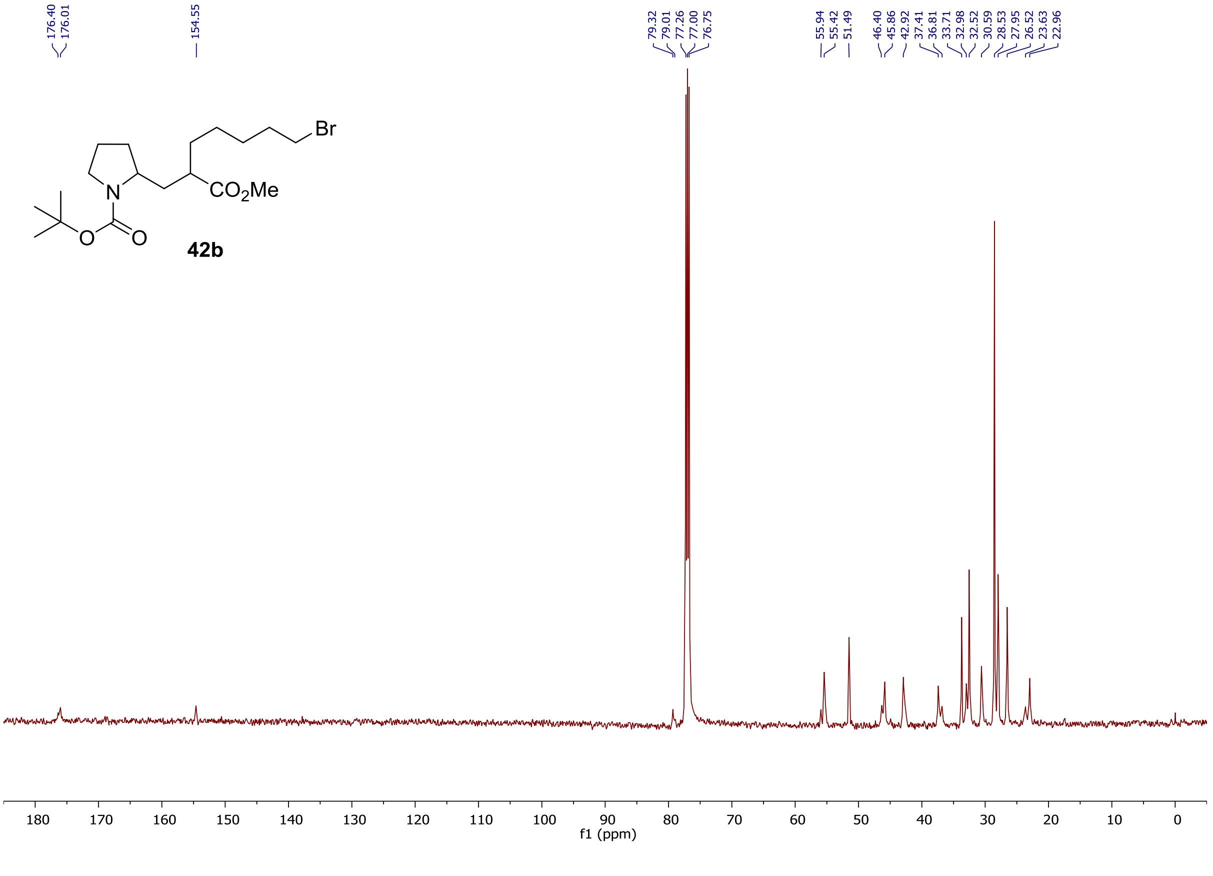

^{1}H NMR (400 MHz, $CDCl_3$)

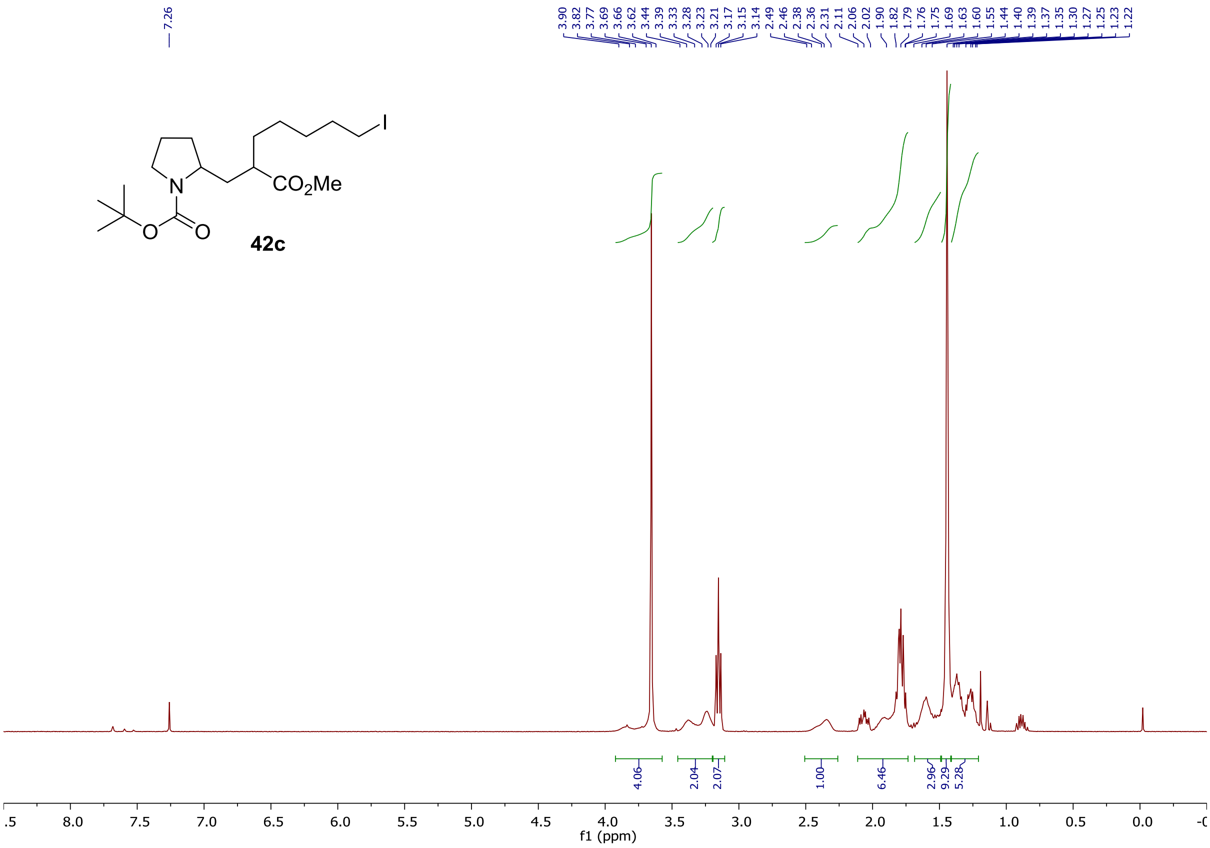

^{13}C NMR (101 MHz, $CDCl_3$)

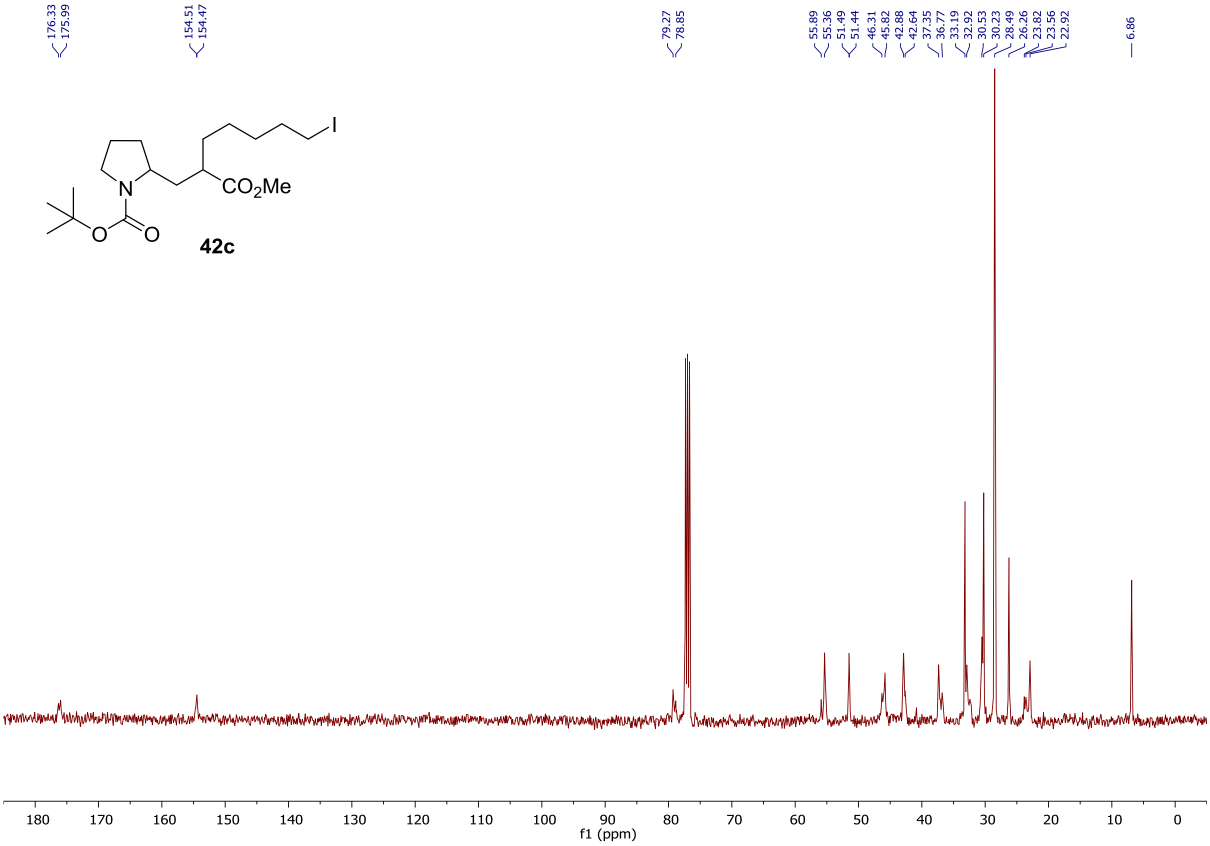

^{1}H NMR (400 MHz, $CDCl_3$)

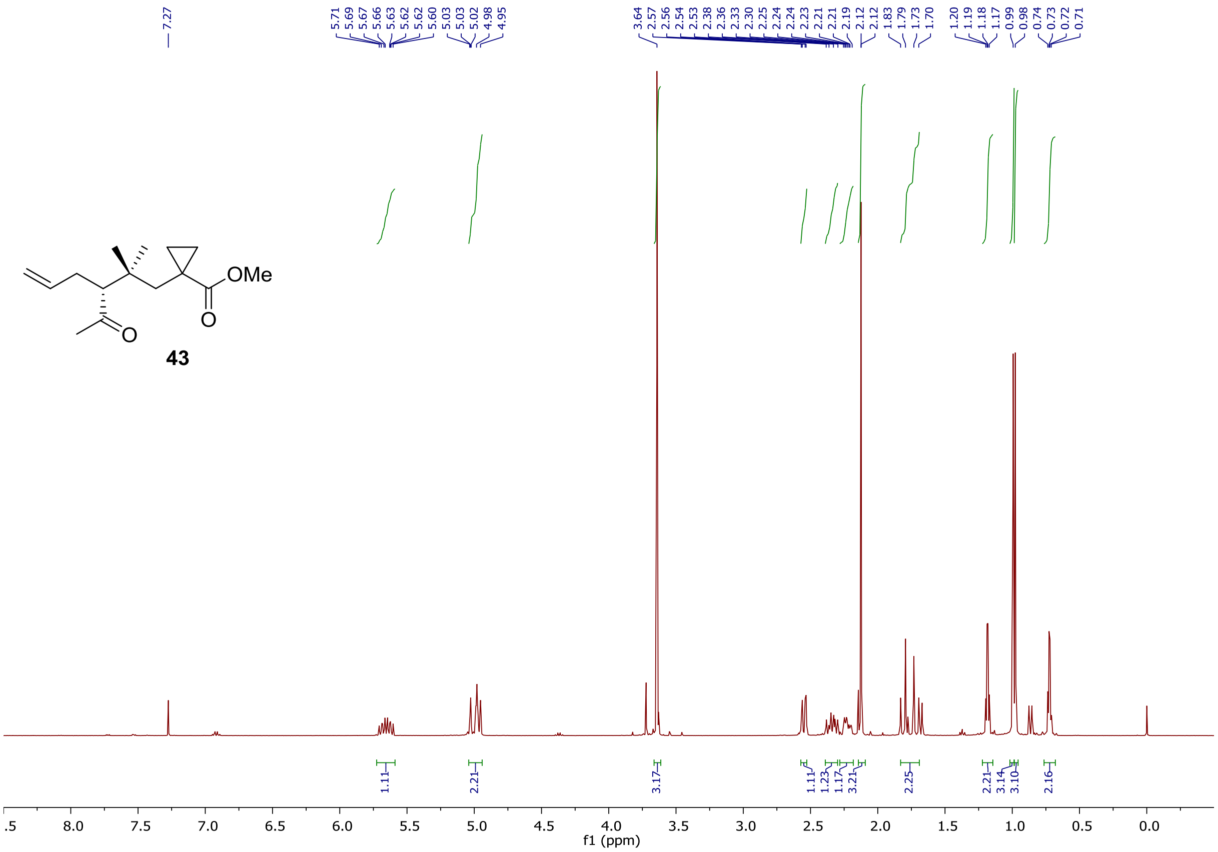

^{13}C NMR (101 MHz, $CDCl_3$)

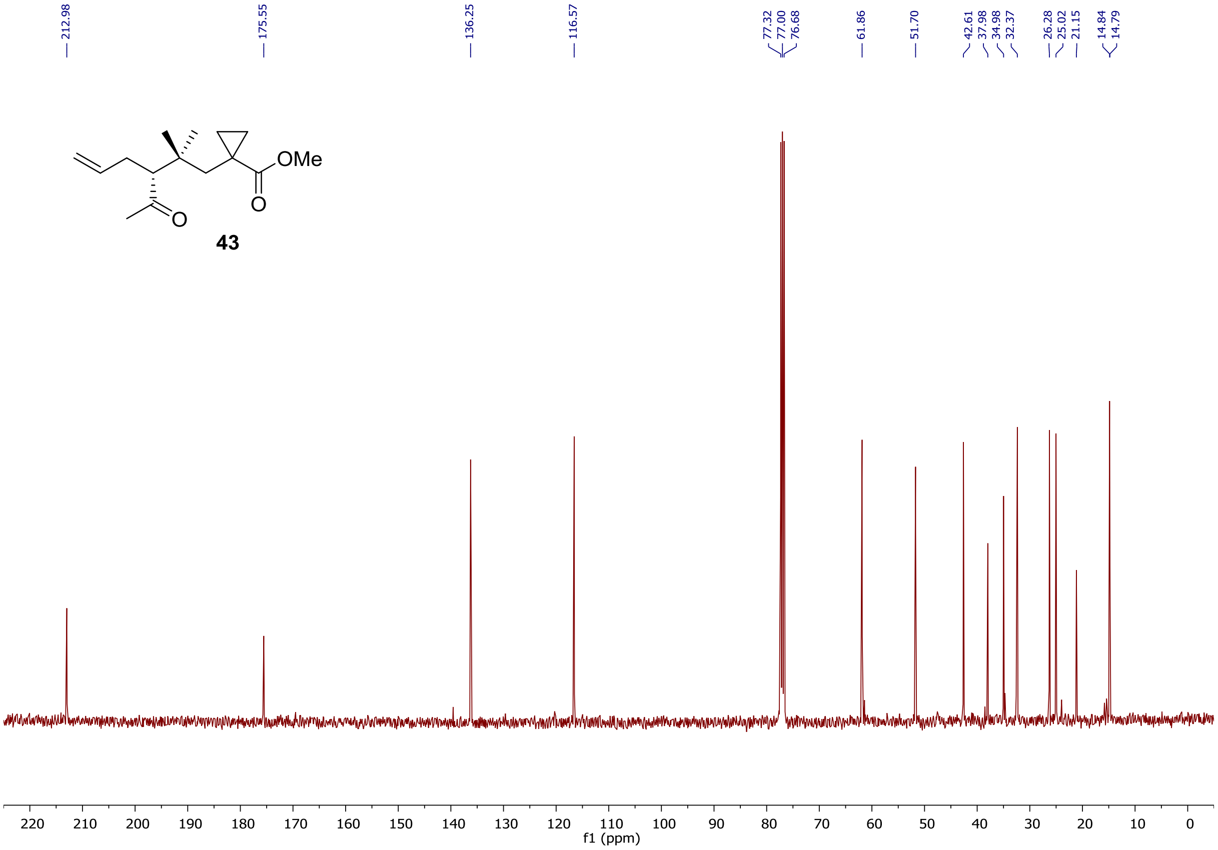

^{1}H NMR (400 MHz, $CDCl_3$)

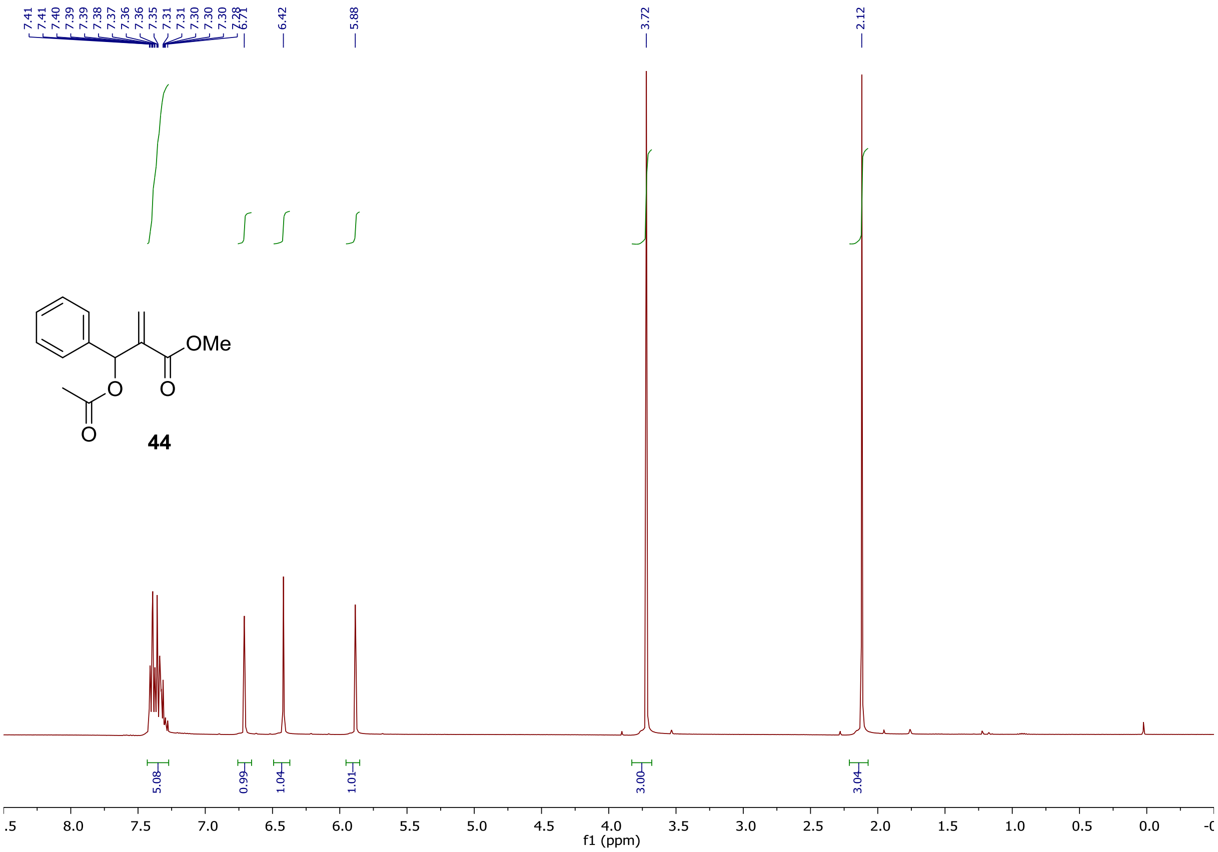

^{13}C NMR (101 MHz, $CDCl_3$)

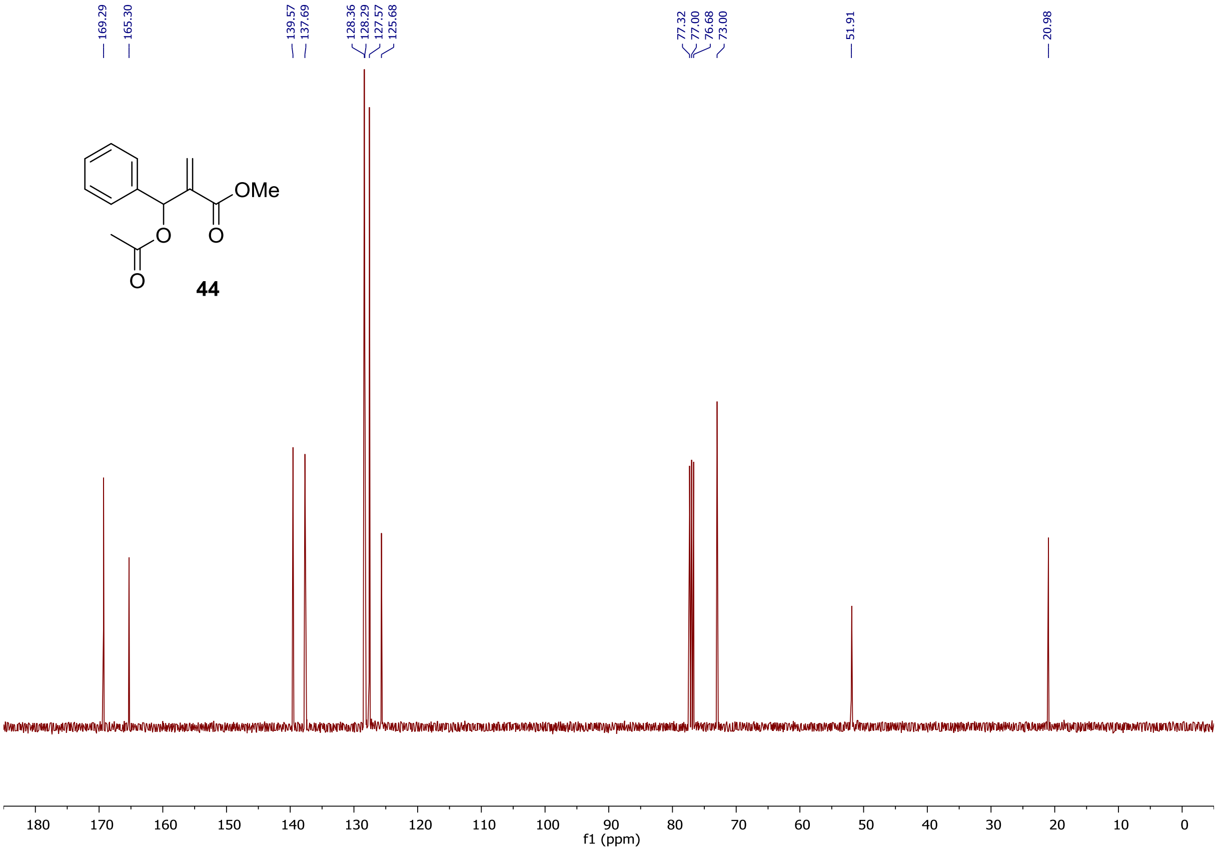

^{1}H NMR (500 MHz, DMSO-d_6, 100 °C)

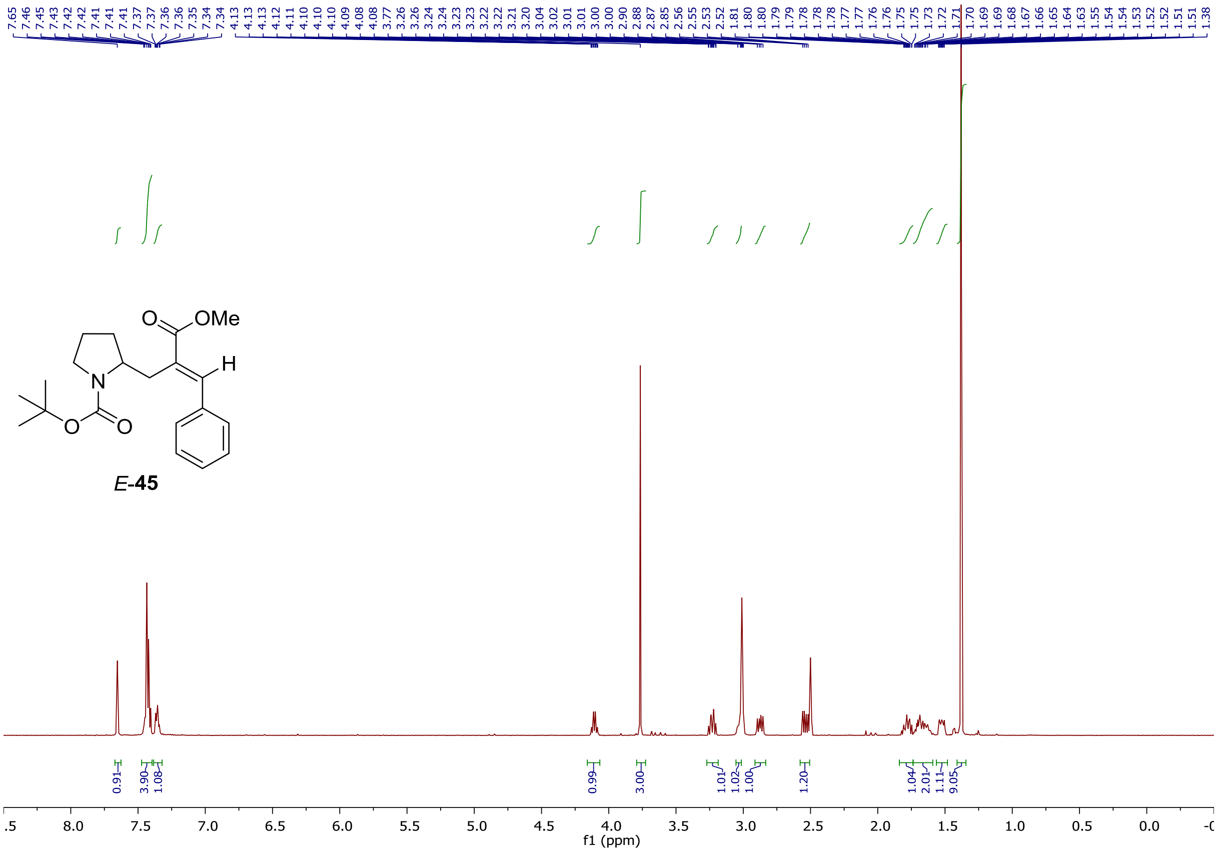

^{13}C NMR (126 MHz, DMSO-d_6, 100 °C)

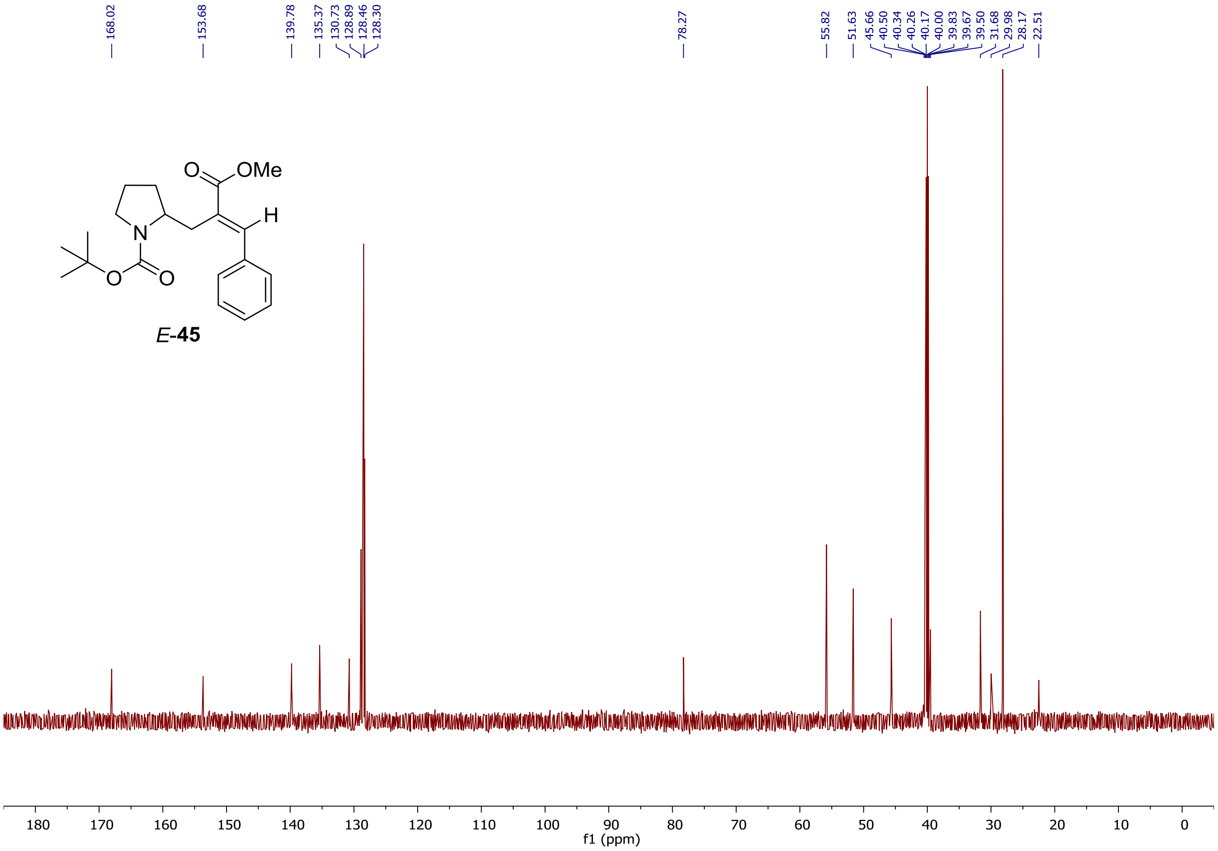

^{1}H NMR (500 MHz, DMSO-d_6, 100 °C)

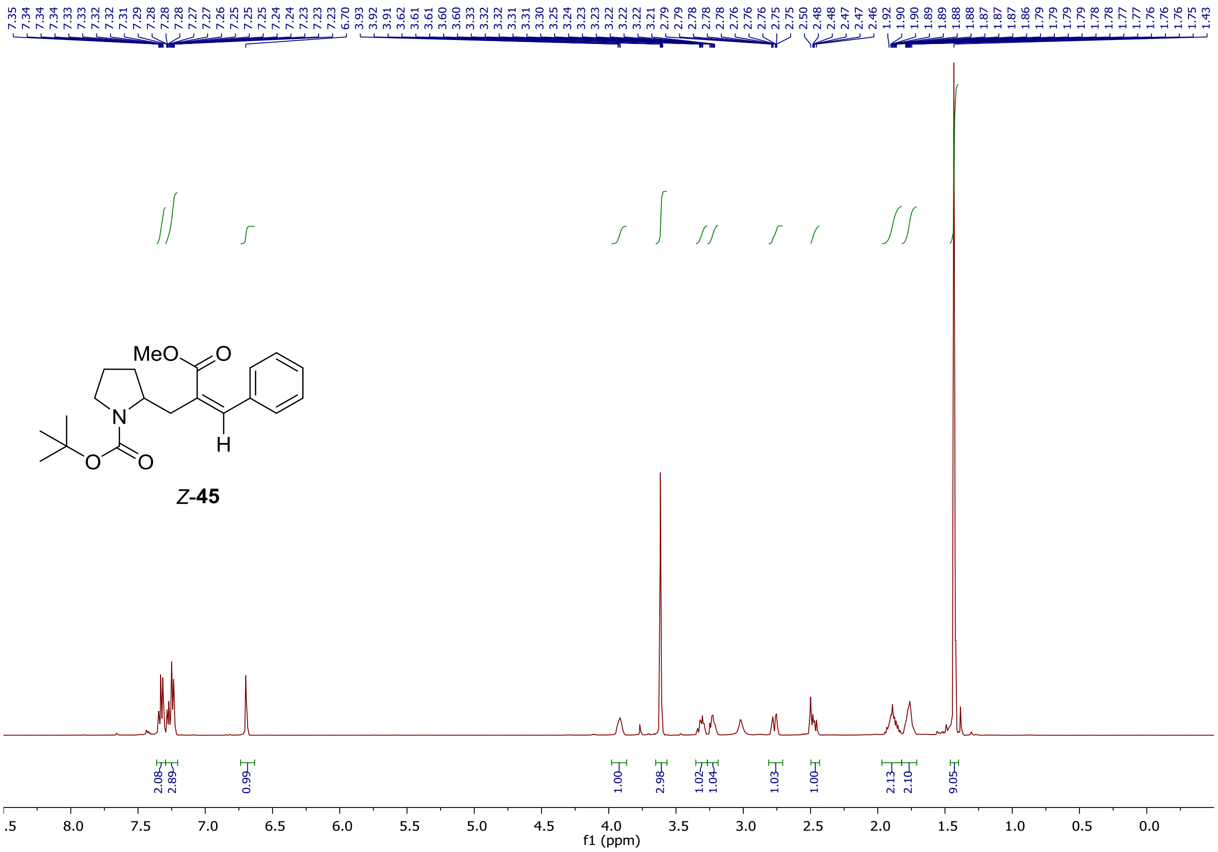

^{13}C NMR (126 MHz, DMSO-d_6, 100 °C)

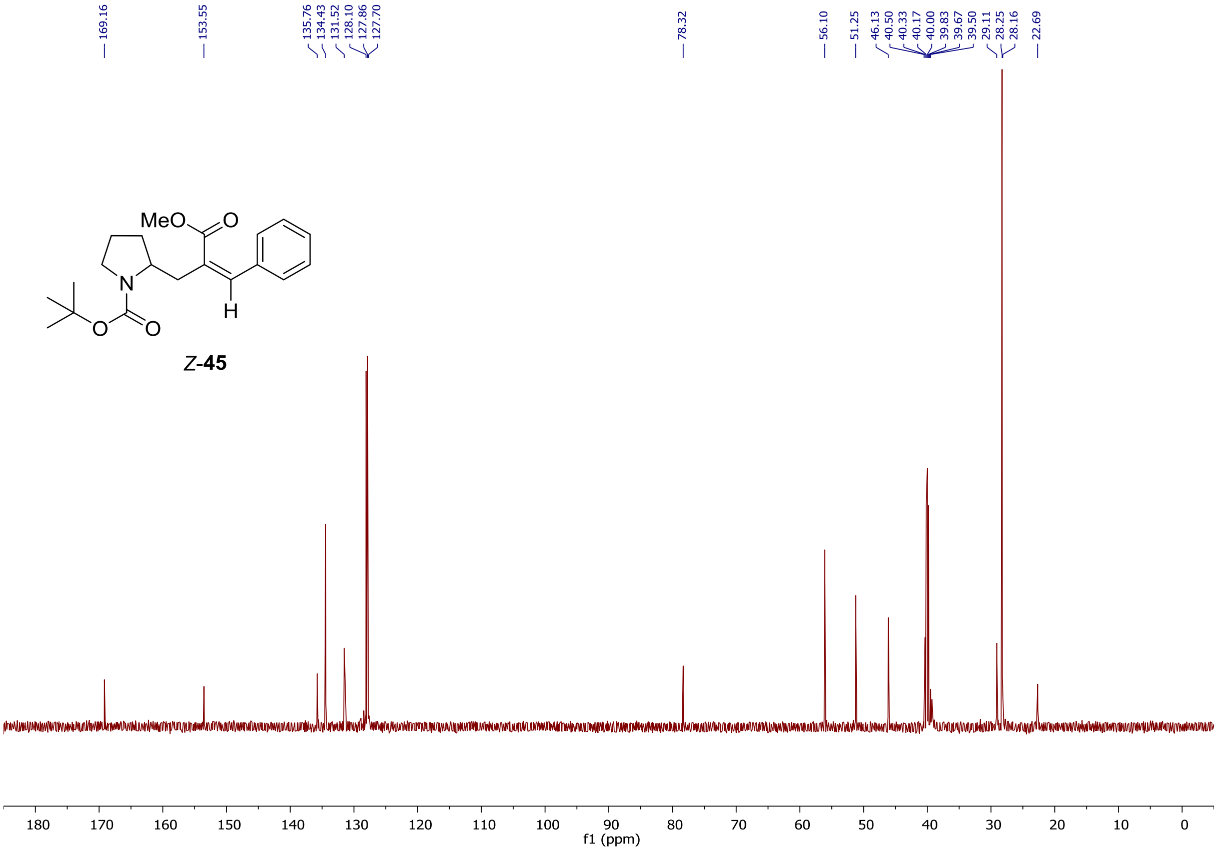

^{1}H NMR (400 MHz, $CDCl_3$)

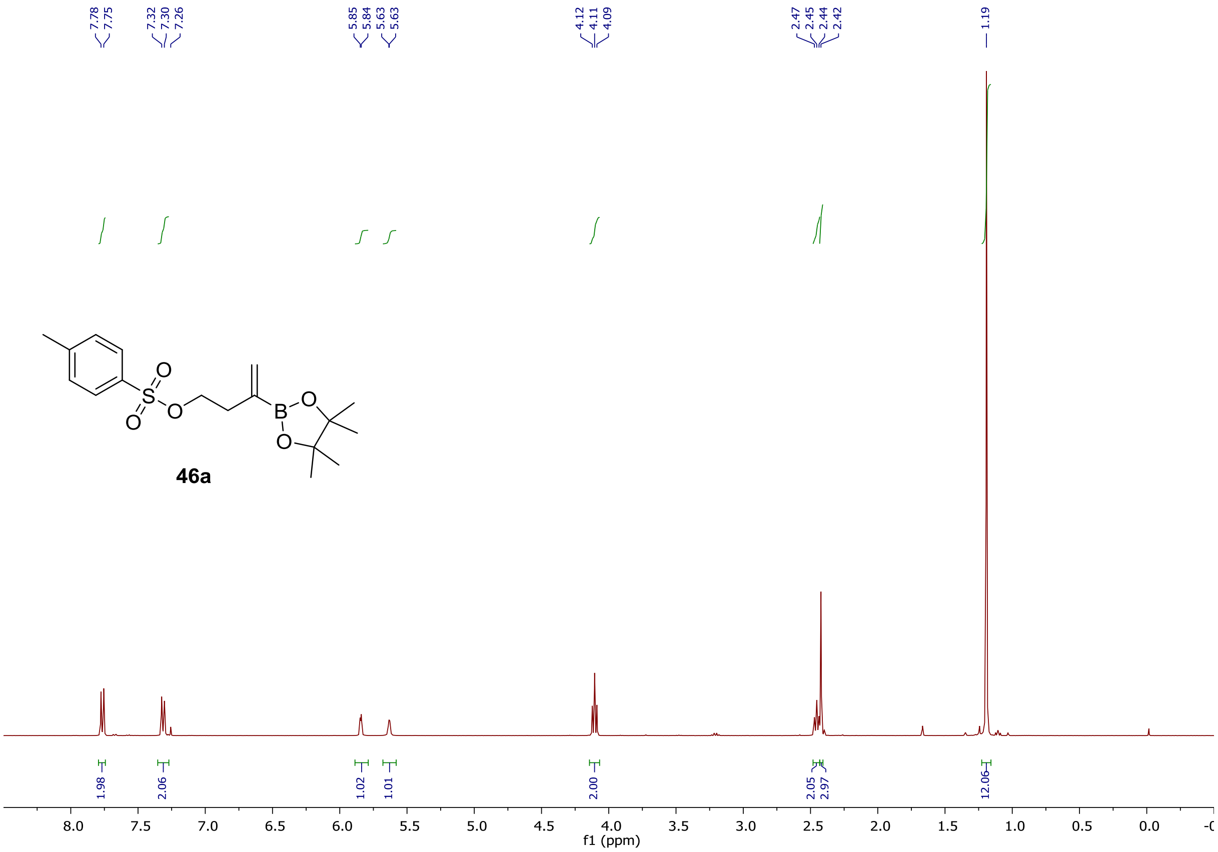

^{13}C NMR (101 MHz, $CDCl_3$)

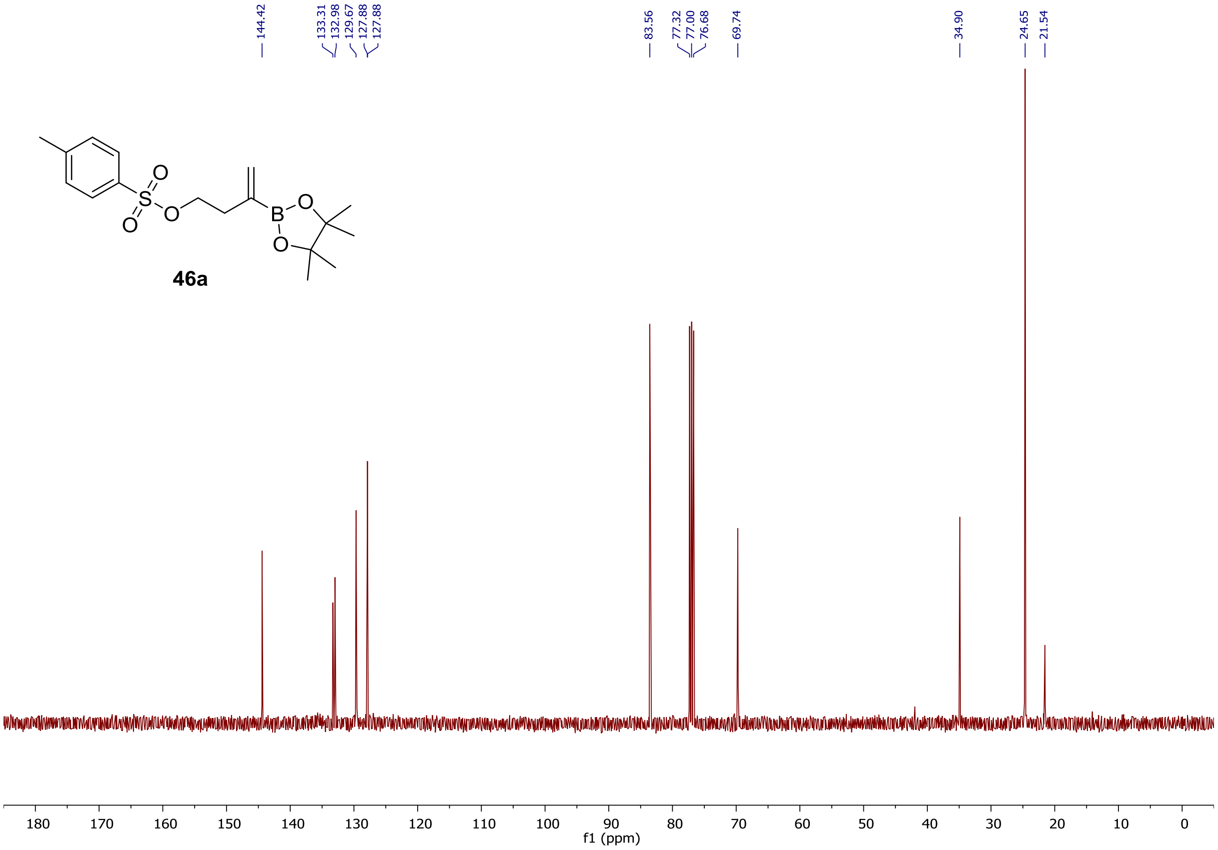

^{1}H NMR (400 MHz, $CDCl_3$)

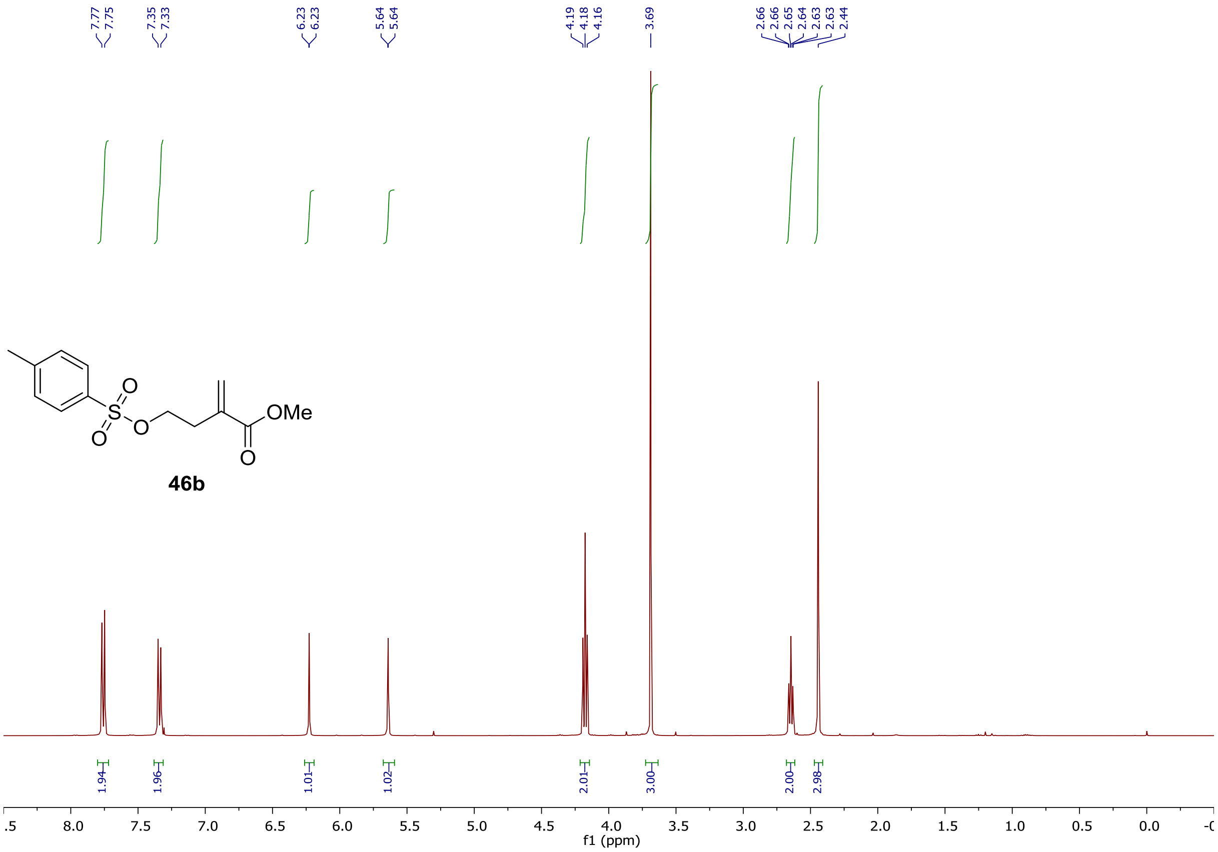

^{13}C NMR (101 MHz, $CDCl_3$)

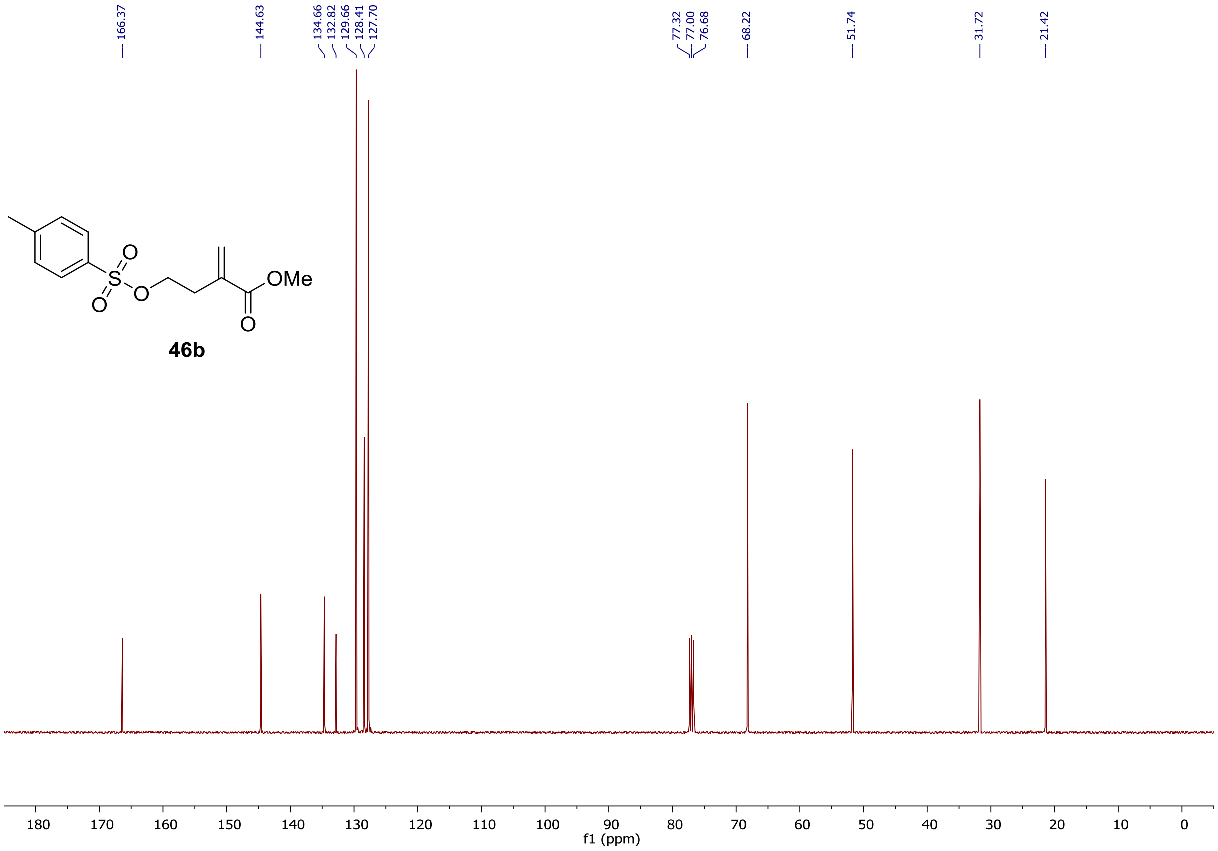

Supplement: Supplementary file 1 — Supplementary [file ANIE-57-15430-s001.pdf]
